# Supplementary material for: Street-wise dog testing: Feasibility and reliability of a behavioural test battery for free-ranging dogs in their natural habitat
Source: PLoS One. 2024 Mar 14;19(3):e0296509. doi: 10.1371/journal.pone.0296509 (PMC10939227; doi:10.1371/journal.pone.0296509)
Supplement: S2 File — (PDF) [file pone.0296509.s003.pdf]

S2 Equipment information

For visual information on the equipment of the Fake dog and Novel Object subtest, please see the website of the manufacturers:

- **Fake Dog** (Melissa & Douk): https://www.amazon.de/Melissa-Doug-Jack-Russell-Terrier-Naturgetreues-Gesichtsausdruck/dp/B003NSBKZ0?ref_=ast_sto_dp&th=1

- **Remotely controlled car** (Deerc): https://store.deerc.com/products/deerc-de42-remote-control-car-rc-racing-cars-1-18-scale-80-min-play-2-4ghz-led-light-auto-mode-off-road-rc-trucks-with-storage-case-all-terrain-suv-jeep-cars-toys-gifts-for-boys-kids-girls-teens-blue
- **Number foil balloon** (Carrefour Maroc): https://www.carrefour.fr/p/ballon-helium-xxl-80-cm-chiffre-0-argent-gris-numero-anniversaire-3663645086609?s=2850
- **Dinosaur foil balloon** (type: Tyrannosaurus; bought through Tedy - Austria): https://www.amazon.de/ballonfritz%C2%AE-Dinosaurier-Ballon-Geburtstagsgeschenk-Kindergeburtstag/dp/B0947GMNJM?th=1
