# Supplementary material for: Street-wise dog testing: Feasibility and reliability of a behavioural test battery for free-ranging dogs in their natural habitat
Source: PLoS One. 2024 Mar 14;19(3):e0296509. doi: 10.1371/journal.pone.0296509 (PMC10939227; doi:10.1371/journal.pone.0296509)
Supplement: S1 Table — (DOCX) [file pone.0296509.s004.docx]

**S3 Table. Detailed Ethogram.** The behaviours are presented by category if applicable, with a detailed description and the unit in cursive in which they were measured. In addition, it is indicated in which subtest(s) the behaviour was coded and towards which modifier(s). Subtests include the Human approach (HA), Fake dog (FD), Novel object (NO), Pointing (P), Begging (B), and Tractability (T) subtest. Modifiers are the main experimenter (E1), both experimenters (E1+2), the fake dog (FD), the novel object (NO), the bowl, and the stool. If the behaviour was coded in the test in general, it was marked as ‘yes’. If the behaviour was not coded in the subtest, it was indicated with a minus (-).

| **Category** | **Behaviour** | **Definition** | **Applicable subtest** | | | | | |
| --- | --- | --- | --- | --- | --- | --- | --- | --- |
|  |  |  | **HA** | **FD** | **NO** | **P** | **B** | **T** |
| Proximity *(Duration)* | Close proximity | Time within 1 body length of person: As soon as paw gets within 1 body length of person (actively or passively) TO last paw leaves distance of 1 body length of person | E1 | FD  E1+2 | NO  E1+2 | - | E1 | E1  Stool |
|  | Medium proximity | Time within 2 body length of person | E1 | FD  E1+2 | NO  E1+2 | - | E1 | E1  Stool |
| Tail  *(Duration)* | Wagging | Tail is actively moved from side to side (not just swinging with body movement) | E1 | FD  E1+2 | NO  E1+2 | - | E1 | E1  Stool |
|  | Between the legs | Tail is between the hind legs or below the belly | Yes | Yes | Yes | - | Yes | Yes |
| Gazing *(Duration)* | Gazing | Head oriented towards the stimulus | E1 | FD E1+E2 | NO  E1+E2 | - | E1 | E1  Stool |
| Vocalization | Barking *(Duration)* | Loud and short typical vocalization, which may be repetitive. | E1 | FD | NO | - | E1 | E1 |
|  | Growling *(Frequency)* | Actor snarls or growls at the specific receiver (only!) the actor's body posture being rigid. Mouth may be open, nose can be wrinkle. | E1 | FD | NO | - | E1 | E1 |
|  | Whining *(Duration)* | The dog making persistently whines. | E1 | FD | NO | - | E1 | E1 |
| Displacement behaviours | Auto-grooming *(Frequency)* | Scratching, biting or licking of the dog’s own body parts. | Excl. | Excl. | Excl. |  | Excl. | Excl. |
|  | Body Shaking *(Frequency)* | Dog's whole body starts moving rapidly, from side to side while the dog stands. | Yes | Yes | Yes | - | Yes | Yes |
|  | Stretching *(Frequency)* | The whole body is stretched in various ways: forelegs may be bent down while the dog's back is arched; hind legs may be straightened while the head is held up high | Yes | Yes | Yes | - | Yes | Yes |
|  | Yawning *(Frequency)* | The mouth is wide open, the neck is stretched. | Yes | Yes | Yes | - | Yes | Yes |
|  | Nose /mouth licking *(Frequency)* | The mouth is slightly open, and the dog licks its upper lip or nose, on the right or on the left | Yes | Yes | Yes | - | Yes | Yes |
|  | Sniffing the ground  *(Duration)* | Nose moving across the ground, head facing down instead of forward | Yes | Excl. | Excl. | - | Excl. | Excl. |
| Physical contact | Biting *(Frequency)* | The dog aggressively (!) bites another subject in the skin and fur. Includes bite-shaking. Aggressively meaning other signs of aggression such as growling, teeth baring, lunging have been shown during/before | E1 | FD | NO | - | E1 | E1 |
|  | Body contact  *(Duration)* | Dog touches or is touched by person (or brush) with any parts of its body | E1 | FD | NO | - | E1 | E1 |
|  | Jumping *(Frequency)* | Dog jumps on or towards person: both front paws off the ground, both hind paws on the ground, front of body higher than back part of body, movement vertically towards person/wall/object | E1 | FD | NO | - | E1 | E1 |
|  | Licking *(Duration)* | Dog using its tongue to touch the person | E1 | FD | NO | - | E1 | E1 |
|  | Mouthing *(Duration)* | Dog takes part of the person/their clothes/object into its mouth without aggression | E1 | FD | NO | - | E1 | E1 |
|  | Sniffing person/object *(Duration)* | Nose close to the person/object | E1 | FD | NO | - | E1 | E1 |
|  | Pawing *(Frequency)* | Dog extends one paw towards person/object (distance less than 10 cm), the other is still on the ground | E1 | FD | NO | - | E1 | E1 |
| Reactions | Stand tall *(Duration)* | Straightens up to full height, with a rigid posture, ears erect and tail perpendicular or above the back. Standing or walking. May include raised hackles. May include T-pose towards fake dog (approaches another’s shoulder/back/head and puts its head on it, typically forming a capital “T”) | E1 | FD | NO |  | E1 | E1 |
|  | Bare teeth *(Frequency)* | Actor bares its canines, or generally curls the lips. | E1 | FD | NO | - | E1 | E1 |
|  | Lunge  *(Frequency)* | Actor moves abruptly and rapidly towards recipient. Can but doesn’t have to result in physical contact | E1 | FD | NO | - | E1 | E1 |
|  | Head dip  *(Frequency)* | Lowers his/her head in response to the receiver either reducing the distance between, displaying dominant/aggressive behaviors, or orienting towards them. During the displaying of this behavioral pattern, the actor’s muzzle may point downwards or forward but the head and neck are leaning downwards. | E1 | FD | NO | - | E1 | E1 |
|  | Belly exposure *(Duration)* | To lie on the back showing the stomach holding the tail between the legs. The ears are held back and close to the head and the subject can raise a hind leg for inguinal presentation. | Yes | Yes | Yes | - | Yes | Yes |
|  | Flee  *(Frequency)* | To run away/ jump away (startle response) from stimuli with tail tucked between the legs and body ducked. | Yes | Yes | Yes | - | Yes | Yes |
|  | Crouch *(Duration)* | Lowered posture bending the legs, arching the back, lowered tail | E1 | FD | NO | - | E1 | E1 |
|  | Risk assessment *(Duration)* | Back legs extended; nose reaches towards object | E1 | FD | NO | - | E1 | E1 |
|  | Play *(Duration)* | Jumping around with wagging tail while looking at person, play-bow (only with tail wagging, relaxed clumsy body posture) | E1 | FD | NO | - | E1 | E1 |
|  | Friendly *(Duration)* | Moves towards the receiver or stays in their vicinity in relaxed posture with ears pointed forward and the head pointed towards the receiver with the tail wagging at or above body line. | E1 | FD | NO | - | E1 | E1 |
|  | Bow *(Frequency)* | Crouches down touching or almost touching forelimbs to the ground with rear end high in the air, orientation is directed towards partner. Not stretching 🡪 weight is equally on all paws, not leaning towards the hind paws | E1 | FD | NO | - | E1 | E1 |
| Not visible  *(Duration)* | Not visible | Out of camera view | Yes | Yes | Yes | Yes | Yes | Yes |
| Disturbance  *(Duration)* | Disturbance | When behaviour is clearly directed at dogs/ other persons passing by/ distracting activities by E1/2 (e.g. feeding other dogs)/ other dog getting close enough to disturb the test | E1 / E2 / Person / Dogs | | | | | |
| Termination *(Occurrence)* | Termination | Termination of subtest or full test | Yes | Yes | Yes | Yes | Yes | Yes |

| **Subtest-specific variables** | | | | | | | | |
| --- | --- | --- | --- | --- | --- | --- | --- | --- |
| **Subtest** | **Behaviour** | **Definition** | **Applicable subtest** | | | | | |
|  |  |  | **HA** | **FD** | **NO** | **P** | **B** | **T** |
| Sociability | First approach  *(Binary)* | Dog approaches E1 within 1 body length distance for the first time | E1 | - | - | - | - | - |
|  | Interacting with toy *(Duration)* | Actively touching the toy with the mouth or paw | Yes |  |  |  |  |  |
| Fake dog | Latency to approach fake dog | Time until the dog actively gets into less than 2 body lengths from the fake dog from first seeing it | - | FD | - | - | - | - |
|  | Genital sniffing *(Frequency)* | To sniff the genital area of the fake dog | - | FD | - | - | - | - |
| Novel object | Latency to approach the novel object | Time until the dog actively gets into less than 2 body lengths from the object from first seeing it | - | - | NO | - | - | - |
| Pointing  *(Frequency)* | Observation | Whether the dog watches the pointing gesture | - | - | - | Yes/No | - | - |
|  | Success | Whether the dog chooses the bowl that was pointed at |  |  |  | Yes/No |  |  |
|  | No choice | A trial where the dog did not make a choice | - | - | - | Yes | - | - |
| Begging  *(Frequency)* | 2-way gaze alternation E1-Bowl | Looking at E1 and then the bowl without looking at anything else in between | - | - | - | - | Yes | Yes |
|  | 2-way gaze alternation Bowl-E1 | looking at bowl and then E1 without looking at anything else in between | - | - | - | - | Yes | Yes |
|  | 3-way gaze alternation E1-Bowl-E1 | looking at E1 then bowl and then E1 without looking at anything else in between | - | - | - | - | Yes | Yes |
|  | 3-way gaze alternation Bowl-E1-Bowl | looking at bowl then E1 and then bowl without looking at anything else in between | - | - | - | - | Yes | Yes |
|  | Attempt to reach the bowl | Jumping towards the bowl, two legs off the ground, trying to climb the car/tree | - | - | - | - | Yes | - |
| Tractability | 2-way gaze alternation E1-Stool *(Frequency)* | Looking at E1 and then the stool without looking at anything else in between | - | - | - | - | - | Yes |
|  | 2-way gaze alternation Stool-E1 *(Frequency)* | looking at stool and then E1 without looking at anything else in between | - | - | - | - | - | Yes |
|  | 3-way gaze alternation E1-Stool-E1 *(Frequency)* | looking at E1 then stool and then E1 stool without looking at anything else in between | - | - | - | - | - | Yes |
|  | 3-way gaze alternation Stool-E1-Stool  *(Frequency)* | looking at stool then E1 and stool without looking at anything else in between | - | - | - | - | - | Yes |
|  | Latency (T) to get to the food | Time from beggining of No-phase to Success eating the food | - | - | - | - | - | Yes |
|  | Phase in which food is eaten  *(Occurrence)* | Dog eats the food | - | - | - | - | - | Yes |
