# Supplementary material for: Street-wise dog testing: Feasibility and reliability of a behavioural test battery for free-ranging dogs in their natural habitat
Source: PLoS One. 2024 Mar 14;19(3):e0296509. doi: 10.1371/journal.pone.0296509 (PMC10939227; doi:10.1371/journal.pone.0296509)
Supplement: S2 Table — (DOCX) [file pone.0296509.s005.docx]

**S4 Table. Detailed inter- and intra-rater reliability results.** Displayed are the ICC for the inter- and intra-rater analysis for each behaviour variable and modifier summed across subtests. The lower and upper 95% confidence interval (CI), F-value (F), degreed of freedom (df), and p-value are presented. The ICC analysis was based on a two-way random effects ANOVA with an absolute agreement estimate. The ICC-target for acceptable coding reliability was 0.7. Moderate reliability below the target (i.e., 0.5<ICC<0.7) is emphasized with a light grey background, poor reliability (ICC<0.5) with a dark grey background. Variables that did not occur in the coded videos are indicated with NA.

| **GENERAL** | | **Inter-rater** | | | | | | |  | **Intra-rater** | | | | | | |
| --- | --- | --- | --- | --- | --- | --- | --- | --- | --- | --- | --- | --- | --- | --- | --- | --- |
| **Category** | **Behavioural variable** | **ICC Inter** | **lower ICC** | **upper ICC** | **F-value** | **df1** | **df2** | **p-value** |  | **ICC Intra** | **lower ICC** | **upper ICC** | **F-value** | **df1** | **df2** | **p-value** |
| **Proximity** | Close | 0.90 | 0.61 | 0.98 | 18.83 | 7 | 7.99 | <0.01 |  | 0.97 | 0.90 | 0.99 | 57.07 | 12 | 12.88 | <0.01 |
|  | Medium | 0.53 | -0.11 | 0.88 | 3.56 | 7 | 7.90 | 0.05 |  | 0.52 | -0.08 | 0.84 | 3.00 | 11 | 11.00 | 0.04 |
| **Tail** | Wagging | 0.91 | 0.66 | 0.98 | 24.94 | 7 | 7.51 | <0.01 |  | 0.91 | 0.68 | 0.97 | 28.76 | 12 | 7.65 | <0.01 |
|  | Between legs | 0.64 | 0.04 | 0.90 | 4.47 | 8 | 8.85 | 0.02 |  | 0.99 | 0.98 | 1.00 | 304.18 | 12 | 11.34 | <0.01 |
| **Gazing** | | 0.90 | 0.60 | 0.98 | 17.81 | 7 | 7.89 | <0.01 |  | 0.98 | 0.90 | 0.99 | 126.08 | 12 | 6.91 | <0.01 |
| **Vocalization** | Barking | NA | NA | NA | NA | 7 | NA | NA |  | NA | NA | NA | NA | 12 | NA | NA |
|  | Growling | 0.80 | 0.37 | 0.95 | 9.00 | 8 | 8.99 | 0.00 |  | NA | NA | NA | NA | 12 | NA | NA |
|  | Whining | NA | NA | NA | NA | 5 | NA | NA |  | NA | NA | NA | NA | 12 | NA | NA |
| **Displacement** | Nose-licking | 0.88 | 0.35 | 0.99 | 15.18 | 4 | 4.98 | 0.01 |  | 0.96 | 0.64 | 1.00 | 36.50 | 4 | 4.06 | <0.01 |
|  | Body shaking | 0.80 | 0.37 | 0.95 | 9.00 | 8 | 8.99 | <0.01 |  | NA | NA | NA | NA | 12 | NA | NA |
|  | Stretching | 0.88 | 0.21 | 0.99 | 15.41 | 3 | 3.99 | 0.01 |  | 1.00 | 1.00 | 1.00 | 4772.57 | 4 | 4.73 | <0.01 |
|  | Yawning | 0.94 | 0.69 | 0.99 | 33.17 | 5 | 6.00 | <0.01 |  | 0.97 | 0.78 | 1.00 | 2.86 | 4 | 4.00 | 0.17 |
|  | Sniffing the ground | 0.82 | 0.36 | 0.96 | 11.24 | 7 | 7.34 | <0.01 |  | 0.79 | 0.40 | 0.94 | 8.06 | 10 | 10.69 | <0.01 |
| **Physical contact** | Biting | NA | NA | NA | NA | 7 | NA | NA |  | NA | NA | NA | NA | 12 | NA | NA |
|  | Body contact | 1.00 | 0.99 | 1.00 | 920.38 | 7 | 7.89 | <0.01 |  | 0.74 | 0.37 | 0.91 | 6.81 | 12 | 12.99 | <0.01 |
|  | Jumping | 0.95 | 0.72 | 0.99 | 35.47 | 5 | 5.81 | <0.01 |  | 0.98 | 0.93 | 0.99 | 85.24 | 12 | 12.01 | <0.01 |
|  | Licking | NA | NA | NA | NA | 5 | NA | NA |  | 0.84 | 0.57 | 0.95 | 11.19 | 12 | 12.49 | <0.01 |
|  | Mouthing | 0.64 | 0.00 | 0.91 | 4.59 | 7 | 7.95 | 0.02 |  | 0.95 | 0.84 | 0.98 | 37.08 | 11 | 12.00 | <0.01 |
|  | Pawing | NA | NA | NA | NA | 7 | NA | NA |  | 0.99 | 0.94 | 1.00 | 142.10 | 7 | 7.00 | <0.01 |
|  | Sniffing | 0.97 | 0.87 | 0.99 | 61.22 | 7 | 7.03 | <0.01 |  | 0.98 | 0.92 | 0.99 | 75.61 | 12 | 12.10 | <0.01 |
| **Reactions** | Stand tall | -0.03 | -0.68 | 0.62 | 0.95 | 8 | 7.92 | 0.53 |  | 0.91 | 0.75 | 0.97 | 21.45 | 12 | 12.79 | <0.01 |
|  | Bare teeth | NA | NA | NA | NA | 7 | NA | NA |  | NA | NA | NA | NA | 12 | NA | NA |
|  | Lunge | NA | NA | NA | NA | 7 | NA | NA |  | NA | NA | NA | NA | 12 | NA | NA |
|  | Head dip | -0.08 | -0.69 | 0.59 | 0.86 | 8 | 7.72 | 0.58 |  | 0.85 | 0.59 | 0.95 | 12.12 | 12 | 12.63 | <0.01 |
|  | Belly exposure | NA | NA | NA | NA | 7 | NA | NA |  | NA | NA | NA | NA | 12 | NA | NA |
|  | Flee | 0.77 | 0.27 | 0.94 | 9.99 | 8 | 6.67 | <0.01 |  | 0.94 | 0.82 | 0.98 | 33.71 | 12 | 12.66 | <0.01 |
|  | Crouch | 0.90 | 0.66 | 0.98 | 20.00 | 8 | 9.00 | <0.01 |  | 0.79 | 0.45 | 0.93 | 8.28 | 12 | 12.79 | <0.01 |
|  | Risk assessment | 0.99 | 0.97 | 1.00 | 272.21 | 8 | 8.85 | <0.01 |  | 0.86 | 0.63 | 0.96 | 13.75 | 12 | 12.99 | <0.01 |
|  | Play | 0.98 | 0.92 | 1.00 | 104.07 | 8 | 9.00 | <0.01 |  | 0.98 | 0.94 | 0.99 | 93.62 | 12 | 12.98 | <0.01 |
|  | Friendly | 0.44 | -0.12 | 0.83 | 3.54 | 8 | 6.25 | 0.07 |  | 0.97 | 0.87 | 0.99 | 73.69 | 12 | 8.29 | <0.01 |
|  | Bow | 0.81 | 0.40 | 0.95 | 9.60 | 8 | 8.99 | <0.01 |  | 0.76 | 0.40 | 0.92 | 7.64 | 12 | 12.87 | <0.01 |
| **Marking** |  | NA | NA | NA | NA | 3 | NA | NA |  | 0.99 | 0.96 | 1.00 | 326.30 | 4 | 5.00 | <0.01 |
| **Defecating** |  | NA | NA | NA | NA | 4 | NA | NA |  | NA | NA | NA | NA | 4 | NA | NA |
| **Human approach** | Phase of first approach | 1.00 | NA | NA | Inf | 7 | NA | NA |  | 1.00 | NA | NA | Inf | 12 | NA | NA |
|  | Interacting with toy | 0.99 | 0.97 | 1.00 | 305.48 | 7 | 8.00 | <0.01 |  | 0.99 | 0.97 | 1.00 | 277.95 | 10 | 10.85 | <0.01 |
| **Fake Dog** | Latency | 1.00 | 0.99 | 1.00 | 1529.48 | 7 | 7.40 | <0.01 |  | 0.97 | 0.87 | 0.99 | 61.07 | 7 | 7.04 | <0.01 |
|  | Genital sniffing | 0.92 | 0.72 | 0.98 | 25.08 | 8 | 9.00 | <0.01 |  | 1.00 | NA | NA | Inf | 8 | NA | NA |
| **Novel object** | Phase of first approach | 1.00 | NA | NA | Inf | 7 | NA | NA |  | 1.00 | NA | NA | Inf | 8 | NA | NA |
|  | Latency | 0.85 | 0.33 | 0.98 | 13.95 | 5 | 5.57 | <0.01 |  | 1.00 | 0.99 | 1.00 | 1206.76 | 6 | 6.56 | <0.01 |
| **Pointing** | Observation of gesture: yes | 1.00 | NA | NA | Inf | 7 | NA | NA |  | 0.99 | 0.97 | 1.00 | 223.23 | 12 | 12.00 | <0.01 |
|  | Observation of gesture: no | 1.00 | NA | NA | Inf | 7 | NA | NA |  | 0.98 | 0.94 | 0.99 | 106.69 | 12 | 12.00 | <0.01 |
|  | Successful choice | 1.00 | NA | NA | Inf | 7 | NA | NA |  | 1.00 | NA | NA | Inf | 12 | NA | NA |
|  | Unsuccessful choice | 1.00 | NA | NA | Inf | 7 | NA | NA |  | 1.00 | 0.99 | 1.00 | 423.67 | 12 | 13.00 | <0.01 |
|  | No choice | 1.00 | NA | NA | Inf | 7 | NA | NA |  | 1.00 | 1.00 | 1.00 | < -200 | 12 | 12.00 | 1.00 |
| **Begging** | Attempts to get bowl | 0.99 | 0.95 | 1.00 | 192.60 | 6 | 7.00 | <0.01 |  | 0.99 | 0.98 | 1.00 | 361.50 | 7 | 8.00 | <0.01 |
| **Begging/** | 2-way gaze alternations | 0.82 | 0.21 | 0.97 | 9.76 | 5 | 5.50 | 0.01 |  | 0.79 | 0.22 | 0.95 | 7.53 | 7 | 7.03 | 0.01 |
| **Tractability** | 3-way gaze alternations | 0.86 | 0.47 | 0.98 | 36.16 | 5 | 5.60 | 0.01 |  | 0.87 | 0.50 | 0.97 | 14.91 | 7 | 7.65 | <0.01 |
| **Tractability** | Latency | 0.90 | 0.55 | 0.98 | 20.41 | 6 | 6.57 | <0.01 |  | 1.00 | 0.99 | 1.00 | 1206.76 | 6 | 6.56 | <0.01 |
|  | Phase of eating | 1.00 | NA | NA | Inf | 7 | NA | NA |  | 1.00 | NA | NA | Inf | 12 | NA | NA |
