# Supplementary material for: Street-wise dog testing: Feasibility and reliability of a behavioural test battery for free-ranging dogs in their natural habitat
Source: PLoS One. 2024 Mar 14;19(3):e0296509. doi: 10.1371/journal.pone.0296509 (PMC10939227; doi:10.1371/journal.pone.0296509)
Supplement: S3 Table — (DOCX) [file pone.0296509.s006.docx]

**S5 Table. Detailed test-retest reliability outcome (n = 26).** Displayed are the ICCs for the test-retest reliability for each subtest, variable, and modifier for all tests and for only the ones where no disturbance was coded. The lower and upper 95% confidence interval (CI), F-value (F), degreed of freedom (df), and p-value are presented. Variables that were not significant are indicated with a grey background. The ICC analysis was based on a two-way mixed effects ANOVA with an absolute agreement estimate. Variables that were excluded in the first validation step (i.e., that did not occur in more than 10% of all testing occasions or had a bad inter- and intra-rater reliability) were not analysed for their test-retest reliability*.*

|  |  |  | **All street tests** | | | | | | | | | | | |  | **Only street tests that had no coded disturbances** | | | | | | | | | | | | | | | | | | | | |
| --- | --- | --- | --- | --- | --- | --- | --- | --- | --- | --- | --- | --- | --- | --- | --- | --- | --- | --- | --- | --- | --- | --- | --- | --- | --- | --- | --- | --- | --- | --- | --- | --- | --- | --- | --- | --- |
|  |  |  |  | | | | | | | | | | | |  |  | | | | | | | | | | | | | | | | | | | | |
| **Subtest** | **Variable** | **Modifier** | **ICC** | **Lower CI** | | | **Upper CI** | **F** | **df1** | | | **df2** | | **p** |  | **ICC** | | **Low-er CI** | | **Upper CI** | | **F** | | | **df1** | | | | **df2** | | | **p** | | | | |
| **Human approach** | **Close proximity** | E1 | 0.71 | | 0.45 | 0.86 | | 6.21 | | 24 | 23.69 | | <0.01 | |  | 0.77 | | 0.45 | | 0.91 | | 9.77 | | 18 | | | | 11.43 | | | | | | <0.01 | | |
|  | **Medium proximity** | E1 | 0.22 | | -0.17 | 0.56 | | 1.57 | | 24 | 24.78 | | 0.14 | |  | 0.34 | | -0.08 | | 0.67 | | 2.14 | | 18 | | | | 18.91 | | | | | | 0.05 | | |
|  | **Tail wagging** | E1 | 0.61 | | 0.2 | 0.82 | | 5.38 | | 24 | 11.01 | | <0.01 | |  | 0.62 | | 0.16 | | 0.84 | | 5.67 | | 18 | | | | 9.3 | | | | | | 0.01 | | |
|  | **Tail between legs** |  | 0.53 | | 0.18 | 0.77 | | 3.25 | | 23 | 23.7 | | 0 | |  | 0.52 | | 3.08 | | 15 | | 15.68 | | 0.02 | | | | 0.05 | | | | | | 0.8 | | |
|  | **Gazing** | E1 | 0.35 | | -0.01 | 0.64 | | 2.4 | | 24 | 18.42 | | 0.03 | |  | 0.34 | | -0.06 | | 0.66 | | 2.27 | | 18 | | | | 16.67 | | | | | | 0.05 | | |
|  | **Barking** | E1 | 0.85 | | 0.7 | 0.93 | | 12.66 | | 24 | 24.96 | | <0.01 | |  | 0.85 | | 0.66 | | 0.94 | | 12.69 | | 18 | | | | 18.92 | | | | | | <0.01 | | |
|  | **Body contact** | E1 | 0.55 | | 0.22 | 0.77 | | 3.57 | | 24 | 24.64 | | <0.01 | |  | 0.55 | | 0.17 | | 0.8 | | 3.72 | | 18 | | | | 18.32 | | | | | | <0.01 | | |
|  | **Jumping** | E1 | 0.5 | | 0.14 | 0.75 | | 3 | | 24 | 24.57 | | <0.01 | |  | 0.46 | | 0.04 | | 0.75 | | 2.76 | | 18 | | | | 18.95 | | | | | | 0.02 | | |
|  | **sniffing E1** | E1 | 0.1 | | -0.25 | 0.45 | | 1.25 | | 24 | 24.86 | | 0.29 | |  | 0.05 | | -0.38 | | 0.48 | | 1.11 | | 18 | | | | 18.29 | | | | | | 0.41 | | |
|  | **Nose /mouth licking** |  | 0.00 | | -0.4 | 0.39 | | 1 | | 24 | 24 | | 0.5 | |  | -0.01 | | -0.48 | | 0.45 | | 0.98 | | 18 | | | | 17.99 | | | | | | 0.52 | | |
|  | **Yawning** |  | -0.03 | | -0.44 | 0.37 | | 0.94 | | 24 | 23.99 | | 0.56 | |  | -0.08 | | -0.53 | | 0.39 | | 0.86 | | 18 | | | | 17.89 | | | | | | 0.62 | | |
|  | **Flee** | E1 | -0.08 | | -0.46 | 0.32 | | 0.85 | | 23 | 22.6 | | 0.65 | |  | -0.16 | | -0.64 | | 0.37 | | 0.74 | | 15 | | | | 14.87 | | | | | | 0.72 | | |
|  | **Friendly** | E1 | 0.45 | | 0.04 | 0.73 | | 3.43 | | 23 | 11.98 | | 0.02 | |  | 0.56 | | 0.11 | | 0.82 | | 4.33 | | 15 | | | | 11.24 | | | | | | 0.01 | | |
|  | **Head dip** | E1 | -0.08 | | -0.49 | 0.34 | | 0.86 | | 23 | 22.97 | | 0.64 | |  | -0.09 | | -0.61 | | 0.43 | | 0.84 | | 15 | | | | 15 | | | | | | 0.63 | | |
|  | **Play** | E1 | -0.03 | | -0.42 | 0.37 | | 0.94 | | 23 | 22.89 | | 0.56 | |  | -0.06 | | -0.58 | | 0.45 | | 0.89 | | 15 | | | | 14.98 | | | | | | 0.59 | | |
|  | **Sniffing the ground** |  | -0.04 | | -0.45 | 0.36 | | 0.92 | | 24 | 23.99 | | 0.58 | |  | -0.04 | | -0.51 | | 0.43 | | 0.93 | | 18 | | | | 17.99 | | | | | | 0.56 | | |
|  | **Disturbance** | ALL | -0.03 | | -0.43 | 0.36 | | 0.93 | | 24 | 23.94 | | 0.56 | |  |  | |  | |  | |  | |  | | | |  | | | | | |  | | |
|  | **Not visible** |  | 0.31 | | -0.11 | 0.62 | | 1.85 | | 24 | 24.02 | | 0.07 | |  | 0.39 | | -0.08 | | 0.72 | | 2.24 | | 18 | | | | 18.04 | | | | | | 0.05 | | |
|  | **First approach E1** |  | 0.49 | | 0.14 | 0.74 | | 2.92 | | 25 | 25.65 | | <0.01 | |  | 0.51 | | 0.1 | | 0.78 | | 3.16 | | 18 | | | | 19 | | | | | | 0.01 | | |
|  |  |  |  | |  |  | |  | |  |  | |  | |  |  | |  | |  | |  | |  | | | |  | | | | | |  | | |
|  |  |  |  | |  |  | |  | |  |  | |  | |  |  | |  | |  | |  | |  | | | |  | | | | | |  | | |
|  | **Close proximity** | Fake dog | 0 | | -0.48 | 0.48 | | 1 | | 15 | 15 | | 0.5 | |  | 0.74 | | 0.02 | | 0.96 | | 10.2 | | 5 | | | | 3.9 | | | | | | 0.02 | | |
|  | **Close proximity** | E1+E2 | -0.08 | | -0.55 | 0.42 | | 0.84 | | 15 | 14.68 | | 0.63 | |  | -0.17 | | -1.06 | | 0.71 | | 0.74 | | 5 | | | | 4.83 | | | | | | 0.62 | | |
|  | **Medium proximity** | Fake dog | 0.12 | | -0.37 | 0.57 | | 1.29 | | 15 | 15.42 | | 0.31 | |  | 0.23 | | -0.73 | | 0.85 | | 1.55 | | 5 | | | | 5.29 | | | | | | 0.32 | | |
|  | **Medium proximity** | E1+E2 | 0.03 | | -0.49 | 0.51 | | 1.05 | | 15 | 15.03 | | 0.46 | |  | -0.07 | | -0.86 | | 0.74 | | 0.88 | | 5 | | | | 4.82 | | | | | | 0.56 | | |
|  | **Tail wagging** | Fake dog | 0.14 | | -0.26 | 0.54 | | 1.39 | | 15 | 16 | | 0.26 | |  | -0.12 | | -0.85 | | 0.71 | | 0.79 | | 5 | | | | 4.61 | | | | | | 0.6 | | |
|  | **Tail between legs** |  | 0.71 | | 0.35 | 0.89 | | 5.82 | | 15 | 15.8 | | 0 | |  | 0.72 | | 0.09 | | 0.94 | | 6.15 | | 6 | | | | 6.97 | | | | | | 0.02 | | |
|  | **Gazing** | Fake dog | 0.16 | | -0.22 | 0.56 | | 1.5 | | 15 | 15.81 | | 0.21 | |  | 0.11 | | -0.12 | | 0.64 | | 1.9 | | 5 | | | | 4.73 | | | | | | 0.26 | | |
| **Fake Dog** | **Barking** | Fake dog | 0.12 | | -0.31 | 0.55 | | 1.31 | | 15 | 15.87 | | 0.3 | |  | 0.12 | | -0.5 | | 0.78 | | 1.32 | | 5 | | | | 5.63 | | | | | | 0.37 | | |
|  | **Nose /mouth licking** |  | 0.16 | | -0.37 | 0.6 | | 1.37 | | 15 | 15.21 | | 0.27 | |  | 0.56 | | -0.32 | | 0.92 | | 3.4 | | 5 | | | | 5.7 | | | | | | 0.09 | | |
|  | **Yawning** |  | -0.06 | | -0.49 | 0.42 | | 0.88 | | 15 | 14.49 | | 0.59 | |  | 0.00 | | -0.75 | | 0.75 | | 1 | | 5 | | | | 5 | | | | | | 0.5 | | |
|  | **Friendly** | Fake dog | -0.07 | | -0.58 | 0.44 | | 0.88 | | 15 | 14.95 | | 0.6 | |  | 0.15 | | -0.46 | | 0.75 | | 1.41 | | 6 | | | | 6.76 | | | | | | 0.33 | | |
|  | **Head dip** | Fake dog | -0.21 | | -0.68 | 0.33 | | 0.67 | | 15 | 14.77 | | 0.78 | |  | 0.00 | | -0.54 | | 0.67 | | 1 | | 6 | | | | 6 | | | | | | 0.5 | | |
|  | **Risk assessment** | Fake dog | -0.04 | | -0.55 | 0.46 | | 0.92 | | 15 | 14.94 | | 0.56 | |  | -0.04 | | -0.78 | | 0.7 | | 0.93 | | 6 | | | | 5.9 | | | | | | 0.54 | | |
|  | **Stand tall** | Fake dog | -0.12 | | -0.62 | 0.41 | | 0.8 | | 15 | 15 | | 0.66 | |  | -0.29 | | -1.09 | | 0.6 | | 0.61 | | 6 | | | | 5.98 | | | | | | 0.72 | | |
|  | **Sniffing object** | Fake dog | 0.03 | | -0.4 | 0.49 | | 1.07 | | 15 | 15.26 | | 0.45 | |  | 0.19 | | -0.27 | | 0.78 | | 1.8 | | 5 | | | | 5.87 | | | | | | 0.25 | | |
|  | **Disturbance** | All | 0.26 | | -0.29 | 0.67 | | 1.67 | | 15 | 15 | | 0.17 | |  |  | |  | |  | |  | |  | | | |  | | | | | |  | | |
|  | **Not visible** |  | -0.01 | | -0.43 | 0.46 | | 0.99 | | 15 | 14.94 | | 0.51 | |  | -0.02 | | -0.55 | | 0.7 | | 0.95 | | 5 | | | | 4.83 | | | | | | 0.52 | | |
|  | **Latency (FD)** |  | -0.15 | | -0.62 | 0.37 | | 0.75 | | 15 | 14.67 | | 0.71 | |  | 0.2 | | -0.92 | | 0.85 | | 1.43 | | 5 | | | | 5 | | | | | | 0.35 | | |
|  | **Genital sniffing** | Fake dog | 0.15 | | -0.29 | 0.57 | | 1.4 | | 15 | 15.93 | | 0.25 | |  | -0.15 | | -0.77 | | 0.62 | | 0.73 | | 6 | | | | 5.25 | | | | | | 0.65 | | |
|  |  |  |  | |  |  | |  | |  |  | |  | |  |  |  |  |  | |  |  |  | | |  |  | | |  |  | |  | |  |  |
|  | **Close proximity** | Novel object | -0.09 | | -0.7 | 0.51 | | 0.84 | | 11 | 11 | | 0.61 | |  | 0.06 | | -0.84 | | 0.84 | | 1.13 | | 4 | | | | 4.15 | | | | | | 0.45 | | |
|  | **Close proximity** | E1+E2 | 0.74 | | 0.3 | 0.92 | | 8.06 | | 11 | 9.02 | | <0.01 | |  | 0.00 | | -0.81 | | 0.81 | | 1 | | 4 | | | | 4 | | | | | | 0.5 | | |
|  | **Medium proximity** | Novel object | 0.01 | | -0.43 | 0.52 | | 1.01 | | 11 | 11.07 | | 0.49 | |  | 0.01 | | -0.59 | | 0.78 | | 1.02 | | 4 | | | | 4.05 | | | | | | 0.49 | | |
|  | **Medium proximity** | E1+E2 | -0.08 | | -0.56 | 0.48 | | 0.84 | | 11 | 10.34 | | 0.61 | |  | 0.00 | | -0.81 | | 0.81 | | 1 | | 4 | | | | 4 | | | | | | 0.5 | | |
| **Novel Object** | **Tail wagging** | Novel object | 0.04 | | -0.4 | 0.54 | | 1.1 | | 11 | 11.41 | | 0.44 | |  | NA | | NA | | NA | | NA | | 4 | | | | NA | | | | | | NA | | |
|  | **Gazing** | Novel object | -0.35 | | -0.85 | 0.31 | | 0.52 | | 11 | 10.84 | | 0.85 | |  | -0.06 | | -0.53 | | 0.73 | | 0.85 | | 4 | | | | 3.44 | | | | | | 0.57 | | |
|  | **Gazing** | E1+E2 | -0.26 | | -0.79 | 0.37 | | 0.61 | | 11 | 10.63 | | 0.79 | |  | -0.08 | | -0.86 | | 0.76 | | 2.23 | | 4 | | | | 4.18 | | | | | | 0.46 | | |
|  | **Nose /mouth licking** |  | 0.37 | | -0.25 | 0.77 | | 2.11 | | 11 | 11.47 | | 0.11 | |  | 0.61 | | -0.15 | | 0.95 | | 5.84 | | 4 | | | | 4 | | | | | | 0.05 | | |
|  | **Yawning** |  | 0.48 | | -0.11 | 0.82 | | 2.74 | | 11 | 11.41 | | 0.05 | |  | 0.45 | | -0.55 | | 0.92 | | 2.55 | | 4 | | | | 4.7 | | | | | | 0.17 | | |
|  | **Sniffing object** | Novel object | -0.04 | | -0.58 | 0.53 | | 0.93 | | 11 | 10.85 | | 0.55 | |  | 0.00 | | -0.81 | | 0.81 | | 1 | | 4 | | | | 4 | | | | | | 0.5 | | |
|  | **Flee** | Novel object | 0.32 | | -0.3 | 0.73 | | 1.87 | | 12 | 12 | | 0.15 | |  | NA | | NA | | NA | | NA | | 2 | | | | NA | | | | | | NA | | |
|  | **Head dip** | Novel Object | 0.00 | | -0.42 | 0.49 | | 1 | | 12 | 12 | | 0.5 | |  | NA | | NA | | NA | | NA | | 2 | | | | NA | | | | | | NA | | |
|  | **Not visible** |  | -0.05 | | -0.63 | 0.53 | | 0.91 | | 11 | 10.91 | | 0.56 | |  | -0.09 | | -0.99 | | 0.8 | | 0.84 | | 4 | | | | 3.8 | | | | | | 0.57 | | |
|  | **Disturbance** | All | -0.27 | | -0.74 | 0.34 | | 0.57 | | 11 | 9.34 | | 0.81 | |  |  | |  | |  | |  | |  | | | |  | | | | | |  | | |
|  | **Latency (NO)** |  | 0.05 | | -0.58 | 0.6 | | 1.09 | | 11 | 11.03 | | 0.45 | |  | -0.9 | | -1.56 | | 0.45 | | 0.24 | | 4 | | | | 3.99 | | | | | | 0.9 | | |
|  | **Approach** |  | 0.2 | | -0.22 | 0.58 | | 1.55 | | 18 | 18.96 | | 0.18 | |  | 0.00 | | -0.78 | | 0.89 | | 1 | | 4 | | | | 4 | | | | | | 0.5 | | |
|  |  |  |  | |  |  | |  | |  |  | |  | |  |  |  |  |  | |  |  |  | | |  |  | | |  |  | |  | |  |  |
| **Pointing** | **Tail wagging** | E1 | 0.5 | | 0.04 | 0.79 | | 2.93 | | 16 | 16.38 | | 0.02 | |  | 0.2 | | -0.62 | | 0.77 | | 1.46 | | 7 | | | | 7.18 | | | | | | 0.31 | | |
|  | **Disturbance** | All | -0.07 | | -0.57 | 0.43 | | 0.88 | | 16 | 15.99 | | 0.6 | |  |  | |  | |  | |  | |  | | | |  | | | | | |  | | |
|  | **Not visible** |  | -0.01 | | -0.45 | 0.45 | | 0.98 | | 16 | 15.95 | | 0.51 | |  | 0 | | -0.67 | | 0.67 | | 1 | | 7 | | | | 7 | | | | | | 0.5 | | |
|  | **No choice** |  | 0.52 | | 0.19 | 0.75 | | 3.39 | | 25 | 24.1 | | <0.01 | |  | 0.17 | | -0.27 | | 0.7 | | 1.63 | | 7 | | | | 7.9 | | | | | | 0.26 | | |
|  | **Observation of gesture** | no | 0.33 | | -0.02 | 0.62 | | 2.2 | | 25 | 23.16 | | 0.03 | |  | -0.2 | | -0.33 | | 0.37 | | 0.35 | | 7 | | | | 0.57 | | | | | | 0.87 | | |
|  | **Observation of gesture** | yes | 0.51 | | 0.09 | 0.75 | | 3.73 | | 25 | 12.74 | | 0.01 | |  | 0.12 | | -0.23 | | 0.64 | | 1.49 | | 7 | | | | 7.84 | | | | | | 0.29 | | |
|  | **Success** | no | 0.55 | | 0.2 | 0.77 | | 3.32 | | 25 | 25.06 | | <0.01 | |  | 0.06 | | -0.8 | | 0.72 | | 1.11 | | 7 | | | | 7 | | | | | | 0.45 | | |
|  | **Success** | yes | 0.64 | | 0.34 | 0.82 | | 4.49 | | 25 | 25.64 | | <0.01 | |  | 0.38 | | -0.16 | | 0.81 | | 3.03 | | 7 | | | | 5.89 | | | | | | 0.1 | | |
|  |  |  |  | |  |  | |  | |  |  | |  | |  |  |  |  |  | |  |  |  | | |  |  | | |  |  | |  | |  |  |
|  | **Close proximity** | E1 | 0.8 | | 0.48 | 0.93 | | 8.56 | | 13 | 13.76 | | <0.01 | |  | 0.76 | | 0.33 | | 0.93 | | 7 | | 10 | | | | 10.63 | | | | | | <0.01 | | |
|  | **Medium Proximity** | E1 | -0.16 | | -0.66 | 0.4 | | 0.73 | | 13 | 12.56 | | 0.71 | |  | -0.25 | | -0.77 | | 0.4 | | 0.61 | | 10 | | | | 9.04 | | | | | | 0.78 | | |
|  | **Tail wagging** | E1 | 0.36 | | -0.15 | 0.73 | | 2.18 | | 13 | 13.99 | | 0.08 | |  | 0.3 | | -0.33 | | 0.75 | | 1.85 | | 10 | | | | 10.59 | | | | | | 0.17 | | |
|  | **Gazing** | E1 | 0 | | -0.44 | 0.48 | | 0.99 | | 13 | 12.97 | | 0.5 | |  | 0.24 | | -0.37 | | 0.71 | | 1.62 | | 10 | | | | 10.65 | | | | | | 0.22 | | |
|  | **Gazing** | Bowl | 0.34 | | -0.24 | 0.73 | | 1.96 | | 13 | 13.26 | | 0.12 | |  | 0.12 | | -0.56 | | 0.67 | | 1.26 | | 10 | | | | 10.06 | | | | | | 0.36 | | |
|  | **Nose /mouth licking** |  | -0.2 | | -0.72 | 0.38 | | 0.68 | | 13 | 12.89 | | 0.75 | |  | -0.28 | | -0.84 | | 0.4 | | 0.6 | | 10 | | | | 9.84 | | | | | | 0.79 | | |
| **Begging** | **Friendly** | E1 | 0.34 | | -0.15 | 0.71 | | 2.08 | | 14 | 15 | | 0.09 | |  | 0.35 | | -0.19 | | 0.75 | | 2.16 | | 11 | | | | 12 | | | | | | 0.1 | | |
|  | **Yawning** |  | -0.09 | | -0.61 | 0.46 | | 0.85 | | 13 | 12.85 | | 0.62 | |  | -0.1 | | -0.73 | | 0.53 | | 0.83 | | 10 | | | | 9.96 | | | | | | 0.61 | | |
|  | **2-way gaze** | Bowl,E1 | -0.04 | | -0.5 | 0.47 | | 0.92 | | 13 | 12.69 | | 0.56 | |  | -0.03 | | -0.64 | | 0.56 | | 0.94 | | 10 | | | | 9.92 | | | | | | 0.54 | | |
|  | **E1-O** |  |  |  |  |  |  |  |  |  |  |  |  |  |  |  |  |  |  |  |  |  |  |  |  |  |  |  |  |  |  |  |  |  |  |  |
|  | **2-way gaze** | Bowl,E1 | 0.09 | | -0.48 | 0.59 | | 1.19 | | 13 | 13.11 | | 0.38 | |  | -0.15 | | -0.68 | | 0.46 | | 0.73 | | 10 | | | | 9.2 | | | | | | 0.69 | | |
|  | **O-E1** |  |  |  |  |  |  |  |  |  |  |  |  |  |  |  |  |  |  |  |  |  |  |  |  |  |  |  |  |  |  |  |  |  |  |  |
|  | **3-way gaze** | Bowl,E1 | -0.13 | | -0.63 | 0.42 | | 0.78 | | 13 | 12.65 | | 0.67 | |  | -0.15 | | -0.73 | | 0.49 | | 0.76 | | 10 | | | | 9.76 | | | | | | 0.66 | | |
|  | **E1-O-E1** |  |  |  |  |  |  |  |  |  |  |  |  |  |  |  |  |  |  |  |  |  |  |  |  |  |  |  |  |  |  |  |  |  |  |  |
|  | **3-way gaze** | Bowl,E1 | 0.34 | | -0.24 | 0.73 | | 1.97 | | 13 | 13.15 | | 0.12 | |  | 0.2 | | -0.44 | | 0.7 | | 1.48 | | 10 | | | | 10.39 | | | | | | 0.27 | | |
|  | **O-E1-O** |  |  |  |  |  |  |  |  |  |  |  |  |  |  |  |  |  |  |  |  |  |  |  |  |  |  |  |  |  |  |  |  |  |  |  |
|  | **Attempt to reach the bowl** | Bowl | 0.75 | | 0.41 | 0.91 | | 7.15 | | 13 | 13.98 | | <0.01 | |  | NA | | NA | | NA | | NA | | 10 | | | | NA | | | | | | NA | | |
|  |  |  |  | |  |  | |  | |  |  | |  | |  |  |  |  |  | |  |  |  | | |  |  | | |  |  | |  | |  |  |
|  | **Close proximity** | E1 | 0.75 | | 0.38 | 0.91 | | 6.68 | | 13 | 13.05 | | <0.01 | |  | 0.72 | | 0.25 | | 0.91 | | 5.63 | | 11 | | | | 11.05 | | | | | | <0.01 | | |
|  | **Close proximity** | Stool | -0.04 | | -0.51 | 0.47 | | 0.92 | | 13 | 12.72 | | 0.56 | |  | -0.02 | | -0.5 | | 0.52 | | 0.96 | | 11 | | | | 10.84 | | | | | | 0.53 | | |
|  | **Medium proximity** | E1 | 0.89 | | 0.7 | 0.96 | | 16.5 | | 13 | 13.3 | | 0 | |  | 0.87 | | 0.62 | | 0.96 | | 14.41 | | 11 | | | | 11.99 | | | | | | 0 | | |
|  | **Medium proximity** | Stool | -0.05 | | -0.57 | 0.48 | | 0.9 | | 13 | 12.85 | | 0.57 | |  | -0.05 | | -0.64 | | 0.53 | | 0.9 | | 11 | | | | 10.91 | | | | | | 0.57 | | |
|  | **Tail wagging** | E1 | -0.14 | | -0.68 | 0.43 | | 0.77 | | 13 | 12.99 | | 0.68 | |  | -0.06 | | -0.63 | | 0.52 | | 0.89 | | 11 | | | | 10.87 | | | | | | 0.58 | | |
|  | **Tail between legs** |  | 0.29 | | -0.28 | 0.71 | | 1.8 | | 12 | 12.71 | | 0.15 | |  | 0.28 | | 1.8 | | 10 | | 10.7 | | 0.18 | | | | -0.33 | | | | | | 0.74 | | |
|  | **Gazing** | E1 | 0.48 | | 0.01 | 0.79 | | 3.19 | | 13 | 12.72 | | 0.02 | |  | 0.44 | | -0.08 | | 0.79 | | 2.76 | | 11 | | | | 11.82 | | | | | | 0.05 | | |
|  | **Gazing** | Stool | 0.18 | | -0.37 | 0.63 | | 1.43 | | 13 | 13.47 | | 0.26 | |  | 0.14 | | -0.45 | | 0.65 | | 1.33 | | 11 | | | | 11.33 | | | | | | 0.32 | | |
|  | **Nose /mouth licking** |  | 0.41 | | -0.13 | 0.76 | | 2.34 | | 13 | 13.62 | | 0.07 | |  | 0.51 | | -0.08 | | 0.83 | | 2.95 | | 11 | | | | 11.32 | | | | | | 0.04 | | |
|  | **Crouch** | E1 | -0.05 | | -0.55 | 0.49 | | 0.89 | | 12 | 11.69 | | 0.58 | |  | -0.02 | | -0.53 | | 0.55 | | 0.96 | | 10 | | | | 9.87 | | | | | | 0.52 | | |
| **Tractability** | **Flee** | E1 | 0.76 | | 0.41 | 0.92 | | 7.67 | | 12 | 12.94 | | <0.01 | |  | 0.58 | | 0.06 | | 0.86 | | 4.24 | | 10 | | | | 10.28 | | | | | | 0.02 | | |
|  | **Friendly** | E1 | 0.13 | | -0.5 | 0.63 | | 1.26 | | 12 | 12 | | 0.35 | |  | 0.14 | | -0.53 | | 0.67 | | 1.3 | | 10 | | | | 10.11 | | | | | | 0.34 | | |
|  | **Head dip** | E1 | -0.07 | | -0.65 | 0.5 | | 0.87 | | 12 | 11.95 | | 0.59 | |  | -0.02 | | -0.58 | | 0.56 | | 0.95 | | 10 | | | | 9.89 | | | | | | 0.53 | | |
|  | **Yawning** |  | -0.21 | | -0.69 | 0.36 | | 0.66 | | 13 | 12.39 | | 0.76 | |  | -0.2 | | -0.69 | | 0.4 | | 0.67 | | 11 | | | | 9.95 | | | | | | 0.74 | | |
|  | **Not visible** |  | -0.06 | | -0.6 | 0.48 | | 0.89 | | 13 | 12.93 | | 0.58 | |  | -0.05 | | -0.63 | | 0.53 | | 0.91 | | 11 | | | | 10.91 | | | | | | 0.56 | | |
|  | **2-way gaze** | Stool,E1 | 0.22 | | -0.29 | 0.65 | | 1.59 | | 13 | 13.86 | | 0.2 | |  | 0.2 | | -0.34 | | 0.67 | | 1.53 | | 11 | | | | 11.82 | | | | | | 0.24 | | |
|  | **E1-O** |  |  |  |  |  |  |  |  |  |  |  |  |  |  |  |  |  |  |  |  |  |  |  |  |  |  |  |  |  |  |  |  |  |  |  |
|  | **2-way gaze** | Stool,E1 | 0.04 | | -0.53 | 0.55 | | 1.07 | | 13 | 13.02 | | 0.45 | |  | -0.06 | | -0.66 | | 0.53 | | 0.9 | | 11 | | | | 10.96 | | | | | | 0.57 | | |
|  | **O-E1** |  |  |  |  |  |  |  |  |  |  |  |  |  |  |  |  |  |  |  |  |  |  |  |  |  |  |  |  |  |  |  |  |  |  |  |
|  | **3-way gaze** | Stool,E1 | 0.47 | | -0.02 | 0.79 | | 2.9 | | 13 | 13.99 | | 0.03 | |  | 0.47 | | -0.05 | | 0.8 | | 2.97 | | 11 | | | | 11.8 | | | | | | 0.04 | | |
|  | **E1-O-E1** |  |  |  |  |  |  |  |  |  |  |  |  |  |  |  |  |  |  |  |  |  |  |  |  |  |  |  |  |  |  |  |  |  |  |  |
|  | **3-way gaze** | Stool,E1 | 0.3 | | -0.27 | 0.71 | | 1.82 | | 13 | 13.28 | | 0.14 | |  | 0.28 | | -0.33 | | 0.73 | | 1.77 | | 11 | | | | 11.41 | | | | | | 0.18 | | |
|  | **O-E1-O** |  |  |  |  |  |  |  |  |  |  |  |  |  |  |  |  |  |  |  |  |  |  |  |  |  |  |  |  |  |  |  |  |  |  |  |
|  | **Latency (T) to eat food** |  | -0.11 | | -0.66 | 0.45 | | 0.81 | | 13 | 12.95 | | 0.65 | |  | -0.08 | | -0.63 | | 0.51 | | 0.86 | | 11 | | | | 10.79 | | | | | | 0.6 | | |
|  | **Success eating the food** | Phase | 0.65 | | 0.35 | 0.82 | | 4.52 | | 25 | 25.09 | | <0.01 | |  | 0.75 | | 0.36 | | 0.92 | | 7.05 | | 11 | | | | 11.96 | | | | | | <0.01 | | |
|  |  |  |  | |  |  | |  | |  |  | |  | |  |  |  |  |  | |  |  |  | | |  |  | | |  |  | |  | |  |  |
|  | **Termination** | Test | 0.71 | | 0.46 | 0.86 | | 6.21 | | 25 | 24.94 | | <0.01 | |  |  | |  | |  | |  | |  | | | |  | | | | | |  | | |
