# Supplementary material for: Street-wise dog testing: Feasibility and reliability of a behavioural test battery for free-ranging dogs in their natural habitat
Source: PLoS One. 2024 Mar 14;19(3):e0296509. doi: 10.1371/journal.pone.0296509 (PMC10939227; doi:10.1371/journal.pone.0296509)
Supplement: Attachment — Submitted filename: PONE-D-23-22870_R2_Nov2023.pdf [file pone.0296509.s009.pdf]

PLOS ONE

Street-wise dog testing: feasibility and reliability of a behavioural test battery for free-ranging dogs in their natural habitat

--Manuscript Draft--

**Manuscript Number:**	PONE-D-23-22870R1
**Article Type:**	Research Article
**Full Title:**	Street-wise dog testing: feasibility and reliability of a behavioural test battery for free-ranging dogs in their natural habitat
**Short Title:**	Feasibility and reliability of a novel test battery with free-ranging dogs in their natural habitat
**Corresponding Author:**	Svenja Capitain, M.Sc.<br>Linköping University: Linkopings universitet<br>Ernstbrunn, AUSTRIA
**Keywords:**	free-ranging dogs; street dogs; test battery; temperament test; Test-retest reliability; fake dog; sociability; novel object; domestic dog; pointing; tractability
**Abstract:**	Behavioural scientists are increasingly recognizing the need to conduct experiments in the wild to achieve a comprehensive understanding of their species' behaviour. For domestic dogs (Canis familiaris), such progress is lagging behind. While the life in human households is often regarded as dogs' natural habitat, this classification disregards most of the global dog population. The value of including free-ranging dogs as study subjects is increasingly being recognized, but no comprehensive behavioural and cognitive test batteries have been conducted so far and their feasibility and reliability are unknown. This study is the starting point to fill this gap by pioneering and validating an elaborate behavioural test battery ~~with~~ street-living dogs. Therein, six common temperament tests (human-/conspecific-directed sociability, neophobia, tractability) and dog-human communication paradigms (pointing, inaccessible object) were adapted to the street conditions. We evaluated the feasibility of the test battery, the coding reliability of the measures, and investigated their temporal consistency in a retest of the same individuals six weeks later (test-retest reliability). The test battery proved feasible with most dogs participating in all subtests, and it showed satisfactory inter- and intra-rater reliability (0.84 and 0.93 respectively), providing evidence that complex behavioural tests can be conducted even in highly variable street conditions. Retesting revealed that some behaviours could be captured reliably across time, especially when the subtest was particularly engaging (e.g., human approach, point following). In contrast, the low retest reliability for subtests relying on sustained novelty and behaviours that were highly susceptible to disturbances (e.g., gazing) reflects the difficulties of street dog testing, including standardisation in disturbance-prone environments, ecology-dependent adaptation of methods, and intrinsic differences between pet and free-ranging dogs. Bearing the suggestions made in the discussion in mind, this test battery can be valuable in investigating cognition, behavioural profiles, and domestication in such an understudied population as free-ranging dogs.
**Order of Authors:**	Svenja Capitain, M.Sc.
	Giulia Cimarelli, PhD
	Urša Blenkuš, M.Sc.
	Friederike Range, Assoz.-Prof. PhD. Priv.-Doz.
	Sarah Marshall-Pescini, PhD, Priv.-Doz.
**Response to Reviewers:**	Additional Editor Comments:<br><br>Dear Authors<br>Your manuscript has been assessed and serious flaws in the study design, DS concept and grammatical errors have been highlighted. Kindly and thoroughly address the issued raised by the reviews and resubmit for another assessment.<br>Thank you<br>We are very grateful for the reviewers' detailed clarifications and suggestions, as well

Powered by Editorial Manager® and ProduXion Manager® from Aries Systems Corporation

as their encouraging words and their concern with the description of the domestication-related focus. We agree with the suggestion that changing the focus of this paper to free-ranging dog testing overall provides more benefit for the scientific community and the reader. Following both reviewers' suggestion, we have rewritten the paper by dropping the domestication syndrome constraints and refocussing on the merits this paper has.

When submitting your revision, we need you to address these additional requirements.
1.Please ensure that your manuscript meets PLOS ONE's style requirements, including those for file naming.
The requirements have been rechecked and implemented. Particularly file naming for figures and supplementary material has been corrected.
2.Please include a complete copy of PLOS' questionnaire on inclusivity in global research in your revised manuscript.
The requested file has been uploaded.
3.We note that Figures 2 and 3 in your submission contain copyrighted images. We require you to either (1) present written permission from the copyright holder to publish these figures specifically under the CC BY 4.0 license, or (2) remove the figures from your submission.
Figure 2 has been remade to only contain MS PowerPoint pictograms which are free to use. Figure 3 has been removed and links to the specific manufacturers have been included in the supplementary information S2 in case visual information is desired.

Reviewer 1
Reviewer #1: GENERAL COMMENTS:
Let me start with saying that I am very happy to see a behaviour study on free-ranging dogs. However, I cannot recommend this study for publication in its current form for several reasons, the main one being that this is not a study of the domestication syndrome and I find its current presentation and narrative to be quite misleading in all honesty.
It is very unclear to me why the authors have chosen to present their manuscript so elaborate around the domestication syndrome when they a) do not confirm the presence of the domestication syndrome in the dog population (there is no test of whether the alleged domestication syndrome behaviours are in fact correlated – a central assumption of the hypothesis), and b) find that their so-called test battery fails to adequately quantify the behaviours they claim to be associated with the domestication syndrome. The eagerness to label their test battery the "Domestication Syndrome test battery" and claim that it is useful going forward in testing domestication syndrome behaviours, in spite of the very results they bring forward, therefore seem very misplaced and quite frankly puzzling. This is especially emphasized by the fact that half of this test battery includes behaviours that cannot in a meaningful way be associated with the domestication syndrome – a point the authors themselves hint at by mentioning that they will also test other domestication hypotheses with this test battery. These other behaviours, as compared to the claimed domestication syndrome behaviours, are even the only behaviours that with some confidence seem to be feasible and reliable to test in a free-ranging population. Why then label it the "Domestication Syndrome test battery"? It is simply misleading.
That being said, and while I understand that the above is a strong critique of the study, I think there is value in the study from the perspective that it is, in my opinion, actually a methods paper and it would benefit immensely from being rewritten as such. The work the authors have done in examining the feasibility and reliability in conducting in-situ behavioural testing of free-ranging dogs does have merit. So, instead of overselling this study by pinning it on a narrative that it simply cannot sustain, my strong recommendation for this manuscript is therefore that it undergoes major revision in order to be restructured to bring forward the valuable contributions it actually has in terms of the insights on general (not domestication syndrome) behavioural testing of free-ranging dogs. Highlighting the challenges of taking behavioural testing outside controlled laboratory settings and emphasize how this is a necessary next step in moving the field forward is valuable enough on its own.

GENERAL ANSWER to Reviewer #1:
Thank you for your valuable perspective and detailed concerns regarding the approach

Powered by Editorial Manager® and ProduXion Manager® from Aries Systems Corporation

we had chosen to present this study in. The paper was indeed supposed to be a methods paper and we regret that it did not come across as such. Taking your suggestions to heart, it has been entirely rewritten, focusing on the necessity of testing free-ranging populations, how the presented test battery fits into that, and what conclusions can be drawn from our results about the possibilities and limitations of street dog testing. This pertains particularly to the introduction and partially to the discussion, where we have additionally addressed issues you raised with the methodology. The methods and results section have stayed largely the same besides clarifying specific issues you have pointed out, and renaming the Sociability test as Human approach test to clarify that all behaviours were tested. We hope that by refocussing this paper on the merits it has in terms of moving the field forward, this version is more to your satisfaction.

DETAILED COMMENTS:
Line 29: The authors need to remove the physiological reference here. Even though hair samples were collected, they were not analyzed and we have no way of knowing if a) this method is reliable in obtaining physiological parameters, and b) if these correlate with the behavioural traits in the domestication syndrome. This reference is there for misleading in this context.
Our answer: With the rewriting of the abstract, this line was taken out.

Line 53: Some clarity is needed here. Selection on tameness is not the mechanism per se. Tameness is likely the behavioural trait that was selected for during initial animal domestication cascading the phenotypic changes as seen in the domestication syndrome. However, the underlying mechanism causing the presumed correlated traits of the domestication syndrome remains unknown, but has been proposed to be neural crest deficit (Wilkins et al. 2016) or attenuated ontogenesis (Balyaev 1979).
Our answer: As expressed above, we want to thank you for your clarifications and suggestions particularly in terms of refocusing the paper. Since the introduction has been entirely rewritten in this effort, many of the issues you have pointed out in this comment and below have been erased from the text. Whenever this is the case, we will mark it with “erased due to focus shift”.

Line 58: What are all the morphological domestication syndrome-associated traits? Details are generally very sparse and the background is too superficial.
Our answer: “erased due to focus shift”

Line 59: What physiological traits? Which traits are part of the DS and which do not fit with the hypothesis in dogs?
Our answer: “erased due to focus shift”

Line 60-61: Which behavioural traits? Which traits are part of the DS and which do not fit with the hypothesis in dogs?
Our answer: “erased due to focus shift”

Line 61- 66: This part has me a bit confused. It seems more like an afterthought and I am not quite sure how it fits in with the narrative. Are the authors saying that the domestication syndrome is not true in dogs or that it is expanded just in dogs with these extra features?
Our answer: “erased due to focus shift”

Line 64: I am quite surprised to see the uncritical and sole use of this outdated reference (Hare and Tomasello 2005), given the long-standing debate (of +20 years) on the topic and the not so few studies refuting the uniqueness in dogs being able to follow human communicative clues. Udell et al. (2008) demonstrated that wolves outperformed dogs in a pointing test when tested under identical conditions. Likewise, Wheat et al. (2022) reanalyzed previously published data claiming that dog puppies have early emerging skills in following human pointing whereas wolves do not, but upon adequately controlling for the puppies’ different environmental exposure dogs and wolves performed equally well. Furthermore, it has been demonstrated that close to 30 species (both wild and domesticated, e.g. elephants, bats, goats, pigs and not the least wolves) across eight taxonomic groups have the ability to follow pointing gestures (see Krause et al. 2018 for review).
Our answer: “erased due to focus shift”. The reference still appears in the manuscript

Powered by Editorial Manager® and ProduXion Manager® from Aries Systems Corporation

but only as a reference to Hare's emotional reactivity hypothesis. In addition, we want to thank you for pointing us to this useful reference. We included Krause's observations in line 214.

Line 73-82: While I agree that it is problematic that some studies do not address the behavioural traits of the domestication syndrome in concert, I am puzzled by the argument that standardized test batteries designed to quantify behaviours, of which some are the basic behaviours of the domestication syndrome, should somehow be unfit for hypothesis testing. The argumentation brought forward by the authors here becomes quite ironic as they a) do not test all the behavioural traits of the domestication syndrome, and b) do not test the covariance between the behaviours they do test. There is thus no actual confirmation of the domestication syndrome in their free-ranging dog population, given one would accept the premise that the behaviours chosen are in fact domestication syndrome behaviours.
Our answer: "erased due to focus shift". In addition, a) is a very fair argument and a major reason why we agreed to drop the Domestication Syndrome narrative. For point b), analyses might follow in the future if the test can be established reliably on the streets, which was the main focus of this paper.

Line 83-94: I generally agree with this paragraph. However, it is too much of a blanket statement that free-ranging dogs in general is not admixed with breed dogs. For instance, Boyko et al. has a paper from 2009 showing that more than 60% of African village dogs could be classified as non-native, i.e. with a potential admixture with modern breeds. This does not mean that free-ranging dogs are not better models for these questions than modern breeds, but introgression with modern breeds must be assumed in free-breeding populations like this.
Our answer: Thank you for this useful reference, it was a pleasure to read. Boyko et al. (2009) do indeed state that village dogs in some areas of Africa (esp. Namibia) show a high admixture rate while this admixture was low or non-existent in the other 84% of their sample. We have softened our statement (see line 84ff) to indicate that the "the majority of free-ranging dog populations are not a mixture of breeds but rather genetically distinct populations that predate the relatively more recent artificial selection and breed formation", and refer to Boyko et al. (2009).

Line 100: I am starting to get a bit confused. Are the authors testing personality, the domestication syndrome or alterative domestication hypotheses?
Our answer: "erased due to focus shift"

Line 103-122: My confusion grows. Now it sounds more like a methods paper…
Our answer: That is indeed what it was supposed to be. We hope this version is more to your satisfaction.

Line 124-126: If the battery test all these different hypotheses (which I do not believe it does, for instance there is no test of correlation between alleged domestication syndrome traits and the majority of domestication syndrome behaviours could not be adequately quantified) how come the authors label it the "Domestication Syndrome test battery"?
Our answer: "erased due to focus shift"

Line 145-146: This could create a sampling bias where only the tamest dogs participate, i.e. the most fearful dogs would not. This can ultimately affect results with exaggerated sociability scores and more fearful or less explorative dogs being underrepresented in the sample.
Our answer: That is indeed an important limitation of street tests since forcing the animals to participate would likely alter their behaviour even more, and we thus have to contend with the voluntary biased sample. While the human approach test somewhat circumvents this problem by starting the data collection before the dog is aware of being approached, we have to concede that this is a problem for all the other following subtests. We have endeavoured to address this point in the discussion (line 583ff) and mention a few possible alternatives there as well.

Line 160: Which domestication syndrome traits?
Our answer: "erased due to focus shift"

Line 193-203: This seems like a good test of human-directed sociability.
Our answer: We are glad you agree.

Line 225-253: Subtests 4-6, sure the authors wish to test other domestication hypotheses (though see my comments on pointing above), but since these tests take up half of the test battery and also seem to have the best feasibility and reliability, why then the eagerness to label the test battery the "Domestication Syndrome test battery". It seems to me, that this test battery unlikely to actually quantify domestication syndrome behaviours, if at all.
Our answer: "erased due to focus shift"

Line 204-214: If the purpose is to quantify conspecific sociability and aggression, I am not sure I understand why the authors did not simply observe behaviours toward other dogs in the area? I am not convinced this test can be disentangled from neophobia and/or boldness.
Our answer: Thank you for stating this concern. We do agree that elaborate long-term observations are the best way to assess a dog's social behaviour, whether that is pet, working, or free-ranging dog. However, this test was conceptualized to circumvent the necessity of elaborate observations to create the possibility to test more dogs without having to learn everything about their population first. In addition, we aimed at exploring the possibility of using established pet and working dog test batteries on the streets. The fake dog test has repeatedly been validated to reliably assess dogs' initial behaviour towards conspecifics (Barnard et al., 2019; Reid et al., 2022) and we were therefore positive that the same could be achieved on the street. With the outcome that the street dogs did indeed show appropriate conspecific behaviour (e.g., growling, barking, stand tall) upon first interaction with the fake dog that was not seen in the novel object test, as well as frequent genital sniffing, we argue that the test did achieve that goal and can be used to assess the target measures when only the initial reaction is taken into account. Nevertheless, we cannot exclude that neophobia and boldness come into play as soon as the animals realize that the opponent is not, in fact, a dog. We discuss this issue in line 528f as well as the value of observational data in line 614f.

Line 365-366: So, no aggression behaviour? Maybe one fear/stress/redirected behaviour (Yawning)?
Our answer: Indeed, the aggressive behaviours and many stress behaviours were rarely shown and could thus not be retained in the test-retest analysis. We discuss the problems with that as well as possible alternatives in line 602ff.

Line 387: But I thought that tail between legs had poor interrater reliability and should be excluded (given that the authors adhere to the 0.07 threshold as stated in several places, e.g. line 345, 347 and 356).
Our answer: We apologize for this confusion. The tail between the leg and mouthing behaviour were close to the threshold and showed marked significance (p=0.02). After long discussion and in light of the somewhat arbitrary 0.7 threshold that is heavily influenced by sample size, we chose to retain the two variables for this preliminary investigation and treat them with caution. However, we do agree that we should have stated that more clearly and understand your annoyance with the lack thereof. An explanation has been added in the methods (line 299f) and the result section (line 356ff).

Line 388: Could this low test-retest reliability be caused by habituation in spite modification of the fake dog. Maybe the testing set-up alone could be cause for habituation? Perhaps testing conspecific sociability and aggression is better in real life social contexts?
Our answer: Absolutely. We share this interpretation wholeheartedly, as we have explained in line 519f. Pitfalls of short retest intervals and too similar set ups are described in the paragraph thereafter (lines 522ff), and we have added the value of long-term observations in line 614.

Line 401: Again, I am puzzled by the continued use of tail between legs even though it does not meet the authors stated 0.07 ICC threshold. I find it to be borderline misleading.
Our answer: Understandably so, we apologize for this lack of explanation. Please refer

to our explanation for line 387 above for our modifications.

Line 404-405: This to me indicates both habituation, as mentioned above, but also that the fake dog was indeed treated as a novel object and not a suitable model of a conspecific.
Our answer: We agree with this interpretation and have discussed the limitations as mentioned above.

Table 3: I find it very misleading, and confusing, to include variables that did not pass the ICC thresholds.
Our answer: Again, we apologize and agree that we should have indicated this in a better way. As mentioned above, respective measures have been taken to do so.

Line 467-475: I disagree with the interpretation of the results on sociability and the conclusions brought forward here. The sample presented here is very likely to be a result of the most social dogs in the population. This is because the dogs' participation was entirely voluntarily and the dogs themselves could decide to leave the test at any given time. Since the study only includes test-retested dogs, this will inherently bias the sampling towards more social individuals and exclude fearful dogs.
Our answer: Thank you for raising this concern. You are entirely correct that the voluntary participation creates a biased sample for the overall test-battery, and we have added a paragraph in lines 583ff to address this issue. At the same time, we would like to draw attention to three points that might alleviate your concern a little in this specific instance, i.e., the sociability (now Human approach) subtest. 1) As it was the very first subtest, the animals were not first required to come to us but rather we started the approach for this subtest before the animals were aware of it. More precisely, we appeared as normal locals to the animal right until I started the (now Human approach) test by calling their attention to me with a short vocalization. Since many animals were foraging or sleeping up to that point, they did not have a chance to flee beforehand. That means that by the time the very fearful animals started to flee, the test had already started, and their behaviour was still recorded and coded as part of the (now Human approach) test. 2) to address your concern about a bias in the retesting, we did find two-thirds of the dogs again that had already fled during the (now Human approach) subtest in the first test, creating a decent sample of fearful animals that is retained in the test-retest analysis for the sociability test. 3) And finally, the fact that only the more social animals stuck around during the tests does not negate the fact that sociability might be heritable. We do not mean to claim here that street dogs are more social than wolves (since we do not have that data and the skew would indeed cause a big issue with that) but rather that the sociability measure seems to be fairly consistent over time in an individual and could therefore serve as a trait based on which selection is possible. Rereading the sentence now I can see where it might have been interpreted differently and we have specified that we refer to consistency (line 481). To additionally counteract the perceived bias during this test, we renamed it to a more neutral "Human approach test" rather than the more directed "Sociability test".

Line 526-532: If the authors wish to speculate about phenotypic plasticity, I encourage them to do so in a more detailed way. What would the adaptive value of this be in free-ranging dogs?
Our answer: Thank you for this encouragement. We have added a few lines how behavioural plasticity might aid free-ranging dogs in adapting to their unstable and variable environment, which is not a necessity for the average breed dog anymore (lines 550f).

GENERAL COMMENT Reviewer 2
There is too little known about the variation in behavior in stray/street dogs, and this study provides a survey of the types of behavioral tests that can and cannot be performed in this difficult testing environment. The study concept is thus very interesting and worthwhile. I enjoyed reading the description of traits tested and the results and discussion. As a preliminary study, I think it would be great to see the battery of tests published so that it could be retested with a larger sample.
However, I think the title, hypotheses, and design of the paper show a fundamental misunderstanding of some of the basic concepts in domestication studies.
Domestication and the Domestication Syndrome (DS) hypothesis are not equivalent

Powered by Editorial Manager® and ProduXion Manager® from Aries Systems Corporation

concepts, and the two are confounded repeatedly throughout the paper. The DS is a well-known and highly debated hypothesis that has been tested in many ways, taken apart, and found to be highly problematic. Most domestication workers agree that is has a very specific applicability when being tested. I provided more details on relevant sources in my "Itemized Review".There is no discussion of these issues in the present manuscript. The DS aims to explain the generalized traits co-ocurring in domestic populations when compared to wild counterparts. Dogs have repeatedly been criticized as a bad model for testing "early domestication" or the DS, due to their artificial breeding. In my opinion your study does not test the DS. It tests the presence of certain human-dog interactions that reflect tameness or lack of aggression in stray dogs. It is successful in that, and would be a great survey to publish as a starting point for behavioral studies in street dogs. However, as stated in my Itemized Review, the DS is not tested here. Tameness is but one trait, and it is present in all domesticated populations that have been studied thus far. I believe the authors could redesign and re-work this study with a better understanding of what they are testing. My best wishes to you and this work. I think it would be great to see this published once it is improved.

GENERAL ANSWER TO REVIEWER #2:
We would like to thank you for your helpful clarifications regarding the domestication syndrome and detailed elaborations how this paper could be improved. We agree that the focus of this paper was not ideally represented. Taking your encouragement and the suggestion of reviewer #1 into account, we chose to entirely refocus the paper towards the possibilities and limitations of free-ranging dog testing and centre our study within that. Since we do not have a large enough sample to reliably represent the distribution of specific traits across the population (as you mention, a larger sample would be needed for that), we solely focus on the presentation and reliability validation of the presented test battery to set a starting point for more elaborate testing. In addition, we discuss important considerations our results illustrate and alternatives that might be viable. These changes can be found especially in the entirely rewritten introduction and partially rewritten discussion, while the methods and result section stayed largely the same. Finally, we defined and used the terms free-ranging and street dogs more carefully and took your suggested references on board wherever possible, thank you for those pointers. We hope that by refocussing this paper on the merits it has in terms of moving the field forward, this version is more to your satisfaction.

Abstract:
A brief explanation about what is meant by “human-directed interactions” vs. “disturbance-prone behaviors” vs. “less disturbance-prone” would help the reader understand your study better from the very beginning.
Our answer: Thank you for pointing this out. We have entirely rewritten the abstract and therein explained the disturbance issue with more detail.

The term “free-ranging” is a bit vague and can be misinterpreted unless you define it specifically. For example, feral dogs like the Dingo have been described as free-ranging. Can your population perhaps be described further as “street dogs”? This is an important item because the type of population you are using relates directly to the testability of your hypothesis.
Our answer: This explanation was very helpful, thank you for that. We have added a definition for free-ranging dogs in the manuscript (line 57f), and while we continue to refer to free-ranging dogs wherever appropriate, we specifically state our use of street dogs for the test battery and adapted our interpretations and discussions to refer to the population our results can or cannot be generalized to.

Lines 44-45: the DS is not an attempt to explain domestication, but the traits associated with domestication. This may seem like a trivial difference but is really important to making
your study more scholarly.
Our answer: That is indeed an important differentiation. While we have erased the words pertaining to this from the ms, we will endeavour to be more accurate in our future work.

Introduction:
Lines 43-44: The methods/pathways used to domesticate animals are pretty well

	known (see Zeder 2012 for a good overview). It is the mechanisms by which multiple morphological/phenotypic traits correlated with domestication are co-occurring repeatedly that is misunderstood. Again, I think this is an important distinction to make throughout your paper. Domestication is something that researchers struggle to define in a simple manner—but there is much literature on this—so defining the "type" of domestication you are testing is critical. I strongly suggest that you read Zeder 2012 for and overview of the different types of processes involved in the different types of "domestication". Another view of what constitutes a "domestic" population is that of Lord et al. (several) – so there is much debate. This will inform whether your sampling is appropriate or not.<br>Our answer: Thank you for the reference suggestions. While we have erased the words pertaining to this from the ms, this is certainly a note we will keep in mind.<br><br>Line 56: Brain size reduction is not inconsistent among domesticated groups—quite the opposite. You can check Balcarcel et al. 2021 for a review of the literature on this subject and references therein, or Kruska's work from the 1970s-2000's. Your study cites Johnsson et al. 2021, which is a great paper, but I do believe there is an error in it regarding the statement of "increase" in brain size in pigs, llamas and alpacas. Johnsson et al cite Kruska (1974, 1982, 1988) and if you look at those original works, they report decreases in the telencephalon (not increases).<br>Our answer: That is an interesting thought and fact I was not aware of, thank you. Again something to keep in mind, though the line itself does not apply to the paper anymore.<br><br>Lines 59-60: There is a tremendous amount of literature debating the claims of the DS, its unsuitability in describing the variation presented by different domestic populations. I would suggest discussing this a bit, to show your understanding of the subject, before getting into testing of the DS.<br>a.For example, some domestication workers agree that dogs are a poor model for testing the DS hypothesis, to due to their directed artificial breeding during the last 200 yrs. (see Larson et al. 2014, McHugh & Larson 2017, Sanchez et al. 2016&2017 on lack of a universal pattern among domestics and the NCC).<br>b.Dogs also don't necessarily show snout shortening, as claimed by the DS. Please see Drake et al. 2010 on the variation in cranio-facial changes in dog breeds which highlights the shortening of the snout as an artificially-selected trait in many breeds.<br>Our answer: Thank you for this suggestion and we agree wholeheartedly, hence our suggestion that free-ranging dogs might be a better population. While most of this is not part of the ms anymore, we have incorporated some of it in line 80ff. Your references were very helpful in that.<br><br>IN GENERAL: You may want to rephrase or discuss the intricacies of the dog as a model for testing evolutionary processes. There is great potential in this model group, but not necessarily for testing the DS, in my opinion, since the DS is supposed to capture basic domestication traits common to all domestic populations…and dogs are highly artificially-selected. I think your study actually tests a *much broader* concept in domestication—the selection for tameness and reduced aggression—which is tremendously interesting.<br>Our answer: That is a valuable suggestion and with the rewriting of this ms we have attempted to shift the focus away from the ms and incorporated more broader domestication ideas particularly in the discussion (e.g., lines 481ff)<br><br>Lines 69-71: Here again, I think the authors may be confounding the domestication with "domestication syndrome".<br>Our answer: This line has been erased from the ms<br><br>"Free-ranging". I am concerned about the use of this term, and what it means for your sample. Hughes and MacDonald 2013 do not exclude feral populations under their definition of free-roaming dogs. Pilot et al. 2015 cite Hughes and MacDonald 2013, adding "not feral" to the definition, but I am not sure if there is evidence for that?<br>Our answer: Please see point 2 under abstract for our answer and implemented changes in regards to that.
**Additional Information:**	
**Question**	**Response**

**Financial Disclosure**<br><br>Enter a financial disclosure statement that describes the sources of funding for the work included in this submission. Review the submission guidelines for detailed requirements. View published research articles from *PLOS ONE* for specific examples.<br><br>This statement is required for submission and **will appear in the published article** if the submission is accepted. Please make sure it is accurate.<br><br>**Unfunded studies**<br>Enter: *The author(s) received no specific funding for this work.*<br><br>**Funded studies**<br>Enter a statement with the following details:<br>• Initials of the authors who received each award<br>• Grant numbers awarded to each author<br>• The full name of each funder<br>• URL of each funder website<br>• Did the sponsors or funders play any role in the study design, data collection and analysis, decision to publish, or preparation of the manuscript?<br>• **NO** - Include this sentence at the end of your statement: *The funders had no role in study design, data collection and analysis, decision to publish, or preparation of the manuscript.*<br>• **YES** - Specify the role(s) played.<br><br>* typeset	The research was funded by the Austrian Science Fund (FWF, www.fwf.ac.at: project number P34749 for GC; I5052-B for SMP, UB and FR). The funders had no role in study design, data collection and analysis, decision to publish, or preparation of the manuscript. SC received no specific funding for this work.
**Competing Interests**<br><br>Use the instructions below to enter a competing interest statement for this submission. On behalf of all authors, disclose any competing interests that could be perceived to bias this work—acknowledging all financial support and any other relevant financial or non-financial competing interests.<br><br>This statement is required for submission	The authors have declared that no competing interests exist.

Powered by Editorial Manager® and ProduXion Manager® from Aries Systems Corporation

and **will appear in the published article** if the submission is accepted. Please make sure it is accurate and that any funding sources listed in your Funding Information later in the submission form are also declared in your Financial Disclosure statement.<br><br>View published research articles from *PLOS ONE* for specific examples.<br><br>**NO authors have competing interests**<br><br>Enter: *The authors have declared that no competing interests exist.*<br><br>**Authors with competing interests**<br><br>Enter competing interest details beginning with this statement:<br><br>*I have read the journal's policy and the authors of this manuscript have the following competing interests: [insert competing interests here]*<br><br>* typeset	
**Ethics Statement**<br><br>Enter an ethics statement for this submission. This statement is required if the study involved:<br><br>• Human participants<br>• Human specimens or tissue<br>• Vertebrate animals or cephalopods<br>• Vertebrate embryos or tissues<br>• Field research<br><br>Write "N/A" if the submission does not require an ethics statement.<br><br>General guidance is provided below. Consult the submission guidelines for detailed instructions. **Make sure that all information entered here is included in the Methods section of the manuscript.**	Ethical approval for this study was obtained from the Ethical committee at the Agronomic and Veterinary Institute Hassan II (Comité d'Éthique de l'Institut Agronomique et Vétérinaire Hassan II) in Rabat, Morocco (Protocol number: CESASPV_2023_05). The free-ranging dogs' participation was voluntary, i.e., dogs were not restrained or forced to take part in the tests, and they were able to leave at any time. Moreover, the procedures were non-invasive and in accordance with the European Union Directive on the protection of animals used for scientific purposes (EU Directive 2010/63/EU).

**Format for specific study types**<br><br>**Human Subject Research (involving human participants and/or tissue)**<br>• Give the name of the institutional review board or ethics committee that approved the study<br>• Include the approval number and/or a statement indicating approval of this research<br>• Indicate the form of consent obtained (written/oral) or the reason that consent was not obtained (e.g. the data were analyzed anonymously)<br><br>**Animal Research (involving vertebrate animals, embryos or tissues)**<br>• Provide the name of the Institutional Animal Care and Use Committee (IACUC) or other relevant ethics board that reviewed the study protocol, and indicate whether they approved this research or granted a formal waiver of ethical approval<br>• Include an approval number if one was obtained<br>• If the study involved *non-human primates*, add *additional details* about animal welfare and steps taken to ameliorate suffering<br>• If anesthesia, euthanasia, or any kind of animal sacrifice is part of the study, include briefly which substances and/or methods were applied<br><br>**Field Research**<br><br>Include the following details if this study involves the collection of plant, animal, or other materials from a natural setting:<br>• Field permit number<br>• Name of the institution or relevant body that granted permission	
**Data Availability**<br><br>Authors are required to make all data underlying the findings described fully available, without restriction, and from the time of publication. PLOS allows rare exceptions to address legal and ethical concerns. See the PLOS Data Policy and FAQ for detailed information.	Yes - all data are fully available without restriction

A Data Availability Statement describing where the data can be found is required at submission. Your answers to this question constitute the Data Availability Statement and **will be published in the article**, if accepted.<br><br>**Important:** Stating 'data available on request from the author' is not sufficient. If your data are only available upon request, select 'No' for the first question and explain your exceptional situation in the text box.<br><br>Do the authors confirm that all data underlying the findings described in their manuscript are fully available without restriction?	
**Describe where the data may be found in full sentences. If you are copying our sample text, replace any instances of XXX with the appropriate details.**<br><br>• If the data are **held or will be held in a public repository**, include URLs, accession numbers or DOIs. If this information will only be available after acceptance, indicate this by ticking the box below. For example: *All XXX files are available from the XXX database (accession number(s) XXX, XXX.).*<br>• If the data are all contained **within the manuscript and/or Supporting Information files**, enter the following: *All relevant data are within the manuscript and its Supporting Information files.*<br>• If neither of these applies but you are able to provide **details of access elsewhere**, with or without limitations, please do so. For example:<br><br>*Data cannot be shared publicly because of [XXX]. Data are available from the XXX Institutional Data Access / Ethics Committee (contact via XXX) for researchers who meet the criteria for access to confidential data.*<br><br>*The data underlying the results presented in the study are available from (include the name of the third party*	All relevant data are within the manuscript and its Supporting Information files.

Powered by Editorial Manager® and ProduXion Manager® from Aries Systems Corporation

and contact information or URL).<br>• This text is appropriate if the data are owned by a third party and authors do not have permission to share the data.<br><br>* typeset	
Additional data availability information:	

Powered by Editorial Manager® and ProduXion Manager® from Aries Systems Corporation

Manuscript
Click here to access/download;Manuscript;DSAdult22_PlosOne_Manuscript_r

Street-wise dog testing: feasibility and reliability of a behavioural test battery for free-ranging dogs in their natural habitat

Svenja Capitain[1*], Giulia Cimarelli[2], Urša Blenkuš[2], Friederike Range[2], Sarah Marshall-Pescini[2*]

[1] Department of Physics, Chemistry and Biology (IFM), Linköping University, Linköping, Sweden

[2] Domestication Lab, Konrad Lorenz Institute of Ethology, Department of Interdisciplinary Life Sciences, University of Veterinary Medicine Vienna, Vienna, Austria

* Corresponding author

Svenja.capitain@vetmeduni.ac.at (SC)

Sarah.marshall@vetmeduni.ac.at (SMP)

Abstract

Behavioural scientists are increasingly recognizing the need to conduct experiments in the wild to achieve a comprehensive understanding of their species' behaviour. For domestic dogs (*Canis familiaris*), such progress is lagging behind. While the life in human households is often regarded as dogs' natural habitat, this classification disregards most of the global dog population. The value of including free-ranging dogs as study subjects is increasingly being recognized, but no comprehensive behavioural and cognitive test batteries have been conducted so far and their feasibility and reliability are unknown. This study is the starting point to fill this gap by pioneering and validating an elaborate behavioural test battery with street-living dogs. Therein, six common temperament tests (human-/conspecific-directed sociability, neophobia, tractability) and dog-human communication paradigms (pointing, inaccessible object) were adapted to the street conditions. We evaluated the feasibility of the test battery, the coding reliability of the measures, and investigated their temporal consistency in a retest of the same individuals six weeks later (test-retest reliability). The test battery proved feasible with most dogs participating in all subtests, and it showed satisfactory inter- and intra-rater reliability (0.84 and 0.93 respectively), providing evidence that complex behavioural tests can be conducted even in highly variable street conditions. Retesting revealed that some behaviours could be captured reliably across time, especially when the subtest was particularly engaging (e.g., human approach, point following). In contrast, the low retest reliability for subtests relying on sustained novelty and behaviours that were highly susceptible to disturbances (e.g., gazing) reflects the difficulties of street dog testing, including standardisation in disturbance-prone environments, ecology-dependent adaptation of methods, and intrinsic differences between pet and free-ranging dogs. Bearing the suggestions made in the discussion in mind, this test battery can be valuable in investigating cognition, behavioural profiles, and domestication in such an understudied population as free-ranging dogs.

Introduction

The behaviour of dogs has been of central interest to humanity for millennia – possibly since the early stages of dog domestication 15.000 years ago, hypothesised to have come about through the selection for specific

behavioural traits [hypothesised as: tameness: 1, lack of aggression: 2, sociability: 3, and/or deferential behaviour: 4]. While dogs appear already in the work of early scientist such as Darwin [5] or Pavlov [6], it is only in the last 30 years that the research into dog behaviour has been recognized for its intrinsic value, resulting in an explosion of studies focusing on multiple aspects from understanding of human communication, theory of mind, learning processes, and beyond [reviewed by 7, 8]. Likewise, comparative studies have seen a surge of interest in dogs' temperament traits and personality [9], with comparisons to other species shedding light on the profound influence of our own anthropogenic environment [e.g., as model for aging: 10] and domestication [reviewed in 11] on behavioural development.

Importantly, ~~however,~~ these efforts have primarily revolved around a relatively limited subset of the global dog population – those under immediate human care and control, such as pets, working, and shelter dogs [9]. While easy to access, this subset represents less than a quarter of the global dog population with the other 75-83% consisting of free-ranging dogs [12, 13]. These animals might or might not be owned but share the characteristic that their movement and mate-choice are largely unrestricted by human control [14], even though the majority still resides as street dogs that depend on human settlements for food and shelter [12].

Efforts to explore the behaviour of free-ranging dogs have so far predominantly relied on observational methods [e.g., 15, 16, reviewed in 17]. While this has led to a more in depth understanding of these populations, it is comparatively time-intensive and lacks the experimental manipulation required to answer some of the questions being addressed in the lab with pet, shelter and working dogs. Several challenges come to mind when hypothesizing the use of more standardized test batteries for free-ranging dogs in their natural habitat: First, with little previous knowledge of the test subject, direct interaction might be hazardous regarding aggression or disease status [e.g., rabies: 18]. Second, restraining the animals for a test would be unethical, potentially dangerous, and obstruct the testing of natural behaviour. Hence, tests must rely on voluntary participation and transportable, flexible test setups. Thus, third, the lack of a stable test environment impedes standardisation and the variability and disturbance risk might distort results [19]. Fourth, our limited knowledge of free-ranging dogs' behaviour during experiments requires adaptation of

methodologies and apparatuses used with other dog populations, with some inevitable loss of direct comparability, given the different contexts in which animals are tested [20-22]. Finally, repeated testing remains a challenging prospect given the rapidly changing dynamics in free-ranging populations [reviewed in 17, 21, 23].

Nonetheless, there are compelling reasons to experimentally assess the behaviour and personality of free-ranging dogs. For one, without studying a representative sample of the global dog population encompassing at least some of the vastly different life experiences they may have, our knowledge about dogs' cognitive abilities, human-animal interactions, the connection between genetics and personality, and beyond remains compromised [for more specific ideas see 19, 21]. This is particularly true for evolutionary questions and the understanding of the domestication process. The intensive artificial selection in our modern breed dogs might have de-coupled morphological and behavioural traits hypothesized to co-occur in domestic species [24, 25] and the use of modern breed dogs as a model for domestication has therefore been contested by a number of authors [26, 27]. In support of this and contrary to the general assumption, the majority of free-ranging dog populations are not a mixture of breeds but rather genetically distinct populations that predate the relatively more recent artificial selection and breed formation [14, 28]. Thus free-ranging dogs might actually be ~~a~~ better candidate to elucidate the main domestication process in dogs from a genetic and ecological perspective [29], than modern breed dogs living in 'western-style' pet-owner relationships [30]. Furthermore, free-ranging dogs' pervasive presence in human settlements (e.g., 2,930 dogs/km² in Kathmandu, Nepal [31]) means that millions of people spend their daily life in dogs' direct proximity. Improving our understanding of their behaviour and behavioural profiles could therefore improve both human and animal welfare and aid in effective population control [19, 29].

Some pioneering groups have already initiated important work ~~with~~ free-ranging dogs, demonstrating that an experimental approach, particularly if involving short, one-off tests can indeed be conducted under the variable yet natural conditions they reside in [20, 22, 32-34]. While presenting a valuable start, these tests have focused on single behavioural aspects in short tests [e.g., persistence in 20, pointing in 22], leaving the

possibility of longer tests, test batteries, and repeated testing that is needed to explore behavioural profiles, largely unexplored.

Furthermore, critical information regarding the reliability of measures obtained from such tests on the streets remains scarce – information that is vital not only for singular behavioural testing but especially for comprehensive temperament assessments. Several aspects of reliability should therein be considered: the coding reliability, which describes how well the behaviours defined in the ethogram and the testing conditions allow for consistency in a raters' observations over time ("intra-rater reliability") and with others ("inter-rater reliability"), and the "test-retest reliability", which assesses a test's ability to capture the behavioural traits reliably. A reliable test would therein be expected to elicit the same behaviour in an individual again when being tested in the same test twice ("retest"). This is particularly important in terms of personality traits, which are defined as being consistent over time and context in an individual [9]. Test-retest reliability outcomes are often rather low in pet breed and shelter dogs, but some kind of consistency over time has often been reported [35, 36]. Whether this will pertain to free-ranging dogs and/or under highly variable street conditions remains to be explored.

In response to this research gap, our study endeavours to address a twofold objective. First, to pilot and evaluate the feasibility of a test battery specifically tailored to street dogs, encompassing a range of behaviours commonly assessed in dog cognition or temperament tests and suggested to be relevant in different domestication hypotheses. These behaviours include human-directed approachability, conspecific sociability through a fake dog test, neophobia and exploration in a novel object test, dogs' understanding of human communication in a pointing test and their use of human-directed communication (gazing) in a begging test, as well as their propensity to shy away from conflict in a tractability test [37-41]. Second, we assess the reliability of this test battery in the natural setting, including inter-rater, intra-rater and test-retest reliability. We draw conclusions from the results on how this test battery could be adapted to be more pervasively used in the field and free-ranging dog testing overall.

Methods

Ethics statement

Ethical approval for this study was obtained from the Ethical committee at the Agronomic and Veterinary Institute Hassan II (Comité d'Éthique de l'Institut Agronomique et Vétérinaire Hassan II) in Rabat, Morocco (Protocol number: CESASPV_2023_05). The street dogs' participation was voluntary, i.e., dogs were not restrained or forced to take part in the tests, and they were able to leave at any time. Moreover, the procedures were non-invasive and in accordance with the European Union Directive on the protection of animals used for scientific purposes (EU Directive 2010/63/EU).

Study area and population

The testing of the study subjects was conducted along beach, industrial, and urban areas in the Sous-Massa region in Morocco. The study subjects were part of a big dynamic free-ranging dog population living as scavengers in and around human settlements, hence classifying as street dogs [21]. The behaviour battery was first piloted on a random sample of eight adult street dogs (4 males, 4 females). Since the test battery was finalized after the first three and thus remained the same for the other five pilot dogs, they were added to the final test sample. Hence, a total of 36 adult street dogs (20 males, 16 females; 5 pilot plus 31 test sample dogs) were tested to assess the feasibility and reliability of the finalized test. Twenty-six of those dogs (15 males, 11 females) were found again for the retest.

Procedure

The test battery consisted of six short behavioural subtests and three physiological samplings conducted in direct succession (Fig 1). The subtests were designed to resemble behaviour tests that have commonly been used to assess pet, shelter, or working dog temperament traits, dogs' cognitive abilities, or traits that have been suggested as central during the domestication process (see description of the respective subtests). The three physiological samples (saliva and hair) were part of the test battery to assess the feasibility of sampling in such a setting and allow for later use of those samples in future exploration.

Fig 1. The sequence of the employed test battery. Subtests in light and physiological sample collections in dark grey.

For the test-retest reliability measure, the test battery was conducted a second time ("retest") with the same study subjects within an average time span of 46.28 days (min. 33, max. 76 days) which was similar to other test validation procedures [e.g., 42, 43-45]. To this end, the location of the initial test was noted, and the area was frequently visited again after five to six weeks until the dog was found, identified with previous pictures, and tested again. It was noted if the dog was retested in a different location than the first test (i.e., not in visual vicinity of the previous location). The procedure in the first and retest was exactly the same as described below. For the object-based subtests (Fake dog and Novel object subtest, see below), two slightly different-looking versions were used respectively for the test and the retest to create novelty. The order of presentation of the two stimuli was randomized across subjects.

Initiation and general circumstances

Two experimenters were involved in each testing occasion: E1 was always the same person (SC) and conducted the actual tests while E2 (three different people) video-recorded, helped set up the subtests, or distracted other dogs if necessary. Dogs were tested individually and had to be alone before and during the test. Tests were conducted between sunrise and 10 a.m. before the areas became too crowded. The experimenters accessed the study area by car and all dogs encountered alone were tested. If at any point during testing, the dog ran off more than 10 m, the subtest during which the dog left was terminated and E1 tried to lure the dog back. If the dog ran too far to be retrieved, or did not respond to the lure, the whole test was terminated. If anyone other than the experimenter approached and visibly distracted the dog (e.g., head turn), the test was interrupted until the criterium was restored and the test resumed. If at any point dogs showed active signs of aggression, the test was terminated (however, this never occurred). For each dog, location, time, weather, health status, and activity upon arrival of E1 (e.g., sleeping, walking, …) were noted.

The exact setup of each subtest is displayed in Fig 2, the procedure of each subtest is demonstrated in the supporting information (S1 Video).

Fig 2. The set-up of each of the six subtests in the presented test battery. The main experimenter (E1) is presented in black (or grey in later stages of the subtest), the assistant experimenter (E2) in white.

Human Approach Test (Subtest 1)

Target: Approachability, human-directed sociability/ docility, tameness, play. This test was modelled after similar tests used in widescale personality assessment tests (e.g., the Dog Mentality Assessment (DMA) in the Swedish Kennel Club [46] and has in part been used with Ethiopian village dogs [34]. Beside the assumption that human-directed sociability is an essential trait under selection during the domestication process [3], assessing the actual human-directed behaviour in a free-ranging population is provides valuable insights for disease- and population control.

Here, E1 stepped out of the car and whistled shortly to grab the dog's attention. She then stepwise proceeded to 1) stand quietly looking at the subject for 30 s, 2) called the dog whilst making friendly gestures (snapping, tapping on leg) for another 30 s, 3) approached the dog for 30 s up to 2 m distance, 4) stood for another 30 s, 5) approached the dog to one body length distance and paused another 30 s, and 6) crouched for another 30 s all while continuing to call the dog over. If the dog approached to less than 10 cm of E1 during any of those phases, E1 kneeled and tried to pet the dog for 30 s. If tolerated, E1 used a small brush to collect hair samples. The dog was then invited to play tug of war with a rag toy (1 m long) for 30 s.

Fake Dog Test (Subtest 2)

Target: Conspecific-directed sociability and aggression. Several studies support the idea that stuffed dogs can be used to reliably assess dogs' initial behaviour towards conspecifics [37, 47]. A reduction in conspecific aggression has been hypothesised as an important shift from wild to domestic types [48], and understanding intraspecific aggression in free-ranging dogs can provide important applications for the local population.

Hence, subjects were exposed to a “Giant Jack Russel Terrier” stuffed fake dog (brand: ‘Melissa & Douk’; wither height 28 cm; S2 Supplementary Information). The two used versions differed in ear posture (pointy or droopy), tail length (medium and long), and colour (one original, one wrapped in dark bags) in the test and in the re-test respectively, in a randomized order across subjects. The fake dog was positioned 5 m from the car behind a V-shape visual barrier with its opening towards the car. This visual barrier allowed only the approaching subject but no other surrounding dogs to see the fake dog. E1 guided the subject towards the fake dog (if needed with food) and quickly walked >5 m away as soon as the subject saw the fake dog. The subject’s behaviour was observed for 2 min.

Novel Object Test (Subtest 3)

Target: Neophobia (exploration, novelty-seeking, curiosity). Presenting a dog with an unknow object is commonly used to assess novelty-seeking and boldness [41, 49], and explorative-behaviour is hypothesised to be lower in domesticated animals as part of the domestication syndrome [48].

To present an object that was truly novel to the dogs, we fixed a 90 – 100 cm high foil balloon with a wooden stick on top of a remotely controlled car (31x18x18 cm, Model: DEERC DE42 RC, S2 Supplementary information). Two balloon forms were used (dinosaur, a number (zero, five) sprayed in neon green and blue) for test and retest and randomized between study subjects. The set up was the same as the Fake dog test. As soon as the dog saw the novel object, E2 started moving it back and forth for 1 min through remote control. The toy was then left inactive for another 1 min. The dog’s behaviour was observed for the full 2 min.

Pointing Test (Subtest 4)

Target: Dog-human communication skill, willingness to follow commands, cooperative communication [reviewed in 30, 50]. Studies have shown that a wide array of species, including canids, can follow pointing cues [for review see 50]. Exploring this phenomenon in free-ranging dogs with differing levels of human experience will further our understanding of the role of life-experience vs. species predisposition (but see [22] for pioneering work in this regard).

The test started with four warm-up trials: E1 placed a baited bowl (12 cm diameter, 10 cm height) on the ground 2 m from the dog, standing 1 m away herself and verbally called the dogs attention. After the dog ate the food, the bowl was picked up, baited, and placed down again to familiarize the dog with the fact that the bowl contained retrievable food. The subtest was terminated if the dog did not approach the bowl within 30 s two times in a row. The warm-up trials were followed by twelve test trials: one empty and one baited bowl (both rubbed in food for smell, baited outside of the dog's view) were placed 1.5 m apart 2 m from the dog. E1 stood equidistant behind the bowls, attracted the dog's attention, and upon eye contact, pointed to the baited bowl for 2 s (momentary distal pointing). The dog was allowed to eat the food if it approached the correct bowl on the first choice, otherwise the bowls were immediately collected by E1. Each trial could be repeated twice if the dog did not choose within 30 s, followed by one repetition of the warm-up trial (i.e., only one bowl with food). If after the third repetition, the dog did not choose, the subtest was terminated. We used six different randomized sequences related to the positioning of the baited/pointed bowl to the right or left of the experimenter (e.g., RLRLLRLRRLLR) that were called out to E1 by E2.

Begging Test (Subtest 5)

Target: human-directed communication, gazing behaviour, suggested to be help-seeking behaviour [38, reviewed by 51, but see for alternative explanations: 52, 53]. Also a behaviour suggested as a trait under selection during domestication but the wide variability between individuals depending on human socialisation calls for further investigation with differentially socialised populations [54, 55].

The dog was allowed to eat from the baited bowl three times. Afterwards, the bowl was visibly baited and placed out of reach but still visible on the car, a wall, or a tree. E1 stepped 1 m to the side and faced away from the bowl with a neutral facial expression. The dog's behaviour was observed for 1 min.

Tractability test (Subtest 6)

Target: Social inhibition, tractability/deferential behaviour (i.e., the ease to yield to higher command, conflict avoidance [4]). According to the deferential hypothesis [4], tractability has been a central selection

factor in dog domestication. Furthermore, assessing dogs' inclination to avoid conflicts with humans in a resource scenario may also have it uses in a more applied setting.

E1 gave the dog a piece of food, then took a big piece of meat, showed it to the dog, and visibly placed it on top of a small stool (40 cm height). E1 loudly stepped between dog and stool, stared the dog in the eyes and firmly said "No, it's mine!". If the dog attempted to get to the stool, E1 loudly stepped in their way and repeated "No" in a firm tone. After 1 min of this, E1 stepped 1 m to the side and stood still for 30 s while looking at the food. Finally, E1 turned around and looked away for 30 s. The test was finished as soon as the dog ate the food or after the last 30 s.

Video coding

The tests were recorded by an action camera (4K 24fps WiFi Sports Action Camera or Action Cam Jeemak WiFi) fixed on E1's chest and a camera (Sony HDR-CX405 Full HD Camcorder, hand-held (subtest 1) or on a tripod (subtest 2-6)) handled by E2. BORIS (version 7.13.8, (Friard & Gamba, 2016)) was used for the video coding.

The ethogram aimed at capturing the target behaviours described for each subtest. We used a bottom-up approach by initially coding 55 variables with different modifiers (i.e., the person or object the behaviour was coded towards) that measured almost every potentially relevant aspect in the different subtests. The coded variables are displayed in Table 1 with a detailed ethogram in S3 Table. Social behaviours were based on the ethograms used at the Wolf Science Center of the University for Veterinary Medicine, Vienna [11], and to observe free-ranging dogs in Italy [16, 56].

This list of variables was narrowed down by 1) assessing their codability through the inter- and intra-rater analysis (for acceptable agreement see Statistical Analysis), 2) assessing the variables' centrality in the subtest by analysing their frequency (i.e., excluding variables that were displayed in less than 10% of tests), and 3) assessing their consistency through the test-retest reliability. Only behavioural variables that passed step 1) and 2) went on to be analysed in step 3) to ensure that the test-retest reliability was based on reliably coded and not overly skewed data [57].

Table 1. Coded behavioural variables and occurrence across subtests.

Category	Behavioural variable	Human approach	Fake Dog	Novel Object	Pointing	Begging	Tractability
**Proximity**	Close	E1	Fake dog, E1+E2	Novel object, E1+E2	-	E1	E1, stool
	Medium	E1	Fake dog, E1+E2	Novel object, E1+E2	-	E1	E1, stool
**Tail**	Wagging	E1	Fake dog	Novel object	E1	E1	E1
	Between legs	Yes	Yes	Yes	-	Yes	Yes
**Gazing**		E1	Fake dog	Novel object, E1+E2	-	E1	E1
**Vocalization**	Barking	E1	Fake dog	Novel object	-	E1	E1
	Growling	E1	Fake dog	Novel object	-	E1	E1
	Whining	E1	Fake dog	Novel object	-	E1	E1
**Displacement**	Nose-licking	Yes	Yes	Yes	-	Yes	Yes
	Body shake	Yes	Yes	Yes	-	Yes	Yes
	Stretching	Yes	Yes	Yes	-	Yes	Yes
	Yawning	Yes	Yes	Yes	-	Yes	Yes
	Sniffing the ground	Yes	-	-	-	-	-
**Physical contact**	Biting	E1	Fake dog	Novel object	-	E1	E1
	Body contact	E1	Fake dog	Novel object	-	E1	E1
	Jumping	E1	Fake dog	Novel object	-	E1	E1
	Licking	E1	Fake dog	Novel object	-	E1	E1
	Mouthing	E1	Fake dog	Novel object	-	E1	E1
	Pawing	E1	Fake dog	Novel object	-	E1	E1
	Sniffing	E1	Fake dog	Novel object	-	E1	E1
**Reactions**	Stand tall	E1	Fake dog	Novel object	-	E1	E1
	Bare teeth	E1	Fake dog	Novel object	-	E1	E1
	Lunge	E1	Fake dog	Novel object	-	E1	E1
	Head dip	E1	Fake dog	Novel object	-	E1	E1
	Belly exposure	Yes	Yes	Yes	-	Yes	Yes
	Flee	E1	Fake dog	Novel object	-	E1	E1
	Crouch	E1	Fake dog	Novel object	-	E1	E1
	Risk assessment	E1	Fake dog	Novel object	-	E1	E1
	Play	E1	Fake dog	Novel object	-	E1	E1
	Friendly	E1	Fake dog	Novel object	-	E1	E1
	Bow	E1	Fake dog	Novel object	-	E1	E1
**Marking**		Yes	Yes	Yes	-	Yes	Yes
**Defecating**		Yes	Yes	Yes	-	*Yes*	Yes
**Non-visible**		Yes	Yes	Yes	Yes	Yes	Yes
**Disturbance**		Yes	Yes	Yes	Yes	Yes	Yes
**Subtest specific variables**			Latency to approach fake dog	Latency to approach novel object	Observation of gesture: no/yes	Attempts to reach bowl	Latency to eat food
		Phase of first approach		Phase of first approach	Successful choice: no/yes		Phase of eating food

	Genital sniffing	No choice	2-way gaze alternation
			3-way gaze alternation

The table shows all behavioural variables (column 2) of a certain category (column 1) that were coded in the respective subtests (columns 3 - 8). The words in the subtest columns describe towards what person or object the behaviour was coded, while 'yes' means that the behaviour was coded regardless of direction, and a minus (-) indicates that the behaviour was not coded in the subtest. Behavioural variables were either coded as duration (D), frequency (F), occurrence (O), or latency since the beginning of the respective subtest (L). Coloured cells indicate that the variable occurred in less than 10 % of tests and was thus excluded from the test-retest analysis.

For the intra-rater reliability measures, a random subsample of 20% of the test videos (n=13; balanced between test and retest, full test batteries and tests that were terminated halfway through) were re-coded by the initial rater after three weeks. For the inter-rater reliability, a random subsample of 20% of the full-test videos (n=9) were coded independently by a second rater [41, for similar methods see 58]. Beforehand, the second rater was trained on three sample videos and the ethogram was further clarified where needed.

Statistical analysis

Inter- and intra-rater reliability was analysed for each behavioural variable summed across all subtests and modifiers to assess their codability. Test-retest reliability, on the other hand, was analysed for every single variable by modifier and subtest. Occurrence variables (e.g., in which phase the dog approached, termination, etc.) were converted into ordinal variables before analysis. To normalize the data for comparison between test and retest, the time the dog was not visible or a disturbance was coded was subtracted from the total time of the subtest of the individual, and the behavioural data was then divided by this normative time. All individual subtests in which more than 70% of the time was coded as disturbance and/or not visible were excluded from further analysis. In case of the test-retest reliability, the specific subtest in both the test and the retest were excluded to allow for fair comparison.

The statistical analysis was then conducted in R (Version 4.2.2 [59]). To account for the systematic errors between raters/rating occasions, intraclass correlation coefficients (ICCs) were calculated to assess the

reliability measures [60, 61]. Additionally, considering that both raters/rating occasions coded the same videos and that the goal was to generalize the reliability results, a two-way random effects ANOVA with an absolute agreement estimate was used to assess the inter- and intra-rater reliability [reviewed by 62]. According to the classification by [63], an ICC below 0.5 is considered poor reliability, between 0.5 and 0.75 it is moderate, between 0.75 and 0.9 it is good, and between 0.9 and 1.0 the reliability is considered excellent. For a behaviour to be regarded as having acceptable coding reliability and move on to the test-retest analysis, the ICC threshold for the inter- and intra-rater reliability was set to a moderate 0.70 [64] which is in accordance with similar studies [weighted average of 0.68 in a review of six studies by 9, 0.77 in 65]. However, careful reconsideration was employed in case the threshold was just about missed by taking both coding reliabilities (inter- and intra-rater) into consideration and examining the p-value (assuming significance level $\alpha=0.05$). For the test-retest reliability in the repeated sample, a two-way mixed effects ANOVA with absolute agreement estimate was applied [63]. Since significance largely depends on sample size and significant test-retest reliabilities in pet dog tests commonly range around an ICC of 0.5 which would usually be classified as poor to moderate reliability [average ICC of 0.43 in a meta-analysis of 31 studies in 35, e.g., 0.58 in 36, 0.57 in 41], a test-retest reliability with a p-value below 0.05 rather than a specific ICC was chosen as reliability indicator. Only variables that were displayed in more than 10% of the testing occasions were included in the test-retest reliability to circumvent unreliable analysis of heavily zero-inflated and low-prevalence data [for a similar approach see 57, 64, 66]. For the ordinal variables, two-way ANOVA's with absolute agreement estimate (equivalent to weighted Cohens's Kappa according to [67]) were used to analyse the coding and test-retest reliability. If the ICC was poor (i.e., below 0.5) for a certain variable, systematic biases in the measurements of both rating occasions were explored through scatter plots and variables were excluded if necessary. To control for the test-retest reliability being low due to disturbances during the tests, we conducted the same analysis again but excluded all individual subtests in which any disturbances were coded (46 of 235 subtests). Disturbance had been coded when the dog clearly turned its head towards an interfering human/dog/other animal for more than three seconds during a running subtest and while the test subject was visible on the video.

Lastly, because curiosity- or exploration-based tests may be more prone to lower consistency due to repeated exposure to similar stimuli [49], we tested if the dogs systematically showed a decreased reaction (exploration and fear) in the fake dog and novel object retest compared to the first test. To that end, applicable variables that were retained in the test-retest reliability (i.e., had good coding reliability and occurred in more than 10% of tests) were summed into an exploration-related and a fear-related variable [therein drawing from 68, 69] for each of the two subtests respectively. After transforming these four left-skewed variables with a square root transformation, a paired t-test was used to compare whether they differed significantly between test and retest.

Results

Subject characteristics

Out of the 36 initially tested street dogs, 23 (14 males, 9 females) were tested in the entire test battery, while nine dogs (5 males, 4 females) terminated the test during the Human approach subtest and four dogs (1 male, 3 female) after the first two or three subtests by running away. For the retest, 26 and thus 72% of the 36 initially tested street dogs were found again: 19 of those were dogs who went through the full test battery initially and 7 were dogs who had run away during the first testing. Fourteen were retested in a different location than the first test. All 26 dogs completed the same number of subtests in the retest as they did in the initial test.

Changes in methods and variables

After the test was adapted in the pilot testing, some additional changes from the above-presented procedure had to be made throughout the main testing period. For one, the play-part (rope presentation for tug-of-war) in the Human approach test caused four (28.57%) of the first 14 dogs to run away and terminate the test. For two more, the play phase had to be terminated to keep the dog from leaving. Thus, the play phase was dropped half-way through the main sample and the variable ‘Interacting with the toy’ was excluded from the analysis.

In addition, two more variables had to be excluded due to the following reasons: 'Auto-grooming' because the dogs had varying amounts of parasites, wounds, and dirt in their fur which influenced the scratching rate, and 'Sniffing the ground' in all but the Human approach test because food was dropped on the ground during the subtests and the different ground surfaces influenced the dogs' sniffing behaviour.

Intra- and inter-rater reliability

Both the intra- and inter-rater reliability coding, assessed across all subtests and modifiers, is reported in Table 2. Almost all variables passed the target ICC-threshold of 0.7 and had a significant p-value at 0.015 or below (the exact statistical outputs for each variable are reported in S4 table). The average of both inter- and intra-rater reliability for all variables was above the target threshold of an ICC of 0.7, with a good reliability of 0.84 in the inter-rater and 0.93 in the intra-rater analysis.

There were only few variables that did not clear the threshold. 'Tail between the legs' and 'Mouthing' had moderate inter-rater reliability, meaning they still had an ICC above 0.5, and the p-value was below 0.05. Likewise, 'Medium proximity' had a moderate reliability within and between raters. The inter-rater reliability for 'Head dip', 'Friendly' and 'Stand tall', on the other hand, were poor with an ICC below 0.5 with a p-value above 0.05.

While 'Tail between the legs' and 'Mouthing' did not clear the initially defined threshold, they were both close and had a p-value of 0.02 which is substantially below the usual 0.05 significance level. Since previous studies have deterred from the arbitrary 0.7 ICC threshold in favour of significant p-values (p<0.05) [9], and both present crucial behaviours in dogs, we tentatively chose to keep them in the analysis going forward but treat their interpretation with utmost care. The other behaviours fell below the 0.05 significance level and were thus excluded from further analysis.

Table 2. Summary of the coding reliability across subtests.

**GENERAL**					**SUBTEST SPECIFIC**			
**Category**	**Behavioural variable**	**Inter-rater ICC**	**Intra-rater ICC**		**Subtest**	**Behavioural variable**	**Inter-rater ICC**	**Intra-rater ICC**

**Proximity**	Close	0.90	0.97
	Medium	0.53	0.52
**Tail**	Wagging	0.91	0.91
	Between legs	0.64	0.99
**Gazing**		0.90	0.98
**Vocalization**	Barking	NA	NA
	Growling	0.80	NA
	Whining	NA	NA
**Displacement**	Nose-licking	0.88	0.96
	Shaking	0.80	NA
	Stretching	0.88	1.00
	Yawning	0.94	0.97
	Sniffing the ground	0.82	0.79
**Physical contact**	Biting	NA	NA
	Body contact	1.00	0.74
	Jumping	0.95	0.98
	Licking	NA	0.84
	Mouthing	0.64	0.95
	Pawing	NA	0.99
	Sniffing	0.97	0.98
**Reactions**	Stand tall	-0.03	0.91
	Bare teeth	NA	NA
	Lunge	NA	NA
	Head dip	-0.08	0.85
	Belly exposure	NA	NA
	Flee	0.77	0.94
	Crouch	0.90	0.79
	Risk assessment	0.99	0.86
	Play	0.98	0.98
	Friendly	0.44	0.97
	Bow	0.81	0.76
**Marking**		NA	0.99
**Defecating**		NA	NA

**Human approach**	Phase of first Approach	1.00	1.00
**Fake Dog**	Latency	1.00	0.97
	Genital sniffing	0.92	1.00
**Novel object**	Phase of first approach	1.00	1.00
	Latency	0.85	1.00
**Pointing**	Observation of gesture: yes	1.00	0.98
	Observation of gesture: no	1.00	0.99
	Successful choice	1.00	1.00
	Unsucessful choice	1.00	1.00
	No choice	1.00	1.00
**Begging**	Attempts to get bowl	0.99	0.99
**Begging/ Tractability**	2-way gaze alternations	0.82	0.79
	3-way gaze alternations	0.86	0.87
**Tractability**	Latency	0.90	1.00
	Phase of eating	1.00	1.00

**Average**		**0.84**	**0.93**

For each variable, the inter- and intra-rater reliability analysed across subtests and modifiers is displayed. Based on similar studies, a coding reliability of an ICC of 0.7 or above was regarded as acceptable (values with no specific highlight). Moderate reliability below this threshold (i.e., 0.5<ICC<0.7) is highlighted in light grey, and poor reliability (i.e., ICC<0.5) is marked in dark grey.

All variables above an ICC of 0.7 had a p-value below 0.015, variables with an ICC between 0.5 and 0.7 had a p-value below 0.05, and all variables below an ICC of 0.5 had a non-significant p-value ($p>0.05$). Detailed analysis results with the 95% confidence interval, F-value statistic, degrees of freedom, and p-value can be found in S4 Table. Variables marked with NA did not occur in the coded tests.

Exclusion of infrequent variables

In the next step, variables that did not occur in more than 10% of the respective subtests were identified (Table 1) and excluded from the test-retest reliability analysis. In short, close proximity, tail wagging, gazing, nose licking and yawning were the only variables that occurred frequently in all subtests. On the other hand, growling, whining, stretching, shaking, biting, licking, mouthing, marking, defecating, baring teeth, lunging, belly exposure, and bow were not displayed in more than 10% of tests in any of the subtests. Even after summing most of them into one pooled 'stress' variable [following the classification of 70, 71], they did not pass the 10% occurrence threshold and were hence excluded from the test-retest analysis. All subtest-specific variables except 'attempt to reach the bowl' in the begging test, occurred frequently enough to pass the 10% threshold.

Test-retest reliability

The final test-retest reliability analysis was conducted for each variable by subtest and modifier, only taking the variables into account that 1) were not already dropped from the ethogram/tests throughout the testing (i.e., auto-grooming, sniffing the ground, play), 2) had an acceptable coding reliability, and 3) occurred in more than 10% of all tests. The resulting test-retest reliabilities are reported in Table 3.

The average test-retest reliability was poor with an average ICC of 0.23. To see whether the poor outcome was influenced by disturbances during the tests that altered the dogs' behaviour, we conducted the same analysis but excluded all subtests in which a disturbance had been coded (i.e., when the dog clearly turned its head towards an interfering human/dog/other animal for more than three seconds during a running subtest and while the test subject was visible on the video). While this changed the reliability of some behaviours,

the average test-retest reliability did not improve (Table 3; the exact statistical outputs of both analyses can be found in S5 Table).

To summarize by subtest, the Human approach test performed well with ten out of 15 behaviours showing significant test-retest reliability, resulting in an average test-retest reliability of 0.32. In the Fake dog test, only ‘tail between the legs’ had a moderate retest reliability, with ‘close proximity to the fake dog’ and ‘nose-mouth licking’ also being moderately reliable after tests with disturbances were removed. The average test-retest reliability was low (ICC = 0.06). Similarly, only ‘close proximity to E1 and E2’ were significant in the Novel object test, being joined by ‘nose-mouth licking’ after disturbed tests were excluded, resulting in an average test-retest reliability of 0.12. The average reliability of the Pointing test was significant with an average ICC of 0.51 with all subtest-specific variables, as well as ‘tail-wagging’, showing moderate test-retest reliability. The Begging test had a low test-retest reliability (average ICC = 0.16) with only the ‘close proximity to E1’ being significant across time when only looking at tests without disturbances. Lastly, ‘close proximity to E1’, ‘gazing at E1’, the ‘phase in which the dog ate the food’, ‘three-way gaze alternation between person and stool’ and ‘fleeing’ had significant reliability in the Tractability test, with the addition of ‘nose-mouth licking’ after disturbances were removed. The average test-retest reliability was 0.22.

Test-retest comparison for the curiosity-based subtests

The summed variables constituted of the following behaviours: “Exploration”-variable: ‘close’ and ‘medium proximity’, ‘gazing’, ‘tail wagging’, ‘friendly’, and ‘sniffing’ the fake dog/novel object, as well as ‘genital sniffing’ in the case of the fake dog. “Fear”-variable: ‘head dip’ and ‘tail between the legs’ for the fake dog subtest. Only ‘flee’ was retained as fear-representing behaviour in the novel object but was displayed so rarely, that it could not be analysed here.

The dogs showed significantly less exploration and less fear behaviours towards the fake dog in the retest than the first test (exploration: $t(14)=3.16$, $p<0.01$; fear: $t(15)=-3.08$, $p<0.01$). Exploration behaviours shown towards the novel object did not differ between test and retest ($t(11)=1.0$, $p=0.34$).

Table 3. Summary of the test-retest reliability in all retained variables.

Category	Behavioural variable	Human approach			Fake Dog			Novel Object			Pointing			Begging			Tractability		
		**M.**	**All**	**w/D**	**M.**	**All**	**w/D**	**M.**	**All**	**w/D**	**M.**	**All**	**w/D**	**M.**	**All**	**w/D**	**M.**	**All**	**w/D**
**Proximity**	Close	*E1*	0.71	0.77	*FD*	0.20	0.74	*NO*	-0.09	0.06	-			*E1*	0.8	0.76	*E1*	0.75	0.72
					E1+E2	-0.08	-0.17	*E1+E2*	0.74	0							*Stool*	-0.04	-0.02
**Tail**	Wagging	*E1*	0.61	0.62	*FD*	0.14	-0.12	*NO*	0.04	NA	*E1*	0.5	0.2	*E1*	0.36	0.3	*E1*	-0.14	-0.06
	Between legs	*Yes*	0.53	0.52	*Yes*	0.71	0.72											0.29	0.28
**Gazing**		*E1*	**0.35**	0.34	*FD*	0.16	0.11	*NO*	-0.35	-0.06				*E1*	0	0.24	*E1*	**0.48**	0.44
								E1+E2	-0.26	-0.08				*Bowl*	0.34	0.12	*Stool*	0.18	0.14
**Vocalization**	Barking	*E1*	0.85	0.85	*FD*	0.12	0.12												
**Displacement**	Nose-licking	*Yes*	0	-0.01	*Yes*	0.16	0.56	*Yes*	0.37	0.61	-			*Yes*	-0.2	-0.28	*Yes*	0.41	0.51
	Yawning	*Yes*	-0.03	-0.08	*Yes*	-0.06	0	*Yes*	0.48	0.45	-			*Yes*	-0.09	-0.1	*Yes*	-0.21	-0.2
	sniffing the ground	*Yes*	-0.04	-0.04	*Excl.*			*Excl.*			-			*Excl.*			*Excl.*		
**Physical contact**	Body contact	*E1*	0.55	0.55															
	Jumping	*E1*	0.5	**0.46**															
	Sniffing	*E1*			*FD*	0.03	0.19	*NO*	-0.04	0	-			*E1*	-0.09	-0.05			
**Reactions**	Flee	*E1*	-0.08	-0.16				*NO*	0.32	NA							*E1*	0.76	0.58
	Crouch																*E1*	-0.05	-0.02
	Friendly	*E1*	**0.45**	0.56	*FD*	-0.07	0.15							*E1*	0.34	0.35	*E1*	0.13	0.14
	Head dip	*E1*	-0.08	-0.09	*FD*	-0.21	0	*NO*	0	NA							*E1*	-0.07	-0.02
	Play	*E1*	-0.03	-0.06															
	Risk assesment				*FD*	-0.04	-0.04												
	Stand tall				*FD*	-0.12	-0.29												
**Subtest-specific variables**	Phase of first Approach	*E1*	**0.49**	0.51				*NO*	0.2	0	-			-			*Stool*	0.65	0.75
	Latency of first approach				*FD*	-0.15	0.2	*NO*	0.05	-0.9	-			-			*Stool*	-0.11	-0.08
	Genital sniffing				*FD*	0.15	-0.15												

	Observation of gesture: yes	-			-			-			*E1*	0.51	0.12	-			-		
	Observation of gesture: no	-			-			-			*E1*	**0.33**	-0.2	-			-		
	Successful choice	-			-			-			*Bowl*	0.64	0.38	-			-		
	Unsucessful choice	-			-			-			*Bowl*	0.55	0.06	-			-		
	No choice	-			-			-			*Bowl*	0.52	0.17	-			-		
**Gaze alternations**	2-way	-			-			-			-			*E1/ bowl*	0.03	-0.08	*E1/ stool*	0.14	0.07
	3-way	-			-			-			-			*E1/ bowl*	0.1	0.08	*E1/ stool*	**0.38**	**0.38**
**Average per subtest**			**0.32**	**0.32**		**0.06**	**0.13**		**0.12**	**0.10**		**0.51**	**0.12**		**0.16**	**0.13**		**0.22**	**0.23**
**Average across the test battery**			**0.23**	**0.17**															

For each variable and subtest, the modifier (M.) and the test-retest reliability is displayed for all tests (All) and for only the tests did not have any coded disturbances (w/D) respectively. The modifiers were the main experimenter (E1), the fake dog (FD), the novel object (NO), the bowl, or the stool. Test-retest reliabilities with a p-value below 0.05 were regarded as acceptable. Behaviours with non-significant test-retest reliability are indicated with a light-grey background and in bold if they were significant but regarded as poor reliability (i.e., below an ICC of 0.5). Variables that were excluded due to low occurrence (i.e., less than 10% of testing occasions) are not shown unless their exclusion only applied to certain subtests. In that case, they are left empty or marked with NA if it only applies to the analysis without disturbances. Variables marked with a minus (-) were not coded in the respective subtest. Detailed analysis results with the 95% confidence interval, F-value statistic, and p-value can be found in S5 Table.

Discussion

In this study, we presented what, to our knowledge, is the first attempt at testing free-ranging dogs in a behavioural test battery and report feasibility and reliability of the test measures. Testing the dogs in their natural environment was indeed feasible and the coding reliability was high, indicating that this (relative to previous studies) long and complex behaviour test battery can be conducted with street dogs. The range from good to poor test-retest reliability, on the other hand, reflects the challenges dog behaviour tests conducted with free-ranging animals on the streets are faced with and we provide possible explanations and further recommendations for the use of this test battery and behavioural testing of free-ranging dogs in general.

Although the subtests in this test battery were adapted from mostly enclosed, standardized pet and working dog behaviour assessments [e.g., 37, 41, 49][but see 22], bringing the test to the free-roaming dogs and standardizing the set up in the streets and beach environments of our populations turned out to be surprisingly manageable, and there were no terminations due to aggression towards people or equipment. More importantly, most dogs completed all six subtests and sample collections despite an average test duration of 30 minutes, suggesting that street dogs (at least those of this population) can remain motivated throughout longer and more complex procedures than those used so far [e.g., 20, 33, 34, 72].

Similarly, the concern that testing and filming in the variable street conditions would negatively impact the coding reliability was unfounded. Our results surpassed the target of a mean ICC of 0.7 with good average inter- and intra-rater reliability of 0.84 and 0.93 respectively. Contrary to our expectations, the coding reliability in this street test was thereby *on par* or even better than many pet and working dog studies [ICC 0.68 in meta-analysis by 9, ICC >0.9 in 41, ICC 0.77 in 65], suggesting that our chosen variables were not only well defined but could also be coded reliably despite the challenging street conditions. Similarly encouraging inter-rater results have been reported for a few other street dog tests as well [20, 22], further underlining that such results are achievable for street testing in general.

However, when looking at the reliability of the individual variables, a few did not meet the threshold. Notably, ‘medium proximity’, i.e., two body lengths distance from the dog to the experimenter or object of interest, was the only one that was low on both inter- and intra-rater reliability. With the constant adaptation of the camera angles to adjust for the variability of the street conditions, it might thus be best to stick to distances that are easier to identify such as the more reliable ‘close proximity’ (i.e., one body length distance). Additionally, ‘head dip’, ‘stand tall’, and ‘friendly’ all had poor agreement between the raters, which might be due to the complexity of these behaviours, with their definitions relying on a variety of subtle behaviours and body postures exhibited together (for the detailed definitions see S3 Table). While these definitions were adapted from previously used ethograms [17, 73, 74], the filming in street conditions likely made the subtle cues harder to detect or the behaviours might have been misinterpreted due to the difference from the natural conspecific setting they were initially defined in. Given the poor coding reliability of those traits, they should thus be dropped from the presented ethogram unless a more reliable definition or training method can be found and validated, and care should be taken in the establishment of future tests to balance between the complexity of target behaviours and filming feasibility in the streets.

While these outcomes so far paint a positive picture for street dog testing, the feasibility in terms of capturing the desired traits cannot be claimed without looking at the test’s ability to elicit the same behaviours consistently over time. The presented test battery resulted in an average test-retest reliability of 0.23, which, according to [63], is regarded as poor reliability. While the definition of personality traits is firmly bound to their consistency over time and across contexts [9], capturing such behavioural consistency has been an issue for many test batteries. A meta-analysis of 31 studies revealed an average trait consistency of 0.43 in pet, shelter and working dogs, which was considered significant although the studies within displayed highly variable results ranging from -0.73 to 1.00 [35]. We found a similarly high variability in the test-retest reliability of the behaviours we assessed, with subtest reliability varying between a moderate ICC of 0.51 for the pointing test and a poorly reliable 0.06 for the fake dog test. Capturing consistent personality or behavioural scores over time is hence an issue not unique to the testing of street dogs in their natural habitat.

Intriguingly, most of the variables that were reliable over time were human-directed behaviours, including proximity to and tail wagging at the experimenter, body contact, and time of first approach. Likewise, the subtests in which the experimenter constantly interacted with the dog (i.e., human approach, pointing, tractability test) had the most reliable variables and highest average test-retest reliability over time. This is in accordance with high reliability and strong heritability for sociability and biddability in pet dogs [e.g., 41, 49, 75] as well as dogs' performance during pointing tasks [76, 77] which, with the same consistency result in free-ranging dogs, might point to a genetic basis on which the selection process may have acted upon during dog domestication [3].

More important in terms of street dog testing, however, is that the discrepancy in reliability between these social tests and behaviours and the other subtests (fake dog, novel object, and begging test in particular) might be driven by their high interactivity, leaving the animal little time to be distracted. Free-ranging dogs have been shown to gaze at a person that does not attend to them directly less than at an attentive person while such a difference does not occur in pet dogs [33]. Moreover, the reality of testing in the free-ranging dogs' natural habitat means testing in an often highly chaotic environment, including frequent disturbances and environmental variations. While we tried to account for this by excluding all subtests with coded disturbances in a second analysis, this did not improve reliability. One reason might be that our choice of coding disturbances only when the dog was visible and attending to the distraction for several seconds underrepresented the influence of the disturbance on the study subject. Indeed, only six of the 35 dogs were alone in both tests (i.e., initial test and retest) while for all others, additional dogs entered the testing area throughout one or both testing occasions and had to be 'distracted away' by the second experimenter. Besides the probable influence of those disturbances on the overall retest reliability, they might explain why the subtests in which the dog had more time to focus on their environment without the direct engagement by the experimenter (fake dog, novel object, begging) showed a lower retest reliability. Whether behaviours like gazing and yawning and the entire begging test, which does not only lack interaction but also novelty, can reliably reflect a dog's behaviour under such circumstances is hence questionable and requires further rigorous testing in alternative test setups on the streets.

To address the disturbance problem on the streets, we propose four not mutually exclusive approaches: 1) Have at least one additional person on the team that is solely responsible to distract approaching dogs out of sight. 2) Allow for longer between-subtest-phases if the disturbance is temporary and let the dog return to an attentive state rather than pushing through the test. However, one should keep in mind that this changes the time dogs spend in the overall test procedure and carefully consider an influence on the respective goal. 3) If the distraction is substantial, terminating the test and either repeating or resuming the test on a different day might be an option, depending on the respective test set up, goal, and familiarity with the population. 4) Meticulously coding any distraction might allow for correction of the disturbances, or at least provide deeper understanding of their influence on the behaviour, based on which decisions for future testing can be made.

Even after considering the disturbance problem, the consistency of dogs' behaviour in the fake dog and novel object tests was notably low. This might seem surprising since both tests are regularly used in dog temperament assessments [e.g., 37, 66]. However, we could not find any reference to the retest reliability of fake dog tests, and studies exploring novel object tests and novelty-seeking and fearlessness traits in general showed that their test-retest reliability even in highly standardised settings is often poor (i.e., ICC<0.50) [e.g., Novelty-seeking: 0.48 in 41, Environmental Sureness: 0.16 in 65]. Accordingly, it has been proposed that test-retest outcomes are sensitive to the novelty of the object and situation due to learning effects [49]. Indeed, the fact that the free-ranging dogs explored the fake dog significantly more in the initial test while showing less fear in the retest supports the assumption that the animals partially remembered the initial encounter and adapted their behaviour accordingly. Although we made efforts to modify the appearance of the fake dog and novel object for the retest, the situation, procedure, and set-up remained the same and seem to have lacked the novelty needed to "fool" the animals twice within just six weeks. Nevertheless, it needs to be stressed that the street dogs frequently displayed appropriate conspecific, startling, and investigation behaviour in the initial presentation. Additionally considering that those subtests have been validated with fake vs. real dogs in case of the fake dog test [37, 47] and deemed reliable despite similar retest problems in case of the novel object [e.g., 41, 49], we suggest that these two subtests are still

suitable to test the street dogs. Particularly for the fake dog test however, the rather short interaction time most dogs showed with the fake dog suggests that a shorter exposure (i.e., test duration) or movement in the fake dog [e.g., 47] might have to be considered to decrease not only the distraction possibilities, but also the risk that neophobia and boldness traits replace the conspecific behaviours as soon as the dogs realize that the fake dog is not in fact real.

At the same time, these two subtests act as a reminder for the importance of appropriate between-test-intervals, particularly for test batteries investigating behavioural profiles. With free-ranging dogs, the balance between waiting out potential learning effects and retesting early enough to find a considerable number of test subjects again can be difficult, especially if the study population is not well-known. Turnover rates can be highly variable between locations and while we were familiar with the dynamics in the surrounding area [21], we had not yet worked with this particular population of street dogs. In addition, more than half of our study subjects were retested in a different location. The substantial retesting rate of 72% within an average of six weeks was hence surprising, though the considerable variation between the individuals (min. 33, max. 76 days) has to be acknowledged. Meticulous notes on appearance, exact finding location, and particular behaviours were crucial in this effort, not only for retesting but also to avoid testing of the same dog as a different individual. While working with a well-known population would streamline such efforts, our results show that it is not a prerequisite. In addition, using fewer memorable cues (e.g., the V-shaped wooden board in the Fake dog and Novel object subtest) might reduce learning effects and allow for a shorter between-test-interval with better outcomes than presented here.

Beside these two probable explanations for the low test-retest reliability in some subtests, an additional – though highly speculative – explanation might be that free-ranging dogs could be less genetically fixed in some behavioural traits than modern breed dogs and thus behave less consistently in general. This idea might not be too farfetched, considering that breed dogs have been selected for specific characteristics and have lost considerable genetic variation along the way [78], whereas free-ranging dogs are still subject to free mate choice and the instability of their environment [14]. Behavioural plasticity for certain traits (e.g., adaptiveness to the highly dynamic group compositions, weather conditions, or anthropogenic changes)

might therefore be a much more useful and thus widely retained trait in free-ranging dogs to deal with the constant changes in their environment, while it might have been selected against in our modern breeds. Indeed, the study of reaction norms in wild fish and birds has provided cautious evidence that behavioural plasticity (i.e., intra-individual variability) can have a heritable component [79, 80]. And though a meta-analysis targeting the question whether behaviour-selected working-breed dogs were more fixed in their behaviour than non-selected non-working breed dogs could not find any support for this hypothesis [35], it was reported that an unstable rearing environment does not seem to have an effect on the consistency of behaviour, further highlighting the stability of certain traits in breed dogs [76]. Exploring how this compares to free-ranging dogs is an exciting avenue for further research.

A last consideration regarding the test itself pertains to the exclusion of the play component in the Human approach test and what that teaches us about the need to consider your study subject in all its facets. Play is frequently used in temperament tests to assess sociability [36, 40, 46], and it has also been suggested as a potential part of the domestication syndrome in terms of neotenic behaviour [reviewed in 48, 81]. When applying this subtest with the street dogs, the low engagement rate and often fearful behaviour of the animal towards the toy might be explained by the lack of or negative experience such unowned free-ranging scavengers had with pulling on an object with a human [23]. In a similar incident, Lazzaroni, Range (20) found that free-ranging dogs' lower persistence in comparison to pet dogs was likely due to their need to preserve energy and a lack of experience with human-manipulated items. Alternatively, the recent findings that both ancient breeds [82] and wolf hybrids [83] also express lower levels of human-directed play behaviour than most modern breeds might suggest that a selection for human-directed play has only relatively recently taken place in modern breed dogs (e.g. as reward use in working dogs [84] or bonding-experience between dogs and humans [81]). In addition, our anecdotal observation with free-ranging populations indicates multiple incidents of conspecific play, running after birds or balls seemingly for fun, and 'playing' with the bowl during the pointing test. Whether play does indeed persist into adulthood in free-ranging dogs in the conspecific context or solitary play, but not with a human partner, or whether our choice of method was just ill-considered, remains to explored and emphasizes how carefully the subject

population, its ecology and life experience must be considered despite our extensive experience with the species itself.

Finally, there are two important limitations that need to be addressed in terms of free-ranging dog testing as well as the representativeness of this study. First, the consideration that testing adult free-ranging dogs must rely on voluntary participation creates a natural bias for more social animals, especially in longer and more interactive tests, and might be particularly challenging for feral populations (i.e., not reliant on human settlements). This bias is somewhat reduced in our Human approach test since the only selection criteria for starting a test was that the dog was seemingly alone. Thus at least the first steps of the test consisting of calling the dog and attempting a friendly approach were carried out with all dogs we found alone. If a dog then fearfully ran away as soon as we attempted the approach, this was analysed as part of our dataset. Although a minority, these dogs are part of our population and the fact that we were able to retest more than half of these ‘escaping’ individuals and they manifested the same behaviour on second testing provides evidence that this measurement was indeed reliable. Nevertheless, it is the case that overly fearful animals were lost for all the following subtests and are therefore not part of the majority of the reliability analysis. Similarly, [22] found in a testing effort with 160 street dogs that half of their testing subjects could be classified as fearful and that these dogs were significantly less likely to approach and choose in a pointing task than neutral and affiliative dogs. At the same time, any constraining of a free-ranging animal during a test would undoubtedly influence its behaviour to a point where it cannot be regarded as natural anymore. Explorable options to test such dogs could be the use of shorter tests with little human presence wherever possible (e.g., reaction- or exploration-based tests) or testing populations that now live in a fenced shelter but genetically belong to the clade of free-ranging dogs and had spent most of their lives roaming freely. However, with the latter option, whether the influence of life experience and current social contexts might have an influence on the target behaviour has to be strongly considered [19]. Secondly, the fact that our study population has previously been recognized as exceptionally friendly [21] kept us from being able to analyse the reliability of aggressive behaviours and stress-related behaviours. For example, only 16.7% of our sample ran away during the sociability test and none showed aggression, while more than 50% of an

Ethiopian village dog sample fled upon approach and 11% reacted aggressively (though one needs to consider that these dogs were not always tested alone or outside [34]). While expanding this test battery to other populations might be a way to start to characterize different free-ranging dog populations and what may affect differences in their behavioural profiles, we acknowledge that these limitations are likely difficult to be overcome.

Exhibition of extreme fear or the slightest sign of human-directed aggression will necessarily lead to aborting the test (particularly considering the risk of lethal disease, [e.g., 18]. And while using a fake dog rather than a real dog was indeed an attempt to test for the presence of aggression in a safe way, collecting complementary observational data (regarding both human and conspecific directed behaviour) of the same individuals may be a complementary method allowing for multiple measures of specific behavioural traits.

In conclusion, the easy implementation, successful participation of most free-ranging dogs, and the high coding reliability presented here confirm that free-ranging dogs can be tested in longer test batteries with complex ethograms despite the challenging street conditions. The wide variability in the test-retest reliability across subtests demonstrate that the management of disturbances, the choice of set up and test-retest intervals, and careful considerations of the study subjects' life experience and ecology are central challenges behavioural tests with free-ranging dogs in their natural habitat face. At the same time, considerable reliability in some of the subtest demonstrates that under well-chosen conditions, testing street dogs is possible and opens a vast array of intriguing pathways to explore [19]. Bearing suggestions made in the discussion in mind, going forward the test battery presented here can be a valuable method to test questions about cognitive traits, behavioural profiles, and domestication in such an understudied population as free-ranging dogs.

Acknowledgements

We thank Professor Ikhlass El Berbri for her support and collaboration in this project. Additionally, we thank the rest of our team in Morocco (in particular Andreas Berghänel, Haytem Bouchri, Manon Delaunay and Magdalena Juskaite) without whom the project would not have been possible. We are also grateful to

the population and authorities in the Sous-Massa region for supporting our field work. Finally, we want to extend our gratitude to the two reviewers for their valuable feedback and suggestions.

Bibliography

1. Trut LN. Early Canid Domestication: The Farm-Fox Experiment: Foxes bred for tamability in a 40-year experiment exhibit remarkable transformations that suggest an interplay between behavioral genetics and development. American Scientist. 1999;87(2):160-9.
2. Hare B, Tomasello M. Human-like social skills in dogs? Trends in cognitive sciences. 2005;9(9):439-44. doi: https://doi.org/10.1016/j.tics.2005.07.003.
3. von Holdt BM, Shuldiner E, Koch IJ, Kartzinel RY, Hogan A, Brubaker L, et al. Structural variants in genes associated with human Williams-Beuren syndrome underlie stereotypical hypersociability in domestic dogs. Science advances. 2017;3(7):e1700398. doi: https://doi.org/10.1126/sciadv.1700398.
4. Range F, Marshall-Pescini S, Kratz C, Virányi Z. Wolves lead and dogs follow, but they both cooperate with humans. Scientific reports. 2019;9(1):1-10. doi: https://doi.org/10.1038/s41598-019-40468-y.
5. Darwin C. The descent of Man; and on Selection in relation to Sex: J. Murray; 1871.
6. Pavlov IP. The scientific investigation of the psychical faculties or processes in the higher animals. Science. 1906;24(620):613-9.
7. Udell MA, Wynne CD. A review of domestic dogs'(Canis familiaris) human-like behaviors: or why behavior analysts should stop worrying and love their dogs. Journal of the experimental analysis of behavior. 2008;89(2):247-61.
8. Bensky MK, Gosling SD, Sinn DL. The world from a dog's point of view: a review and synthesis of dog cognition research. Advances in the Study of Behavior. 2013;45:209-406.
9. Gartner MC. Pet personality: A review. Personality and Individual Differences. 2015;75:102-13. doi: https://doi.org/10.1016/j.paid.2014.10.042.
10. Creevy KE, Akey JM, Kaeberlein M, Promislow DE. An open science study of ageing in companion dogs. Nature. 2022;602(7895):51-7.
11. Range F, Marshall-Pescini S. Domestication Hypotheses Relating to Behaviour and Cognition: Which Are Supported by the Current Data? Wolves and Dogs: Springer; 2022. p. 335-73.
12. Hughes J, Macdonald DW. A review of the interactions between free-roaming domestic dogs and wildlife. Biological conservation. 2013;157:341-51.
13. Lord K, Feinstein M, Smith B, Coppinger R. Variation in reproductive traits of members of the genus Canis with special attention to the domestic dog (Canis familiaris). Behavioural processes. 2013;92:131-42.
14. Pilot M, Malewski T, Moura AE, Grzybowski T, Oleński K, Ruść A, et al. On the origin of mongrels: evolutionary history of free-breeding dogs in Eurasia. Proceedings of the Royal Society B: Biological Sciences. 2015;282(1820):20152189. doi: https://doi.org/10.1098/rspb.2015.2189.
15. Bonanni R, Cafazzo S. The social organisation of a population of free-ranging dogs in a suburban area of Rome: a reassessment of the effects of domestication on dogs' behaviour. The social dog: Elsevier; 2014. p. 65-104.
16. Cafazzo S, Valsecchi P, Bonanni R, Natoli E. Dominance in relation to age, sex, and competitive contexts in a group of free-ranging domestic dogs. Behavioral Ecology. 2010;21(3):443-55. doi: https://doi.org/10.1093/beheco/arq001.
17. Range F, Marshall-Pescini S. Wolves and Dogs: Between Myth and Science: Springer Nature; 2022.
18. Hampson K, Coudeville L, Lembo T, Sambo M, Kieffer A, Attlan M, et al. Estimating the global burden of endemic canine rabies. PLoS neglected tropical diseases. 2015;9(4):e0003709.

19. Horn L, Cimarelli G, Boucherie PH, Šlipogor V, Bugnyar T. Beyond the dichotomy between field and lab—the importance of studying cognition in context. Current Opinion in Behavioral Sciences. 2022;46:101172.
20. Lazzaroni M, Range F, Bernasconi L, Darc L, Holtsch M, Massimei R, et al. The role of life experience in affecting persistence: A comparative study between free-ranging dogs, pet dogs and captive pack dogs. PLOS ONE. 2019;14(4):e0214806. doi: https://doi.org/10.1371/journal.pone.0214806.
21. Lazzaroni M, Marshall-Pescini S. Der Streuner- das fehlende Bindeglied. In: Kitchenham K, editor. Streuner Hunde: Von Moskaus U-Bahn-Hunden bis Indiens Underdogs: Komsos; 2020.
22. Bhattacharjee D, Mandal S, Shit P, Varghese MG, Vishnoi A, Bhadra A. Free-ranging dogs are capable of utilizing complex human pointing cues. Frontiers in Psychology. 2020;10:2818. doi: https://doi.org/10.3389/fpsyg.2019.02818.
23. Paul MM, Sreejani Sen; Sau, Shubhra; Nandi, Anjan K.; Bhadra, Anindita. High early life mortality in free-ranging dogs is largely influenced by humans. Sci Rep. 2016;6(19641). doi: https://doi.org/10.1038/srep19641.
24. Hansen Wheat C, Fitzpatrick JL, Rogell B, Temrin H. Behavioural correlations of the domestication syndrome are decoupled in modern dog breeds. Nature communications. 2019;10(1):1-9. doi: https://doi.org/10.1038/s41467-019-10426-3.
25. Hansen Wheat C, van der Bijl W, Wheat CW. Morphology does not covary with predicted behavioral correlations of the domestication syndrome in dogs. Evolution letters. 2020;4(3):189-99. doi: https://doi.org/10.1002/evl3.168.
26. Larson G, Fuller DQ. The evolution of animal domestication. Annual Review of Ecology, Evolution, and Systematics. 2014;45:115-36. doi: https://doi.org/10.1146/annurev-ecolsys-110512-135813.
27. Sánchez-Villagra MR, Geiger M, Schneider RA. The taming of the neural crest: a developmental perspective on the origins of morphological covariation in domesticated mammals. Royal Society open science. 2016;3(6):160107. doi: https://doi.org/10.1098/rsos.160107.
28. Boyko AR, Boyko RH, Boyko CM, Parker HG, Castelhano M, Corey L, et al. Complex population structure in African village dogs and its implications for inferring dog domestication history. Proceedings of the National Academy of Sciences. 2009;106(33):13903-8.
29. Vanak AT, Gompper ME. Dogs Canis familiaris as carnivores: their role and function in intraguild competition. Mammal review. 2009;39(4):265-83. doi: https://doi.org/10.1111/j.1365-2907.2009.00148.x.
30. Range F, Marshall-Pescini S. Comparing wolves and dogs: current status and implications for human 'self-domestication'. Trends in Cognitive Sciences. 2022. doi: https://doi.org/10.1016/j.tics.2022.01.003.
31. Kato M, Yamamoto H, Inukai Y, Kira S. Survey of the stray dog population and the health education program on the prevention of dog bites and dog-acquired infections: a comparative study in Nepal and Okayama Prefecture, Japan. Acta Medica Okayama. 2003;57(5):261-6.
32. Bhattacharjee D, Sau S, Das J, Bhadra A. Free-ranging dogs prefer petting over food in repeated interactions with unfamiliar humans. Journal of Experimental Biology. 2017;220(24):4654-60. doi: https://doi.org/10.1242/jeb.166371.
33. Brubaker L, Dasgupta S, Bhattacharjee D, Bhadra A, Udell MA. Differences in problem-solving between canid populations: Do domestication and lifetime experience affect persistence? Animal Cognition. 2017;20(4):717-23. doi: https://doi.org/10.1007/s10071-017-1093-7.
34. Ortolani A, Vernooij H, Coppinger R. Ethiopian village dogs: Behavioural responses to a stranger's approach. Applied Animal Behaviour Science. 2009;119(3-4):210-8.
35. Fratkin JL, Sinn DL, Patall EA, Gosling SD. Personality consistency in dogs: a meta-analysis. PloS one. 2013;8(1):e54907. doi: https://doi.org/10.1371/journal.pone.0054907.

36. Valsecchi P, Barnard S, Stefanini C, Normando S. Temperament test for re-homed dogs validated through direct behavioral observation in shelter and home environment. Journal of Veterinary Behavior. 2011;6(3):161-77. doi: https://doi.org/10.1016/j.jveb.2011.01.002.
37. Barnard S, Kennedy D, Watson R, Valsecchi P, Arnott G. Revisiting a previously validated temperament test in shelter dogs, including an examination of the use of fake model dogs to assess conspecific sociability. Animals. 2019;9(10):835. doi: https://doi.org/10.3390/ani9100835.
38. Heberlein MT, Turner DC, Range F, Virányi Z. A comparison between wolves, Canis lupus, and dogs, Canis familiaris, in showing behaviour towards humans. Animal Behaviour. 2016;122:59-66. doi: https://doi.org/10.1016/j.anbehav.2016.09.023.
39. Mendes JWW, Resende B, Savalli C. A review of the unsolvable task in dog communication and cognition: comparing different methodologies. Animal Cognition. 2021;24(5):907-22. doi: https://doi.org/10.1007/s10071-021-01501-8.
40. Svartberg K. The hierarchical structure of dog personality in a new behavioural assessment: A validation approach. Applied Animal Behaviour Science. 2021;238:105302. doi: https://doi.org/10.1016/j.applanim.2021.105302.
41. Turcsán B, Wallis L, Virányi Z, Range F, Müller CA, Huber L, et al. Personality traits in companion dogs—Results from the VIDOPET. PLoS One. 2018;13(4):e0195448. doi: https://doi.org/10.1371/journal.pone.0195448.
42. Barnard S, Marshall-Pescini S, Passalacqua C, Beghelli V, Capra A, Normando S, et al. Does Subjective Rating Reflect Behavioural Coding? Personality in 2 Month-Old Dog Puppies: An Open-Field Test and Adjective-Based Questionnaire. PLOS ONE. 2016;11(3):e0149831. doi: https://doi.org/10.1371/journal.pone.0149831.
43. Brady K, Hewison L, Wright H, Zulch H, Cracknell N, Mills D. A spatial discounting test to assess impulsivity in dogs. Applied Animal Behaviour Science. 2018;202:77-84. doi: https://doi.org/10.1016/j.applanim.2018.01.003.
44. Plutchik R. Individual and breed differences in approach and withdrawal in dogs. Behaviour. 1971;40(3-4):302-11.
45. Wright HF, Mills DS, Pollux PM. Behavioural and physiological correlates of impulsivity in the domestic dog (Canis familiaris). Physiology & behavior. 2012;105(3):676-82. doi: https://doi.org/10.1016/j.physbeh.2011.09.019.
46. Svartberg K, Forkman B. Personality traits in the domestic dog (Canis familiaris). Applied animal behaviour science. 2002;79(2):133-55. doi: https://doi.org/10.1016/S0168-1591(02)00121-1.
47. Reid PJ, Cussen VA, Collins LK, Lockwood R. The Utility of Model Dogs for Assessing Conspecific Aggression in Fighting Dogs. Applied Animal Behaviour Science. 2022:105710. doi: https://doi.org/10.1016/j.applanim.2022.105710.
48. Kaiser S, Hennessy MB, Sachser N. Domestication affects the structure, development and stability of biobehavioural profiles. Frontiers in Zoology. 2015;12(1):1-11. doi: https://doi.org/10.1186/1742-9994-12-S1-S19.
49. Svartberg K, Tapper I, Temrin H, Radesäter T, Thorman S. Consistency of personality traits in dogs. Animal Behaviour. 2005;69(2):283-91. doi: https://doi.org/10.1016/j.anbehav.2004.04.011.
50. Krause MA, Udell MA, Leavens DA, Skopos L. Animal pointing: Changing trends and findings from 30 years of research. Journal of Comparative Psychology. 2018;132(3):326. doi: https://doi.org/10.1037/com0000125.
51. Mendes JWW, Resende B, Savalli C. A review of the unsolvable task in dog communication and cognition: comparing different methodologies. Animal Cognition. 2021:1-16. doi: https://doi.org/10.1007/s10071-021-01501-8.
52. Marshall-Pescini S, Rao A, Virányi Z, Range F. The role of domestication and experience in 'looking back'towards humans in an unsolvable task. Scientific reports. 2017;7(1):46636.

53. Lazzaroni M, Marshall-Pescini S, Manzenreiter H, Gosch S, Přibilová L, Darc L, et al. Why do dogs look back at the human in an impossible task? Looking back behaviour may be over-interpreted. Animal Cognition. 2020;23(3):427-41.
54. Miklósi Á, Kubinyi E, Topál J, Gácsi M, Virányi Z, Csányi V. A simple reason for a big difference: wolves do not look back at humans, but dogs do. Current biology. 2003;13(9):763-6. doi: https://doi.org/10.1016/S0960-9822(03)00263-X.
55. D'Aniello B, Scandurra A. Ontogenetic effects on gazing behaviour: a case study of kennel dogs (Labrador Retrievers) in the impossible task paradigm. Animal cognition. 2016;19:565-70.
56. Bonanni R, Cafazzo S, Valsecchi P, Natoli E. Effect of affiliative and agonistic relationships on leadership behaviour in free-ranging dogs. Animal Behaviour. 2010;79(5):981-91. doi: https://doi.org/10.1016/j.anbehav.2010.02.021.
57. Pompey KT. A Unified Approach to Estimating the Intraclass Correlation Coefficient and Its Bias: an Exploratory Study: University of South Carolina; 2021.
58. Boström AF, Hyytiäinen HK, Koho P, Cizinauskas S, Hielm-Björkman AK. Development of the Finnish neurological function testing battery for dogs and its intra-and inter-rater reliability. Acta Veterinaria Scandinavica. 2018;60(1):1-9. doi: https://doi.org/10.1186/s13028-018-0408-2.
59. Team RDC. R: A language and environment for statistical computing. (No Title). 2010.
60. Shrout PE, Fleiss JL. Intraclass correlations: uses in assessing rater reliability. Psychological bulletin. 1979;86(2):420. doi: https://doi.org/10.1037/0033-2909.86.2.420.
61. Harvey ND. A Simple Guide to Inter-rater, Intra-rater and Test-retest Reliability for Animal Behaviour Studies. 2021. doi: https://doi.org/10.31219/osf.io/8stpy.
62. Harvey ND, Craigon PJ, Sommerville R, McMillan C, Green M, England GC, et al. Test-retest reliability and predictive validity of a juvenile guide dog behavior test. Journal of Veterinary Behavior. 2016;11:65-76. doi: https://doi.org/10.1016/j.jveb.2015.09.005.
63. Koo TK, Li MY. A guideline of selecting and reporting intraclass correlation coefficients for reliability research. Journal of chiropractic medicine. 2016;15(2):155-63. doi: https://doi.org/10.1016/j.jcm.2016.02.012.
64. Patronek GJ, Bradley J, Arps E. What is the evidence for reliability and validity of behavior evaluations for shelter dogs? A prequel to "No better than flipping a coin". Journal of Veterinary Behavior. 2019;31:43-58. doi: https://doi.org/10.1016/j.jveb.2019.03.001.
65. Sinn DL, Gosling SD, Hilliard S. Personality and performance in military working dogs: Reliability and predictive validity of behavioral tests. Applied Animal Behaviour Science. 2010;127(1-2):51-65. doi: https://doi.org/10.1016/j.applanim.2010.08.007.
66. van der Borg JA, Beerda B, Ooms M, de Souza AS, van Hagen M, Kemp B. Evaluation of behaviour testing for human directed aggression in dogs. Applied Animal Behaviour Science. 2010;128(1-4):78-90. doi: https://doi.org/10.1016/j.applanim.2010.09.016.
67. Fleiss JL, Cohen J. The equivalence of weighted kappa and the intraclass correlation coefficient as measures of reliability. Educational and psychological measurement. 1973;33(3):613-9. doi: https://doi.org/10.1177/001316447303300309.
68. Moretti L, Hentrup M, Kotrschal K, Range F. The influence of relationships on neophobia and exploration in wolves and dogs. Animal Behaviour. 2015;107:159-73. doi: https://doi.org/10.1016/j.anbehav.2015.06.008.
69. Klausz B, Kis A, Persa E, Miklósi Á, Gácsi M. A quick assessment tool for human-directed aggression in pet dogs. Aggressive behavior. 2014;40(2):178-88. doi: https://doi.org/10.1002/ab.21501.
70. Beerda B, Schilder MB, van Hooff JA, de Vries HW. Manifestations of chronic and acute stress in dogs. Applied Animal Behaviour Science. 1997;52(3-4):307-19.
71. Pedretti G, Canori C, Biffi E, Marshall-Pescini S, Valsecchi P. Appeasement function of displacement behaviours? Dogs' behavioural displays exhibited towards threatening and neutral humans. Animal Cognition. 2023:1-10. doi: https://doi.org/10.1007/s10071-023-01742-9.

72. Lazzaroni M, Range F, Backes J, Portele K, Scheck K, Marshall-Pescini S. The Effect of Domestication and Experience on the Social Interaction of Dogs and Wolves With a Human Companion. Frontiers in Psychology. 2020;11. doi: https://doi.org/10.3389/fpsyg.2020.00785.
73. Cafazzo S, Marshall-Pescini S, Lazzaroni M, Virányi Z, Range F. The effect of domestication on post-conflict management: Wolves reconcile while dogs avoid each other. Royal Society Open Science. 2018;5(7):171553. doi: https://doi.org/10.1098/rsos.171553.
74. Cimarelli G, Marshall-Pescini S, Range F, Berghänel A, Virányi Z. Relationship quality affects social stress buffering in dogs and wolves. Animal Behaviour. 2021;178:127-40. doi: https://doi.org/10.1016/j.anbehav.2021.06.008.
75. Morrill K, Hekman J, Li X, McClure J, Logan B, Goodman L, et al. Ancestry-inclusive dog genomics challenges popular breed stereotypes. Science. 2022;17(6592):e0274383. doi: https://doi.org/10.1126/science.abk0639.
76. Gácsi M, Kara E, Belényi B, Topál J, Miklósi Á. The effect of development and individual differences in pointing comprehension of dogs. Animal cognition. 2009;12(3):471-9. doi: https://doi.org/10.1007/s10071-008-0208-6.
77. Bray EE, Gnanadesikan GE, Horschler DJ, Levy KM, Kennedy BS, Famula TR, et al. Early-emerging and highly heritable sensitivity to human communication in dogs. Current Biology. 2021;31(14):3132-6. e5. doi: https://doi.org/10.1016/j.cub.2021.04.055.
78. Gray MM, Granka JM, Bustamante CD, Sutter NB, Boyko AR, Zhu L, et al. Linkage disequilibrium and demographic history of wild and domestic canids. Genetics. 2009;181(4):1493-505. doi: https://doi.org/10.1534/genetics.108.098830.
79. Dingemanse NJ, Barber I, Wright J, Brommer J. Quantitative genetics of behavioural reaction norms: genetic correlations between personality and behavioural plasticity vary across stickleback populations. Journal of evolutionary biology. 2012;25(3):485-96. doi: https://doi.org/10.1111/j.1420-9101.2011.02439.x.
80. Araya-Ajoy YG, Dingemanse NJ. Repeatability, heritability, and age-dependence of seasonal plasticity in aggressiveness in a wild passerine bird. Journal of Animal Ecology. 2017;86(2):227-38. doi: https://doi.org/10.1111/1365-2656.12621.
81. Bradshaw JW, Pullen AJ, Rooney NJ. Why do adult dogs ‘play’? Behavioural processes. 2015;110:82-7. doi: https://doi.org/10.1016/j.beproc.2014.09.023.
82. Kolm N, Temrin H, Miklósi Á, Kubinyi E, Garamszegi LZ. The link between selection for function and human-directed play behaviour in dogs. Biology letters. 2020;16(9):20200366. doi: https://doi.org/10.1098/rsbl.2020.0366.
83. Hansen Wheat C, Fitzpatrick J, Tapper I, Temrin H. Wolf (Canis lupus) hybrids highlight the importance of human-directed play behavior during domestication of dogs (Canis familiaris). Journal of Comparative Psychology. 2018;132(4):373. doi: https://doi.org/10.1037/com0000119.
84. Brownell D. The Brownell-Marsolais scale: a proposal for the qualitative evaluation of SAR/disaster K9 candidates. Adv Rescue Technol. 2002;5:57-67.

Supplementary Information

S1 Exemplary video showing the different subtests of the test battery

S2 Equipment information

S3 Table. Detailed Ethogram

S4 Table. Detailed inter- and intra-rater reliability results

S5 Table. Detailed test-retest reliability outcome

Fig1

Click here to access/download;Figure;Fig1.tif

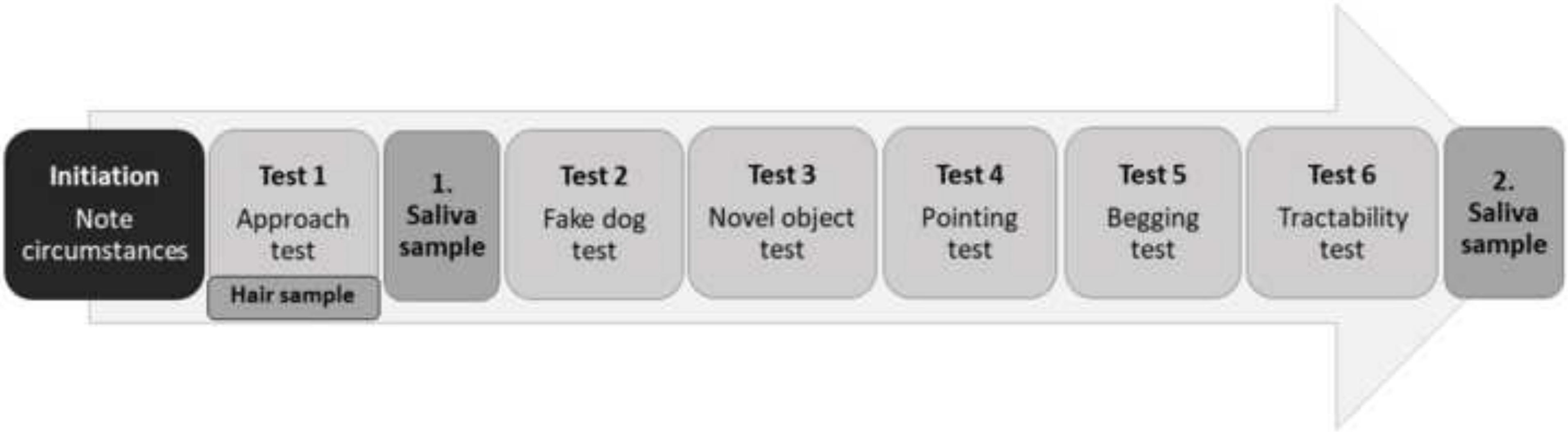

Fig2

Click here to access/download;Figure;Fig2.tif

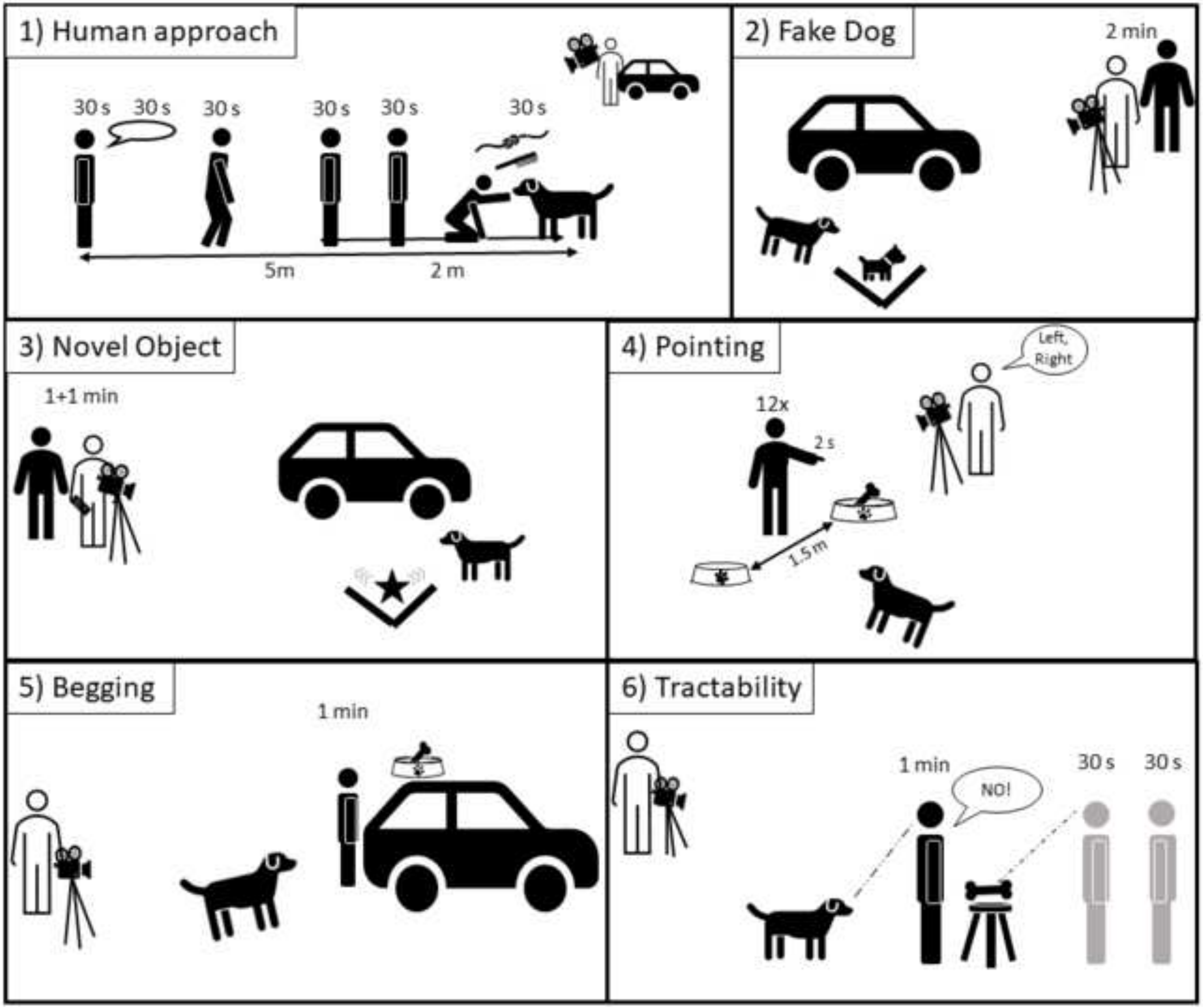

Click here to access/download
Supporting Information - Compressed/ZIP File Archive
Supplementary material.zip

Click here to access/download
Supporting Information - Compressed/ZIP File Archive
DSAdult22_PlosOne_Data.zip

~~Tackling the lack of behavioural evidence for the domestication syndrome – Feasibility and reliability of a novel Domestication Syndrome test battery with free-ranging dogs~~

Street-wise dog testing: feasibility and reliability of a behavioural test battery for free-ranging dogs in their natural habitat

Svenja Capitain[1*], Giulia Cimarelli[2], Urša Blenkuš[2], Friederike Range[2], Sarah Marshall-Pescini[2*]

[1] Department of Physics, Chemistry and Biology (IFM), Linköping University, Linköping, Sweden

[2] Domestication Lab, Konrad Lorenz Institute of Ethology, Department of Interdisciplinary Life Sciences, University of Veterinary Medicine Vienna, Vienna, Austria

* Corresponding author

Svenja.capitain@vetmeduni.ac.at (SC)

Sarah.marshall@vetmeduni.ac.at (SMP)

(Old) Abstract

While the domestication syndrome (DS) has long been the central explanation for the emergence of convergent but unelated traits in domesticated animals, the actual evidence is mixed. Recent studies suggest that the DS-related traits have become decoupled in modern breeds due to the directed breed selection for specific morphologies and behaviours. Free-ranging dogs might hence represent a better domestication model as they pre-date modern breed selection genetically and live a lifestyle more similar to the initial domesticates. Hence, here we present and assess the feasibility and reliability of a novel Domestication Syndrome test battery that targets the DS associated behavioural and physiological traits of free-ranging dogs (*Canis familiaris*), including human- and conspecific-directed sociability, neophobia, and various aspects of dog-human interaction. The test battery, consisting of six subtests, proved feasible with most dogs participating in the full test and an overall good inter- and intra-rater reliability (0.84 and 0.93 respectively), providing evidence that even in highly variable street conditions, complex behaviour tests can be conducted. Retesting the dogs after six weeks revealed that the frequent human-directed interactions were consistent over time while more disturbance-prone behaviours (for example human-directed gazing behaviour) had a low test-retest reliability. Though the subtests related to exploration of presented stimuli (novel object, fake dog) failed to create the same behavioural reactions at second presentation, these subtests elicited the expected target behaviours in the initial testing and are therefore argued to be suitable to test the target traits. If the less disturbance-prone variables are focused on going forward and disturbances are managed well, the Domestication Syndrome test battery can be a valuable tool in testing the Domestication Syndrome hypothesis.

New Abstract

Behavioural scientists are increasingly recognizing the need to conduct experiments in the wild to achieve a comprehensive understanding of their species' behaviour. For domestic dogs (*Canis familiaris*), such progress is lagging behind. While the life in human households is often regarded as dogs' natural habitat,

this classification disregards most of the global dog population. The value of including free-ranging dogs as study subjects is increasingly being recognized, but no comprehensive behavioural and cognitive test batteries have been conducted so far and their feasibility and reliability are unknown. This study is the starting point to fill this gap by pioneering and validating an elaborate behavioural test battery with street-living dogs. Therein, six common temperament tests (human-/conspecific-directed sociability, neophobia, tractability) and dog-human communication paradigms (pointing, inaccessible object) were adapted to the street conditions. We evaluated the feasibility of the test battery, the coding reliability of the measures, and investigated their temporal consistency in a retest of the same individuals six weeks later (test-retest reliability). The test battery proved feasible with most dogs participating in ~~the full test~~all subtests and ~~an overall good~~ it showed satisfactory inter- and intra-rater reliability (0.84 and 0.93 respectively), providing evidence that ~~even in highly variable street conditions,~~ complex behaviour tests can be conducted even in highly variable street conditions. Retesting revealed that some behaviours could be captured reliably across time, especially when the subtest was particularly engaging (e.g., human approach, point following). In contrast, the low retest reliability for subtests relying on sustained novelty and behaviours that were highly susceptible to disturbances (e.g., gazing) reflects the difficulties of street dog testing, including standardisation in disturbance-prone environments, ecology-dependent adaptation of methods, and intrinsic differences between pet and free-ranging dogs. Bearing the suggestions made in the discussion in mind, this test battery can be valuable in investigating cognition, behavioural profiles, and domestication in such an understudied population as free-ranging dogs.

(Old) Introduction

~~While domestication, the human-led selection of animals and plants to a life under human control, has been a part of human history for thousands of years [1], many of its mechanisms are still not fully understood. One aspect pertains to the so called ‘Domestication Syndrome’ (DS), first described in mammals by Darwin [2] as a set of inextricably linked morphological, behavioural, and physiological traits that differentiate domesticated species from their wild type counterparts. According to this idea, domesticated species are~~

more sociable and playful than their wild counterparts, less aggressive and explorative, show reduced stress responses, more coat depigmentation, and changes in their craniofacial structure, to name a few [3-5]. Since most of these traits have likely never been directly selected for, their convergence across species has led to the suggestion that a common selection factor was the driver for the emergence of these phenotypic changes [2]. Several studies have since pointed to the selection for tameness, i.e. reduced fear of humans as the underlying mechanism [5, 6] [7, 8], with a potential selection pathway through the neural crest cells [9].

However, as recent reviews reveal, empirical evidence for this idea remains scarce: not only do many of the domesticated species lack the majority of the expected traits [10, 11], but many of the observed traits were shown not to be consistent in their direction of change [e.g., skull morphology: 12, brain size: 13]. Even in dogs (*Canis familiaris*), the longest domesticated species [14] which is thus often regarded as the prime example for the DS, evidence remains inconclusive. While dogs show all morphological DS associated traits, some physiological measures were shown to reflect the opposite of the proposed changes depending on the situation [e.g., higher cortisol levels in dogs, 15], and the behavioural traits did not follow the expected correlations either [16]. At the same time, comparative research on dogs and wolves has given rise to alternative domestication hypotheses for driving selection factors instead of or in addition to tameness. Particularly hyper-sociability [17], increased deferential behaviour (i.e., tractability, otherwise known as higher sensitivity towards social inhibition [18]), and advanced dog-human communication skills [19, reviewed by 20] have been discussed as some of the main characteristics that differentiate canine domesticates from their wild ancestors.

This recent rise in criticism emphasises that particularly around the behavioural traits supposedly linked in the Domestication Syndrome, evidence for their more frequent presence in domesticates vs. wild-type and their co-variance in domesticates is lacking [10, 11]. According to the DS, domesticated species are tamer, more sociable and less aggressive (opinions diverge whether that is towards humans and/or conspecifics [reviewed in 20]), more playful, less explorative/novelty seeking, and less fearful than their wild counterparts [3-5]. To assess these traits in dogs and beyond, 'personality' or 'temperament' test batteries are used in which the animals are exposed to a range of stimuli to elicit the target reaction. However, studies

investigating the DS-related personality traits in dogs so far either only focused on one/ a few traits at a time, thereby not allowing for covariance between traits to be investigated [e.g. novelty-seeking/ exploration in 21, human-directed play in 22]. Or the studies were not conceptualized to test the DS-traits in themselves but rather used existing data from personality tests in kennel clubs to investigate co-variance of DS-related traits [e.g., the Swedish Kennel Club database in 16, 23]. It is thus possible that both these approaches might have been unfit to capture the potential co-variance between DS-related behavioural traits. To address this, we developed a novel Domestication Syndrome test battery that is designed to test all the behavioural traits associated with the DS directly in dogs (and potentially human-socialized captive wolves for the needed comparisons).

Another potential limitation of the current studies investigating the co-variance of DS traits, is their reliance on pet/breed dogs. The majority of our working and pet dogs are not just the result of domestication but additionally underwent modern breed selection for specific functions and morphologies during the last few centuries, leading to a highly variable species with a reduced genome [24, 25]. Interestingly, the negative correlation between human-directed sociability and aggressive traits that would be expected in domesticated animals according to the DS only emerges in ancient but not modern dog breeds [23]. It was thus suggested that the DS-traits and their underlying genes might have become uncoupled during the targeted selection for specific traits in modern dog breeds [23, 25]. Considering these results, free-ranging dogs might be a better dog population to reconstruct the state of dog domestication. Contrary to the general assumption, the majority of free-ranging dogs are not a mixture of breeds but rather genetically distinct populations that predate the recent artificial selection and thus formation of breeds [26]. They would therefore be an ideal candidate population in which to test the DS- and other dog domestication-traits.

Paradoxically, this potential of free-ranging dogs has so far remained unexplored, and little is known about the feasibility of testing freely roaming dogs in their natural environment. While shorter tests on free-ranging dogs' cognitive abilities and socio-ecology have indeed been performed successfully [e.g., 27, 28, 29], to our knowledge, no long and complex personality test such as those that would be needed for the investigation of the behavioural DS traits has been implemented so far. Validity and reliability measures

that are pivotal when creating a novel personality test are thus unknown under the variable street conditions which are far from the standardized, secluded testing sessions generally employed for pet, shelter and working dog tests.

Validity of such tests with owned or shelter dogs is generally assessed through a third knowledgeable party or tool that can verify the correctness of results (e.g., assessing consistency between behaviour tests and owner/shelter worker questionnaire) [for review see 30]. However, this method is not applicable in such unowned, free-roaming animals. Thus, the alternative is a rigorous assessment of the test battery including a number of reliability aspects. First, the coding reliability which describes how well a rater agrees with themselves over time ("intra-rater reliability") and with others (i.e., "inter-rater reliability") about the behaviours observed (typically on video). This measure gives an indication whether the ethogram is well defined and whether the target behaviours can be coded reliably in the given testing conditions. For the behavioural variables to be of use in the trait analysis, this agreement should be substantial with acceptable intraclass correlation of 0.7 or above [31, 32]. Second, the test's ability to capture the behavioural traits reliably is assessed employing a "test-retest reliability" procedure. Since the DS behavioural traits are classified as personality traits, they should be consistent over time and context in an individual [33]. A reliable test would therefore be expected to elicit the same behaviour in an individual when being tested in the same test again ("retest"). Test-retest reliability outcomes are often rather low in pet breed and shelter dogs [0.57 in 34, e.g., 0.58 in 35, average ICC of 0.43 in a meta-analysis of 31 studies in 36] and highly dependent on the sample size, test conditions, target traits, dogs' age, and between-test intervals. Adult dogs and shorter between-test intervals generally showed better results whereas curiosity-based traits performed poorly with shorter test-retest intervals [36, 37]. Nevertheless, some kind of consistency over time has often been reported both for pet and shelter dogs [35, 36]. Whether this will pertain to free-ranging dogs and/or under highly variable street conditions remains to be explored.

In this paper, we therefore assessed the reliability of the 'Domestication Syndrome test battery' in free ranging dogs. The battery was specifically designed to test the DS-related behavioural traits and other traits considered crucial in a number of dog domestication hypotheses, i.e., tameness [DS hypothesis, 7],

hypersociability [17], deferential behaviour [18], and human-dog communication skills [19]. We exposed free-ranging dogs to the test battery twice, approximately six weeks apart, in order to investigate the feasibility of testing in street conditions as well assess the inter-rater, intra-rater, and test-retest reliability of the target behaviours.

Based on previous literature [31, 32], we determined that the inter- and intra-rater reliability should show intraclass correlation of 0.7 or above despite the more unreliable street conditions. For the test-retest reliability, we predicted significant consistency in behavioural displays across tests, however, we expected the test-retest reliability to be lower than for similar tests on pet and shelter dogs because of the greater environmental distractions in the street environment. Furthermore, we expected behavioural consistency to be lower in curiosity-based subtests where the dogs might still remember the target object/context from the previous presentation.

If ultimately deemed reliable, this approach will provide a pathway for a more in-depth understanding of the DS, allowing for an assessment of the correlations between behaviour traits, as well as between behavioural, morphological, and physiological traits. Furthermore, combined with DNA testing, it may provide a valid method for gene-behaviour association studies in free-ranging dogs.

New Introduction

The behaviour of dogs has been of central interest to humanity for millennia – possibly since the early stages of dog domestication 15.000 years ago, hypothesised to have come about through the selection for specific behavioural traits [hypothesised as: tameness: 8, sociability: 17, and/or deferential behaviour: 18, lack of aggression: 19]. While dogs appear already in the work of early scientist such as Darwin [38] or Pavlov [39], it is only in the last 30 years that the research into dog behaviour has been recognized for its intrinsic value, resulting in an explosion of studies focusing on multiple aspects from understanding of human communication, theory of mind, learning processes, and beyond [reviewed by 40, 41]. Likewise, comparative studies have seen a surge of interest in dogs' temperament traits and personality [33], with

comparisons to other species shedding light on the profound influence of our own anthropogenic environment [e.g., as model for aging: 42] and domestication [reviewed in 43] on behavioural development.

Importantly, however, these efforts have primarily revolved around a relatively limited subset of the global dog population – those under immediate human care and control, such as pets, working, and shelter dogs [33]. While easy to access, this subset represents less than a quarter of the global dog population with the other 75-83% consisting of free-ranging dogs [44, 45]. These animals might or might not be owned but share the characteristic that their movement and mate-choice are largely unrestricted by human control [26], even though the majority still resides as street dogs that depend on human settlements for food and shelter [44].

Efforts to explore the behaviour of free-ranging dogs have so far predominantly relied on observational methods [e.g., 46, 47, reviewed in 48]. While this has led to a more in depth understanding of these populations, it is comparatively time-intensive and lacks the experimental manipulation required to answer some of the questions being addressed in the lab with pet, shelter and working dogs. Several challenges come to mind when hypothesizing the use of more standardized test batteries for free-ranging dogs in their natural habitat: First, with little previous knowledge of the test subject, direct interaction might be hazardous regarding aggression or disease status [e.g., rabies: 49]. Second, restraining the animals for a test would be unethical, potentially dangerous, and obstruct the testing of natural behaviour. Hence, tests must rely on voluntary participation and transportable, flexible test setups. Thus, third, the lack of a stable test environment impedes standardisation and the variability and disturbance risk might distort results [50]. Fourth, our limited knowledge of free-ranging dogs' behaviour during experiments requires adaptation of methodologies and apparatuses used with other dog populations, with some inevitable loss of direct comparability, given the different contexts in which animals are tested [28, 29, 51]. Finally, repeated testing remains a challenging prospect given the rapidly changing dynamics in free-ranging populations [reviewed in 48, 51, 52].

Nonetheless, there are compelling reasons to experimentally assess the behaviour and personality of free-ranging dogs. For one, without studying a representative sample of the global dog population encompassing at least some of the vastly different life experiences they may have, our knowledge about dogs' cognitive abilities, human-animal interactions, the connection between genetics and personality, and beyond remains compromised [for more specific ideas see 50, 51]. This is particularly true for evolutionary questions and the understanding of the domestication process. The intensive artificial selection in our modern breed dogs might have de-coupled morphological and behavioural traits hypothesized to co-occur in domestic species [16, 23] and the use of modern breed dogs as a model for domestication has therefore been contested by a number of authors [11, 14]. In support of this and c~~C~~ontrary to the general assumption, the majority of free-ranging dogs are not a mixture of breeds but rather genetically distinct populations that predate the relatively more recent artificial selection and ~~thus~~ breed formation ~~of breeds~~ [26]. Thus, ~~Considering these results~~, free-ranging dogs might actually be a better candidate to elucidate the main domestication process in dogs from a genetic and ecological perspective [53], than modern breed dogs living in 'western-style' pet-owner relationships [20].

Furthermore, free-ranging dogs' pervasive presence in human settlements (e.g., 2,930 dogs/km² in Kathmandu, Nepal [54]) means that millions of people spend their daily life in dogs' direct proximity. Improving our understanding of their behaviour and behavioural profiles could therefore improve both human and animal welfare and aid in effective population control [50, 53].

Some pioneering groups have already initiated important work with free-ranging dogs, demonstrating that an experimental approach, particularly if involving short, one-off tests can indeed be conducted under the variable yet natural conditions they reside in [27-29, 55, 56]. While presenting a valuable start, these tests have focused on single behavioural aspects in short tests [e.g., persistence in 28, pointing in 29], leaving the possibility of longer tests, test batteries, and repeated testing that is needed to explore behavioural profiles, largely unexplored.

Furthermore, critical information regarding the reliability of measures obtained from such tests on the streets remains scarce – information that is vital not only for singular behavioural testing but especially for comprehensive temperament assessments. Several aspects of reliability should therein be considered: the coding reliability which describes how well the behaviours defined in the ethogram and the testing conditions allow for consistency in a raters' observations ~~a rater agrees with themselves~~ over time ("intra-rater reliability") and with others (i.e., "inter-rater reliability") ~~about the behaviours observed (typically on video).~~ , and the "test-retest reliability", which assesses a test's ability to capture the behavioural traits reliably. A reliable test would therein~~fore~~ be expected to elicit the same behaviour in an individual again when being tested in the same test ~~again~~ twice ("retest"). This is particularly important in terms of personality traits, which are defined as being consistent over time and context in an individual [33]. Test-retest reliability outcomes are often rather low in pet breed and shelter dogs, but some kind of consistency over time has often been reported [35, 36]. Whether this will pertain to free-ranging dogs and/or under highly variable street conditions remains to be explored.

In response to this research gap, our study endeavours to address a twofold objective. First, to pilot and evaluate the feasibility of a test battery specifically tailored to street dogs, encompassing a range of behaviours commonly assessed in dog cognition or temperament tests and suggested to be relevant in different domestication hypotheses. These behaviours include human-directed approachability, conspecific sociability through a fake dog test, neophobia and exploration in a novel object test, dogs' understanding of human communication in a pointing test and their use of human-directed communication (gazing) in a begging test, as well as their propensity to shy away from conflict in a tractability test [34, 57-60]. Second, we assess the reliability of this test battery in the natural setting, including inter-rater, intra-rater and test-retest reliability. We draw conclusions from the results on how this test battery could be adapted to be more pervasively used in the field and free-ranging dog testing overall.

Methods

Ethics statement

Ethical approval for this study was obtained from the Ethical committee at the Agronomic and Veterinary Institute Hassan II (Comité d'Éthique de l'Institut Agronomique et Vétérinaire Hassan II) in Rabat, Morocco (Protocol number: CESASPV_2023_05). The free-ranging dogs' participation was voluntary, i.e., dogs were not restrained or forced to take part in the tests, and they were able to leave at any time. Moreover, the procedures were non-invasive and in accordance with the European Union Directive on the protection of animals used for scientific purposes (EU Directive 2010/63/EU).

Study area and population

The testing of the free-ranging dogs was conducted along beach, industrial, and urban areas in the Sous-Massa region in Morocco. The study subjects were part of a big dynamic free-ranging dog population living as scavengers in and around human settlements, hence classifying as street dogs [51]. The behaviour battery was first piloted on a random sample of eight adult ~~free-ranging~~street dogs (4 males, 4 females). Since the test battery was finalized after the first three and thus remained the same for the last five pilot dogs, they were added to the final test sample. Hence, a total of 36 adult ~~free-ranging~~street dogs (20 males, 16 females; 5 pilot plus 31 test sample dogs) were then tested to assess the feasibility and reliability of the finalized test. Twenty-six of those dogs (15 males, 11 females) were found again for the retest.

Procedure

The test battery consisted of six short behavioural subtests and three physiological samplings conducted in direct succession (Fig 1~~2~~). The subtests were designed to resemble behaviour tests that have commonly been used to assess pet, shelter, or working dog temperament traits, dogs' cognitive abilities, or traits that have been suggested as central during the domestication process (see description of the respective subtests).~~The first three subtests were conceptualized to test the DS traits in particular while the last three subtests were designed to assess behaviours emphasized in other selection hypotheses relating to dog domestication. Likewise, t~~The three physiological samples (saliva and hair) were part of the test battery to assess the

feasibility of sampling in such a setting and allow for later use of those samples in future exploration. ~~correlation of the behavioural and morphological to physiological traits and genetic makeup in the future of this test if the test battery turned out feasible and reliable.~~

Fig 1. The sequence of the ~~Domestication Syndrome~~employed test battery. Subtests in light and ~~targeted the DS traits (blue) or traits emphasized in other dog domestication hypotheses (salmon), in addition to~~ physiological sample collections in dark ~~(~~ grey~~)~~.

For the test-retest reliability measure, the test battery was conducted a second time ("retest") with the same study subjects within an average time span of 46.28 days (min. 33, max. 76 days) which was similar to other test validation procedures [e.g., 61, 62-64]. To this end, the location of the initial test was noted and the area was frequently visited again after five to six weeks until the dog was found, identified with previous pictures, and tested again. It was noted if the dog was retested in a different location than the first test (i.e., not in visual vicinity of the previous location). The procedure in the first and retest was exactly the same as described below. For the object-based subtests (Fake dog and Novel object subtest, see below), two slightly different-looking versions were used respectively for the test and the retest to create ~~the necessary~~ novelty. The order of presentation of the two stimuli was randomized across subjects.

Initiation and general circumstances

Two experimenters were involved in each testing occasion: E1 was always the same person (SC) and conducted the actual tests while E2 (three different people) video-recorded, helped set up the subtests, or distracted other dogs if necessary. Dogs were tested individually and had to be alone before and during the test. Tests were conducted between sunrise and 10 a.m. before the areas became too crowded. The experimenters accessed the study area by car and all dogs encountered alone were tested.~~,~~ If at any point during testing, the dog ran off more than 10 m, the ~~current~~ subtest during which the dog left was terminated and E1 tried to lure the dog back. If the dog ran too far to be retrieved, the whole test was terminated.

If anyone but the experimenter approached and visibly distracted the dog (e.g., head turn), the test was interrupted until the criterium was restored. If at any point dogs ~~had~~ showed~~n~~ active signs of aggression, the

test ~~would have been~~was terminated. However, this never occurred. For each dog, location, time, weather, health status, and activity upon arrival of E1 (e.g., sleeping, walking, …) were noted.

~~Tests were conducted between sunrise and 10 a.m. before the areas became too crowded. The experimenters accessed the study area by car. If the dog ran off more than 10 m, the current subtest was terminated and E1 tried to lure the dog back. If the dog ran too far to be retrieved, the test was terminated.~~

The exact setup of each subtest is displayed in Fig 2, the procedure of each subtest is demonstrated in the supporting information (S1 Video).

Fig 2. The set-up of each of the six subtests in the ~~Domestication Syndrome~~presented test battery. The main experimenter (E1) is presented in black (or grey in later stages of the subtest), the assistant experimenter (E2) in white.

~~Sociability~~ Human Approach Test (Subtest 1)

Target: Approachability, h~~H~~uman-directed sociability/ docility, tameness, play. This test was modelled after similar tests ~~which target the same traits and are~~ used in widescale personality assessment tests (e.g., the Dog Mentality Assessment (DMA) in the Swedish Kennel Club [65]) and has in part been used with Ethiopian village dogs [56]. Beside the assumption that human-directed sociability is an essential trait under selection during the domestication process [17], assessing the actual human-directed behaviour in a free-ranging population is provides valuable insights for disease- and population control.

Here, E1 stepped out of the car and whistled shortly to grab the dog's attention. She then stepwise proceeded to 1) stand quietly looking at the subject for 30 s, 2) called the dog and made friendly gestures (snapping, tapping on leg) for another 30 s, 3) approached the dog for 30 s up to 2 m distance, 4) stood for another 30 s, 5) approached the dog to one body length distance and paused another 30 s, and 6) crouched for another 30 s all while continuing to call the dog over. If the dog approached to less than 10 cm of E1 during any of those phases, E1 kneeled and tried to pet the dog for 30 s. If tolerated, E1 used a small brush to collect hair samples. The dog was then invited to play tug of war with a rag toy (1 m long) for 30 s.

Fake Dog Test (Subtest 2)

Target: Conspecific-directed sociability and aggression. Several studies support the idea that stuffed dogs can be used to reliably assess dogs' initial behaviour towards conspecifics [57, 66]. A reduction in conspecific aggression has been hypothesised as an important shift from wild to domestic types [3], and understanding intraspecific aggression in free-ranging dogs can provide important applications for the local population.

Hence, subjects were exposed to a "Giant Jack Russel Terrier" stuffed fake dog (brand: 'Melissa & Douk'; wither height 28 cm; S2 Supplementary Information). The two used versions differed in ear posture (pointy or droopy), tail length (medium and long), and colour (one original, one wrapped in dark bags) (Fig 3A) in the test and in the re-test respectively, in a randomized order across subjects. The fake dog was positioned 5 m from the car behind a V-shape visual barrier with its opening towards the car. This visual barrier allowed only the approaching subject but no other surrounding dogs to see the fake dog. E1 guided the subject towards the fake dog (if needed with food) and quickly walked >5 m away as soon as the subject saw the fake dog. The subject's behaviour was observed for 2 min.

Novel Object Test (Subtest 3)

Target: Neophobia (exploration, novelty-seeking, curiosity). Presenting a dog with an unknow object is commonly used to assess novelty-seeking and boldness [34, 37], and explorative-behaviour is hypothesised to be lower in domesticated animals as part of the domestication syndrome [3].

To present an object that was truly novel to the dogs, we fixed a 90 – 100 cm high foil balloon with a wooden stick on top of a remotely controlled car (31x18x18 cm, Model: DEERC DE42 RC; S2 Supplementary information). Two balloon forms were used (dinosaur, a number (zero, five) sprayed in neon green and blue; Fig 3B) for test and retest and randomized between study subjects. The set up was the same as the Fake dog test. As soon as the dog saw the novel object, E2 started moving it back and forth for 1 min through remote control. The toy was then left inactive for another 1 min. The dog's behaviour was observed for the full 2 min.

Fig 3: The two versions of a) fake dogs used in the Fake Dog test and b) novel objects used in the Novel Object test.

Pointing Test (Subtest 4)

Target: Dog-human communication skill, willingness to follow commands, cooperative communication [reviewed in 20, 67]. Studies have shown that a wide array of species, including canids, can follow pointing cues [for review see 67]. Exploring this phenomenon in free-ranging dogs with differing levels of human experience will further our understanding of the role of life-experience vs. species predisposition (but see [29] for pioneering work in this regard).

The test started with four warm-up trials: E1 placed a baited bowl (12 cm diameter, 10 cm height) on the ground 2 m from the dog, standing 1 m away herself and verbally called the dogs attention. After the dog ate the food, the bowl was picked up, baited, and placed down again to familiarize the dog with the fact that the bowl contained retrievable food. The subtest was terminated if the dog did not approach the bowl within 30 s two times in a row. The warm-up trials were followed by twelve test trials: one empty and one baited bowl (both rubbed in food for smell, baited outside of the dog's view) were placed 1.5 m apart 2 m from the dog. E1 stood equidistant behind the bowls, attracted the dog's attention, and upon eye contact, pointed to the baited bowl for 2 s (momentary distal pointing). The dog was allowed to eat the food if it approached the correct bowl on the first choice, otherwise the bowls were immediately collected by E1. Each trial could be repeated twice if the dog did not choose within 30 s, followed by one repetition of the warm-up trial (i.e., only one bowl with food). If after the third repetition, the dog did not choose, the subtest was terminated. We used six different randomized sequences related to the positioning of the baited/pointed bowl to the right or left of the experimenter (e.g., RLRLLRLRRLLR) that were called out to E1 by E2.

Begging Test (Subtest 5)

Target: Looking behaviour, suggested to be help-seeking behaviour [58, reviewed by 68, but see for alternative explanations: 69, 70]. Also a behaviour suggested as a trait under selection during

domestication but the wide variability between individuals depending on human socialisation calls for further investigation with differentially socialised populations [71, 72].

[58, reviewed by 68]. The dog was allowed to eat from the baited bowl three times. Afterwards, the bowl was visibly baited and placed out of reach but still visible on the car, a wall, or a tree. E1 stepped 1 m to the side and faced away from the bowl with a neutral facial expression. The dog's behaviour was observed for 1 min.

Tractability test (Subtest 6)

Target: Social inhibition, tractability/deferential behaviour (i.e., the ease to yield to higher command [18]). According to the deferential hypothesis [18], tractability has been a central selection factor in dog domestication. Furthermore, assessing dogs' inclination to avoid conflicts with humans in a resource scenario may also have it uses in a more applied setting.

E1 gave the dog a piece of food, then took a big piece of meat, showed it to the dog, and visibly placed it on top of a small stool (40 cm height). E1 loudly stepped between dog and stool, stared the dog in the eyes and firmly said "No, it's mine!". If the dog attempted to get to the stool, E1 loudly stepped in their way and repeated "No" in a firm tone. After 1 min of this, E1 stepped 1 m to the side and stood still for 30 s while looking at the food. Finally, E1 turned around and looked away for 30 s. The test was finished as soon as the dog ate the food or after the last 30 s.

Video coding

The tests were recorded by an action camera (4K 24fps WiFi Sports Action Camera or Action Cam Jeemak WiFi) fixed on E1's chest and a camera (Sony HDR-CX405 Full HD Camcorder, hand-held (subtest 1) or on a tripod (subtest 2-6)) handled by E2. BORIS (version 7.13.8, (Friard & Gamba, 2016)) was used for the video coding.

The ethogram aimed at capturing the target behaviours described for each subtestof interest related to the traits in the DS or other domestication hypotheses in dogs. We used a bottom-up approach by initially coding

55 variables with different modifiers (i.e., the person or object the behaviour was coded towards) that measured almost every potentially relevant aspect in the different subtests. The coded variables are displayed in Table 1 with a detailed ethogram in S32 Table. Social behaviours were based on the ethograms used at the Wolf Science Center of the University for Veterinary Medicine, Vienna [48, 73, 74], and to observe free-ranging dogs in Italy [47, 75].

This list of variables was narrowed down by 1) assessing their codability through the inter- and intra-rater analysis (for acceptable agreement see Statistical Analysis), 2) assessing the variables' centrality in the subtest by analysing their frequency (i.e., excluding variables that were displayed in less than 10% of tests), and 3) assessing their consistency through the test-retest reliability. Only behavioural variables that passed step 1) and 2) went on to be analysed in step 3) to ensure that the test-retest reliability was based on reliably coded and not overly skewed data [76].

Table 1. Coded behavioural variables and occurrence across subtests.

Category	Behavioural variable	SociabilityHuman approach	Fake Dog	Novel Object	Pointing	Begging	Tractability
Proximity	Close	E1	Fake dog, E1+E2	Novel object, E1+E2	-	E1	E1, stool
	Medium	E1	Fake dog, E1+E2	Novel object, E1+E2	-	E1	E1, stool
Tail	Wagging	E1	Fake dog	Novel object	E1	E1	E1
	Between legs	Yes	Yes	Yes	-	Yes	Yes
Gazing		E1	Fake dog	Novel object, E1+E2	-	E1	E1
Vocalization	Barking	E1	Fake dog	Novel object	-	E1	E1
	Growling	E1	Fake dog	Novel object	-	E1	E1
	Whining	E1	Fake dog	Novel object	-	E1	E1
Displacement	Nose-licking	Yes	Yes	Yes	-	Yes	Yes
	Body shake	Yes	Yes	Yes	-	Yes	Yes
	Stretching	Yes	Yes	Yes	-	Yes	Yes
	Yawning	Yes	Yes	Yes	-	Yes	Yes
	Sniffing the ground	Yes	-	-	-	-	-
Physical contact	Biting	E1	Fake dog	Novel object	-	E1	E1
	Body contact	E1	Fake dog	Novel object	-	E1	E1
	Jumping	E1	Fake dog	Novel object	-	E1	E1
	Licking	E1	Fake dog	Novel object	-	E1	E1
	Mouthing	E1	Fake dog	Novel object	-	E1	E1
	Pawing	E1	Fake dog	Novel object	-	E1	E1
	Sniffing	E1	Fake dog	Novel object	-	E1	E1

Reactions	Stand tall	E1	Fake dog	Novel object	-	E1	E1
	Bare teeth	E1	Fake dog	Novel object	-	E1	E1
	Lunge	E1	Fake dog	Novel object	-	E1	E1
	Head dip	E1	Fake dog	Novel object	-	E1	E1
	Belly exposure	Yes	Yes	Yes	-	Yes	Yes
	Flee	E1	Fake dog	Novel object	-	E1	E1
	Crouch	E1	Fake dog	Novel object	-	E1	E1
	Risk assessment	E1	Fake dog	Novel object	-	E1	E1
	Play	E1	Fake dog	Novel object	-	E1	E1
	Friendly	E1	Fake dog	Novel object	-	E1	E1
	Bow	E1	Fake dog	Novel object	-	E1	E1
**Marking**		Yes	Yes	Yes	-	Yes	Yes
**Defecating**		Yes	Yes	Yes	-	*Yes*	Yes
**Non-visible**		Yes	Yes	Yes	Yes	Yes	Yes
**Disturbance**		Yes	Yes	Yes	Yes	Yes	Yes
**Subtest specific variables**			Latency to approach fake dog	Latency to approach novel object	Observation of gesture: no/yes	Attempts to reach bowl	Latency to eat food
		Phase of first approach		Phase of first approach	Successful choice: no/yes		Phase of eating food
			Genital sniffing		No choice	2-way gaze alternation	
						3-way gaze alternation	

The table shows all behavioural variables (column 2) of a certain category (column 1) that were coded in the respective subtests (columns 3 - 8). The words in the subtest columns describe towards what person or object the behaviour was coded, while 'yes' means that the behaviour was coded regardless of direction, and a minus (-) indicates that the behaviour was not coded in the subtest. Behavioural variables were either coded as duration (D), frequency (F), occurrence (O), or latency since the beginning of the respective subtest (L). Coloured cells indicate that the variable occurred in less than 10 % of tests and was thus excluded from the test-retest analysis.

For the intra-rater reliability measures, a random subsample of 20% of the test videos (n=13; balanced between test and retest, full test batteries and tests that were terminated halfway through) were re-coded by the initial rater after three weeks. For the inter-rater reliability, a random subsample of 20% of the full-test videos (n=9) were coded independently by a second rater [34, for similar methods see 77]. Beforehand, the second rater was trained on three sample videos and the ethogram was further clarified where needed.

Statistical analysis

Inter- and intra-rater reliability was analysed for each behavioural variable summed across all subtests and modifiers to assess their codability. Test-retest reliability, on the other hand, was analysed for every single variable by modifier and subtest. Occurrence variables (e.g., in which phase the dog approached, termination, etc.) were converted into ordinal variables before analysis. To normalize the data for comparison between test and retest, the time the dog was not visible or a disturbance was coded was subtracted from the total time of the subtest of the individual, and the behavioural data was then divided by this normative time. All individual subtests in which more than 70% of the time was coded as disturbance and/or not visible were excluded from further analysis. In case of the test-retest reliability, the specific subtest in both the test and the retest were excluded to allow for fair comparison.

The statistical analysis was then conducted in R (Version 4.2.2 [78]). To account for the systematic errors between raters/ rating occasions, intraclass correlation coefficients (ICCs) were calculated to assess the reliability measures [79, 80]. Additionally, considering that both raters/rating occasions coded the same videos and that the goal was to generalize the reliability results, a two-way random effects ANOVA with an absolute agreement estimate was used to assess the inter- and intra-rater reliability [reviewed by 80]. According to the classification by Koo and Li (32), an ICC below 0.5 is considered poor reliability, between 0.5 and 0.75 it is moderate, between 0.75 and 0.9 it is good, and between 0.9 and 1.0 the reliability is considered excellent. For a behaviour t~~T~~o be regarded as having acceptable coding reliability and move on to the test-retest reliability analysis, the ~~target~~ ICC threshold for the inter- and intra-rater reliability was set to a moderate 0.70 [31], which is in accordance with similar studies [weighted average of 0.68 in a review of six studies by 33, 0.77 in 81]. [weighted average of 0.68 in a review of six studies by 33, 0.77 in 81]. However, careful reconsideration was employed in case the threshold was just about missed by taking both coding reliabilities (inter- and intra-rater) into consideration and examining the p-value (assuming significance level α=0.05). For the test-retest reliability in the repeated sample, a two-way mixed effects ANOVA with absolute agreement estimate was applied [32]~~(Koo & Li, 2016).~~ . Since significance largely depends on sample size and significant test-retest reliabilities in pet dog tests commonly range around an ICC of 0.5 which would usually be classified as poor to moderate reliability [0.57 in 34, e.g., 0.58 in 35,

average ICC of 0.43 in a meta-analysis of 31 studies in 36], a test-retest reliability with a p-value below 0.05 rather than a specific ICC was chosen as reliability indicator. Only variables that were displayed in more than 10% of the testing occasions were included in the test-retest reliability to circumvent unreliable analysis of heavily zero-inflated and low-prevalence data [for a similar approach see 31, 76, 82]. For the ordinal variables, two-way ANOVA’s with absolute agreement estimate (equivalent to weighted Cohens’s Kappa according to Fleiss and Cohen (83)) were used to analyse the coding and test-retest reliability. If the ICC was poor (i.e., below 0.5) for a certain variable, systematic biases in the measurements of both rating occasions were explored through scatter plots and variables were excluded if necessary. To control for the test-retest reliability being low due to disturbances during the tests, we conducted the same analysis again but excluded all individual subtests in which any disturbances were coded (46 of 235 subtests). Disturbance had been coded when the dog clearly turned its head towards an interfering human/dog/other animal for more than three seconds during a running subtest and while the test subject was visible on the video.

Lastly, because curiosity or exploration based tests may be more prone to lower consistency due to repeated exposure to similar stimuli [37], we tested if the dogs systematically showed a decreased reaction (exploration and fear) in the fake dog and novel object re-test compared to the first test. To that end, applicable variables that were retained in the test-retest reliability (i.e., had good coding reliability and occurred in more than 10% of tests) were summed into an exploration-related and a fear-related variable [therein drawing from 21, 84] for each of the two subtests respectively. After transforming these four left-skewed variables with a square root transformation, a paired t-test was used to compare whether they differed significantly between test and retest.

Results

Subject characteristics

Out of the 36 initially tested ~~free-ranging~~street dogs, 23 (14 males, 9 females) were tested in the entire test battery, while nine dogs (5 males, 4 females) terminated the test during the Human approach~~sociability~~ subtest and four dogs (1 male, 3 female) after the first two or three subtests by running away. For the retest,

26 and thus 72 % of the 36 initially tested street~~free-ranging~~ dogs were found again: 19 of those were dogs who went through the full test battery initially and 7 were dogs who had run away during the first testing. Fourteen~~ with 14 being~~ were retested in a different location than the first test. All 26 dogs completed the same number of subtests in the retest as they did in the initial test.

Changes in methods and variables

After the test was adapted in the pilot testing, some additional changes from the above-presented procedure had to be made throughout the main testing period. For one, the play-part (rope presentation for tug-of-war) in the Human approach ~~sociability~~ test caused four (28.57%) of the first 14 dogs to run away and terminate the test. For two more, the play phase had to be terminated to keep the dog from leaving. Thus, the play phase was dropped half-way through the main sample and the variable 'Interacting with the toy' was excluded from the analysis.

In addition, two more variables had to be excluded due to the following reasons: 'Auto-grooming' because the dogs had varying amounts of parasites, wounds, and dirt in their fur which influenced the scratching rate, and 'Sniffing the ground' in all but the ~~Sociability~~ Human approach test because food was dropped on the ground during the subtests and the different ground surfaces influenced the dogs' sniffing behaviour.

Intra- and inter-rater reliability

Both the intra- and inter-rater reliability coding, assessed across all subtests and modifiers, is reported in Table 2. All variables passed the target ICC-threshold of 0.7 and had a significant p-value at 0.015 or below (the exact statistical outputs for each variable are reported in S~~3~~4 table). The average of both inter- and intra-rater reliability for all variables was above the target threshold of an ICC of 0.7, with a good reliability of 0.84 in the inter-rater and 0.93 in the intra-rater analysis.

There were only few variables that did not clear the threshold. 'Tail between the legs' and 'Mouthing' had moderate inter-rater reliability, meaning they still had an ICC above 0.5, and the p-value was below 0.05. Likewise, 'Medium proximity' had a moderate reliability within and between raters. The inter-rater

reliability for ‘Head dip’, ‘Friendly’ and ‘Stand tall’, on the other hand, were poor with an ICC below 0.5 with a p-value above 0.05.

While ‘Tail between the legs’ and ‘Mouthing’ did not clear the initially defined threshold, they were both close and had a p-value of 0.02 which is substantially below the usual 0.05 significance level. Since previous studies have deterred from the arbitrary 0.7 ICC threshold in favour of significant p-values ($p<0.05$) [33], and both present crucial behaviours in dogs, we tentatively chose to keep them in the analysis going forward but treat their interpretation with utmost care. The other behaviours fell below the 0.05 significance level and were thus excluded from further analysis.

Table 2. Summary of the coding reliability across subtests.

GENERAL			
**Category**	**Behavioural variable**	**Inter-rater ICC**	**Intra-rater ICC**
**Proximity**	Close	0.90	0.97
	Medium	0.53	0.52
**Tail**	Wagging	0.91	0.91
	Between legs	0.64	0.99
**Gazing**		0.90	0.98
**Vocalization**	Barking	NA	NA
	Growling	0.80	NA
	Whining	NA	NA
**Displacement**	Nose-licking	0.88	0.96
	Shaking	0.80	NA
	Stretching	0.88	1.00
	Yawning	0.94	0.97
	Sniffing the ground	0.82	0.79
**Physical contact**	Biting	NA	NA
	Body contact	1.00	0.74
	Jumping	0.95	0.98
	Licking	NA	0.84
	Mouthing	0.64	0.95
	Pawing	NA	0.99
	Sniffing	0.97	0.98

SUBTEST SPECIFIC			
**Subtest**	**Behavioural variable**	**Inter-rater ICC**	**Intra-rater ICC**
**~~Sociability~~Human approach**	Phase of first Approach	1.00	1.00
**Fake Dog**	Latency	1.00	0.97
	Genital sniffing	0.92	1.00
**Novel object**	Phase of first approach	1.00	1.00
	Latency	0.85	1.00
**Pointing**	Observation of gesture: yes	1.00	0.98
	Observation of gesture: no	1.00	0.99
	Successful choice	1.00	1.00
	Unsessful choice	1.00	1.00
	No choice	1.00	1.00
**Begging**	Attempts to get bowl	0.99	0.99
**Begging/ Tractability**	2-way gaze alternations	0.82	0.79
	3-way gaze alternations	0.86	0.87
**Tractability**	Latency	0.90	1.00
	Phase of eating	1.00	1.00

**Reactions**	Stand tall	-0.03	0.91
	Bare teeth	NA	NA
	Lunge	NA	NA
	Head dip	-0.08	0.85
	Belly exposure	NA	NA
	Flee	0.77	0.94
	Crouch	0.90	0.79
	Risk assessment	0.99	0.86
	Play	0.98	0.98
	Friendly	0.44	0.97
	Bow	0.81	0.76
**Marking**		NA	0.99
**Defecating**		NA	NA
**Average**		**0.84**	**0.93**

For each variable, the inter- and intra-rater reliability analysed across subtests and modifiers is displayed. Based on similar studies, a coding reliability of an ICC of 0.7 or above was regarded as acceptable (values with no specific highlight). Moderate reliability below this threshold (i.e., 0.5<ICC<0.7) is highlighted in light grey, and poor reliability (i.e., ICC<0.5) is marked in dark grey. All variables above an ICC of 0.7 had a p-value below 0.015, variables with an ICC between 0.5 and 0.7 had a p-value below 0.05, and all variables below an ICC of 0.5 had a non-significant p-value (p>0.05). Detailed analysis results with the 95% confidence interval, F-value statistic, degrees of freedom, and p-value can be found in S34 Table). Variables marked with NA did not occur in the coded tests.

Exclusion of infrequent variables

In the next step, variables that did not occur in more than 10% of the respective subtests were identified (Table 1) and excluded from the test-retest reliability analysis. In short, close proximity, tail wagging, gazing, nose licking and yawning were the only variables that occurred frequently in all subtests. On the other hand, growling, whining, stretching, shaking, biting, licking, mouthing, marking, defecating, baring teeth, lunging, belly exposure, and bow were not displayed in more than 10% of tests in any of the subtests. Even after summing most of them into one pooled ‘stress’ variable [following the classifications of 85, 86], they did not pass the 10% occurrence threshold and were hence excluded from the test-retest analysis. All

~~subject~~subtest-specific variables except ‘attempt to reach the bowl’ in the begging test, occurred frequently enough to pass the 10% threshold.

Test-retest reliability

The final test-retest reliability analysis was conducted for each variable by subtest and modifier, only taking the variables into account that 1) were not already dropped from the ethogram/tests throughout the testing (i.e., auto-grooming, sniffing the ground, play), 2) had an acceptable coding reliability~~ (i.e., ICC > 0.7)~~, and 3) occurred in more than 10% of all tests. The resulting test-retest reliabilities are reported in Table 3.

The average test-retest reliability was poor with an average ICC of 0.23~~19~~. To see whether the poor outcome was influenced by disturbances during the tests that altered the dogs’ behaviour, we conducted the same analysis but excluded all subtests in which a disturbance had been coded (i.e., when the dog clearly turned its head towards an interfering human/dog/other animal for more than three seconds during a running subtest and while the test subject was visible on the video). While this changed the reliability of some behaviours, the average test-retest reliability did not improve (Table 3; the exact statistical outputs of both analyses can be found in S5~~4~~ Table).

To summarize by subtest, the Sociability test performed well with ten out of 15~~6~~ behaviours showing significant test-retest reliability, resulting in an average test-retest reliability of 0.32~~0~~. In the Fake dog test, only ‘tail between the legs’ had a moderate retest reliability, with ‘close proximity to the fake dog’ and ‘nose-mouth licking’ also being moderately reliable after tests with disturbances were removed. The average test-retest reliability was low (ICC = 0.06~~5~~). Similarly, only ‘close proximity to E1 and E2’ were significant in the Novel object test, being joined by ‘nose-mouth licking’ after disturbed tests were excluded, resulting in an average test-retest reliability of 0.12~~0~~. The average reliability of the Pointing test was significant with an average ICC of 0.51 with all subtest-specific variables, as well as ‘tail-wagging’, showing moderate test-retest reliability. The Begging test had a low test-retest reliability (average ICC = 0.16~~4~~) with only the ‘close proximity to E1’ being significant across time when only looking at tests without disturbances. Lastly, ‘close proximity to E1’, ‘gazing at E1’, the ‘phase in which the dog ate the food’, ‘three-way gaze alternation

between person and stool’ and ‘fleeing’ had significant reliability in the Tractability test, with the addition of ‘nose-mouth licking’ after disturbances were removed. The average test-retest reliability was 0.224.

Test-retest comparison for the curiosity-based subtests

The summed variables constituted of the following behaviours: “Exploration”-variable: ‘close’ and ‘medium proximity’, ‘gazing’, ‘tail wagging’, ‘friendly’, and ‘sniffing’ the fake dog/novel object, as well as ‘genital sniffing’ in the case of the fake dog. “Fear”-variable: ‘head dip’ and ‘tail between the legs’ for the fake dog subtest. Only ‘flee’ was retained as fear-representing behaviour in the novel object but was displayed so rarely, that it could not be analysed here.

The dogs showed significantly less exploration and less fear behaviours towards the fake dog in the retest than the first test (exploration: $t(14)=3.16$, $p<0.01$; fear: $t(15)=-3.08$, $p<0.01$). Exploration behaviours shown towards the novel object did not differ between test and retest ($t(11)=1.0$, $p=0.34$).

Table 3. Summary of the test-retest reliability in all retained variables.

Category	Behavioural variable	~~Sociability~~Human Approach			Fake Dog			Novel Object			Pointing			Begging			Tractability		
		M.	All	w/D	M.	All	w/D	M.	All	w/D	M.	All	w/D	M.	All	w/D	M.	All	w/D
Proximity	Close	*E1*	0.71	0.77	*FD*	0.20	0.74	*NO*	-0.09	0.06	-			*E1*	0.8	0.76	*E1*	0.75	0.72
					E1+E2	-0.08	-0.17	*E1+E2*	0.74	0							*Stool*	-0.04	-0.02
	~~Medium~~	~~*E1*~~	~~0.22~~	~~0.34~~	~~*FD*~~	~~0.12~~	~~0.23~~	~~*NO*~~	~~0.01~~	~~0.01~~	~~-~~	~~-~~	~~-~~	~~*E1*~~	~~-0.16~~	~~-0.25~~	~~*E1*~~	~~0.89~~	~~0.87~~
					~~*E1+E2*~~	~~0.03~~	~~-0.07~~	~~*E1+E2*~~	~~-0.08~~	~~0~~							~~*Stool*~~	~~-0.05~~	~~-0.05~~
Tail	Wagging	*E1*	0.61	0.62	*FD*	0.14	-0.12	*NO*	0.04	NA	*E1*	0.5	0.2	*E1*	0.36	0.3	*E1*	-0.14	-0.06
	Between legs	*Yes*	0.53	0.52	*Yes*	0.71	0.72											0.29	0.28
Gazing		*E1*	**0.35**	0.34	*FD*	0.16	0.11	*NO*	-0.35	-0.06				*E1*	0	0.24	*E1*	**0.48**	0.44
								E1+E2	-0.26	-0.08				*Bowl*	0.34	0.12	*Stool*	0.18	0.14
Vocalization	Barking	*E1*	0.85	0.85	*FD*	0.12	0.12												
Displacement	Nose-licking	*Yes*	0	-0.01	*Yes*	0.16	0.56	*Yes*	0.37	0.61	-			*Yes*	-0.2	-0.28	*Yes*	0.41	0.51
	Yawning	*Yes*	-0.03	-0.08	*Yes*	-0.06	0	*Yes*	0.48	0.45	-			*Yes*	-0.09	-0.1	*Yes*	-0.21	-0.2
	sniffing the ground	*Yes*	-0.04	-0.04	*Excl.*			*Excl.*			-			*Excl.*			*Excl.*		
Physical contact	Body contact	*E1*	0.55	0.55															
	Jumping	*E1*	0.5	**0.46**															
	Sniffing	*E1*			*FD*	0.03	0.19	*NO*	-0.04	0	-			*E1*	-0.09	-0.05			
Reactions	Flee	*E1*	-0.08	-0.16				*NO*	0.32	NA							*E1*	0.76	0.58
	Crouch																*E1*	-0.05	-0.02
	Friendly	*E1*	**0.45**	0.56	*FD*	-0.07	0.15							*E1*	0.34	0.35	*E1*	0.13	0.14
	Head dip	*E1*	-0.08	-0.09	*FD*	-0.21	0	*NO*	0	NA							*E1*	-0.07	-0.02
	Play	*E1*	-0.03	-0.06															
	Risk assesment				*FD*	-0.04	-0.04												
	Stand tall				*FD*	-0.12	-0.29												
Subtest-specific variables	Phase of first Approach	*E1*	**0.49**	0.51				*NO*	0.2	0	-			-			*Stool*	0.65	0.75
	Latency of first approach				*FD*	-0.15	0.2	*NO*	0.05	-0.9	-			-			*Stool*	-0.11	-0.08

	Genital sniffing				*FD*	0.15	-0.15												
	Observation of gesture: yes	-			-			-			*E1*	0.51	0.12	-			-		
	Observation of gesture: no	-			-			-			*E1*	**0.33**	-0.2	-			-		
	Successful choice	-			-			-			*Bowl*	0.64	0.38	-			-		
	Unsucessful choice	-			-			-			*Bowl*	0.55	0.06	-			-		
	No choice	-			-			-			*Bowl*	0.52	0.17	-			-		
**Gaze alternations**	2-way	-			-			-			-			*E1/ bowl*	0.03	-0.08	*E1/ stool*	0.14	0.07
	3-way	-			-			-			-			*E1/ bowl*	0.1	0.08	*E1/ stool*	**0.38**	**0.38**
**Average per subtest**			**0.320**	**0.320**		**06.05**	**0.13**		**0.120**	**0.01**		**0.51**	**0.12**		**0.164**	**0.132**		**0.224**	**0.234**
**Average across the test battery**			**0.2319**	**0.17**															

For each variable and subtest, the modifier (M.) and the test-retest reliability is displayed for all tests (All) and for only the tests did not have any coded disturbances (w/D) respectively. The modifiers were the main experimenter (E1), the fake dog (FD), the novel object (NO), the bowl, or the stool. Test-retest reliabilities with a p-value below 0.05 were regarded as acceptable. Behaviours with non-significant test-retest reliability are indicated with a light-grey background and in bold if they were significant but regarded as poor reliability (i.e., below an ICC of 0.5). Variables that were excluded due to low occurrence (i.e., less than 10% of testing occasions) are not shown unless their exclusion only applied to certain subtests. In that case, they are left empty or marked with NA if it only applies to the analysis without disturbances. Variables marked with a minus (-) were not coded in the respective subtest. Detailed analysis results with the 95% confidence interval, F-value statistic, and p-value can be found in S45 Table.

Discussion

In this study, ~~Herein~~ we presented what, to our knowledge, is the first attempt at testing a population of free-ranging dogs in a behavioural test battery and report on feasibility and reliability of the test measures~~a new Domestication Syndrome test battery conducted on free-ranging dogs and the assessment of its validity~~. Testing the dogs in their natural environment was indeed feasible and the coding reliability was high, indicating that this (relative to preview studies) long and complex behaviour tests can be conducted with ~~free-ranging~~street dogs. The range from good to poor test-retest reliability, on the other hand, reflects the challenges dog personality tests on the streets are faced with and we provide possible explanations and further recommendations for the use of this battery and behavioural testing of free-ranging digs in general. ~~before the test battery can be used as a reliable tool to investigate the domestication syndrome and other dog domestication related traits.~~

Although the subtests ~~used~~ in th~~is~~e ~~Domestication Syndrome~~ test battery were adapted from mostly enclosed, standardized pet and working dog behaviour~~personality~~ assessments [but see 29, 34, e.g., 37, 57] ~~[but see 29, 34, e.g., 37, 57~~], bringing the test to the free-roaming dogs and standardizing the set up in the streets and beach environments of our populations turned out to be surprisingly manageable, ~~feasible and (relatively) easy to handle~~, and there were no terminations due to aggression towards the person or the equipment. More importantly, most dogs ~~went through~~completed all six subtests and sample collections despite an average test duration of 30 minutes, suggesting that ~~free-ranging~~street dogs (at least those of this population) can remain motivated throughout longer and more complex procedures ~~not only be tested in short and engaging tests~~ than those used so far [e.g., 28, 55, 87]. ~~but can remain motivated throughout longer and more complex procedures~~.

Similarly, the concern that testing and filming in the variable street conditions would negatively impact the coding reliability was unfounded. Our results surpassed the target of an ICC of 0.7 with a good average inter- and intra-rater reliability of 0.84 and 0.93 respectively. Contrary to our expectations, the coding reliability in this street test was thereby on *a par* or even better than many pet and working dog studies [ICC

0.68 in meta-analysis by 33, ICC >0.9 in 34, ICC 0.77 in 81], suggesting that our chosen variables were not only well defined but could also be coded reliably despite the more difficult challenging street conditions. Similarly encouraging inter-rater results have been reported for a few other street dog tests as well [28, 29], further underlining that such results are achievable for street testing in general.

However, when looking at the reliability of the individual variables, a few did not meet the there were a handful of variables that did not pass the reliability threshold. Among the ones that still had moderate reliabilityNotably, 'medium proximity', i.e., two body lengths distance from the dog to the experimenter or object of interest, stood out since it was the only one that was low on both inter- and intra-rater reliability. With the constant adaptation of the camera angles to adjust for the variability of the street conditions, Iit might thus be best to stick to distances that are easier to identify such as to drop this variable in favour of the more reliable 'close proximity' (i.e., one body length distance). More importantlyIn addition, 'head dip', 'stand tall', and 'friendly' all had poor agreement between the raters, which might be due to the complexity of these behaviours, . This is not particularly surprising since these were the most complex behaviours, with their definitions being relyingiant on a variety of subtle behaviours and body postures exhibited together (for the detailed definitions see S2 Table). While these definitions were adapted from previously used tests ethograms [48, 73, 74], the filming in street conditions likely made the subtle cues harder to detecteasier to miss or the behaviours might have been misinterpreted in the experimental setting that somewhat differed from the natural conspecific setting they were initially defined in. Given the poor coding reliability of those traits, they should thus be dropped from the presented ethogram unless a more reliable definition or training method can be found and validated, and care should be taken in the establishment of future tests to balance between the complexity of target behaviours and filming feasibility in the streets.In addition, our two raters had very different experience with dogs, which has been suggested to strongly influence coding outcomes [88-90]. Indeed, only one of the raters was able to pick up on more subtle cues which could not be mitigated despite the discussions in the training phase. Based on the presented poor coding reliability of those traits, they should thus be dropped from the ethogram unless a more reliable definition or training method can be found and validated.

While these outcomes so far paint a positive picture for street dog testing~~support the feasibility of the Domestication Syndrome test battery~~, the feasibility in terms of capturing the desired traits cannot be claimed without looking at the tests' ability to elicit the same behaviours consistently over time. The presented test battery resulted in an average test-retest reliability of 0.23~~19~~, which, according to ~~Koo and Li~~ [32], is regarded as poor reliability. While the definition of personality traits is firmly bound to ~~–~~their consistency over time and contexts [33], capturing such behavioural consistency has been an issue for many test batteries. A meta-analysis of 31 studies revealed an average trait consistency of 0.43 in pet, shelter and working dogs, which was reported to be significant~~,~~ although the studies within displayed highly variable results ranging from -0.73 to 1.00 [36]. ~~Similarly, w~~We found a similarly high variability ~~between~~ in the test-retest reliability of the behaviours we assessed, with the subtest ~~average~~ reliability ~~of the subtests~~ varying between a moderate~~ly reliable~~ ICC of 0.51 for the pointing test and a poorly reliable 0.0~~5~~6 for the fake dog test. Capturing consistent personality or behavioural scores over time is hence an issue not unique to the testing of street dogs in their natural habitat.~~Hence, the issue of capturing consistent personality or behavioural scores over time is not unique to the Domestication Syndrome test battery.~~

Intriguingly, most of the variables that were reliable over time were human-directed behaviours, including proximity to ~~the experimenter,~~and tail wagging at the experimenter, body contact, and time of first approach. Likewise, the subtests in which the experimenter constantly interacted with the dog (i.e., ~~sociability~~human approach, pointing, tractability test) had the most reliable variables and highest average test-retest reliability over time. This is in accordance with high reliability and strong heritability for sociability and biddability in pet dogs [e.g., 34, 37, 91] as well as dogs' performance during pointing tasks [92, 93] which, with the same consistency result in free-ranging dogs, might point to a genetic basis on which the selection process may have acted upon during dog domestication [17].~~ previous dog personality assessments that have identified sociability as one of the most reliable character traits across time [e.g., 34, 37] with a strong heritability for sociability and biddability in pet dogs [91]. The finding that this is not only strong in breed/pet dogs, but also free-ranging dogs highlights the possibility that sociability and/or tractability may~~

~~have a genetic basis and hence provide a likely basis on which the selection process may have acted upon during dog domestication [17, 18].~~

~~Cognitive skills honed for dog-human communication have been suggested as another factor potentially influential during dog domestication [19]. Previous pointing studies did not only find consistent performance in dogs from puppy into adulthood and across several months in adult dogs [92], but also provided evidence that dog's gazing and choosing behaviour during pointing seems to have a high heritable component [93]. Interestingly, this is further supported by our finding that observation and following the pointing gesture was reliable over time in our sample of free-ranging dogs.~~

~~Overall, the outcome that several main variables and most of the subtest-specific variables of the sociability, pointing, and tractability test were reliable over time suggests that these tests can be used reliably to assess the target behaviours. Only the play part in the sociability test should be avoided since it did not elicit play with a majority of the dogs, possibly due to a lack of experience with this kind of play behaviour or, more importantly, its perception as a threatening stimulus similar to aversive human interactions which may be prominent in their lives [e.g., 52].~~

More important in terms of street dog testing, however, is that the discrepancy in reliability between these social tests and behaviours and the other subtests (fake dog, novel object, and begging test in particular) might be driven by their high interactivity, leaving the animal little time to be distracted. Free-ranging dogs have been shown to gaze at a person that does not attend to them directly less than at an attentive person while such a difference does not occur in pet dogs [27]. Moreover, the reality of testing in the free-ranging dogs' natural habitat means testing in an often highly chaotic environment, including frequent disturbances and environmental variations. While we tried to account for this by excluding all subtests with coded disturbances in a second analysis, this did not improve ~~the~~ reliability. One reason might be our choice of coding disturbances only when the dog was visible and attending to the distraction for several seconds underrepresented the influence of the disturbance on the study subject~~that a disturbance was only noted when the dog was looking at it for several seconds and when the dog was visible on camera, but this was~~

likely an underestimation of the phenomenon since dogs could also be affected by sounds occurring during testing. Indeed, only six of the 35 dogs were alone in both tests (i.e., initial test and retest) while for all others, additional dogs entered the testing area throughout one or both of the testing occasions and had to be 'distracted away' by the second experimenter. Besides the probable influence of those disturbances on the overall retest reliability, they might explain why the subtests in which the dog had more time to focus on their environment without the direct engagement by the experimenter (fake dog, novel object, begging) showed a lower retest reliability. Whether behaviours like gazing and yawning and the entire begging test, which does not only lack interaction but also novelty, can reliably reflect a dog's behaviour under such circumstances is hence questionable and requires further rigorous testing in alternative test setups on the streets.

To address the disturbance problem on the streets, we propose four not mutually exclusive approaches: 1) Have at least one additional person on the team that is solely responsible to distract approaching dogs out of sight. 2) Allow for longer between-subtest-phases if the disturbance is temporary and let the dog return to an attentive state rather than pushing through the test. However, one should keep in mind that this changes the time dogs spend in the overall test procedure and carefully consider an influence on the respective goal. 3) If the distraction is substantial, terminating the test and either repeating or resuming the test on a different day might be an option, depending on the respective test set up, goal, and familiarity with the population. 4) Meticulously coding any distraction might allow for correction of the disturbances, or at least provide deeper understanding of their influence on the behaviour, based on which decisions for future testing can be made.

Even after considering the disturbance problemOn the other hand, the consistency of dogs' behaviour in the fake dog and novel object tests was notably low. This might seem particularly surprising since both tests have been regularly used in dog temperament assessments [e.g., 57, 82]. However, we could not find any reference to the retest reliability of fake dog tests, and studies exploring novel object tests and novelty-seeking and fearlessness traits in general showed that their test-retest reliability even in highly standardised settings is often poor to make a judgement about the quality of the subtests, we need to question whether

~~this was caused by the subtest's inability to capture the traits reliably or whether other factors were at play that impeded a repeated elicitation of the same behaviours. Indeed, the retest reliability of novelty-seeking and fearlessness traits is often low~~ (i.e., ICC<0.50) ~~–~~[e.g., Novelty-seeking: 0.48 in 34, Environmental Sureness: 0.16 in 81].~~,~~ Accordingly, ~~and~~ it has been proposed that test-retest outcomes are sensitive to the novelty of the object and situation due to learning effects [37]. Indeed, the fact that the free-ranging dogs explored the fake dog significantly more in the initial test while showing significantly less fear in the retest supports the assumption that the animals partially remembered the initial encounter and adapted their behaviour accordingly. ~~While~~ Although we made ~~an~~ efforts to modify the appearance~~change the look~~ of the fake dog and novel object for the retest, the situation, procedure and set-up ~~were still~~remained the same and seem to have lacked the novelty needed to "fool" the animals twice within just six weeks. The poor test-retest reliability of those two subtests was hence more likely due to learning effects rather than an inability to capture the traits themselves. Nevertheless, it needs to be stressed that the street dogs frequently displayed appropriate conspecific, startling, and investigation behaviour in the initial presentation. Additionally considering that those subtests have been validated with fake vs. real dogs in case of the fake dog test [57, 66] and deemed reliable despite similar retest problems in case of the novel object [e.g., 34, 37]~~,Additionally considering that the street dogs frequently displayed appropriate conspecific, startling, and investigation behaviour in the initial presentation, suggests that the subtests in themselves did indeed elicit the target traits. And since the personality tests we based these subtests on had similar retest problems but were still deemed reliable [e.g., 34, 37]~~, we suggest that these two subtests are suitable to test the street dogs. Particularly for the fake dog test however, the rather short interaction time most dogs showed with the fake dog suggests that a shorter exposure (i.e., test duration) or movement in the fake dog [e.g., 66] might have to be considered to decrease not only the distraction possibilities, but also the risk that neophobia and boldness traits replace the conspecific behaviours as soon as the dogs realize that the fake dog is not in fact real.

At the same time, these two subtests act as a reminder for the importance of appropriate between-test-intervals, particularly for test batteries investigating behavioural profiles. With free-ranging dogs, the

balance between waiting out potential learning effects and retesting early enough to find a considerable number of test subjects again can be difficult, especially if the study population is not well-known. Turnover rates can be highly variable between locations and while we were familiar with the dynamics in the surrounding area [51], we had not yet worked with this particular population of street dogs. In addition, more than half of our study subjects were retested in a different location. The substantial retesting rate of 72% within an average of six weeks was hence surprising, though the considerable variation between the individuals (min. 33, max. 76 days) has to be acknowledged. Meticulous notes on appearance, exact finding location, and particular behaviours were crucial in this effort, not only for retesting but also to avoid testing of the same dog as a different individual. While working with a well-known population would streamline such efforts, our results show that it is not a prerequisite. In addition, using fewer memorable cues (e.g., the V-shaped wooden board in the Fake dog and Novel object subtest) might reduce learning effects and allow for a shorter between-test-interval with better outcomes than presented here.

Another aspect to consider in relation to the low test-retest reliability of some of the subtests (fake dog, novel object, and begging test in particular) is the often highly chaotic environment in which animals were tested, including frequent disturbances and environmental variations. While we tried to account for this by excluding all subtests with coded disturbances in a second analysis, this did not improve the reliability. One reason might be that a disturbance was only noted when the dog was looking at it for several seconds and when the dog was visible on camera, but this was likely an underestimation of the phenomenon since dogs could also be affected by sounds occurring during testing. Indeed, only six of the 35 dogs were alone in both tests (i.e., initial test and retest) while for all others, additional dogs entered the testing area throughout one or both of the testing occasions and had to be 'distracted away' by the second experimenter. In addition, the fact that less than half of the dogs were tested and retested in the same location may also have played a role. The role of disruptions during the test might explain why predominantly human-directed and interactive variables and subtests (sociability, pointing, tractability) showed significant reliability over time while the subtests where the experimenters ignored and did not respond to the dog (fake dog, novel object and begging test) did not. In the latter cases, without the direct engagement by the experimenter, dogs may have been

more prone to the distractions occurring around them. Whether behaviours like gazing and yawning and the entire begging test can reliably reflect a dog's behaviour under such circumstances is hence questionable. Overall, the disturbance problem on the streets could be alleviated by testing populations that the experimenters are highly familiar with in less disturbed testing locations, prioritizing the distraction of on-coming dogs, and terminating a test to redo it on a different day in cases where distractions cannot be circumvented.

Beside these two probable explanations for the low test-retest reliability in some subtests, Aan additional – though highly speculative – explanation might be that free-ranging dogs could be less genetically fixed in some behavioural traits than modern breed dogs and thus behave less consistently in general. This idea might not be too farfetched, considering thatSince breed dogs have been selected for specific characteristics and have lost considerable genetic variation along the way [24], whereas free-ranging dogs are still subject to free mate choice and the instability of their environment [26]. Behavioural plasticity for certain traits (e.g., adaptiveness to the highly dynamic group compositions, weather conditions, or anthropogenic changes) might therefore be a much more useful and thus widely retained trait in free-ranging dogs to deal with the constant changes in their environment, while it might have been selected against in our modern breeds. , this idea might not be too farfetched. Indeed, the study of reaction norms in wild fish and birds has provided cautious evidence that behavioural plasticity (i.e., intra-individual variability) can have a heritable component [94, 95]. And though a meta-analysis targeting the question whether behaviour-selected working-breed dogs were more fixed, i.e. consistent in their behaviour, than non-selected non-working breed dogs could not find any support for this hypothesis [36], it was reported that an unstable rearing environment does not seem to have an effect on the consistency of behaviour, further highlighting the stability of certain traits in breed dogs [92]. Exploring how this compares to free-ranging dogs is an exciting avenue for further research.

Finally, many behaviours in the presented ethogram were only rarely or never displayed which did not allow testing their reliability. Since the majority of these were easily recognizable frequency behaviours, they could be expected to have a similar coding reliability to the other highly distinct variables in the ethogram

once they are recorded in higher frequencies in a bigger sample. However, we do concede that the fit with the ICC analysis might not have been ideal for the very infrequent, highly skewed variables. One step to alleviate this was to drop the variables with the highest skew (occurrence in less than 10% of all tests) for the test-retest reliability analysis. In addition, we argue that it is less about the exact reliability and rather about the overall pattern that emerges, with mainly frequent, human-directed, interactive variables showing reliability over time. We therefore decided to retain the ICC statistic since it was reliable enough for the purpose of this study, provides better comparability due to its wide-spread use, and is more convenient for datasets with many variables compared to less well-developed alternative methods. For the future exploration and correlations of those traits, on the other hand, and if frequencies of single behaviours remain low despite a bigger sample size, we suggest creating higher category variables or composite scales by summing behaviours that clearly represent an underlying concept, e.g., aggression would include lunging, baring teeth, growling etc. Such an approach has already been used in several studies on personality traits in dogs [e.g., 34, 96].

A last consideration regarding the test itself pertains to the exclusion of the play component in the Human approach test and what that teaches us about the need to consider your study subject in all its facets. Play is frequently used in temperament tests to assess sociability [35, 60, 65], and it has also been suggested as a potential part of the domestication syndrome in terms of neotenic behaviour [reviewed in 3, 97]. When applying this subtest with the street dogs, the low engagement rate and often fearful behaviour of the animal towards the toy might be explained by the lack of or negative experience such unowned free-ranging scavengers had with pulling on an object with a human [52]. In a similar incident, Lazzaroni, Range (28) found that free-ranging dogs' lower persistence in comparison to pet dogs was likely due to their need to preserve energy and a lack of experience with human-manipulated items. Alternatively, the recent findings that both ancient breeds [98] and wolf hybrids [99] also express lower levels of human-directed play behaviour than most modern breeds might suggest that a selection for human-directed play has only relatively recently taken place in modern breed dogs (e.g. as reward use in working dogs [100] or bonding-experience between dogs and humans [97]). In addition, our anecdotal observation with free-ranging

populations indicates multiple incidents of conspecific play, running after birds or balls seemingly for fun, and ‘playing’ with the bowl during the pointing test. Whether play does indeed persist into adulthood in free-ranging dogs in the conspecific context or solitary play, but not with a human partner, or whether our choice of method was just ill-considered, remains to explored and emphasizes how carefully the subject population, its ecology and life experience must be considered despite our extensive experience with the species itself.

Finally, there are two important limitations that need to be addressed in terms of free-ranging dog testing as well as the representativeness of this study. First, the consideration that testing adult free-ranging dogs must rely on voluntary participation creates a natural bias for more social animals, especially in longer and more interactive tests, and might be particularly challenging for feral populations (i.e., not reliant on human settlements). This bias is somewhat reduced in our Human approach test since the only selection criteria for starting a test was that the dog was seemingly alone. Thus at least the first steps of the test consisting of calling the dog and attempting a friendly approach were carried out with all dogs we found alone. If a dog then fearfully ran away as soon as we attempted the approach, this was analysed as part of our dataset. Although a minority, these dogs are part of our population and the fact that we were able to retest more than half of these ‘escaping’ individuals and they manifested the same behaviour on second testing provides evidence that this measurement was indeed reliable. Nevertheless, it is the case that overly fearful animals were lost for all the following subtests and are therefore not part of the majority of the reliability analysis. Similarly, [29] found in a testing effort with 160 street dogs that half of their testing subjects could be classified as fearful and that these dogs were significantly less likely to approach and choose in a pointing task than neutral and affiliative dogs. At the same time, any constraining of a free-ranging animal during a test would undoubtedly influence its behaviour to a point where it cannot be regarded as natural anymore. Explorable options to test such dogs could be the use of shorter tests with little human presence wherever possible (e.g., reaction- or exploration-based tests) or testing populations that now live in a fenced shelter but genetically belong to the clade of free-ranging dogs and had spent most of their lives roaming freely. However, with the latter option, whether the influence of life experience and current social contexts might

have an influence on the target behaviour has to be strongly considered [50]. Secondly, the fact that our study population has previously been recognized as exceptionally friendly [51] kept us from being able to analyse the reliability of aggressive behaviours and stress-related behaviours. For example, only 16.7% of our sample ran away during the sociability test and none showed aggression, while more than 50% of an Ethiopian village dog sample fled upon approach and 11% reacted aggressively (though one needs to consider that these dogs were not always tested alone or outside [56]). While expanding this test battery to other populations might be a way to start to characterize different free-ranging dog populations and what may affect differences in their behavioural profiles, we acknowledge that these limitations are likely difficult to be overcome.

Exhibition of extreme fear or the slightest sign of human-directed aggression will necessarily lead to aborting the test (particularly considering the risk of lethal disease, [e.g., 49]. And while using a fake dog rather than a real dog was indeed an attempt to test for the presence of aggression in a safe way, collecting complementary observational data (regarding both human and conspecific directed behaviour) of the same individuals may be a complementary method allowing for multiple measures of specific behavioural traits.

Taken together, the Domestication Syndrome test battery presented here has considerable potential to be used with free-ranging dogs and beyond to test predictions deriving from the DS. In conclusion, Tthe easy implementation, successful participation of most free-ranging dogs and the high coding reliability presented here confirmss that free-ranging dogs can be tested in longer test batteries with complex ethograms despite the challenging street conditions. The wide variability in the test-retest reliability across subtests demonstrate that the management of disturbances, the choice of set up and test-retest intervals, and careful considerations of the study subjects' life experience and ecology are central challenges behavioural tests with free-ranging dogs in their natural habitat face. At the same time, considerable reliability in some of the subtest demonstrates that under well-chosen conditions, testing street dogs is possible and opens a vast array of intriguing pathways to explore [50].While the overall test-retest reliability was low, the interactive human-directed variables demonstrated acceptable consistency over time, in particular in the sociability, pointing, and tractability subtest. This might suggest that the remaining variables were more disturbance-

prone. Finally, we argue that the low test-retest reliability in the fake dog and novel object subtest did not adequately reflect the tests' ability to capture the target traits but was rather due to habituation effects after a short between-test period, and these subtests can therefore be used as such going forward. Bearing the suggestions made in the discussion in mind, going forward the test battery presented here can be a valuable method to test questions about cognitive traits, behavioural profiles, and domestication in such an understudied population as free-ranging dogs. the Domestication Syndrome test battery can be a valuable method to test the target behaviours in such an understudied population as free-ranging dogs, and to assess the controversial validity of the domestication syndrome as well as other dog domestication hypotheses.

Acknowledgements

We thank Professor Ikhlass El Berbri for her support and collaboration in this project. Additionally, we thank the rest of our team in Morocco (in particular Andreas Berghänel, Haytem Bouchri, Manon Delaunay and Magdalena Juskaite) without whom the project would not have been possible. We are also grateful to the population and authorities in the Sous-Massa region for supporting our field work. Finally, we want to extend our gratitude to the two reviewers for their valuable feedback and suggestions.

Bibliography

1. Gepts P, Papa R. Evolution during domestication. e LS. 2001. doi: https://doi.org/10.1038/npg.els.0003071.
2. Darwin C. The Variation of Animals and Plants Under Domestication. London1868.
3. Kaiser S, Hennessy MB, Sachser N. Domestication affects the structure, development and stability of biobehavioural profiles. Frontiers in Zoology. 2015;12(1):1-11. doi: https://doi.org/10.1186/1742-9994-12-S1-S19.
4. Price EO. Behavioral aspects of animal domestication. The quarterly review of biology. 1984;59(1):1-32.
5. Trut LN, Oskina I, Kharlamova A. Animal evolution during domestication: the domesticated fox as a model. Bioessays. 2009;31(3):349-60. doi: https://doi.org/10.1002/bies.200800070.
6. Albert FW, Shchepina O, Winter C, Römpler H, Teupser D, Palme R, et al. Phenotypic differences in behavior, physiology and neurochemistry between rats selected for tameness and for defensive aggression towards humans. Hormones and behavior. 2008;53(3):413-21. doi: https://doi.org/10.1016/j.yhbeh.2007.11.010.
7. Belyaev DK. Destabilizing selection as a factor in domestication. Journal of Heredity. 1979;70(5):301-8.

8. Trut LN. Early Canid Domestication: The Farm-Fox Experiment: Foxes bred for tamability in a 40-year experiment exhibit remarkable transformations that suggest an interplay between behavioral genetics and development. American Scientist. 1999;87(2):160-9.
9. Wilkins AS, Wrangham RW, Fitch WT. The “domestication syndrome” in mammals: a unified explanation based on neural crest cell behavior and genetics. Genetics. 2014;197(3):795-808. doi: https://doi.org/10.1534/genetics.114.165423.
10. Lord KA, Larson G, Coppinger RP, Karlsson EK. The history of farm foxes undermines the animal domestication syndrome. Trends in ecology & evolution. 2020;35(2):125-36. doi: https://doi.org/10.1016/j.tree.2019.10.011.
11. Sánchez-Villagra MR, Geiger M, Schneider RA. The taming of the neural crest: a developmental perspective on the origins of morphological covariation in domesticated mammals. Royal Society open science. 2016;3(6):160107. doi: https://doi.org/10.1098/rsos.160107.
12. Sánchez-Villagra MR, Segura V, Geiger M, Heck L, Veitschegger K, Flores D. On the lack of a universal pattern associated with mammalian domestication: differences in skull growth trajectories across phylogeny. Royal Society open science. 2017;4(10):170876. doi: https://doi.org/10.1098/rsos.170876.
13. Johnsson M, Henriksen R, Wright D. The neural crest cell hypothesis: no unified explanation for domestication. Genetics. 2021;219(1). doi: https://doi.org/10.1093/genetics/iyab097.
14. Larson G, Fuller DQ. The evolution of animal domestication. Annual Review of Ecology, Evolution, and Systematics. 2014;45:115-36. doi: https://doi.org/10.1146/annurev-ecolsys-110512-135813.
15. Wirobski G, Range F, Schaebs F, Palme R, Deschner T, Marshall-Pescini S. Endocrine changes related to dog domestication: Comparing urinary cortisol and oxytocin in hand-raised, pack-living dogs and wolves. Hormones and Behavior. 2021;128:104901. doi: https://doi.org/10.1016/j.yhbeh.2020.104901.
16. Hansen Wheat C, van der Bijl W, Wheat CW. Morphology does not covary with predicted behavioral correlations of the domestication syndrome in dogs. Evolution letters. 2020;4(3):189-99. doi: https://doi.org/10.1002/evl3.168.
17. von Holdt BM, Shuldiner E, Koch IJ, Kartzinel RY, Hogan A, Brubaker L, et al. Structural variants in genes associated with human Williams-Beuren syndrome underlie stereotypical hypersociability in domestic dogs. Science advances. 2017;3(7):e1700398. doi: https://doi.org/10.1126/sciadv.1700398.
18. Range F, Marshall-Pescini S, Kratz C, Virányi Z. Wolves lead and dogs follow, but they both cooperate with humans. Scientific reports. 2019;9(1):1-10. doi: https://doi.org/10.1038/s41598-019-40468-y.
19. Hare B, Tomasello M. Human-like social skills in dogs? Trends in cognitive sciences. 2005;9(9):439-44. doi: https://doi.org/10.1016/j.tics.2005.07.003.
20. Range F, Marshall-Pescini S. Comparing wolves and dogs: current status and implications for human ‘self-domestication’. Trends in Cognitive Sciences. 2022. doi: https://doi.org/10.1016/j.tics.2022.01.003.
21. Moretti L, Hentrup M, Kotrschal K, Range F. The influence of relationships on neophobia and exploration in wolves and dogs. Animal Behaviour. 2015;107:159-73. doi: https://doi.org/10.1016/j.anbehav.2015.06.008.
22. Wheat CH, Temrin H. Intrinsic ball retrieving in wolf puppies suggests standing ancestral variation for human-directed play behavior. Iscience. 2020;23(2):100811. doi: https://doi.org/10.1016/j.isci.2019.100811.
23. Hansen Wheat C, Fitzpatrick JL, Rogell B, Temrin H. Behavioural correlations of the domestication syndrome are decoupled in modern dog breeds. Nature communications. 2019;10(1):1-9. doi: https://doi.org/10.1038/s41467-019-10426-3.
24. Gray MM, Granka JM, Bustamante CD, Sutter NB, Boyko AR, Zhu L, et al. Linkage disequilibrium and demographic history of wild and domestic canids. Genetics. 2009;181(4):1493-505. doi: https://doi.org/10.1534/genetics.108.098830.

25. Pilot M, Malewski T, Moura AE, Grzybowski T, Oleński K, Kamiński S, et al. Diversifying selection between pure-breed and free-breeding dogs inferred from genome-wide SNP analysis. G3: Genes, Genomes, Genetics. 2016;6(8):2285-98. doi: https://doi.org/10.1534/g3.116.029678.

26. Pilot M, Malewski T, Moura AE, Grzybowski T, Oleński K, Ruść A, et al. On the origin of mongrels: evolutionary history of free-breeding dogs in Eurasia. Proceedings of the Royal Society B: Biological Sciences. 2015;282(1820):20152189. doi: https://doi.org/10.1098/rspb.2015.2189.

27. Brubaker L, Dasgupta S, Bhattacharjee D, Bhadra A, Udell MA. Differences in problem-solving between canid populations: Do domestication and lifetime experience affect persistence? Animal Cognition. 2017;20(4):717-23. doi: https://doi.org/10.1007/s10071-017-1093-7.

28. Lazzaroni M, Range F, Bernasconi L, Darc L, Holtsch M, Massimei R, et al. The role of life experience in affecting persistence: A comparative study between free-ranging dogs, pet dogs and captive pack dogs. PLOS ONE. 2019;14(4):e0214806. doi: https://doi.org/10.1371/journal.pone.0214806.

29. Bhattacharjee D, Mandal S, Shit P, Varghese MG, Vishnoi A, Bhadra A. Free-ranging dogs are capable of utilizing complex human pointing cues. Frontiers in Psychology. 2020;10:2818. doi: https://doi.org/10.3389/fpsyg.2019.02818.

30. Taylor KD, Mills DS. The development and assessment of temperament tests for adult companion dogs. Journal of Veterinary Behavior. 2006;1(3):94-108. doi: https://doi.org/10.1016/j.jveb.2006.09.002.

31. Patronek GJ, Bradley J, Arps E. What is the evidence for reliability and validity of behavior evaluations for shelter dogs? A prequel to “No better than flipping a coin”. Journal of Veterinary Behavior. 2019;31:43-58. doi: https://doi.org/10.1016/j.jveb.2019.03.001.

32. Koo TK, Li MY. A guideline of selecting and reporting intraclass correlation coefficients for reliability research. Journal of chiropractic medicine. 2016;15(2):155-63. doi: https://doi.org/10.1016/j.jcm.2016.02.012.

33. Gartner MC. Pet personality: A review. Personality and Individual Differences. 2015;75:102-13. doi: https://doi.org/10.1016/j.paid.2014.10.042.

34. Turcsán B, Wallis L, Virányi Z, Range F, Müller CA, Huber L, et al. Personality traits in companion dogs—Results from the VIDOPET. PLoS One. 2018;13(4):e0195448. doi: https://doi.org/10.1371/journal.pone.0195448.

35. Valsecchi P, Barnard S, Stefanini C, Normando S. Temperament test for re-homed dogs validated through direct behavioral observation in shelter and home environment. Journal of Veterinary Behavior. 2011;6(3):161-77. doi: https://doi.org/10.1016/j.jveb.2011.01.002.

36. Fratkin JL, Sinn DL, Patall EA, Gosling SD. Personality consistency in dogs: a meta-analysis. PloS one. 2013;8(1):e54907. doi: https://doi.org/10.1371/journal.pone.0054907.

37. Svartberg K, Tapper I, Temrin H, Radesäter T, Thorman S. Consistency of personality traits in dogs. Animal Behaviour. 2005;69(2):283-91. doi: https://doi.org/10.1016/j.anbehav.2004.04.011.

38. Darwin C. The descent of Man; and on Selection in relation to Sex: J. Murray; 1871.

39. Pavlov IP. The scientific investigation of the psychical faculties or processes in the higher animals. Science. 1906;24(620):613-9.

40. Udell MA, Wynne CD. A review of domestic dogs'(Canis familiaris) human-like behaviors: or why behavior analysts should stop worrying and love their dogs. Journal of the experimental analysis of behavior. 2008;89(2):247-61.

41. Bensky MK, Gosling SD, Sinn DL. The world from a dog’s point of view: a review and synthesis of dog cognition research. Advances in the Study of Behavior. 2013;45:209-406.

42. Creevy KE, Akey JM, Kaeberlein M, Promislow DE. An open science study of ageing in companion dogs. Nature. 2022;602(7895):51-7.

43. Range F, Marshall-Pescini S. Domestication Hypotheses Relating to Behaviour and Cognition: Which Are Supported by the Current Data? Wolves and Dogs: Springer; 2022. p. 335-73.

44. Hughes J, Macdonald DW. A review of the interactions between free-roaming domestic dogs and wildlife. Biological conservation. 2013;157:341-51.

45. Lord K, Feinstein M, Smith B, Coppinger R. Variation in reproductive traits of members of the genus Canis with special attention to the domestic dog (Canis familiaris). Behavioural processes. 2013;92:131-42.

46. Bonanni R, Cafazzo S. The social organisation of a population of free-ranging dogs in a suburban area of Rome: a reassessment of the effects of domestication on dogs' behaviour. The social dog: Elsevier; 2014. p. 65-104.

47. Cafazzo S, Valsecchi P, Bonanni R, Natoli E. Dominance in relation to age, sex, and competitive contexts in a group of free-ranging domestic dogs. Behavioral Ecology. 2010;21(3):443-55. doi: https://doi.org/10.1093/beheco/arq001.

48. Range F, Marshall-Pescini S. Wolves and Dogs: Between Myth and Science: Springer Nature; 2022.

49. Hampson K, Coudeville L, Lembo T, Sambo M, Kieffer A, Attlan M, et al. Estimating the global burden of endemic canine rabies. PLoS neglected tropical diseases. 2015;9(4):e0003709.

50. Horn L, Cimarelli G, Boucherie PH, Šlipogor V, Bugnyar T. Beyond the dichotomy between field and lab—the importance of studying cognition in context. Current Opinion in Behavioral Sciences. 2022;46:101172.

51. Lazzaroni M, Marshall-Pescini S. Der Streuner- das fehlende Bindeglied. In: Kitchenham K, editor. Streuner Hunde: Von Moskaus U-Bahn-Hunden bis Indiens Underdogs: Komsos; 2020.

52. Paul MM, Sreejani Sen; Sau, Shubhra; Nandi, Anjan K.; Bhadra, Anindita. High early life mortality in free-ranging dogs is largely influenced by humans. Sci Rep. 2016;6(19641). doi: https://doi.org/10.1038/srep19641.

53. Vanak AT, Gompper ME. Dogs Canis familiaris as carnivores: their role and function in intraguild competition. Mammal review. 2009;39(4):265-83. doi: https://doi.org/10.1111/j.1365-2907.2009.00148.x.

54. Kato M, Yamamoto H, Inukai Y, Kira S. Survey of the stray dog population and the health education program on the prevention of dog bites and dog-acquired infections: a comparative study in Nepal and Okayama Prefecture, Japan. Acta Medica Okayama. 2003;57(5):261-6.

55. Bhattacharjee D, Sau S, Das J, Bhadra A. Free-ranging dogs prefer petting over food in repeated interactions with unfamiliar humans. Journal of Experimental Biology. 2017;220(24):4654-60. doi: https://doi.org/10.1242/jeb.166371.

56. Ortolani A, Vernooij H, Coppinger R. Ethiopian village dogs: Behavioural responses to a stranger's approach. Applied Animal Behaviour Science. 2009;119(3-4):210-8.

57. Barnard S, Kennedy D, Watson R, Valsecchi P, Arnott G. Revisiting a previously validated temperament test in shelter dogs, including an examination of the use of fake model dogs to assess conspecific sociability. Animals. 2019;9(10):835. doi: https://doi.org/10.3390/ani9100835.

58. Heberlein MT, Turner DC, Range F, Virányi Z. A comparison between wolves, Canis lupus, and dogs, Canis familiaris, in showing behaviour towards humans. Animal Behaviour. 2016;122:59-66. doi: https://doi.org/10.1016/j.anbehav.2016.09.023.

59. Mendes JWW, Resende B, Savalli C. A review of the unsolvable task in dog communication and cognition: comparing different methodologies. Animal Cognition. 2021;24(5):907-22. doi: https://doi.org/10.1007/s10071-021-01501-8.

60. Svartberg K. The hierarchical structure of dog personality in a new behavioural assessment: A validation approach. Applied Animal Behaviour Science. 2021;238:105302. doi: https://doi.org/10.1016/j.applanim.2021.105302.

61. Barnard S, Marshall-Pescini S, Passalacqua C, Beghelli V, Capra A, Normando S, et al. Does Subjective Rating Reflect Behavioural Coding? Personality in 2 Month-Old Dog Puppies: An Open-Field Test and Adjective-Based Questionnaire. PLOS ONE. 2016;11(3):e0149831. doi: https://doi.org/10.1371/journal.pone.0149831.

Formatted: German (Austria)

62. Brady K, Hewison L, Wright H, Zulch H, Cracknell N, Mills D. A spatial discounting test to assess impulsivity in dogs. Applied Animal Behaviour Science. 2018;202:77-84. doi: https://doi.org/10.1016/j.applanim.2018.01.003.
63. Plutchik R. Individual and breed differences in approach and withdrawal in dogs. Behaviour. 1971;40(3-4):302-11.
64. Wright HF, Mills DS, Pollux PM. Behavioural and physiological correlates of impulsivity in the domestic dog (Canis familiaris). Physiology & behavior. 2012;105(3):676-82. doi: https://doi.org/10.1016/j.physbeh.2011.09.019.
65. Svartberg K, Forkman B. Personality traits in the domestic dog (Canis familiaris). Applied animal behaviour science. 2002;79(2):133-55. doi: https://doi.org/10.1016/S0168-1591(02)00121-1.
66. Reid PJ, Cussen VA, Collins LK, Lockwood R. The Utility of Model Dogs for Assessing Conspecific Aggression in Fighting Dogs. Applied Animal Behaviour Science. 2022:105710. doi: https://doi.org/10.1016/j.applanim.2022.105710.
67. Krause MA, Udell MA, Leavens DA, Skopos L. Animal pointing: Changing trends and findings from 30 years of research. Journal of Comparative Psychology. 2018;132(3):326. doi: https://doi.org/10.1037/com0000125.
68. Mendes JWW, Resende B, Savalli C. A review of the unsolvable task in dog communication and cognition: comparing different methodologies. Animal Cognition. 2021:1-16. doi: https://doi.org/10.1007/s10071-021-01501-8.
69. Marshall-Pescini S, Rao A, Virányi Z, Range F. The role of domestication and experience in 'looking back'towards humans in an unsolvable task. Scientific reports. 2017;7(1):46636.
70. Lazzaroni M, Marshall-Pescini S, Manzenreiter H, Gosch S, Přibilová L, Darc L, et al. Why do dogs look back at the human in an impossible task? Looking back behaviour may be over-interpreted. Animal Cognition. 2020;23(3):427-41.
71. Miklósi Á, Kubinyi E, Topál J, Gácsi M, Virányi Z, Csányi V. A simple reason for a big difference: wolves do not look back at humans, but dogs do. Current biology. 2003;13(9):763-6. doi: https://doi.org/10.1016/S0960-9822(03)00263-X.
72. D'Aniello B, Scandurra A. Ontogenetic effects on gazing behaviour: a case study of kennel dogs (Labrador Retrievers) in the impossible task paradigm. Animal cognition. 2016;19:565-70.
73. Cafazzo S, Marshall-Pescini S, Lazzaroni M, Virányi Z, Range F. The effect of domestication on post-conflict management: Wolves reconcile while dogs avoid each other. Royal Society Open Science. 2018;5(7):171553. doi: https://doi.org/10.1098/rsos.171553.
74. Cimarelli G, Marshall-Pescini S, Range F, Berghänel A, Virányi Z. Relationship quality affects social stress buffering in dogs and wolves. Animal Behaviour. 2021;178:127-40. doi: https://doi.org/10.1016/j.anbehav.2021.06.008.
75. Bonanni R, Cafazzo S, Valsecchi P, Natoli E. Effect of affiliative and agonistic relationships on leadership behaviour in free-ranging dogs. Animal Behaviour. 2010;79(5):981-91. doi: https://doi.org/10.1016/j.anbehav.2010.02.021.
76. Pompey KT. A Unified Approach to Estimating the Intraclass Correlation Coefficient and Its Bias: an Exploratory Study: University of South Carolina; 2021.
77. Boström AF, Hyytiäinen HK, Koho P, Cizinauskas S, Hielm-Björkman AK. Development of the Finnish neurological function testing battery for dogs and its intra-and inter-rater reliability. Acta Veterinaria Scandinavica. 2018;60(1):1-9. doi: https://doi.org/10.1186/s13028-018-0408-2.
78. R Core Team. R: A Language and Environment for Statistical Computing. R Foundation for Statistical Computing; 2019.
79. Shrout PE, Fleiss JL. Intraclass correlations: uses in assessing rater reliability. Psychological bulletin. 1979;86(2):420. doi: https://doi.org/10.1037/0033-2909.86.2.420.
80. Harvey ND. A Simple Guide to Inter-rater, Intra-rater and Test-retest Reliability for Animal Behaviour Studies. 2021. doi: https://doi.org/10.31219/osf.io/8stpy.

81. Sinn DL, Gosling SD, Hilliard S. Personality and performance in military working dogs: Reliability and predictive validity of behavioral tests. Applied Animal Behaviour Science. 2010;127(1-2):51-65. doi: https://doi.org/10.1016/j.applanim.2010.08.007.

82. van der Borg JA, Beerda B, Ooms M, de Souza AS, van Hagen M, Kemp B. Evaluation of behaviour testing for human directed aggression in dogs. Applied Animal Behaviour Science. 2010;128(1-4):78-90. doi: https://doi.org/10.1016/j.applanim.2010.09.016.

83. Fleiss JL, Cohen J. The equivalence of weighted kappa and the intraclass correlation coefficient as measures of reliability. Educational and psychological measurement. 1973;33(3):613-9. doi: https://doi.org/10.1177/001316447303300309.

84. Klausz B, Kis A, Persa E, Miklósi Á, Gácsi M. A quick assessment tool for human-directed aggression in pet dogs. Aggressive behavior. 2014;40(2):178-88. doi: https://doi.org/10.1002/ab.21501.

85. Beerda B, Schilder MB, van Hooff JA, de Vries HW. Manifestations of chronic and acute stress in dogs. Applied Animal Behaviour Science. 1997;52(3-4):307-19.

86. Pedretti G, Canori C, Biffi E, Marshall-Pescini S, Valsecchi P. Appeasement function of displacement behaviours? Dogs' behavioural displays exhibited towards threatening and neutral humans. Animal Cognition. 2023:1-10. doi: https://doi.org/10.1007/s10071-023-01742-9.

87. Lazzaroni M, Range F, Backes J, Portele K, Scheck K, Marshall-Pescini S. The Effect of Domestication and Experience on the Social Interaction of Dogs and Wolves With a Human Companion. Frontiers in Psychology. 2020;11. doi: https://doi.org/10.3389/fpsyg.2020.00785.

88. Rayment DJ, De Groef B, Peters RA, Marston LC. Applied personality assessment in domestic dogs: Limitations and caveats. Applied Animal Behaviour Science. 2015;163:1-18. doi: https://doi.org/10.1016/j.applanim.2014.11.020.

89. Wan M, Bolger N, Champagne FA. Human perception of fear in dogs varies according to experience with dogs. PLoS one. 2012;7(12):e51775. doi: https://doi.org/10.1371/journal.pone.0051775.

90. Epperlein T, Kovacs G, Oña LS, Amici F, Bräuer J. Context and prediction matter for the interpretation of social interactions across species. Plos one. 2022;17(12):e0277783. doi: https://doi.org/10.1371/journal.pone.0277783.

91. Morrill K, Hekman J, Li X, McClure J, Logan B, Goodman L, et al. Ancestry-inclusive dog genomics challenges popular breed stereotypes. Science. 2022;17(6592):e0274383. doi: https://doi.org/10.1126/science.abk0639.

92. Gácsi M, Kara E, Belényi B, Topál J, Miklósi Á. The effect of development and individual differences in pointing comprehension of dogs. Animal cognition. 2009;12(3):471-9. doi: https://doi.org/10.1007/s10071-008-0208-6.

93. Bray EE, Gnanadesikan GE, Horschler DJ, Levy KM, Kennedy BS, Famula TR, et al. Early-emerging and highly heritable sensitivity to human communication in dogs. Current Biology. 2021;31(14):3132-6. e5. doi: https://doi.org/10.1016/j.cub.2021.04.055.

94. Dingemanse NJ, Barber I, Wright J, Brommer J. Quantitative genetics of behavioural reaction norms: genetic correlations between personality and behavioural plasticity vary across stickleback populations. Journal of evolutionary biology. 2012;25(3):485-96. doi: https://doi.org/10.1111/j.1420-9101.2011.02439.x.

95. Araya-Ajoy YG, Dingemanse NJ. Repeatability, heritability, and age-dependence of seasonal plasticity in aggressiveness in a wild passerine bird. Journal of Animal Ecology. 2017;86(2):227-38. doi: https://doi.org/10.1111/1365-2656.12621.

96. Sherwell E-G, Panteli E, Krulik T, Dilley A, Root-Gutteridge H, Mills DS. Changes in Dog Behaviour Associated with the COVID-19 Lockdown, Pre-Existing Separation-Related Problems and Alterations in Owner Behaviour. Veterinary Sciences. 2023;10(3):195. doi: https://doi.org/10.3390/vetsci10030195.

97. Bradshaw JW, Pullen AJ, Rooney NJ. Why do adult dogs 'play'? Behavioural processes. 2015;110:82-7. doi: https://doi.org/10.1016/j.beproc.2014.09.023.

98. Kolm N, Temrin H, Miklósi Á, Kubinyi E, Garamszegi LZ. The link between selection for function and human-directed play behaviour in dogs. Biology letters. 2020;16(9):20200366. doi: https://doi.org/10.1098/rsbl.2020.0366.
99. Hansen Wheat C, Fitzpatrick J, Tapper I, Temrin H. Wolf (Canis lupus) hybrids highlight the importance of human-directed play behavior during domestication of dogs (Canis familiaris). Journal of Comparative Psychology. 2018;132(4):373. doi: https://doi.org/10.1037/com0000119.
100. Brownell D. The Brownell-Marsolais scale: a proposal for the qualitative evaluation of SAR/disaster K9 candidates. Adv Rescue Technol. 2002;5:57-67.

Supplementary Information

S1 Exemplary video showing the different subtests of the test battery

S2 Equipment information

S23 Table. Detailed Ethogram. The behaviours are presented by category if applicable, with a detailed description and the unit in cursive in which they were measured. In addition, it is indicated in which subtest(s) the behaviour was coded and towards which modifier(s). Subtests include the Sociability (S), Fake dog (FD), Novel object (NO), Pointing (P), Begging (B), and Tractability (T) subtest. Modifiers are the main experimenter (E1), both experimenters (E1+2), the fake dog (FD), the novel object (NO), the bowl, and the stool. If the behaviour was coded in the test in general, it was marked as ‘yes’. If the behaviour was not coded in the subtest, it was indicated with a minus (-).

S43 Table. Detailed inter- and intra-rater reliability results. Displayed are the ICC for the inter- and intra-rater analysis for each behaviour variable and modifier summed across subtests. The lower and upper 95% confidence interval (CI), F-value (F), degreed of freedom (df), and p-value are presented. The ICC analysis was based on a two-way random effects ANOVA with an absolute agreement estimate. The ICC-target for acceptable coding reliability was 0.7. Moderate reliability below the target (i.e., 0.5<ICC<0.7) is emphasized with a light grey background, poor reliability (ICC<0.5) with a dark grey background. Variables that did not occur in the coded videos are indicated with NA.

S54 Table. Detailed test-retest reliability outcome (n = 26). Displayed are the ICCs for the test-retest reliability for each subtest, variable, and modifier for all tests and for only the ones where no disturbance was coded. The lower and upper 95% confidence interval (CI), F-value (F), degreed of freedom (df), and p-value are presented. Variables that were not significant are indicated with a grey background. The ICC analysis was based on a two-way mixed effects ANOVA with an absolute agreement estimate. Variables that were excluded in the first validation step (i.e., that did not occur in more than 10% of all testing occasions or had a bad inter- and intra-rater reliability) were not analysed for their test-retest reliability.

Additional Editor Comments:

Dear Authors
Your manuscript has been assessed and serious flaws in the study design, DS concept and grammatical errors have been highlighted. Kindly and thoroughly address the issued raised by the reviews and resubmit for another assessment.
Thank you

We are very grateful for the reviewers' detailed clarifications and suggestions, as well as their encouraging words and their concern with the description of the domestication-related focus. We agree with the suggestion that changing the focus of this paper to free-ranging dog testing overall provides more benefit for the scientific community and the reader. Following both reviewers' suggestion, we have rewritten the paper by dropping the domestication syndrome constraints and refocussing on the merits this paper has.

When submitting your revision, we need you to address these additional requirements.

1. Please ensure that your manuscript meets PLOS ONE's style requirements, including those for file naming.
   The requirements have been rechecked and implemented. Particularly file naming for figures and supplementary material has been corrected.
2. Please include a complete copy of PLOS' questionnaire on inclusivity in global research in your revised manuscript.
   The requested file has been uploaded.
3. We note that Figures 2 and 3 in your submission contain copyrighted images. We require you to either (1) present written permission from the copyright holder to publish these figures specifically under the CC BY 4.0 license, or (2) remove the figures from your submission.
   Figure 2 has been remade to only contain MS PowerPoint pictograms which are free to use. Figure 3 has been removed and links to the specific manufacturers have been included in the supplementary information S2 in case visual information is desired.

Reviewer 1

Reviewer #1: GENERAL COMMENTS:
Let me start with saying that I am very happy to see a behaviour study on free-ranging dogs. However, I cannot recommend this study for publication in its current form for several reasons, the main one being that this is not a study of the domestication syndrome and I find its current presentation and narrative to be quite misleading in all honesty.
It is very unclear to me why the authors have chosen to present their manuscript so elaborate around the domestication syndrome when they a) do not confirm the presence

of the domestication syndrome in the dog population (there is no test of whether the alleged domestication syndrome behaviours are in fact correlated – a central assumption of the hypothesis), and b) find that their so-called test battery fails to adequately quantify the behaviours they claim to be associated with the domestication syndrome. The eagerness to label their test battery the "Domestication Syndrome test battery" and claim that it is useful going forward in testing domestication syndrome behaviours, in spite of the very results they bring forward, therefore seem very misplaced and quite frankly puzzling. This is especially emphasized by the fact that half of this test battery includes behaviours that cannot in a meaningful way be associated with the domestication syndrome – a point the authors themselves hint at by mentioning that they will also test other domestication hypotheses with this test battery. These other behaviours, as compared to the claimed domestication syndrome behaviours, are even the only behaviours that with some confidence seem to be feasible and reliable to test in a free-ranging population. Why then label it the "Domestication Syndrome test battery"? It is simply misleading.
That being said, and while I understand that the above is a strong critique of the study, I think there is value in the study from the perspective that it is, in my opinion, actually a methods paper and it would benefit immensely from being rewritten as such. The work the authors have done in examining the feasibility and reliability in conducting in-situ behavioural testing of free-ranging dogs does have merit. So, instead of overselling this study by pinning it on a narrative that it simply cannot sustain, my strong recommendation for this manuscript is therefore that it undergoes major revision in order to be restructured to bring forward the valuable contributions it actually has in terms of the insights on general (not domestication syndrome) behavioural testing of free-ranging dogs. Highlighting the challenges of taking behavioural testing outside controlled laboratory settings and emphasize how this is a necessary next step in moving the field forward is valuable enough on its own.

GENERAL ANSWER to Reviewer #1:

Thank you for your valuable perspective and detailed concerns regarding the approach we had chosen to present this study in. The paper was indeed supposed to be a methods paper and we regret that it did not come across as such. Taking your suggestions to heart, it has been entirely rewritten, focusing on the necessity of testing free-ranging populations, how the presented test battery fits into that, and what conclusions can be drawn from our results about the possibilities and limitations of street dog testing. This pertains particularly to the introduction and partially to the discussion, where we have additionally addressed issues you raised with the methodology. The methods and results section have stayed largely the same besides clarifying specific issues you have pointed out, and renaming the Sociability test as Human approach test to clarify that all behaviours were tested. We hope that by refocussing this paper on the merits it has in terms of moving the field forward, this version is more to your satisfaction.

DETAILED COMMENTS:
Line 29: The authors need to remove the physiological reference here. Even though hair samples were collected, they were not analyzed and we have no way of knowing if a) this method is reliable in obtaining physiological parameters, and b) if these correlate with

the behavioural traits in the domestication syndrome. This reference is there for misleading in this context.

With the rewriting of the abstract, this line was taken out.

Line 53: Some clarity is needed here. Selection on tameness is not the mechanism per se. Tameness is likely the behavioural trait that was selected for during initial animal domestication cascading the phenotypic changes as seen in the domestication syndrome. However, the underlying mechanism causing the presumed correlated traits of the domestication syndrome remains unknown, but has been proposed to be neural crest deficit (Wilkins et al. 2016) or attenuated ontogenesis (Balyaev 1979).

As expressed above, we want to thank you for your clarifications and suggestions particularly in terms of refocusing the paper. Since the introduction has been entirely rewritten in this effort, many of the issues you have pointed out in this comment and below have been erased from the text. Whenever this is the case, we will mark it with "erased due to focus shift".

Line 58: What are all the morphological domestication syndrome-associated traits? Details are generally very sparse and the background is too superficial.

"erased due to focus shift"

Line 59: What physiological traits? Which traits are part of the DS and which do not fit with the hypothesis in dogs?

"erased due to focus shift"

Line 60-61: Which behavioural traits? Which traits are part of the DS and which do not fit with the hypothesis in dogs?

"erased due to focus shift"

Line 61- 66: This part has me a bit confused. It seems more like an afterthought and I am not quite sure how it fits in with the narrative. Are the authors saying that the domestication syndrome is not true in dogs or that it is expanded just in dogs with these extra features?

"erased due to focus shift"

Line 64: I am quite surprised to see the uncritical and sole use of this outdated reference (Hare and Tomasello 2005), given the long-standing debate (of +20 years) on the topic and the not so few studies refuting the uniqueness in dogs being able to follow human communicative clues. Udell et al. (2008) demonstrated that wolves outperformed dogs in a pointing test when tested under identical conditions. Likewise, Wheat et al. (2022) reanalyzed previously published data claiming that dog puppies have early emerging skills in following human pointing whereas wolves do not, but upon adequately controlling for the puppies' different environmental exposure dogs and wolves performed equally well. Furthermore, it has been demonstrated that close to 30 species (both wild and domesticated, e.g. elephants, bats, goats, pigs and not the least wolves)

across eight taxonomic groups have the ability to follow pointing gestures (see Krause et al. 2018 for review).

“erased due to focus shift”. The reference still appears in the manuscript but only as a reference to Hare’s emotional reactivity hypothesis. In addition, we want to thank you for pointing us to this useful reference. We included Krause’s observations in line 214.

Line 73-82: While I agree that it is problematic that some studies do not address the behavioural traits of the domestication syndrome in concert, I am puzzled by the argument that standardized test batteries designed to quantify behaviours, of which some are the basic behaviours of the domestication syndrome, should somehow be unfit for hypothesis testing. The argumentation brought forward by the authors here becomes quite ironic as they a) do not test all the behavioural traits of the domestication syndrome, and b) do not test the covariance between the behaviours they do test. There is thus no actual confirmation of the domestication syndrome in their free-ranging dog population, given one would accept the premise that the behaviours chosen are in fact domestication syndrome behaviours.

“erased due to focus shift”. In addition, a) is a very fair argument and a major reason why we agreed to drop the Domestication Syndrome narrative. For point b), analyses might follow in the future if the test can be established reliably on the streets, which was the main focus of this paper.

Line 83-94: I generally agree with this paragraph. However, it is too much of a blanket statement that free-ranging dogs in general is not admixed with breed dogs. For instance, Boyko et al. has a paper from 2009 showing that more than 60% of African village dogs could be classified as non-native, i.e. with a potential admixture with modern breeds. This does not mean that free-ranging dogs are not better models for these questions than modern breeds, but introgression with modern breeds must be assumed in free-breeding populations like this.

Thank you for this useful reference, it was a pleasure to read. Boyko et al. (2009) do indeed state that village dogs in some areas of Africa (esp. Namibia) show a high admixture rate while this admixture was low or non-existent in the other 84% of their sample. We have softened our statement (see line 84ff) to indicate that the “the majority of free-ranging dog populations are not a mixture of breeds but rather genetically distinct populations that predate the relatively more recent artificial selection and breed formation”, and refer to Boyko et al. (2009).

Line 100: I am starting to get a bit confused. Are the authors testing personality, the domestication syndrome or alterative domestication hypotheses?

“erased due to focus shift”

Line 103-122: My confusion grows. Now it sounds more like a methods paper…

That is indeed what it was supposed to be. We hope this version is more to your satisfaction.

Line 124-126: If the battery test all these different hypotheses (which I do not believe it does, for instance there is no test of correlation between alleged domestication

syndrome traits and the majority of domestication syndrome behaviours could not be adequately quantified) how come the authors label it the "Domestication Syndrome test battery"?

"erased due to focus shift"

Line 145-146: This could create a sampling bias where only the tamest dogs participate, i.e. the most fearful dogs would not. This can ultimately affect results with exaggerated sociability scores and more fearful or less explorative dogs being underrepresented in the sample.

That is indeed an important limitation of street tests since forcing the animals to participate would likely alter their behaviour even more, and we thus have to contend with the voluntary biased sample. While the human approach test somewhat circumvents this problem by starting the data collection before the dog is aware of being approached, we have to concede that this is a problem for all the other following subtests. We have endeavoured to address this point in the discussion (line 583ff) and mention a few possible alternatives there as well.

Line 160: Which domestication syndrome traits?

"erased due to focus shift"

Line 193-203: This seems like a good test of human-directed sociability.

We are glad you agree.

Line 225-253: Subtests 4-6, sure the authors wish to test other domestication hypotheses (though see my comments on pointing above), but since these tests take up half of the test battery and also seem to have the best feasibility and reliability, why then the eagerness to label the test battery the "Domestication Syndrome test battery". It seems to me, that this test battery unlikely to actually quantify domestication syndrome behaviours, if at all.

"erased due to focus shift"

Line 204-214: If the purpose is to quantify conspecific sociability and aggression, I am not sure I understand why the authors did not simply observe behaviours toward other dogs in the area? I am not convinced this test can be disentangled from neophobia and/or boldness.

Thank you for stating this concern. We do agree that elaborate long-term observations are the best way to assess a dog's social behaviour, whether that is pet, working, or free-ranging dog. However, this test was conceptualized to circumvent the necessity of elaborate observations to create the possibility to test more dogs without having to learn everything about their population first. In addition, we aimed at exploring the possibility of using established pet and working dog test batteries on the streets. The fake dog test has repeatedly been validated to reliably assess dogs' initial behaviour towards conspecifics (Barnard et al., 2019; Reid et al., 2022) and we were therefore positive that the same could be achieved on the street. With the outcome that the street dogs did indeed show appropriate conspecific behaviour (e.g., growling, barking, stand tall) upon

first interaction with the fake dog that was not seen in the novel object test, as well as frequent genital sniffing, we argue that the test did achieve that goal and can be used to assess the target measures when only the initial reaction is taken into account. Nevertheless, we cannot exclude that neophobia and boldness come into play as soon as the animals realize that the opponent is not, in fact, a dog. We discuss this issue in line 528f as well as the value of observational data in line 614f.

Line 365-366: So, no aggression behaviour? Maybe one fear/stress/redirected behaviour (Yawning)?

Indeed, the aggressive behaviours and many stress behaviours were rarely shown and could thus not be retained in the test-retest analysis. We discuss the problems with that as well as possible alternatives in line 602ff**.**

Line 387: But I thought that tail between legs had poor interrater reliability and should be excluded (given that the authors adhere to the 0.07 threshold as stated in several places, e.g. line 345, 347 and 356).

We apologize for this confusion. The tail between the leg and mouthing behaviour were close to the threshold and showed marked significance (p=0.02). After long discussion and in light of the somewhat arbitrary 0.7 threshold that is heavily influenced by sample size, we chose to retain the two variables for this preliminary investigation and treat them with caution. However, we do agree that we should have stated that more clearly and understand your annoyance with the lack thereof. An explanation has been added in the methods (line 299f) and the result section (line 356ff).

Line 388: Could this low test-retest reliability be caused by habituation in spite modification of the fake dog. Maybe the testing set-up alone could be cause for habituation? Perhaps testing conspecific sociability and aggression is better in real life social contexts?

Absolutely. We share this interpretation wholeheartedly, as we have explained in line 519f. Pitfalls of short retest intervals and too similar set ups are described in the paragraph thereafter (lines 522ff), and we have added the value of long-term observations in line 614.

Line 401: Again, I am puzzled by the continued use of tail between legs even though it does not meet the authors stated 0.07 ICC threshold. I find it to be borderline misleading.

Understandably so, we apologize for this lack of explanation. Please refer to our explanation for line 387 above for our modifications.

Line 404-405: This to me indicates both habituation, as mentioned above, but also that the fake dog was indeed treated as a novel object and not a suitable model of a conspecific.

We agree with this interpretation and have discussed the limitations as mentioned above.

Table 3: I find it very misleading, and confusing, to include variables that did not pass the ICC thresholds.

Again, we apologize and agree that we should have indicated this in a better way. As mentioned above, respective measures have been taken to do so.

Line 467-475: I disagree with the interpretation of the results on sociability and the conclusions brought forward here. The sample presented here is very likely to be a result of the most social dogs in the population. This is because the dogs' participation was entirely voluntarily and the dogs themselves could decide to leave the test at any given time. Since the study only includes test-retested dogs, this will inherently bias the sampling towards more social individuals and exclude fearful dogs.

Thank you for raising this concern. You are entirely correct that the voluntary participation creates a biased sample for the overall test-battery, and we have added a paragraph in lines 583ff to address this issue. At the same time, we would like to draw attention to three points that might alleviate your concern a little in this specific instance, i.e., the sociability (now Human approach) subtest. 1) As it was the very first subtest, the animals were not first required to come to us but rather we started the approach for this subtest before the animals were aware of it. More precisely, we appeared as normal locals to the animal right until I started the (now Human approach) test by calling their attention to me with a short vocalization. Since many animals were foraging or sleeping up to that point, they did not have a chance to flee beforehand. That means that by the time the very fearful animals started to flee, the test had already started, and their behaviour was still recorded and coded as part of the (now Human approach) test. 2) to address your concern about a bias in the retesting, we did find two-thirds of the dogs again that had already fled during the (now Human approach) subtest in the first test, creating a decent sample of fearful animals that is retained in the test-retest analysis for the sociability test. 3) And finally, the fact that only the more social animals stuck around during the tests does not negate the fact that sociability might be heritable. We do not mean to claim here that street dogs are more social than wolves (since we do not have that data and the skew would indeed cause a big issue with that) but rather that the sociability measure seems to be fairly consistent over time in an individual and could therefore serve as a trait based on which selection is possible. Rereading the sentence now I can see where it might have been interpreted differently and we have specified that we refer to consistency (line 481). To additionally counteract the perceived bias during this test, we renamed it to a more neutral "Human approach test" rather than the more directed "Sociability test".

Line 526-532: If the authors wish to speculate about phenotypic plasticity, I encourage them to do so in a more detailed way. What would the adaptive value of this be in free-ranging dogs?

Thank you for this encouragement. We have added a few lines how behavioural plasticity might aid free-ranging dogs in adapting to their unstable and variable environment, which is not a necessity for the average breed dog anymore (lines 550f).

Reviewer 2

There is too little known about the variation in behavior in stray/street dogs, and this study provides a survey of the types of behavioral tests that can and cannot be performed in this difficult testing environment. The study concept is thus very interesting and worthwhile. I enjoyed reading the description of traits tested and the results and discussion. As a preliminary study, I think it would be great to see the battery of tests published so that it could be retested with a larger sample.
However, I think the title, hypotheses, and design of the paper show a fundamental misunderstanding of some of the basic concepts in domestication studies. Domestication and the Domestication Syndrome (DS) hypothesis are not equivalent concepts, and the two are confounded repeatedly throughout the paper. The DS is a well-known and highly debated hypothesis that has been tested in many ways, taken apart, and found to be highly problematic. Most domestication workers agree that is has a very specific applicability when being tested. I provided more details on relevant sources in my "Itemized Review".There is no discussion of these issues in the present manuscript. The DS aims to explain the generalized traits co-ocurring in domestic populations when compared to wild counterparts. Dogs have repeatedly been criticized as a bad model for testing "early domestication" or the DS, due to their artificial breeding. In my opinion your study does not test the DS. It tests the presence of certain human-dog interactions that reflect tameness or lack of aggression in stray dogs. It is successful in that, and would be a great survey to publish as a starting point for behavioral studies in street dogs. However, as stated in my Itemized Review, the DS is not tested here. Tameness is but one trait, and it is present in all domesticated populations that have been studied thus far. I believe the authors could redesign and re-work this study with a better understanding of what they are testing. My best wishes to you and this work. I think it would be great to see this published once it is improved.

GENERAL ANSWER TO REVIEWER #2:

We would like to thank you for your helpful clarifications regarding the domestication syndrome and detailed elaborations how this paper could be improved. We agree that the focus of this paper was not ideally represented. Taking your encouragement and the suggestion of reviewer #1 into account, we chose to entirely refocus the paper towards the possibilities and limitations of free-ranging dog testing and centre our study within that. Since we do not have a large enough sample to reliably represent the distribution of specific traits across the population (as you mention, a larger sample would be needed for that), we solely focus on the presentation and reliability validation of the presented test battery to set a starting point for more elaborate testing. In addition, we discuss important considerations our results illustrate and alternatives that might be viable. These changes can be found especially in the entirely rewritten introduction and partially rewritten discussion, while the methods and result section stayed largely the same. Finally, we defined and used the terms free-ranging and street dogs more carefully and took your suggested references on board wherever possible, thank you for those pointers. We hope that by refocussing this paper on the merits it has in terms of moving the field forward, this version is more to your satisfaction.

Abstract:

1. A brief explanation about what is meant by “human-directed interactions” vs. “disturbance-prone behaviors” vs. “less disturbance-prone” would help the reader understand your study better from the very beginning.
   Thank you for pointing this out. We have entirely rewritten the abstract and therein explained the disturbance issue with more detail.
2. The term “free-ranging” is a bit vague and can be misinterpreted unless you define it specifically. For example, feral dogs like the Dingo have been described as free-ranging. Can your population perhaps be described further as “street dogs”? This is an important item because the type of population you are using relates directly to the testability of your hypothesis.
   This explanation was very helpful, thank you for that. We have added a definition for free-ranging dogs in the manuscript (line 57f), and while we continue to refer to free-ranging dogs wherever appropriate, we specifically state our use of street dogs for the test battery and adapted our interpretations and discussions to refer to the population our results can or cannot be generalized to.
3. Lines 44-45: the DS is not an attempt to explain domestication, but the *traits* associated with domestication. This may seem like a trivial difference but is really important to making your study more scholarly.
   That is indeed an important differentiation. While we have erased the words pertaining to this from the ms, we will endeavour to be more accurate in our future work.

Introduction:

1. Lines 43-44: The methods/pathways used to domesticate animals are pretty well known (see Zeder 2012 for a good overview). It is the mechanisms by which *multiple morphological/phenotypic traits correlated with domestication are co-occurring* repeatedly that is misunderstood. Again, I think this is an important distinction to make throughout your paper.

   Domestication is something that researchers struggle to define in a simple manner—but there is much literature on this—so defining the “type” of domestication you are testing is critical. I strongly suggest that you read Zeder 2012 for and overview of the different types of processes involved in the different types of “domestication”. Another view of what constitutes a “domestic” population is that of Lord et al. (several) – so there is much debate. This will inform whether your sampling is appropriate or not.

   Thank you for the reference suggestions. While we have erased the words pertaining to this from the ms, this is certainly a note we will keep in mind.
2. Line 56: Brain size reduction is not inconsistent among domesticated groups—quite the opposite. You can check Balcarcel et al. 2021 for a review of the literature on this subject and references therein, or Kruska’s work from the 1970s-2000’s. Your study cites Johnsson et al. 2021, which is a great paper, but I do believe there is an error in it regarding the statement of “increase” in brain size in pigs, llamas and alpacas. Johnsson et al cite Kruska (1974, 1982, 1988) and if you look at those original works, they report *decreases* in the telencephalon (not increases).
   That is an interesting thought and fact I was not aware of, thank you. Again something to keep in mind, though the line itself does not apply to the paper anymore.
3. Lines 59-60: There is a tremendous amount of literature debating the claims of the DS, its unsuitability in describing the variation presented by different domestic populations. I would

suggest discussing this a bit, to show your understanding of the subject, before getting into testing of the DS.

   a. For example, some domestication workers agree that dogs are a poor model for testing the DS hypothesis, to due to their directed artificial breeding during the last 200 yrs. (see Larson et al. 2014, McHugh & Larson 2017, Sanchez et al. 2016&2017 on lack of a universal pattern among domestics and the NCC).
   b. Dogs also don't necessarily show snout shortening, as claimed by the DS. Please see Drake et al. 2010 on the variation in cranio-facial changes in dog breeds which highlights the shortening of the snout as an artificially-selected trait in many breeds.

Thank you for this suggestion and we agree wholeheartedly, hence our suggestion that free-ranging dogs might be a better population. While most of this is not part of the ms anymore, we have incorporated some of it in line 80ff. Your references were very helpful in that.

4. IN GENERAL: You may want to rephrase or discuss the intricacies of the dog as a model for testing evolutionary processes. There is great potential in this model group, but not necessarily for testing the DS, in my opinion, since the DS is supposed to capture basic domestication traits common to all domestic populations…and dogs are highly artificially-selected. I think your study actually tests a *much broader* concept in domestication—the selection for tameness and reduced aggression—which is tremendously interesting.
   That is a valuable suggestion and with the rewriting of this ms we have attempted to shift the focus away from the ms and incorporated more broader domestication ideas particularly in the discussion (e.g., lines 481ff)
5. Lines 69-71: Here again, I think the authors may be confounding the domestication with "domestication syndrome".
   This line has been erased from the ms
6. "Free-ranging". I am concerned about the use of this term, and what it means for your sample. Hughes and MacDonald 2013 do not exclude feral populations under their definition of free-roaming dogs. Pilot et al. 2015 cite Hughes and MacDonald 2013, adding "not feral" to the definition, but I am not sure if there is evidence for that?
   Please see point 2 under abstract for our answer and implemented changes in regards to that.
