# Supplementary material for: Street-wise dog testing: Feasibility and reliability of a behavioural test battery for free-ranging dogs in their natural habitat
Source: PLoS One. 2024 Mar 14;19(3):e0296509. doi: 10.1371/journal.pone.0296509 (PMC10939227; doi:10.1371/journal.pone.0296509)
Supplement: Attachment — Submitted filename: PONE-D-23-22870_R2_DEC 2023.pdf [file pone.0296509.s012.pdf]

PLOS ONE

Street-wise dog testing: feasibility and reliability of a behavioural test battery for free-ranging dogs in their natural habitat

--Manuscript Draft--

**Manuscript Number:**	PONE-D-23-22870R2
**Article Type:**	Research Article
**Full Title:**	Street-wise dog testing: feasibility and reliability of a behavioural test battery for free-ranging dogs in their natural habitat
**Short Title:**	Feasibility and reliability of a novel test battery with free-ranging dogs in their natural habitat
**Corresponding Author:**	Svenja Capitain, M.Sc.<br>Linköping University: Linkopings universitet<br>Ernstbrunn, AUSTRIA
**Keywords:**	free-ranging dogs; street dogs; test battery; temperament test; test-retest reliability; fake dog; sociability; novel object; domestic dog; pointing; tractability
**Abstract:**	Behavioural scientists are increasingly recognizing the need to conduct experiments in the wild to achieve a comprehensive understanding of their species' behaviour. For domestic dogs (Canis familiaris), such progress has been slow. While the life in human households is often regarded as dogs' natural habitat, this classification disregards most of the global dog population. The value of experimentally testing free-ranging dogs' cognition and behaviour is increasingly being recognized, but no comprehensive test batteries have been conducted on those populations so far, leaving the feasibility and reliability of such endeavours unknown. This study is the starting point to fill this gap by pioneering and validating an elaborate behavioural test battery on street-living dogs. Therein, six common temperament tests (human-/conspecific-directed sociability, neophobia, tractability) and dog-human communication paradigms (pointing, inaccessible object) were adapted to the street conditions. We evaluated the feasibility of the test battery, the coding reliability of the measures, and investigated their temporal consistency in a retest of the same individuals six weeks later (test-retest reliability). The test battery proved feasible with most dogs participating in all subtests, and it showed satisfactory inter- and intra-rater reliability (0.84 and 0.93 respectively), providing evidence that complex behavioural tests can be conducted even in highly variable street conditions. Retesting revealed that some behaviours could be captured reliably across time, especially when the subtest was particularly engaging (e.g., human approach, point following). In contrast, the low retest reliability for subtests relying on sustained novelty and behaviours that were highly susceptible to disturbances (e.g., gazing) reflects the difficulties of street dog testing, including standardisation in disturbance-prone environments, ecology-dependent adaptation of methods, and intrinsic differences between pet and free-ranging dogs. With some adaptations, this test battery can be valuable in investigating cognition, behavioural profiles, and domestication in such an understudied population as free-ranging dogs.
**Order of Authors:**	Svenja Capitain, M.Sc.
	Giulia Cimarelli, PhD
	Urša Blenkuš, M.Sc.
	Friederike Range, Assoz.-Prof. PhD. Priv.-Doz.
	Sarah Marshall-Pescini, PhD, Priv.-Doz.
**Response to Reviewers:**	Reviewer #1:<br>Reviewer #1: Thank you for giving me the opportunity to review the revised version of this manuscript. I really appreciate the great effort the authors have put into changing the narrative to a methods paper. While I think the manuscript is much improved, I, however, still have some comments that I feel could still substantially improve the submission.<br>My main issue with the manuscript in its current form is that the great clarity and conciseness of the abstract is somewhat lost in the main text, where especially the

Powered by Editorial Manager® and ProduXion Manager® from Aries Systems Corporation

introduction, and to some extend the discussion, get overly wordy and complicated. This is a shame, as the message in the abstract is so to the point and all the extra fill added in the manuscript text takes away from this, leaving the reader with a quite confused impression in which the developed methods end up coming across much vaguer than they actually are. I therefore mainly have general comments regarding this issue and suggestions on how to deal with them:

General answer to Reviewer #1:
We would like to take this opportunity to thank you for your valuable thoughts and explaining your point of view in detail. We agree with the majority of your points and have taken them on board (detailed below). Therein, particularly the first half of the introduction has been slimmed down and the domestication subject has been reduced, though we do argue for its merit and hence retained a more nuanced picture of it. We hope that this version makes for an easier and more satisfying read and does the topic justice.

INTRODUCTION
The narrative about changes during domestication still seems to be forced into the manuscript and it is distracting from what it is really about: a methods paper testing whether it is feasible to use a behavioral test battery on free-ranging dogs (like it is used on pet dogs).
Answer: Thank you for seeing the merit in our paper and your detailed suggestions. We do agree that the domestication subject can be dropped in some areas (detailed below). However, we believe that disregarding the value free-ranging dogs could have for domestication research would not do the topic justice. And since reviewer 2 was delighted about the inclusion of this topic ("I am certain that the study adds great value to domestication studies focused on behavioral differences across populations"), we have chosen to retain it as a point of focus. Nevertheless, your arguments were convincing in that our iteration of the topic was not differentiated enough and took up too much space. Hence, we have cut it down in line with your suggestions and only retain it as one of the arguments for studying free-ranging populations, including the needed differentiation of breed-admixture in free-roaming populations.

For instance, in the very first line in the introduction (line 45-47) it is mentioned which behavioral traits are expected to change during dog domestication but this is irrelevant to the narrative of the sentence and the rest of the paragraph. Why not just focus on the history of dog domestication and the interest of dogs in science?
Answer: L45: the part about specific hypotheses has been removed to improve clarity. To underline for the lay person why behaviour might have been of interest to humans since early on, we did change the second part of the sentence to simply point to human-directed selection, though excluding the points to the specific hypotheses.

This line of argumentation leads me to the paragraph spanning line 76-92. I have already stated in my previous review that I largely agree that pet/breed dogs are not the best lineages of dogs for questions relating to domestication. This point has been emphasized by several large-scale genetic and behavioral studies. However, I do have to object to the statement that free-ranging dogs per se are better candidates for studying dog domestication. I suspect that the authors might be conflating the generic term free-ranging dogs with village dogs, which are independent canid lineages that predates breed dog formation. While true that the majority of the worlds' dog population is indeed free-ranging, all free-ranging dogs are not village dogs. A significant proportion of free-ranging dogs are abandoned pet dogs and should not automatically be labeled as an independent genetic lineage without some sort of genetic evidence hereof. I also feel that the Boyko references is particularly misguided in its use in line 86 as they show that while authentic village dog lineages do exist in Africa, many dogs are heavily admixed with breed dog lineages.
Answer: L76-92: Thank you for pointing out this important differentiation! We have changed the paragraph to point to the possible admixture more rigorously (now L73-78).

This is not to say that I disagree with the argumentation that 1) free-ranging dog populations are worth studying (they are and we should), and 2) that some of them are better representatives than breed dogs. I just think that especially point 2) should be approached with caution and maybe in this context, where the authors do not in fact

Powered by Editorial Manager® and ProduXion Manager® from Aries Systems Corporation

have any evidence for the genetic makeup of their free-ranging dog population, should err on the side of the narrative of point 1) and simply argue that because the vast majority of the worlds dog population is free-ranging AND thus free of strong artificial selection, we should simply make the effort to develop validated methods to quantify the behaviors of these dogs. That alone is a strong and clear argument – just like in the abstract. The other narrative (i.e. point 2)) is not important to the methods presented and just adds clutter and confusion.
Answer: We understand your point, and it has merit! We concede that we dwelled on the domestication part too much in the face of what this paper is actually about, particularly in this indicated part. Nevertheless, we do not want to overlook the value free-ranging populations can have for this topic altogether, and we see great importance in advocating for it. And since the paragraph is about the value of testing free-ranging dogs in general rather than our specific population, we argue that our current lack of knowledge about the latter's genetics does not invalidate that point. This paper is but a starting point rather than an insight into this population in particular. Hence, we retained part of our argumentation here but shortened it and phrased it more adequately to honour the very valid point you made above about admixture interferences (see point above, L73-78).

Line 93-120 is what it is all about – rigor and repeatability, love to see it!
Answer: Now L82-111. We are very grateful to hear that! This part has remained largely unchanged besides your comments below and the writing-related comments made by reviewer 2.

Specific comments:
Line 112: Interchanging use of free-ranging dogs and street dogs throughout the manuscript, which is a bit confusing.
Answer: We chose to alternate between street and free-ranging dogs on purpose to differentiate between our population and free-ranging dogs overall. While we argue for the importance of considering and testing free-ranging dogs, our sample focussed on street dogs, as defined in L56 and declared in L101. Hence, we mostly use free-ranging in the intro and then switch to street dogs in the methods and results. In the discussion, we alternate depending on whether it pertains to free-ranging populations overall or whether we can only infer to (these) street dogs in particular. While we understand that this might be a bit confusing, we do not want to make overgeneralized statements that we do not have any evidence for, and we trust the readers to understand the difference after rereading the definition in case confusion does occur. Nevertheless, to further aid this understanding, we added the definition again when introducing our methods at the end of the introduction (L101).

Line 114: Which domestication hypotheses. Why is this relevant to this methods paper?
Answer: These were initially mentioned in the first line of the paper but we have removed them from there now. Hence, we added them in brackets here (now L103).

Line 186: Seems like the authors are testing multiple behaviors at the same time here. Playfulness could be independent of sociability, no? Will sociability and play be separated in the analyses? Ah, I can see that playfulness was dropped from the analyses…
Answer: Indeed, we were testing sociability behaviours as well as play behaviour in this subtest as indicated in L165, divided by the different phases (Human approach – play). The two behaviour categories would have been analysed separately (as suggested by the different categories in the ethogram), but as you correctly identified, play had to be excluded due to low engagement.

Line 187: My objections to using the fake dog as a test of conspecific aggression from the first review remain unchanged given the results presented (even though the issues with the test possibly quantifying neophobia and boldness instead of conspecific aggression is now lightly touched upon in line 530-532).
Answer: We do agree that a fake dog is not the most ideal measure to assess conspecific behaviour. Its merits in this situation (safety, time-effectiveness, flexibility, standardisation) and the fact that is has been validated repeatedly, as well as the significant drawbacks (see paragraph on the fake dog (L511-521) and in the limitations (L586-589)) are discussed in the discussion and we make suggestions for better

approaches (shorter time, real life observations). Since it is the goal of this paper to present our used methods and discuss their pros and cons in depth, we will retain this part in the methods as is and trust the reader to make their own final judgement depending on their situation.

Line 211: Please also see Nawroth, C., Wiesmann, K., Schlup, P. et al. Domestication and breeding objective did not shape the interpretation of physical and social cues in goats (Capra hircus). Sci Rep 13, 19098 (2023) https://doi.org/10.1038/s41598-023-46373-9 for the latest on the pointing debate.
Answer: Thank you for this lovely read! It was very insightful. We have added it as a reference towards the point you make right below (L472).

Line 479-483: Not necessarily. These dogs could also have learned that humans mean food, which seems highly likely given that they are all scavengers. So, the behaviors the authors here associate with consistency and heritability could simply be the result of a learned behavioral process. How can the authors disentangle these concepts when they do not know the background (genetic, social etc.) of the dogs they are testing? This part is very speculative at best.
Answer: We agree that this part is speculative, and it was not intended otherwise. We did not mean to argue that it is hereditary in our population but rather point to other papers that have provided such evidence. Regardless, you are undoubtedly correct that we cannot disentangle whether life experience or genetics are the reason for such consistency here. We have added a few sentences in the paragraph (L470-472) to bring out that point and a possible argument that might nevertheless suggest a genetic component.

Line 502-511: This is a thoughtful paragraph. I wonder if point 3) does not leave room for habituation to the test situation, which might affect behavioral measurements?
Answer: Now L494: That is absolutely correct. We had some further elaborations towards those points initially but deleted it to decrease the word count, hoping that the reader could infer as much themselves. But we do agree that particularly for people not familiar with such testing methods, it should be pointed out. One solution might be that the time before the second testing should be substantially longer than 6 weeks, but seeing as we don't know when they would stop remembering the test, that is not ideal. Likewise, just starting from where the initial test was stopped might alter the behaviour in the subsequent subtests as well. Hence, we now added a half-sentence suggesting that the best way might be to repeat the test (for the sake of the missing subtests) but take the measures from the initial test for the subtests that are being repeated to exclude habituation. However, which approach is ultimately the best depends on the methods, goal, and testing constraints, and should thus be selected by the respective researchers themselves.

Line 565-566: The authors have not introduced the domestication syndrome until this point. The reader does not know that this syndrome, or neoteny, is and it is not relevant to the narrative. Strongly suggest deleting this part.
Answer: Thank you for this observant comment. We did delete the reference to the DS and neoteny and simply referred to retained play behaviour as a frequent trait in domesticated animals (L552).

Line 571-575: This part is only relevant if the authors know for sure that their dog population predates breed formation and they currently do not have the genetic evidence to back this statement up. In this light I find this line of argumentation irrelevant and speculative in regard to the results presented.
Answer: This part was taken out.

Reviewer #2:
I thank the Authors for addressing previous comments. The scope is much more constrained and I find it has improved the work significantly, resulting in a methods paper that domestication workers will find useful in the design of larger studies, and very informative on the challenges of behavioral studies outside of laboratory environments.
This manuscript is much improved, has better focus, and most importantly, now covers

Powered by Editorial Manager® and ProduXion Manager® from Aries Systems Corporation

its intended scope and stays within it. The study offers a behavioral testing method for street or free-roaming dogs, and presents results on the reliability of coding and retesting in a non-laboratory environment. It is presented as the longest and most complex behavioral test for this type of setting and sample. The methods are clearly described and the protocol is well explained. The suite of tests is reported to be successful in the amount of coding reliability, and less so in the retesting consistency, but overall, is informative on the feasibility of this type of behavioral assessment and on the challenges that can be encountered. It was a very interesting read, and I learned much while doing so. I am certain that the study adds great value to domestication studies focused on behavioral differences across populations, and hopefully will serve as a starting point for larger works. Importantly, it points out certain pitfalls in standard protocols that did not work well in a street environment, all which could improve and inform future work in this area. It is very interesting that the most consistent traits were those involving human engagement/communication.
I find the Abstract needs work. The wording is awkward in several sentences, and it makes the setup of the article confusing. There were several problems with sentence structure throughout the MS. Please see the pdf where I tracked the suggested edits, as well as edits to the Abstract. Of course, these are only suggestions.

General answer to reviewer #2:
Thank you very much for this detailed effort! We have taken your suggestion seriously and changed the ms accordingly (see below for detail). Since reviewer 1 expressed his great appreciation for the abstract, we chose to keep changes in this part to a minimum – besides honouring your suggestions. We hope this has created an easier read that is satisfactory for readers from many different scopes.

A few more specific comments + In-text comments:
Abstract
L24: lagging behind what?
Answer: This sentence has been changed to “has been slow” to take out the need for comparison.

L25: THis is another confusing, awkward sentence. You need to keep the subject of the sentence clear. "no tests have been conducted on [what]..."their [subject here is the tests, not the dogs, but structure the sentence so this is clear] reliability is unknown.."
Answer: Thank you for pointing this out. The sentences have been restructured to provide more clarity (L24-28).

L28; i suggest "on"
Answer: Accepted.

L 40: I am not sure there is evidence of this in your paper. Is there? I agree with that your results highlight difficulties in controlling the environment, but I missed how they find evidence of "intrinsic differences between pet/street dogs.
Answer: We were referring particularly to the play part in the human approach test here where a standardly used play and sociability test did not work at all with the free-ranging population since they did not seem to understand this concept. This intrinsic difference might be due to both learned and potentially genetic differences, as argued in L559-562.

L40: Reword: "bearing in mind the suggestions made in the discussion". Otherwise, sentence does not make sense. I would even start with""This test battery...." and removing the phrase before.
Answer: Now L41: The “bearing in mind” part has been deleted and replaced with “with some adaptations” since we did not want to advocate for this test battery in its unchanged form but do agree that the sentence structure was awkward.

Intro
L47: should be plural
Answer: Accepted.

L49: (a)if you start a phrase with "from" you need to follow up with "to"..... (b) multiple aspects of [what??] This whole phrase is vague...please specify what you are trying to say.

Powered by Editorial Manager® and ProduXion Manager® from Aries Systems Corporation

Answer: In order to shorten the intro as per reviewer 1's request, these two sentences were merged, which in turn deleted the words you commented on here. Nevertheless, we thank you for this useful suggestion.

L51: Again, awkward sentence structure...perhaps "there has been a surge in comparative studies..?""
Answer: Same as above.

L54: I suggest either "Importantly"or "however", but not both.
Answer: Thank you. We chose to stick with "however".

L57: Wow. Very interesting.
Answer: Right?! Hence the need to include them more.

L63: here you imply this is compared to something...compared to [what?]
Answer: Useful observation, thank you. For the sake or decreasing wordiness, we simply deleted this word.

L84: You would need to specify what "this" is, here.
Answer: As above, this part has been rewritten for the sake of wordiness and better nuancing.

L87; should be plural
Answer: Correct, thank you. However, this was deleted in line with reviewer 1's comment about nuancing this paragraph.

L93: I suggest "on"
Answer: Accepted.

L95: "in which they reside". I strongly encourage never ending a sentence with a preposition.
Answer: A valuable suggestion, thank you. Accepted.

L103: rater's.
Answer: Accepted.

L111: Grammatical suggestion: either (a) you "address" an issue or problem... (b) or you "have" objectives.
Answer: We have changed this to "achieve an objective".

L120: "to be used more.."
Answer: Since we do not only refer to a higher frequency but also a more widespread use, we changed this phrasing to "more widely used".

Methods
L202: this is not the definition of neophobia, correct? I suggest correcting this, it is confusing. // Did the authors err in their definition of "neophobia"? Or is it perhaps that in the writing style it seems as if they misinterpret its meaning? Please check. (Methods section).
Answer: Thank you for pointing this out. It was indeed not meant as the definition but rather as a clarification for all the assessable target behaviours. The line has been written more clearly (L193).

Discussion
L485: this sentence is unclear...you have differences between 3 things here...did you mean between 2 things?
Answer: This line was indeed confusing, thank you for catching that awkward phrasing. We deleted the obsolete part (L474).

L499: do not
Answer: Since we referred to the begging test here, the grammar was correct. However, we see how the sentence can be confusing, so we added this part in brackets to refer to the begging test specifically rather than have it as a half sentence (L486).

Powered by Editorial Manager® and ProduXion Manager® from Aries Systems Corporation

	L555: 1. Line 555 (Discussion): I am not sure how one could justify making the statement that “behavioral plasticity is selected against in modern [dog] breeds. But if made, please provide a citation or source. // Please provide a citation or supportive evidence for this from a source. I am not sure how one could justify making this statement that behavioral plasticity is selected against in modern breeds.<br>Answer: We do not mean to suggest that it was consciously selected against but rather that it might have been decreased through the selection for stable behavioural traits and the loss of genetic variation associated with that. This has been rewritten accordingly to make that point clearer and references have been added for the latter two points (L540-542).<br><br>L624: Reword to: "Bearing in mind the suggestions made..."<br>Answer: Accepted.
**Additional Information:**	
**Question**	**Response**
**Financial Disclosure**<br><br>Enter a financial disclosure statement that describes the sources of funding for the work included in this submission. Review the submission guidelines for detailed requirements. View published research articles from *PLOS ONE* for specific examples.<br><br>This statement is required for submission and **will appear in the published article** if the submission is accepted. Please make sure it is accurate.<br><br>**Unfunded studies**<br>Enter: *The author(s) received no specific funding for this work.*<br><br>**Funded studies**<br>Enter a statement with the following details:<br>• Initials of the authors who received each award<br>• Grant numbers awarded to each author<br>• The full name of each funder<br>• URL of each funder website<br>• Did the sponsors or funders play any role in the study design, data collection and analysis, decision to publish, or preparation of the manuscript?<br>• **NO** - Include this sentence at the end of your statement: *The funders had no role in study design, data collection and analysis, decision to publish, or preparation of the manuscript.*<br>• **YES** - Specify the role(s) played.<br><br>* typeset	The research was funded by the Austrian Science Fund (FWF, www.fwf.ac.at: project number P34749 for GC; I5052-B for SMP, UB and FR). The funders had no role in study design, data collection and analysis, decision to publish, or preparation of the manuscript. SC received no specific funding for this work.

Powered by Editorial Manager® and ProduXion Manager® from Aries Systems Corporation

**Competing Interests**<br><br>Use the instructions below to enter a competing interest statement for this submission. On behalf of all authors, disclose any competing interests that could be perceived to bias this work—acknowledging all financial support and any other relevant financial or non-financial competing interests.<br><br>This statement is required for submission and **will appear in the published article** if the submission is accepted. Please make sure it is accurate and that any funding sources listed in your Funding Information later in the submission form are also declared in your Financial Disclosure statement.<br><br>View published research articles from *PLOS ONE* for specific examples.<br><br>**NO authors have competing interests**<br><br>Enter: *The authors have declared that no competing interests exist.*<br><br>**Authors with competing interests**<br><br>Enter competing interest details beginning with this statement:<br><br>*I have read the journal's policy and the authors of this manuscript have the following competing interests: [insert competing interests here]*<br><br>* typeset	The authors have declared that no competing interests exist.
**Ethics Statement**<br><br>Enter an ethics statement for this submission. This statement is required if the study involved:<br><br>• Human participants<br>• Human specimens or tissue<br>• Vertebrate animals or cephalopods	Ethical approval for this study was obtained from the Ethical committee at the Agronomic and Veterinary Institute Hassan II (Comité d'Éthique de l'Institut Agronomique et Vétérinaire Hassan II) in Rabat, Morocco (Protocol number: CESASPV_2023_05). The free-ranging dogs' participation was voluntary, i.e., dogs were not restrained or forced to take part in the tests, and they were able to leave at any time. Moreover, the procedures were non-invasive and in accordance with the European Union Directive on the protection of animals used for scientific purposes (EU Directive 2010/63/EU).

• Vertebrate embryos or tissues<br>• Field research<br><br>Write "N/A" if the submission does not require an ethics statement.<br><br>General guidance is provided below. Consult the submission guidelines for detailed instructions. **Make sure that all information entered here is included in the Methods section of the manuscript.**	

Powered by Editorial Manager® and ProduXion Manager® from Aries Systems Corporation

**Format for specific study types**<br><br>**Human Subject Research (involving human participants and/or tissue)**<br>• Give the name of the institutional review board or ethics committee that approved the study<br>• Include the approval number and/or a statement indicating approval of this research<br>• Indicate the form of consent obtained (written/oral) or the reason that consent was not obtained (e.g. the data were analyzed anonymously)<br><br>**Animal Research (involving vertebrate animals, embryos or tissues)**<br>• Provide the name of the Institutional Animal Care and Use Committee (IACUC) or other relevant ethics board that reviewed the study protocol, and indicate whether they approved this research or granted a formal waiver of ethical approval<br>• Include an approval number if one was obtained<br>• If the study involved *non-human primates*, add *additional details* about animal welfare and steps taken to ameliorate suffering<br>• If anesthesia, euthanasia, or any kind of animal sacrifice is part of the study, include briefly which substances and/or methods were applied<br><br>**Field Research**<br><br>Include the following details if this study involves the collection of plant, animal, or other materials from a natural setting:<br>• Field permit number<br>• Name of the institution or relevant body that granted permission	
**Data Availability**<br><br>Authors are required to make all data underlying the findings described fully available, without restriction, and from the time of publication. PLOS allows rare exceptions to address legal and ethical concerns. See the PLOS Data Policy and FAQ for detailed information.	Yes - all data are fully available without restriction

A Data Availability Statement describing where the data can be found is required at submission. Your answers to this question constitute the Data Availability Statement and **will be published in the article**, if accepted.<br><br>**Important:** Stating 'data available on request from the author' is not sufficient. If your data are only available upon request, select 'No' for the first question and explain your exceptional situation in the text box.<br><br>Do the authors confirm that all data underlying the findings described in their manuscript are fully available without restriction?	
**Describe where the data may be found in full sentences. If you are copying our sample text, replace any instances of XXX with the appropriate details.**<br><br>• If the data are **held or will be held in a public repository**, include URLs, accession numbers or DOIs. If this information will only be available after acceptance, indicate this by ticking the box below. For example: *All XXX files are available from the XXX database (accession number(s) XXX, XXX.)*.<br>• If the data are all contained **within the manuscript and/or Supporting Information files**, enter the following: *All relevant data are within the manuscript and its Supporting Information files.*<br>• If neither of these applies but you are able to provide **details of access elsewhere**, with or without limitations, please do so. For example:<br><br>*Data cannot be shared publicly because of [XXX]. Data are available from the XXX Institutional Data Access / Ethics Committee (contact via XXX) for researchers who meet the criteria for access to confidential data.*<br><br>*The data underlying the results presented in the study are available from (include the name of the third party*	All relevant data are within the manuscript and its Supporting Information files.

and contact information or URL).<br>• This text is appropriate if the data are owned by a third party and authors do not have permission to share the data.<br><br>* typeset	
Additional data availability information:	

Manuscript
Click here to access/download;Manuscript;DSAdult22_PlosOne_2ndRevManu

Street-wise dog testing: feasibility and reliability of a behavioural test battery for free-ranging dogs in their natural habitat

Svenja Capitain[1*], Giulia Cimarelli[2], Urša Blenkuš[2], Friederike Range[2], Sarah Marshall-Pescini[2*]

[1] Department of Physics, Chemistry and Biology (IFM), Linköping University, Linköping, Sweden

[2] Domestication Lab, Konrad Lorenz Institute of Ethology, Department of Interdisciplinary Life Sciences, University of Veterinary Medicine Vienna, Vienna, Austria

* Corresponding author

Svenja.capitain@vetmeduni.ac.at (SC)

Sarah.marshall@vetmeduni.ac.at (SMP)

Abstract

Behavioural scientists are increasingly recognizing the need to conduct experiments in the wild to achieve a comprehensive understanding of their species' behaviour. For domestic dogs (*Canis familiaris*), such progress has been slow. While the life in human households is often regarded as dogs' natural habitat, this classification disregards most of the global dog population. The value of experimentally testing free-ranging dogs' cognition and behaviour is increasingly being recognized, but no comprehensive test batteries have been conducted on those populations so far, leaving the feasibility and reliability of such endeavours unknown. This study is the starting point to fill this gap by pioneering and validating an elaborate behavioural test battery on street-living dogs. Therein, six common temperament tests (human-/conspecific-directed sociability, neophobia, tractability) and dog-human communication paradigms (pointing, inaccessible object) were adapted to the street conditions. We evaluated the feasibility of the test battery, the coding reliability of the measures, and investigated their temporal consistency in a retest of the same individuals six weeks later (test-retest reliability). The test battery proved feasible with most dogs participating in all subtests, and it showed satisfactory inter- and intra-rater reliability (0.84 and 0.93 respectively), providing evidence that complex behavioural tests can be conducted even in highly variable street conditions. Retesting revealed that some behaviours could be captured reliably across time, especially when the subtest was particularly engaging (e.g., human approach, point following). In contrast, the low retest reliability for subtests relying on sustained novelty and behaviours that were highly susceptible to disturbances (e.g., gazing) reflects the difficulties of street dog testing, including standardisation in disturbance-prone environments, ecology-dependent adaptation of methods, and intrinsic differences between pet and free-ranging dogs. With some adaptations, this test battery can be valuable in investigating cognition, behavioural profiles, and domestication in such an understudied population as free-ranging dogs.

Introduction

The behaviour of dogs has been of central interest to humanity for millennia – possibly since the early stages of dog domestication 15.000 years ago through human-directed selection for specific traits [1-3]. While

dogs appear already in the work of early scientists such as Darwin [5] or Pavlov [6], it is only in the last 30 years that the research into dogs' behaviour and cognition has been recognized for its intrinsic value, resulting in an explosion of studies focusing on dogs' cognitive and human-directed capabilities [reviewed by 7, 8], their temperament and personality traits [9], and the influence of domestication [reviewed in 10] and our own anthropogenic environment [e.g., as model for aging: 11] on behavioural development.

However, these efforts have primarily revolved around a relatively limited subset of the global dog population – those under immediate human care and control, such as pets, working, and shelter dogs [9]. While easy to access, this subset represents less than a quarter of the global dog population with the other 75-83% consisting of free-ranging dogs [12, 13]. These animals might or might not be owned but share the characteristic that their movement and mate-choice are largely unrestricted by human control [14], even though the majority still resides as street dogs that depend on human settlements for food and shelter [12].

Efforts to explore the behaviour of free-ranging dogs have so far predominantly relied on observational methods [e.g., 15, 16, reviewed in 17], leading to important insights into these populations. While such studies are time-intensive and lack the experimental manipulation required to answer some of the questions addressed in the lab, they were so far chosen over experimental approaches due to several challenges that are associated with conducting more standardized test batteries for free-ranging dogs in their natural habitat: Beside the risk of aggression and disease status [e.g., rabies: 18], restraining the animals for a test would be unethical and obstruct the testing of natural behaviour. Hence, tests must rely on voluntary participation and transportable, flexible test setups. Established methodologies and apparatuses thus have to be adapted to the mostly unexplored street conditions, with some inevitable loss of direct comparability, given the different contexts in which animals are tested [19-21]. Finally, this lack of a stable test environment impedes standardisation and the variability and disturbance risk might distort results [22], particularly if the aim is to test the highly dynamic populations repeatedly [reviewed in 17, 20, 23].

Nonetheless, there are compelling reasons to experimentally assess the behaviour and personality of free-ranging dogs. For one, without studying a representative sample of the global dog population encompassing

at least some of the vastly different life experiences and mating choice they may have, our knowledge about dogs' cognitive abilities, human-animal interactions, the connection between genetics and personality, and beyond remains compromised [20, for more specific ideas see 22]. Secondly, it has been shown that, while the admixture of different breeds can be high in free-ranging populations [24], a large proportion is represented by genetically distinct populations that predate the recent strict artificial selection for breed formation [14, 24]. Compared to our heavily selected modern breeds where domestication traits might have become uncoupled [25, 26], those populations provide important insights to domestication research both from a genetic and ecological perspective [27]. Finally, free-ranging dogs' pervasive presence in human settlements (e.g., 2,930 dogs/km² in Kathmandu, Nepal [31]) means that millions of people spend their daily life in dogs' direct proximity. Increasing our understanding of their behaviour and behavioural profiles could therefore improve both human and animal welfare and aid in effective population control [22, 27].

Some pioneering groups have already initiated work on free-ranging dogs, demonstrating that an experimental approach, particularly if involving short, one-off tests can indeed be conducted under the variable yet natural conditions in which they reside [19, 21, 32-34]. While presenting a valuable start, these tests have focused on single behavioural aspects in short tests [e.g., persistence in 19, pointing in 21], leaving the possibility of longer tests, test batteries, and repeated testing that is needed to explore behavioural profiles, largely unexplored.

Furthermore, critical information regarding the reliability of measures obtained from such tests on the streets remains scarce – information that is vital not only for singular behavioural testing but especially for comprehensive temperament assessments. Several aspects of reliability should therein be considered: the coding reliability, which describes how well the behaviours defined in the ethogram and the testing conditions allow for consistency in a rater's observations over time ("intra-rater reliability") and with others ("inter-rater reliability"), and the "test-retest reliability", which assesses a test's ability to capture the behavioural traits reliably. A reliable test would be expected to elicit the same behaviour in an individual again when being tested in the same test twice ("retest"). This is particularly important in terms of personality traits, which are defined as being consistent over time and context in an individual [9]. Test-

retest reliability outcomes are often rather low in pet breed and shelter dogs, but some kind of consistency over time has often been reported [35, 36]. Whether this also pertains to free-ranging dogs and/or under highly variable street conditions remains to be explored.

In response to this research gap, our study endeavours to achieve a twofold objective. First, to pilot and evaluate the feasibility of a test battery specifically tailored to street dogs (i.e., free-ranging dogs living in and around human settlements), encompassing a range of behaviours commonly assessed in dog cognition or temperament tests and suggested to be relevant for different domestication hypotheses [tameness: 1, sociability: 2, deferential behaviour: 3, lack of aggression: 4]. These behaviours include human-directed approachability, conspecific sociability using a 'fake dog' test, neophobia and exploration in a novel object test, dogs' understanding of human communication in a pointing test and their use of human-directed communication (gazing) in a begging test, as well as their propensity to shy away from conflict in a tractability test [37-41]. Second, we assess the reliability of this test battery in the natural street setting, including inter-rater, intra-rater and test-retest reliability. We draw conclusions from the results on free-ranging dog testing overall, as well as how this test battery could be adapted to be more widely used in the field.

Methods

Ethics statement

Ethical approval for this study was obtained from the Ethical committee at the Agronomic and Veterinary Institute Hassan II (Comité d'Éthique de l'Institut Agronomique et Vétérinaire Hassan II) in Rabat, Morocco (Protocol number: CESASPV_2023_05). The street dogs' participation was voluntary, i.e., dogs were not restrained or forced to take part in the tests, and they were able to leave at any time. Moreover, the procedures were non-invasive and in accordance with the European Union Directive on the protection of animals used for scientific purposes (EU Directive 2010/63/EU).

Study area and population

The testing of the study subjects was conducted along beach, industrial, and urban areas in the Sous-Massa region in Morocco. The study subjects were part of a big dynamic free-ranging dog population living as scavengers in and around human settlements, hence classifying as street dogs [20]. The behaviour battery was first piloted on a random sample of eight adult street dogs (4 males, 4 females). Since the test battery was finalized after the first three and thus remained the same for the other five pilot dogs, they were added to the final test sample. Hence, a total of 36 adult street dogs (20 males, 16 females; 5 pilot plus 31 test sample dogs) were tested to assess the feasibility and reliability of the finalized test. Twenty-six of those dogs (15 males, 11 females) were found again for the retest.

Procedure

The test battery consisted of six short behavioural subtests and three physiological samplings conducted in direct succession (Fig 1). The subtests were designed to resemble behaviour tests that have commonly been used to assess pet, shelter, or working dog temperament traits, dogs' cognitive abilities, or traits that have been suggested as central during the domestication process (see description of the respective subtests). The three physiological samples (saliva and hair) were part of the test battery to assess the feasibility of sampling in such a setting and allow for later use of those samples in future exploration.

Fig 1. The sequence of the employed test battery. Subtests in light and physiological sample collections in dark grey.

For the test-retest reliability measure, the test battery was conducted a second time ("retest") with the same study subjects within an average time span of 46.28 days (min. 33, max. 76 days) which was similar to other test validation procedures [e.g., 42, 43-45]. To this end, the location of the initial test was noted, and the area was frequently visited again after five to six weeks until the dog was found, identified with previous pictures, and tested again. It was noted if the dog was retested in a different location than the first test (i.e., not in visual vicinity of the previous location). The procedure in the first and retest was exactly the same as described below. For the object-based subtests (Fake dog and Novel object subtest, see below), two slightly

different-looking versions were used respectively for the test and the retest to create novelty. The order of presentation of the two stimuli was randomized across subjects.

Initiation and general circumstances

Two experimenters were involved in each testing occasion: E1 was always the same person (SC) and conducted the actual tests while E2 (three different people) video-recorded, helped set up the subtests, or distracted other dogs if necessary. Dogs were tested individually and had to be alone before and during the test. Tests were conducted between sunrise and 10 a.m. before the areas became too crowded. The experimenters accessed the study area by car and all dogs encountered alone were tested. If at any point during testing, the dog ran off more than 10 m, the subtest during which the dog left was terminated and E1 tried to lure the dog back. If the dog ran too far to be retrieved, or did not respond to the lure, the whole test was terminated. If anyone other than the experimenter approached and visibly distracted the dog (e.g., head turn), the test was interrupted until the criterium was restored and the test resumed. If at any point dogs showed active signs of aggression, the test was terminated (however, this never occurred). For each dog, location, time, weather, health status, and activity upon arrival of E1 (e.g., sleeping, walking, …) were noted.

The exact setup of each subtest is displayed in Fig 2, the procedure of each subtest is demonstrated in the supporting information (S1 Video).

Fig 2. The set-up of each of the six subtests in the presented test battery. The main experimenter (E1) is presented in black (or grey in later stages of the subtest), the assistant experimenter (E2) in white.

Human Approach Test (Subtest 1)

Target: Approachability, human-directed sociability/ docility, tameness, play. This test was modelled after similar tests used in widescale personality assessment tests (e.g., the Dog Mentality Assessment (DMA) in the Swedish Kennel Club [46] and has in part been used with Ethiopian village dogs [34]. Beside the assumption that human-directed sociability is an essential trait under selection during the domestication

process [2], assessing the actual human-directed behaviour in a free-ranging population is provides valuable insights for disease- and population control.

Here, E1 stepped out of the car and whistled shortly to grab the dog's attention. She then stepwise proceeded to 1) stand quietly looking at the subject for 30 s, 2) called the dog whilst making friendly gestures (snapping, tapping on leg) for another 30 s, 3) approached the dog for 30 s up to 2 m distance, 4) stood for another 30 s, 5) approached the dog to one body length distance and paused another 30 s, and 6) crouched for another 30 s all while continuing to call the dog over. If the dog approached to less than 10 cm of E1 during any of those phases, E1 kneeled and tried to pet the dog for 30 s. If tolerated, E1 used a small brush to collect hair samples. The dog was then invited to play tug of war with a rag toy (1 m long) for 30 s.

Fake Dog Test (Subtest 2)

Target: Conspecific-directed sociability and aggression. Several studies support the idea that stuffed dogs can be used to reliably assess dogs' initial behaviour towards conspecifics [37, 47]. A reduction in conspecific aggression has been hypothesised as an important shift from wild to domestic types [48], and understanding intraspecific aggression in free-ranging dogs can provide important applications for the local population.

Hence, subjects were exposed to a "Giant Jack Russel Terrier" stuffed fake dog (brand: 'Melissa & Douk'; wither height 28 cm; S2 Supplementary Information). The two used versions differed in ear posture (pointy or droopy), tail length (medium and long), and colour (one original, one wrapped in dark bags) in the test and in the re-test respectively, in a randomized order across subjects. The fake dog was positioned 5 m from the car behind a V-shape visual barrier with its opening towards the car. This visual barrier allowed only the approaching subject but no other surrounding dogs to see the fake dog. E1 guided the subject towards the fake dog (if needed with food) and quickly walked >5 m away as soon as the subject saw the fake dog. The subject's behaviour was observed for 2 min.

Novel Object Test (Subtest 3)

Target: Neophobia, exploration/novelty-seeking. Presenting a dog with an unknow object is commonly used to assess novelty-seeking and boldness [41, 49], and explorative-behaviour is hypothesised to be lower in domesticated animals as part of the domestication syndrome [48].

To present an object that was truly novel to the dogs, we fixed a 90 – 100 cm high foil balloon with a wooden stick on top of a remotely controlled car (31x18x18 cm, Model: DEERC DE42 RC, S2 Supplementary information). Two balloon forms were used (dinosaur, a number (zero, five) sprayed in neon green and blue) for test and retest and randomized between study subjects. The set up was the same as the Fake dog test. As soon as the dog saw the novel object, E2 started moving it back and forth for 1 min through remote control. The toy was then left inactive for another 1 min. The dog's behaviour was observed for the full 2 min.

Pointing Test (Subtest 4)

Target: Dog-human communication skill, willingness to follow commands, cooperative communication [reviewed in 30, 50]. Studies have shown that a wide array of species, including canids, can follow pointing cues [for review see 50]. Exploring this phenomenon in free-ranging dogs with differing levels of human experience will further our understanding of the role of life-experience vs. species predisposition (but see [21] for pioneering work in this regard).

The test started with four warm-up trials: E1 placed a baited bowl (12 cm diameter, 10 cm height) on the ground 2 m from the dog, standing 1 m away herself and verbally called the dogs attention. After the dog ate the food, the bowl was picked up, baited, and placed down again to familiarize the dog with the fact that the bowl contained retrievable food. The subtest was terminated if the dog did not approach the bowl within 30 s two times in a row. The warm-up trials were followed by twelve test trials: one empty and one baited bowl (both rubbed in food for smell, baited outside of the dog's view) were placed 1.5 m apart 2 m from the dog. E1 stood equidistant behind the bowls, attracted the dog's attention, and upon eye contact, pointed to the baited bowl for 2 s (momentary distal pointing). The dog was allowed to eat the food if it approached the correct bowl on the first choice, otherwise the bowls were immediately collected by E1. Each trial could

be repeated twice if the dog did not choose within 30 s, followed by one repetition of the warm-up trial (i.e., only one bowl with food). If after the third repetition, the dog did not choose, the subtest was terminated. We used six different randomized sequences related to the positioning of the baited/pointed bowl to the right or left of the experimenter (e.g., RLRLLRLRRLLR) that were called out to E1 by E2.

Begging Test (Subtest 5)

Target: human-directed communication, gazing behaviour, suggested to be help-seeking behaviour [38, reviewed by 39, but see for alternative explanations: 51, 52]. Also a behaviour suggested as a trait under selection during domestication but the wide variability between individuals depending on human socialisation calls for further investigation with differentially socialised populations [53, 54].

The dog was allowed to eat from the baited bowl three times. Afterwards, the bowl was visibly baited and placed out of reach but still visible on the car, a wall, or a tree. E1 stepped 1 m to the side and faced away from the bowl with a neutral facial expression. The dog's behaviour was observed for 1 min.

Tractability test (Subtest 6)

Target: Social inhibition, tractability/deferential behaviour (i.e., the ease to yield to higher command, conflict avoidance [3]). According to the deferential hypothesis [3], tractability has been a central selection factor in dog domestication. Furthermore, assessing dogs' inclination to avoid conflicts with humans in a resource scenario may also have it uses in a more applied setting.

E1 gave the dog a piece of food, then took a big piece of meat, showed it to the dog, and visibly placed it on top of a small stool (40 cm height). E1 loudly stepped between dog and stool, stared the dog in the eyes and firmly said "No, it's mine!". If the dog attempted to get to the stool, E1 loudly stepped in their way and repeated "No" in a firm tone. After 1 min of this, E1 stepped 1 m to the side and stood still for 30 s while looking at the food. Finally, E1 turned around and looked away for 30 s. The test was finished as soon as the dog ate the food or after the last 30 s.

Video coding

The tests were recorded by an action camera (4K 24fps WiFi Sports Action Camera or Action Cam Jeemak WiFi) fixed on E1's chest and a camera (Sony HDR-CX405 Full HD Camcorder, hand-held (subtest 1) or on a tripod (subtest 2-6)) handled by E2. BORIS (version 7.13.8, (Friard & Gamba, 2016)) was used for the video coding.

The ethogram aimed at capturing the target behaviours described for each subtest. We used a bottom-up approach by initially coding 55 variables with different modifiers (i.e., the person or object the behaviour was coded towards) that measured almost every potentially relevant aspect in the different subtests. The coded variables are displayed in Table 1 with a detailed ethogram in S3 Table. Social behaviours were based on the ethograms used at the Wolf Science Center of the University for Veterinary Medicine, Vienna [10], and to observe free-ranging dogs in Italy [16, 55].

This list of variables was narrowed down by 1) assessing their codability through the inter- and intra-rater analysis (for acceptable agreement see Statistical Analysis), 2) assessing the variables' centrality in the subtest by analysing their frequency (i.e., excluding variables that were displayed in less than 10% of tests), and 3) assessing their consistency through the test-retest reliability. Only behavioural variables that passed step 1) and 2) went on to be analysed in step 3) to ensure that the test-retest reliability was based on reliably coded and not overly skewed data [56].

Table 1. Coded behavioural variables and occurrence across subtests.

Category	Behavioural variable	Human approach	Fake Dog	Novel Object	Pointing	Begging	Tractability
**Proximity**	Close	E1	Fake dog, E1+E2	Novel object, E1+E2	-	E1	E1, stool
	Medium	E1	Fake dog, E1+E2	Novel object, E1+E2	-	E1	E1, stool
**Tail**	Wagging	E1	Fake dog	Novel object	E1	E1	E1
	Between legs	Yes	Yes	Yes	-	Yes	Yes
**Gazing**		E1	Fake dog	Novel object, E1+E2	-	E1	E1
**Vocalization**	Barking	E1	Fake dog	Novel object	-	E1	E1
	Growling	E1	Fake dog	Novel object	-	E1	E1
	Whining	E1	Fake dog	Novel object	-	E1	E1
**Displacement**	Nose-licking	Yes	Yes	Yes	-	Yes	Yes

	Body shake	Yes	Yes	Yes	-	Yes	Yes
	Stretching	Yes	Yes	Yes	-	Yes	Yes
	Yawning	Yes	Yes	Yes	-	Yes	Yes
	Sniffing the ground	Yes	-	-	-	-	-
**Physical contact**	Biting	E1	Fake dog	Novel object	-	E1	E1
	Body contact	E1	Fake dog	Novel object	-	E1	E1
	Jumping	E1	Fake dog	Novel object	-	E1	E1
	Licking	E1	Fake dog	Novel object	-	E1	E1
	Mouthing	E1	Fake dog	Novel object	-	E1	E1
	Pawing	E1	Fake dog	Novel object	-	E1	E1
	Sniffing	E1	Fake dog	Novel object	-	E1	E1
**Reactions**	Stand tall	E1	Fake dog	Novel object	-	E1	E1
	Bare teeth	E1	Fake dog	Novel object	-	E1	E1
	Lunge	E1	Fake dog	Novel object	-	E1	E1
	Head dip	E1	Fake dog	Novel object	-	E1	E1
	Belly exposure	Yes	Yes	Yes	-	Yes	Yes
	Flee	E1	Fake dog	Novel object	-	E1	E1
	Crouch	E1	Fake dog	Novel object	-	E1	E1
	Risk assessment	E1	Fake dog	Novel object	-	E1	E1
	Play	E1	Fake dog	Novel object	-	E1	E1
	Friendly	E1	Fake dog	Novel object	-	E1	E1
	Bow	E1	Fake dog	Novel object	-	E1	E1
**Marking**		Yes	Yes	Yes	-	Yes	Yes
**Defecating**		Yes	Yes	Yes	-	*Yes*	Yes
**Non-visible**		Yes	Yes	Yes	Yes	Yes	Yes
**Disturbance**		Yes	Yes	Yes	Yes	Yes	Yes
**Subtest specific variables**			Latency to approach fake dog	Latency to approach novel object	Observation of gesture: no/yes	Attempts to reach bowl	Latency to eat food
		Phase of first approach		Phase of first approach	Successful choice: no/yes		Phase of eating food
			Genital sniffing		No choice	2-way gaze alternation	
						3-way gaze alternation	

The table shows all behavioural variables (column 2) of a certain category (column 1) that were coded in the respective subtests (columns 3 - 8). The words in the subtest columns describe towards what person or object the behaviour was coded, while 'yes' means that the behaviour was coded regardless of direction, and a minus (-) indicates that the behaviour was not coded in the subtest. Behavioural variables were either coded as duration (D), frequency (F), occurrence (O), or latency since the beginning of the respective subtest (L). Coloured cells indicate that the variable occurred in less than 10 % of tests and was thus excluded from the test-retest analysis.

For the intra-rater reliability measures, a random subsample of 20% of the test videos (n=13; balanced between test and retest, full test batteries and tests that were terminated halfway through) were re-coded by the initial rater after three weeks. For the inter-rater reliability, a random subsample of 20% of the full-test videos (n=9) were coded independently by a second rater [41, for similar methods see 57]. Beforehand, the second rater was trained on three sample videos and the ethogram was further clarified where needed.

Statistical analysis

Inter- and intra-rater reliability was analysed for each behavioural variable summed across all subtests and modifiers to assess their codability. Test-retest reliability, on the other hand, was analysed for every single variable by modifier and subtest. Occurrence variables (e.g., in which phase the dog approached, termination, etc.) were converted into ordinal variables before analysis. To normalize the data for comparison between test and retest, the time the dog was not visible or a disturbance was coded was subtracted from the total time of the subtest of the individual, and the behavioural data was then divided by this normative time. All individual subtests in which more than 70% of the time was coded as disturbance and/or not visible were excluded from further analysis. In case of the test-retest reliability, the specific subtest in both the test and the retest were excluded to allow for fair comparison.

The statistical analysis was then conducted in R (Version 4.2.2 [58]). To account for the systematic errors between raters/rating occasions, intraclass correlation coefficients (ICCs) were calculated to assess the reliability measures [59, 60]. Additionally, considering that both raters/rating occasions coded the same videos and that the goal was to generalize the reliability results, a two-way random effects ANOVA with an absolute agreement estimate was used to assess the inter- and intra-rater reliability [reviewed by 61]. According to the classification by [62], an ICC below 0.5 is considered poor reliability, between 0.5 and 0.75 it is moderate, between 0.75 and 0.9 it is good, and between 0.9 and 1.0 the reliability is considered excellent. For a behaviour to be regarded as having acceptable coding reliability and move on to the test-retest analysis, the ICC threshold for the inter- and intra-rater reliability was set to a moderate 0.70 [63] which is in accordance with similar studies [weighted average of 0.68 in a review of six studies by 9, 0.77 in 64]. However, careful reconsideration was employed in case the threshold was just about missed by taking

both coding reliabilities (inter- and intra-rater) into consideration and examining the p-value (assuming significance level α=0.05). For the test-retest reliability in the repeated sample, a two-way mixed effects ANOVA with absolute agreement estimate was applied [62]. Since significance largely depends on sample size and significant test-retest reliabilities in pet dog tests commonly range around an ICC of 0.5 which would usually be classified as poor to moderate reliability [average ICC of 0.43 in a meta-analysis of 31 studies in 35, e.g., 0.58 in 36, 0.57 in 41], a test-retest reliability with a p-value below 0.05 rather than a specific ICC was chosen as reliability indicator. Only variables that were displayed in more than 10% of the testing occasions were included in the test-retest reliability to circumvent unreliable analysis of heavily zero-inflated and low-prevalence data [for a similar approach see 56, 63, 65]. For the ordinal variables, two-way ANOVA's with absolute agreement estimate (equivalent to weighted Cohen's Kappa according to [66]) were used to analyse the coding and test-retest reliability. If the ICC was poor (i.e., below 0.5) for a certain variable, systematic biases in the measurements of both rating occasions were explored through scatter plots and variables were excluded if necessary. To control for the test-retest reliability being low due to disturbances during the tests, we conducted the same analysis again but excluded all individual subtests in which any disturbances were coded (46 of 235 subtests). Disturbance had been coded when the dog clearly turned its head towards an interfering human/dog/other animal for more than three seconds during a running subtest and while the test subject was visible on the video.

Lastly, because curiosity- or exploration-based tests may be more prone to lower consistency due to repeated exposure to similar stimuli [49], we tested if the dogs systematically showed a decreased reaction (exploration and fear) in the fake dog and novel object retest compared to the first test. To that end, applicable variables that were retained in the test-retest reliability (i.e., had good coding reliability and occurred in more than 10% of tests) were summed into an exploration-related and a fear-related variable [therein drawing from 67, 68] for each of the two subtests respectively. After transforming these four left-skewed variables with a square root transformation, a paired t-test was used to compare whether they differed significantly between test and retest.

Results

Subject characteristics

Out of the 36 initially tested street dogs, 23 (14 males, 9 females) were tested in the entire test battery, while nine dogs (5 males, 4 females) terminated the test during the Human approach subtest and four dogs (1 male, 3 female) after the first two or three subtests by running away. For the retest, 26 and thus 72% of the 36 initially tested street dogs were found again: 19 of those were dogs who went through the full test battery initially and 7 were dogs who had run away during the first testing. Fourteen were retested in a different location than the first test. All 26 dogs completed the same number of subtests in the retest as they did in the initial test.

Changes in methods and variables

After the test was adapted in the pilot testing, some additional changes from the above-presented procedure had to be made throughout the main testing period. For one, the play-part (rope presentation for tug-of-war) in the Human approach test caused four (28.57%) of the first 14 dogs to run away and terminate the test. For two more, the play phase had to be terminated to keep the dog from leaving. Thus, the play phase was dropped half-way through the main sample and the variable 'Interacting with the toy' was excluded from the analysis.

In addition, two more variables had to be excluded due to the following reasons: 'Auto-grooming' because the dogs had varying amounts of parasites, wounds, and dirt in their fur which influenced the scratching rate, and 'Sniffing the ground' in all but the Human approach test because food was dropped on the ground during the subtests and the different ground surfaces influenced the dogs' sniffing behaviour.

Intra- and inter-rater reliability

Both the intra- and inter-rater reliability coding, assessed across all subtests and modifiers, is reported in Table 2. Almost all variables passed the target ICC-threshold of 0.7 and had a significant p-value at 0.015 or below (the exact statistical outputs for each variable are reported in S4 table). The average of both inter-

and intra-rater reliability for all variables was above the target threshold of an ICC of 0.7, with a good reliability of 0.84 in the inter-rater and 0.93 in the intra-rater analysis.

There were only few variables that did not clear the threshold. ‘Tail between the legs’ and ‘Mouthing’ had moderate inter-rater reliability, meaning they still had an ICC above 0.5, and the p-value was below 0.05. Likewise, ‘Medium proximity’ had a moderate reliability within and between raters. The inter-rater reliability for ‘Head dip’, ‘Friendly’ and ‘Stand tall’, on the other hand, were poor with an ICC below 0.5 with a p-value above 0.05.

While ‘Tail between the legs’ and ‘Mouthing’ did not clear the initially defined threshold, they were both close and had a p-value of 0.02 which is substantially below the usual 0.05 significance level. Since previous studies have deterred from the arbitrary 0.7 ICC threshold in favour of significant p-values ($p<0.05$) [9], and both present crucial behaviours in dogs, we tentatively chose to keep them in the analysis going forward but treat their interpretation with utmost care. The other behaviours fell below the 0.05 significance level and were thus excluded from further analysis.

Table 2. Summary of the coding reliability across subtests.

**GENERAL**			
**Category**	**Behavioural variable**	**Inter-rater ICC**	**Intra-rater ICC**
**Proximity**	Close	0.90	0.97
	Medium	0.53	0.52
**Tail**	Wagging	0.91	0.91
	Between legs	0.64	0.99
**Gazing**		0.90	0.98
**Vocalization**	Barking	NA	NA
	Growling	0.80	NA
	Whining	NA	NA
**Displacement**	Nose-licking	0.88	0.96
	Shaking	0.80	NA
	Stretching	0.88	1.00
	Yawning	0.94	0.97

**SUBTEST SPECIFIC**			
**Subtest**	**Behavioural variable**	**Inter-rater ICC**	**Intra-rater ICC**
**Human approach**	Phase of first Approach	1.00	1.00
**Fake Dog**	Latency	1.00	0.97
	Genital sniffing	0.92	1.00
**Novel object**	Phase of first approach	1.00	1.00
	Latency	0.85	1.00
**Pointing**	Observation of gesture: yes	1.00	0.98
	Observation of gesture: no	1.00	0.99
	Successful choice	1.00	1.00
	Unsuccessful choice	1.00	1.00
	No choice	1.00	1.00
**Begging**	Attempts to get bowl	0.99	0.99
**Begging/ Tractability**	2-way gaze alternations	0.82	0.79

	Sniffing the ground	0.82	0.79		3-way gaze alternations	0.86	0.87
**Physical contact**	Biting	NA	NA	**Tractability**	Latency	0.90	1.00
	Body contact	1.00	0.74		Phase of eating	1.00	1.00
	Jumping	0.95	0.98				
	Licking	NA	0.84				
	Mouthing	0.64	0.95				
	Pawing	NA	0.99				
	Sniffing	0.97	0.98				
**Reactions**	Stand tall	-0.03	0.91				
	Bare teeth	NA	NA				
	Lunge	NA	NA				
	Head dip	-0.08	0.85				
	Belly exposure	NA	NA				
	Flee	0.77	0.94				
	Crouch	0.90	0.79				
	Risk assessment	0.99	0.86				
	Play	0.98	0.98				
	Friendly	0.44	0.97				
	Bow	0.81	0.76				
**Marking**		NA	0.99				
**Defecating**		NA	NA				
**Average**		**0.84**	**0.93**				

For each variable, the inter- and intra-rater reliability analysed across subtests and modifiers is displayed. Based on similar studies, a coding reliability of an ICC of 0.7 or above was regarded as acceptable (values with no specific highlight). Moderate reliability below this threshold (i.e., 0.5<ICC<0.7) is highlighted in light grey, and poor reliability (i.e., ICC<0.5) is marked in dark grey. All variables above an ICC of 0.7 had a p-value below 0.015, variables with an ICC between 0.5 and 0.7 had a p-value below 0.05, and all variables below an ICC of 0.5 had a non-significant p-value (p>0.05). Detailed analysis results with the 95% confidence interval, F-value statistic, degrees of freedom, and p-value can be found in S4 Table. Variables marked with NA did not occur in the coded tests.

Exclusion of infrequent variables

In the next step, variables that did not occur in more than 10% of the respective subtests were identified (Table 1) and excluded from the test-retest reliability analysis. In short, close proximity, tail wagging, gazing, nose licking and yawning were the only variables that occurred frequently in all subtests. On the

other hand, growling, whining, stretching, shaking, biting, licking, mouthing, marking, defecating, baring teeth, lunging, belly exposure, and bow were not displayed in more than 10% of tests in any of the subtests. Even after summing most of them into one pooled ‘stress’ variable [following the classification of 69, 70], they did not pass the 10% occurrence threshold and were hence excluded from the test-retest analysis. All subtest-specific variables except ‘attempt to reach the bowl’ in the begging test, occurred frequently enough to pass the 10% threshold.

Test-retest reliability

The final test-retest reliability analysis was conducted for each variable by subtest and modifier, only taking the variables into account that 1) were not already dropped from the ethogram/tests throughout the testing (i.e., auto-grooming, sniffing the ground, play), 2) had an acceptable coding reliability, and 3) occurred in more than 10% of all tests. The resulting test-retest reliabilities are reported in Table 3.

The average test-retest reliability was poor with an average ICC of 0.23. To see whether the poor outcome was influenced by disturbances during the tests that altered the dogs’ behaviour, we conducted the same analysis but excluded all subtests in which a disturbance had been coded (i.e., when the dog clearly turned its head towards an interfering human/dog/other animal for more than three seconds during a running subtest and while the test subject was visible on the video). While this changed the reliability of some behaviours, the average test-retest reliability did not improve (Table 3; the exact statistical outputs of both analyses can be found in S5 Table).

To summarize by subtest, the Human approach test performed well with ten out of 15 behaviours showing significant test-retest reliability, resulting in an average test-retest reliability of 0.32. In the Fake dog test, only ‘tail between the legs’ had a moderate retest reliability, with ‘close proximity to the fake dog’ and ‘nose-mouth licking’ also being moderately reliable after tests with disturbances were removed. The average test-retest reliability was low (ICC = 0.06). Similarly, only ‘close proximity to E1 and E2’ were significant in the Novel object test, being joined by ‘nose-mouth licking’ after disturbed tests were excluded, resulting in an average test-retest reliability of 0.12. The average reliability of the Pointing test was significant with

an average ICC of 0.51 with all subtest-specific variables, as well as ‘tail-wagging’, showing moderate test-retest reliability. The Begging test had a low test-retest reliability (average ICC = 0.16) with only the ‘close proximity to E1’ being significant across time when only looking at tests without disturbances. Lastly, ‘close proximity to E1’, ‘gazing at E1’, the ‘phase in which the dog ate the food’, ‘three-way gaze alternation between person and stool’ and ‘fleeing’ had significant reliability in the Tractability test, with the addition of ‘nose-mouth licking’ after disturbances were removed. The average test-retest reliability was 0.22.

Test-retest comparison for the curiosity-based subtests

The summed variables constituted of the following behaviours: “Exploration”-variable: ‘close’ and ‘medium proximity’, ‘gazing’, ‘tail wagging’, ‘friendly’, and ‘sniffing’ the fake dog/novel object, as well as ‘genital sniffing’ in the case of the fake dog. “Fear”-variable: ‘head dip’ and ‘tail between the legs’ for the fake dog subtest. Only ‘flee’ was retained as fear-representing behaviour in the novel object but was displayed so rarely, that it could not be analysed here.

The dogs showed significantly less exploration and less fear behaviours towards the fake dog in the retest than the first test (exploration: $t(14)=3.16$, $p<0.01$; fear: $t(15)=-3.08$, $p<0.01$). Exploration behaviours shown towards the novel object did not differ between test and retest ($t(11)=1.0$, $p=0.34$).

Table 3. Summary of the test-retest reliability in all retained variables.

Category	Behavioural variable	Human approach			Fake Dog			Novel Object			Pointing			Begging			Tractability		
		**M.**	**All**	**w/D**	**M.**	**All**	**w/D**	**M.**	**All**	**w/D**	**M.**	**All**	**w/D**	**M.**	**All**	**w/D**	**M.**	**All**	**w/D**
**Proximity**	Close	*E1*	0.71	0.77	*FD*	0.20	0.74	*NO*	-0.09	0.06	-			*E1*	0.8	0.76	*E1*	0.75	0.72
					E1+E2	-0.08	-0.17	*E1+E2*	0.74	0							*Stool*	-0.04	-0.02
**Tail**	Wagging	*E1*	0.61	0.62	*FD*	0.14	-0.12	*NO*	0.04	NA	*E1*	0.5	0.2	*E1*	0.36	0.3	*E1*	-0.14	-0.06
	Between legs	*Yes*	0.53	0.52	*Yes*	0.71	0.72											0.29	0.28
**Gazing**		*E1*	**0.35**	0.34	*FD*	0.16	0.11	*NO*	-0.35	-0.06				*E1*	0	0.24	*E1*	**0.48**	0.44
								E1+E2	-0.26	-0.08				*Bowl*	0.34	0.12	*Stool*	0.18	0.14
**Vocalization**	Barking	*E1*	0.85	0.85	*FD*	0.12	0.12												
**Displacement**	Nose-licking	*Yes*	0	-0.01	*Yes*	0.16	0.56	*Yes*	0.37	0.61	-			*Yes*	-0.2	-0.28	*Yes*	0.41	0.51
	Yawning	*Yes*	-0.03	-0.08	*Yes*	-0.06	0	*Yes*	0.48	0.45	-			*Yes*	-0.09	-0.1	*Yes*	-0.21	-0.2
	sniffing the ground	*Yes*	-0.04	-0.04	*Excl.*			*Excl.*			-			*Excl.*			*Excl.*		
**Physical contact**	Body contact	*E1*	0.55	0.55															
	Jumping	*E1*	0.5	**0.46**															
	Sniffing	*E1*			*FD*	0.03	0.19	*NO*	-0.04	0	-			*E1*	-0.09	-0.05			
**Reactions**	Flee	*E1*	-0.08	-0.16				*NO*	0.32	NA							*E1*	0.76	0.58
	Crouch																*E1*	-0.05	-0.02
	Friendly	*E1*	**0.45**	0.56	*FD*	-0.07	0.15							*E1*	0.34	0.35	*E1*	0.13	0.14
	Head dip	*E1*	-0.08	-0.09	*FD*	-0.21	0	*NO*	0	NA							*E1*	-0.07	-0.02
	Play	*E1*	-0.03	-0.06															
	Risk assesment				*FD*	-0.04	-0.04												
	Stand tall				*FD*	-0.12	-0.29												
**Subtest-specific variables**	Phase of first Approach	*E1*	**0.49**	0.51				*NO*	0.2	0	-			-			*Stool*	0.65	0.75
	Latency of first approach				*FD*	-0.15	0.2	*NO*	0.05	-0.9	-			-			*Stool*	-0.11	-0.08
	Genital sniffing				*FD*	0.15	-0.15												

	Observation of gesture: yes	-			-			-			*E1*	0.51	0.12	-			-		
	Observation of gesture: no	-			-			-			*E1*	**0.33**	-0.2	-			-		
	Successful choice	-			-			-			*Bowl*	0.64	0.38	-			-		
	Unsuccessful choice	-			-			-			*Bowl*	0.55	0.06	-			-		
	No choice	-			-			-			*Bowl*	0.52	0.17	-			-		
**Gaze alternations**	2-way	-			-			-			-			*E1/ bowl*	0.03	-0.08	*E1/ stool*	0.14	0.07
	3-way	-			-			-			-			*E1/ bowl*	0.1	0.08	*E1/ stool*	**0.38**	**0.38**
**Average per subtest**			**0.32**	**0.32**		**0.06**	**0.13**		**0.12**	**0.10**		**0.51**	**0.12**		**0.16**	**0.13**		**0.22**	**0.23**
**Average across the test battery**			**0.23**	**0.17**															

For each variable and subtest, the modifier (M.) and the test-retest reliability is displayed for all tests (All) and for only the tests did not have any coded disturbances (w/D) respectively. The modifiers were the main experimenter (E1), the fake dog (FD), the novel object (NO), the bowl, or the stool. Test-retest reliabilities with a p-value below 0.05 were regarded as acceptable. Behaviours with non-significant test-retest reliability are indicated with a light-grey background and in bold if they were significant but regarded as poor reliability (i.e., below an ICC of 0.5). Variables that were excluded due to low occurrence (i.e., less than 10% of testing occasions) are not shown unless their exclusion only applied to certain subtests. In that case, they are left empty or marked with NA if it only applies to the analysis without disturbances. Variables marked with a minus (-) were not coded in the respective subtest. Detailed analysis results with the 95% confidence interval, F-value statistic, and p-value can be found in S5 Table.

Discussion

In this study, we presented what, to our knowledge, is the first attempt at testing free-ranging dogs in a behavioural test battery and report feasibility and reliability of the test measures. Testing the dogs in their natural environment was indeed feasible and the coding reliability was high, indicating that this (relative to previous studies) long and complex behaviour test battery can be conducted with street dogs. The range from good to poor test-retest reliability, on the other hand, reflects the challenges inherent in conducting behaviour tests with free-ranging dogs on the streets.

Although the subtests in this test battery were adapted from mostly ‘lab-based’ behaviour assessments [e.g., 37, 41, 49] [but see 21], ~~and~~ standardizing the set up and testing our dog population in the streets turned out to be surprisingly manageable. Importantly, there were no terminations due to aggression towards people or equipment and most dogs completed all six subtests and sample collections despite an average test duration of 30 minutes, suggesting that street dogs (at least those of this population) can remain motivated throughout longer and more complex procedures than those used so far [e.g., 19, 33, 34, 71].

Similarly, the concern that testing and filming in the variable street conditions would negatively impact the coding reliability was unfounded. Our results surpassed the target of a mean ICC of 0.7 with good average inter- and intra-rater reliability of 0.84 and 0.93 respectively. Contrary to our expectations, the coding reliability in this street test was thereby *on par* or even better than many pet and working dog studies [ICC 0.68 in meta-analysis by 9, ICC >0.9 in 41, ICC 0.77 in 64], suggesting that our chosen variables were not only well defined but could also be coded reliably despite the more challenging conditions. Similarly encouraging inter-rater results have been reported for a few other street dog tests as well [19, 21], further highlighting that such results are achievable for street testing in general.

However, when looking at the reliability of the individual variables, a few did not meet the threshold. Notably, ‘medium proximity’, i.e., two body lengths distance from the experimenter, was the only variable which was low both in the inter- and intra-rater reliability analyses. With the constant adaptation of the camera angles to adjust for the variability of the street conditions, it might thus be best to stick to distances

that are easier to identify such as the more reliable 'close proximity' (i.e., one body length distance). Additionally, 'head dip', 'stand tall', and 'friendly' all had poor agreement between the raters, which might be due to the complex definitions of these behaviours (S3 Table). While these definitions were adapted from previously used ethograms [17, 72, 73], the filming in street conditions likely made the subtle cues harder to detect. Given the poor coding reliability of those traits, they should thus be dropped from the presented ethogram unless a more reliable definition or training method can be found and validated, and care should be taken in the establishment of future tests to balance between the complexity of target behaviours and filming feasibility in the streets.

While these outcomes paint a positive picture for street dog testing so far, the feasibility in terms of capturing the desired traits cannot be claimed without looking at the test's ability to elicit the same behaviours consistently over time. The presented test battery resulted in an average test-retest reliability of 0.23, which, according to [62], is regarded as poor reliability. While the definition of personality traits is firmly bound to their consistency over time and across contexts [9], capturing such behavioural consistency has been an issue for many test batteries. A meta-analysis of 31 studies revealed an average trait consistency of 0.43 in pet, shelter and working dogs, which was considered significant although the studies within displayed highly variable results ranging from -0.73 to 1.00 [35]. We found a similarly high variability in the test-retest reliability of the behaviours we assessed, with subtest reliability varying between a moderate ICC of 0.51 for the pointing test and a poorly reliable 0.06 for the fake dog test. Capturing consistent personality or behavioural scores over time is hence an issue not unique to the testing of street dogs in their natural habitat.

Intriguingly, most of the variables that were reliable over time were human-directed behaviours, including proximity to and tail wagging at the experimenter, body contact, and time of first approach. Likewise, the subtests in which the experimenter constantly interacted with the dog (i.e., human approach, pointing, tractability test) had the highest average test-retest reliability over time. Our results are consistent with other studies that report high reliability and strong heritability for sociability and biddability in pet dogs [e.g., 41, 49, 77] as well as dogs' performance during pointing tasks [76, 78], which might tentatively point to a genetic basis for these traits [2]. Simultaneously, while it has been shown that dogs already follow such

pointing cues as pups [74] and that their performance is stable regardless of keeping condition, time spent with the owner, or training [76], we cannot rule out that the individuals' life experience with human food provisioning played a part in these stable outcomes (as human socialisation seems to play a big part in pointing performance in other species [e.g., wolves: 74, goats: 75]).

More important in terms of street dog testing, however, is that the discrepancy in reliability between these social tests and the other subtests (fake dog, novel object, and begging test in particular) might be driven by the former's high interactivity, leaving the animal little time to be distracted. Contrary to pet dogs, free-ranging dogs have been shown to gaze at an inattentive person less than at an attentive person [33]. Moreover, testing in the free-ranging dogs' natural habitat means testing in highly variable and frequently disturbed environments. We tried to account for this by excluding all subtests with coded disturbances in a second analysis, however this did not improve reliability, possibly because we underrepresented the disturbances to our study subjects. Indeed, only six of the 35 dogs were alone in both tests (i.e., initial test and retest). For all others, additional dogs appeared during the test(s) and had to be 'distracted away' by the second experimenter. The appearance of other dogs is an unavoidable occurrence, and whilst testing can continue in such cases, it might explain why the subtests in which the dog had more time to focus on their environment without the direct engagement by the experimenter (fake dog, novel object, begging) showed a lower retest reliability. Whether behaviours like gazing and yawning and the entire begging test (which does not only lack interaction but also novelty) can reliably reflect a dog's behaviour under such circumstances is hence questionable and requires further rigorous testing in alternative test setups on the streets.

To address the disturbance problem on the streets, we propose four not mutually exclusive approaches: 1) Have at least one additional person on the team that is solely responsible for distracting approaching dogs out of sight. 2) Allow for longer between-subtest-phases if the disturbance is temporary and let the dog return to an attentive state rather than pushing through the test. However, one should keep in mind that this changes the time dogs spend in the overall test procedure and carefully consider an influence on the respective goal. 3) If the distraction is substantial, terminating the test and either repeating (though the

measures from the initial testing should be used for the repeated subtests) or resuming the test on a different day might be an option, depending on the respective test set up, goal, and familiarity with the population. 4) Meticulously coding any distraction might allow for correction of the disturbances, or at least provide deeper understanding of their influence on the behaviour, based on which decisions for future testing can be made.

Even after considering the disturbance problem, the consistency of the dogs' behaviour in the fake dog and novel object tests was notably low. This might seem surprising since both tests are regularly used in dog temperament assessments [e.g., 37, 65]. However, we could not find any reference to the retest reliability of fake dog tests, and even highly standardised studies exploring novel object tests and novelty-seeking and fearlessness traits often showed poor test-retest reliability (i.e., ICC<0.50) [e.g., Novelty-seeking: 0.48 in 41, Environmental Sureness: 0.16 in 64]. Accordingly, it has been proposed that test-retest outcomes are sensitive to the novelty of the object and situation due to learning effects [49]. Indeed, the fact that the free-ranging dogs explored the fake dog significantly more in the initial test while showing less fear in the retest supports the assumption that the animals partially remembered the initial encounter and adapted their behaviour accordingly. Although we made efforts to modify the appearance of the fake dog and novel object for the retest, the situation, procedure, and set-up remained the same and seem to have lacked the novelty needed to "fool" the animals twice within just six weeks. Nevertheless, it needs to be stressed that the street dogs frequently displayed appropriate conspecific, startling, and investigation behaviour in the initial presentation. Additionally considering that those subtests have been validated with fake vs. real dogs in case of the fake dog test [37, 47] and deemed reliable despite similar retest problems in case of the novel object [e.g., 41, 49], we suggest that these two subtests are still suitable to test the street dogs when only a single exposure to the test is needed. Additionally for the fake dog test, the social behaviours towards the stimulus were mostly exhibited in the first half of the subtest, suggesting that it is the initial reaction to the fake dog that is the most representative of conspecific behaviours. A shorter test duration and/or movement in the fake dog [e.g., 47] could thus be considered to decrease not only the influence of distractions, but also the

risk that neophobia and boldness traits replace the conspecific behaviours as soon as the dogs realize that the fake dog is not in fact real.

At the same time, these two subtests act as a reminder of the importance of appropriate between-test-intervals, particularly for test batteries investigating behavioural profiles. With free-ranging dogs, the balance between waiting out potential learning effects and retesting early enough to find a considerable number of test subjects again can be difficult, especially if the study population is not well-known. Our substantial retesting rate of 72% within an average of six weeks was hence surprising, though the considerable variation between the individuals (min. 33, max. 76 days) has to be acknowledged. Meticulous notes on appearance, location, and particular behaviours were crucial in this effort. While working with a well-known population would streamline such efforts, our results show that it is not a prerequisite. In addition, using fewer memorable cues (e.g., the V-shaped wooden board in the Fake dog and Novel object subtest) might reduce learning effects and allow for a shorter between-test-interval with better outcomes than presented here.

Beside these two probable explanations for the low test-retest reliability in some subtests, an additional – though highly speculative – explanation might be that free-ranging dogs could be less genetically fixed in some behavioural traits than modern breed dogs and thus behave less consistently in general. This idea might not be too farfetched, considering that free-ranging dogs are still subject to free mate choice and the instability of their environment [14]. Behavioural plasticity for certain traits (e.g., adaptiveness to the highly dynamic group compositions, weather conditions, or anthropogenic changes) might therefore be a valuable and thus widely retained trait in free-ranging dogs to deal with those constant environmental changes. Meanwhile, the directional selection for certain reliable breed characteristics in modern breed dogs [80, 81] and the resulting considerable loss of genetic variation [79] might have decreased behavioural plasticity in our modern breeds. Indeed, the study of reaction norms in wild fish and birds provides cautious evidence that behavioural plasticity (i.e., intra-individual variability) can have a heritable component [82, 83]. And though a meta-analysis targeting the question whether behaviour-selected working-breed dogs were more fixed in their behaviour than non-selected non-working breed dogs could not find any support for this

hypothesis [35], it was reported that an unstable rearing environment does not seem to have an effect on the consistency of behaviour, further highlighting the stability of certain traits in breed dogs [76]. Exploring how this compares to free-ranging dogs is an exciting avenue for further research.

A last consideration regarding the test itself pertains to the exclusion of the play component in the Human approach test and what that teaches us about the need to consider one's study subject in all its facets. Play is frequently used in temperament tests to assess sociability [36, 40, 46], and the retainment of play behaviour into adulthood has also been suggested as a consequence of domestication in several species [reviewed in 48, 84]. When applying this subtest with the street dogs, the low engagement rate and frequent fearful behaviour towards the toy might be explained by the negative or lack of experience such unowned free-ranging scavengers had with pulling on an object with a human [23]. Similarly, [19] suggested that free-ranging dogs' lower persistence in comparison to pet dogs was likely due to their need to preserve energy and a lack of experience with human-manipulated items. On the other hand, our anecdotal observation with free-ranging populations indicates multiple incidents of conspecific and object play. Whether play behaviour does indeed persist into adulthood in free-ranging dogs in the conspecific context or solitary play, but not with a human partner, or whether our choice of method was just ill-considered, remains to explored and emphasizes how carefully the subject population, its ecology and life experience must be considered despite our extensive experience with the species itself.

Finally, two important limitations need to be addressed in terms of free-ranging dog testing as well as the representativeness of this study. First, the reliance on voluntary participation when testing adult free-ranging dogs creates a natural bias for more social animals, especially in longer and more interactive tests, and might be particularly challenging for feral populations (i.e., not reliant on human settlements). This bias is somewhat reduced in our Human approach test since the only selection criteria for starting a test was that the dog was seemingly alone. Hence, even the dogs that ran away fearfully upon approach were analysed as part of our dataset. The fact that we were able to retest more than half of these 'escaping' individuals and they manifested the same behaviour on second testing provides evidence that this measurement was indeed reliable. Nevertheless, these individuals were lost for all the following subtests and are therefore not part of

the majority of the reliability analysis. At the same time, constraining a free-ranging animal would undoubtedly alter its natural behaviour in a test situation. Explorable options to test such dogs could be the use of shorter tests with little human presence wherever possible (e.g., reaction- or exploration-based tests) or testing populations that now live in a fenced shelter but genetically belong to the clade of free-ranging dogs and had spent most of their lives roaming freely. However, the influence of recent life experiences and social context in the shelter on the target behaviour have to be strongly considered [22]. Secondly, the fact that our study population has previously been recognized as exceptionally friendly [20] did not allow us to analyse the reliability of aggressive and many stress-related behaviours. For example, only 16.7% of our sample fled immediately and none showed aggression, while more than 50% of an Ethiopian village dog sample fled upon approach and 11% reacted aggressively (though one needs to consider that these dogs were not always tested alone or outside [34]). While expanding the presented test battery to other populations would initiate the characterisation of different free-ranging dog populations and what may affect differences in their behavioural profiles, we acknowledge that these limitations are likely difficult to be overcome. Extreme fear or the slightest sign of human-directed aggression will necessarily lead to aborting the test (particularly considering the risk of lethal disease, [e.g., 18]). And while using a fake dog rather than a real dog was indeed an attempt to test for the presence of aggression in a safe way, collecting additional observational data (regarding both human and conspecific directed behaviour) of the same individuals may be a complementary method allowing for multiple measures of specific behavioural traits.

In conclusion, the easy implementation, successful participation of most street dogs, and the high coding reliability presented here confirm that free-ranging dogs can be tested in longer test batteries with complex ethograms despite the challenging natural conditions. The wide variability in the test-retest reliability across subtests demonstrate that the management of disturbances, the choice of set up and test-retest intervals, and careful considerations of the subjects' life experience and ecology are central challenges when testing free-ranging dogs in their natural habitat. At the same time, the acceptable reliability in some of the subtest demonstrates that under well-chosen conditions, testing those populations is possible and opens a vast array of intriguing pathways to explore [22]. Bearing in mind the suggestions made in the discussion, going

forward the test battery presented here can be a valuable method to test questions about cognitive traits, behavioural profiles, ~~and domestication~~ in such an understudied population as free-ranging dogs.

Acknowledgements

We thank Professor Ikhlass El Berbri for her support and collaboration in this project. Additionally, we thank the rest of our team in Morocco (in particular Andreas Berghänel, Haytem Bouchri, Manon Delaunay, and Magdalena Juskaite) without whom the project would not have been possible. We are also grateful to the population and authorities in the Sous-Massa region for supporting our field work. Finally, we want to extend our gratitude to the two reviewers for their valuable feedback and suggestions.

Bibliography

1. Trut LN. Early Canid Domestication: The Farm-Fox Experiment: Foxes bred for tamability in a 40-year experiment exhibit remarkable transformations that suggest an interplay between behavioral genetics and development. American Scientist. 1999;87(2):160-9.
2. von Holdt BM, Shuldiner E, Koch IJ, Kartzinel RY, Hogan A, Brubaker L, et al. Structural variants in genes associated with human Williams-Beuren syndrome underlie stereotypical hypersociability in domestic dogs. Science advances. 2017;3(7):e1700398. doi: https://doi.org/10.1126/sciadv.1700398.
3. Range F, Marshall-Pescini S, Kratz C, Virányi Z. Wolves lead and dogs follow, but they both cooperate with humans. Scientific reports. 2019;9(1):1-10. doi: https://doi.org/10.1038/s41598-019-40468-y.
4. Hare B, Tomasello M. Human-like social skills in dogs? Trends in cognitive sciences. 2005;9(9):439-44. doi: https://doi.org/10.1016/j.tics.2005.07.003.
5. Darwin C. The descent of Man; and on Selection in relation to Sex: J. Murray; 1871.
6. Pavlov IP. The scientific investigation of the psychical faculties or processes in the higher animals. Science. 1906;24(620):613-9.
7. Udell MA, Wynne CD. A review of domestic dogs'(Canis familiaris) human-like behaviors: or why behavior analysts should stop worrying and love their dogs. Journal of the experimental analysis of behavior. 2008;89(2):247-61. doi: https://doi.org/10.1901/jeab.2008.89-247.
8. Bensky MK, Gosling SD, Sinn DL. The world from a dog's point of view: a review and synthesis of dog cognition research. Advances in the Study of Behavior. 2013;45:209-406.
9. Gartner MC. Pet personality: A review. Personality and Individual Differences. 2015;75:102-13. doi: https://doi.org/10.1016/j.paid.2014.10.042.
10. Range F, Marshall-Pescini S. Domestication Hypotheses Relating to Behaviour and Cognition: Which Are Supported by the Current Data? Wolves and Dogs: Springer; 2022. p. 335-73.
11. Creevy KE, Akey JM, Kaeberlein M, Promislow DE. An open science study of ageing in companion dogs. Nature. 2022;602(7895):51-7. doi: https://doi.org/10.1038/s41586-021-04282-9.
12. Hughes J, Macdonald DW. A review of the interactions between free-roaming domestic dogs and wildlife. Biological conservation. 2013;157:341-51. doi: https://doi.org/10.1016/j.biocon.2012.07.005.

13. Lord K, Feinstein M, Smith B, Coppinger R. Variation in reproductive traits of members of the genus Canis with special attention to the domestic dog (Canis familiaris). Behavioural processes. 2013;92:131-42. doi: https://doi.org/10.1016/j.beproc.2012.10.009.
14. Pilot M, Malewski T, Moura AE, Grzybowski T, Oleński K, Ruść A, et al. On the origin of mongrels: evolutionary history of free-breeding dogs in Eurasia. Proceedings of the Royal Society B: Biological Sciences. 2015;282(1820):20152189. doi: https://doi.org/10.1098/rspb.2015.2189.
15. Bonanni R, Cafazzo S. The social organisation of a population of free-ranging dogs in a suburban area of Rome: a reassessment of the effects of domestication on dogs' behaviour. The social dog: Elsevier; 2014. p. 65-104.
16. Cafazzo S, Valsecchi P, Bonanni R, Natoli E. Dominance in relation to age, sex, and competitive contexts in a group of free-ranging domestic dogs. Behavioral Ecology. 2010;21(3):443-55. doi: https://doi.org/10.1093/beheco/arq001.
17. Range F, Marshall-Pescini S. Wolves and Dogs: Between Myth and Science: Springer Nature; 2022.
18. Hampson K, Coudeville L, Lembo T, Sambo M, Kieffer A, Attlan M, et al. Estimating the global burden of endemic canine rabies. PLoS neglected tropical diseases. 2015;9(4):e0003709. doi: https://doi.org/10.1371/journal.pntd.0003709.
19. Lazzaroni M, Range F, Bernasconi L, Darc L, Holtsch M, Massimei R, et al. The role of life experience in affecting persistence: A comparative study between free-ranging dogs, pet dogs and captive pack dogs. PLOS ONE. 2019;14(4):e0214806. doi: https://doi.org/10.1371/journal.pone.0214806.
20. Lazzaroni M, Marshall-Pescini S. Der Streuner- das fehlende Bindeglied. In: Kitchenham K, editor. Streuner Hunde: Von Moskaus U-Bahn-Hunden bis Indiens Underdogs: Komsos; 2020.
21. Bhattacharjee D, Mandal S, Shit P, Varghese MG, Vishnoi A, Bhadra A. Free-ranging dogs are capable of utilizing complex human pointing cues. Frontiers in Psychology. 2020;10:2818. doi: https://doi.org/10.3389/fpsyg.2019.02818.
22. Horn L, Cimarelli G, Boucherie PH, Šlipogor V, Bugnyar T. Beyond the dichotomy between field and lab—the importance of studying cognition in context. Current Opinion in Behavioral Sciences. 2022;46:101172. doi: https://doi.org/10.1016/j.cobeha.2022.101172.
23. Paul MM, Sreejani Sen; Sau, Shubhra; Nandi, Anjan K.; Bhadra, Anindita. High early life mortality in free-ranging dogs is largely influenced by humans. Sci Rep. 2016;6(19641). doi: https://doi.org/10.1038/srep19641.
24. Boyko AR, Boyko RH, Boyko CM, Parker HG, Castelhano M, Corey L, et al. Complex population structure in African village dogs and its implications for inferring dog domestication history. Proceedings of the National Academy of Sciences. 2009;106(33):13903-8. doi: https://doi.org/10.1073/pnas.0902129106.
25. Hansen Wheat C, Fitzpatrick JL, Rogell B, Temrin H. Behavioural correlations of the domestication syndrome are decoupled in modern dog breeds. Nature communications. 2019;10(1):1-9. doi: https://doi.org/10.1038/s41467-019-10426-3.
26. Hansen Wheat C, van der Bijl W, Wheat CW. Morphology does not covary with predicted behavioral correlations of the domestication syndrome in dogs. Evolution letters. 2020;4(3):189-99. doi: https://doi.org/10.1002/evl3.168.
27. Vanak AT, Gompper ME. Dogs Canis familiaris as carnivores: their role and function in intraguild competition. Mammal review. 2009;39(4):265-83. doi: https://doi.org/10.1111/j.1365-2907.2009.00148.x.
28. Larson G, Fuller DQ. The evolution of animal domestication. Annual Review of Ecology, Evolution, and Systematics. 2014;45:115-36. doi: https://doi.org/10.1146/annurev-ecolsys-110512-135813.
29. Sánchez-Villagra MR, Geiger M, Schneider RA. The taming of the neural crest: a developmental perspective on the origins of morphological covariation in domesticated mammals. Royal Society open science. 2016;3(6):160107. doi: https://doi.org/10.1098/rsos.160107.

30. Range F, Marshall-Pescini S. Comparing wolves and dogs: current status and implications for human 'self-domestication'. Trends in Cognitive Sciences. 2022. doi: https://doi.org/10.1016/j.tics.2022.01.003.
31. Kato M, Yamamoto H, Inukai Y, Kira S. Survey of the stray dog population and the health education program on the prevention of dog bites and dog-acquired infections: a comparative study in Nepal and Okayama Prefecture, Japan. Acta Medica Okayama. 2003;57(5):261-6. doi: https://doi.org/10.18926/AMO/32829.
32. Bhattacharjee D, Sau S, Das J, Bhadra A. Free-ranging dogs prefer petting over food in repeated interactions with unfamiliar humans. Journal of Experimental Biology. 2017;220(24):4654-60. doi: https://doi.org/10.1242/jeb.166371.
33. Brubaker L, Dasgupta S, Bhattacharjee D, Bhadra A, Udell MA. Differences in problem-solving between canid populations: Do domestication and lifetime experience affect persistence? Animal Cognition. 2017;20(4):717-23. doi: https://doi.org/10.1007/s10071-017-1093-7.
34. Ortolani A, Vernooij H, Coppinger R. Ethiopian village dogs: Behavioural responses to a stranger's approach. Applied Animal Behaviour Science. 2009;119(3-4):210-8. doi: https://doi.org/10.1016/j.applanim.2009.03.011.
35. Fratkin JL, Sinn DL, Patall EA, Gosling SD. Personality consistency in dogs: a meta-analysis. PloS one. 2013;8(1):e54907. doi: https://doi.org/10.1371/journal.pone.0054907.
36. Valsecchi P, Barnard S, Stefanini C, Normando S. Temperament test for re-homed dogs validated through direct behavioral observation in shelter and home environment. Journal of Veterinary Behavior. 2011;6(3):161-77. doi: https://doi.org/10.1016/j.jveb.2011.01.002.
37. Barnard S, Kennedy D, Watson R, Valsecchi P, Arnott G. Revisiting a previously validated temperament test in shelter dogs, including an examination of the use of fake model dogs to assess conspecific sociability. Animals. 2019;9(10):835. doi: https://doi.org/10.3390/ani9100835.
38. Heberlein MT, Turner DC, Range F, Virányi Z. A comparison between wolves, Canis lupus, and dogs, Canis familiaris, in showing behaviour towards humans. Animal Behaviour. 2016;122:59-66. doi: https://doi.org/10.1016/j.anbehav.2016.09.023.
39. Mendes JWW, Resende B, Savalli C. A review of the unsolvable task in dog communication and cognition: comparing different methodologies. Animal Cognition. 2021;24(5):907-22. doi: https://doi.org/10.1007/s10071-021-01501-8.
40. Svartberg K. The hierarchical structure of dog personality in a new behavioural assessment: A validation approach. Applied Animal Behaviour Science. 2021;238:105302. doi: https://doi.org/10.1016/j.applanim.2021.105302.
41. Turcsán B, Wallis L, Virányi Z, Range F, Müller CA, Huber L, et al. Personality traits in companion dogs—Results from the VIDOPET. PLoS One. 2018;13(4):e0195448. doi: https://doi.org/10.1371/journal.pone.0195448.
42. Barnard S, Marshall-Pescini S, Passalacqua C, Beghelli V, Capra A, Normando S, et al. Does Subjective Rating Reflect Behavioural Coding? Personality in 2 Month-Old Dog Puppies: An Open-Field Test and Adjective-Based Questionnaire. PLOS ONE. 2016;11(3):e0149831. doi: https://doi.org/10.1371/journal.pone.0149831.
43. Brady K, Hewison L, Wright H, Zulch H, Cracknell N, Mills D. A spatial discounting test to assess impulsivity in dogs. Applied Animal Behaviour Science. 2018;202:77-84. doi: https://doi.org/10.1016/j.applanim.2018.01.003.
44. Plutchik R. Individual and breed differences in approach and withdrawal in dogs. Behaviour. 1971;40(3-4):302-11.
45. Wright HF, Mills DS, Pollux PM. Behavioural and physiological correlates of impulsivity in the domestic dog (Canis familiaris). Physiology & behavior. 2012;105(3):676-82. doi: https://doi.org/10.1016/j.physbeh.2011.09.019.

46. Svartberg K, Forkman B. Personality traits in the domestic dog (Canis familiaris). Applied animal behaviour science. 2002;79(2):133-55. doi: https://doi.org/10.1016/S0168-1591(02)00121-1.
47. Reid PJ, Cussen VA, Collins LK, Lockwood R. The Utility of Model Dogs for Assessing Conspecific Aggression in Fighting Dogs. Applied Animal Behaviour Science. 2022:105710. doi: https://doi.org/10.1016/j.applanim.2022.105710.
48. Kaiser S, Hennessy MB, Sachser N. Domestication affects the structure, development and stability of biobehavioural profiles. Frontiers in Zoology. 2015;12(1):1-11. doi: https://doi.org/10.1186/1742-9994-12-S1-S19.
49. Svartberg K, Tapper I, Temrin H, Radesäter T, Thorman S. Consistency of personality traits in dogs. Animal Behaviour. 2005;69(2):283-91. doi: https://doi.org/10.1016/j.anbehav.2004.04.011.
50. Krause MA, Udell MA, Leavens DA, Skopos L. Animal pointing: Changing trends and findings from 30 years of research. Journal of Comparative Psychology. 2018;132(3):326. doi: https://doi.org/10.1037/com0000125.
51. Marshall-Pescini S, Rao A, Virányi Z, Range F. The role of domestication and experience in 'looking back'towards humans in an unsolvable task. Scientific reports. 2017;7(1):46636. doi: https://doi.org/10.1038/srep46636.
52. Lazzaroni M, Marshall-Pescini S, Manzenreiter H, Gosch S, Přibilová L, Darc L, et al. Why do dogs look back at the human in an impossible task? Looking back behaviour may be over-interpreted. Animal Cognition. 2020;23(3):427-41.
53. Miklósi Á, Kubinyi E, Topál J, Gácsi M, Virányi Z, Csányi V. A simple reason for a big difference: wolves do not look back at humans, but dogs do. Current biology. 2003;13(9):763-6. doi: https://doi.org/10.1016/S0960-9822(03)00263-X.
54. D'Aniello B, Scandurra A. Ontogenetic effects on gazing behaviour: a case study of kennel dogs (Labrador Retrievers) in the impossible task paradigm. Animal cognition. 2016;19:565-70. doi: https://doi.org/10.1007/s10071-016-0958-5.
55. Bonanni R, Cafazzo S, Valsecchi P, Natoli E. Effect of affiliative and agonistic relationships on leadership behaviour in free-ranging dogs. Animal Behaviour. 2010;79(5):981-91. doi: https://doi.org/10.1016/j.anbehav.2010.02.021.
56. Pompey KT. A Unified Approach to Estimating the Intraclass Correlation Coefficient and Its Bias: an Exploratory Study: University of South Carolina; 2021.
57. Boström AF, Hyytiäinen HK, Koho P, Cizinauskas S, Hielm-Björkman AK. Development of the Finnish neurological function testing battery for dogs and its intra-and inter-rater reliability. Acta Veterinaria Scandinavica. 2018;60(1):1-9. doi: https://doi.org/10.1186/s13028-018-0408-2.
58. Team RDC. R: A language and environment for statistical computing. 2010.
59. Shrout PE, Fleiss JL. Intraclass correlations: uses in assessing rater reliability. Psychological bulletin. 1979;86(2):420. doi: https://doi.org/10.1037/0033-2909.86.2.420.
60. Harvey ND. A Simple Guide to Inter-rater, Intra-rater and Test-retest Reliability for Animal Behaviour Studies. 2021. doi: https://doi.org/10.31219/osf.io/8stpy.
61. Harvey ND, Craigon PJ, Sommerville R, McMillan C, Green M, England GC, et al. Test-retest reliability and predictive validity of a juvenile guide dog behavior test. Journal of Veterinary Behavior. 2016;11:65-76. doi: https://doi.org/10.1016/j.jveb.2015.09.005.
62. Koo TK, Li MY. A guideline of selecting and reporting intraclass correlation coefficients for reliability research. Journal of chiropractic medicine. 2016;15(2):155-63. doi: https://doi.org/10.1016/j.jcm.2016.02.012.
63. Patronek GJ, Bradley J, Arps E. What is the evidence for reliability and validity of behavior evaluations for shelter dogs? A prequel to "No better than flipping a coin". Journal of Veterinary Behavior. 2019;31:43-58. doi: https://doi.org/10.1016/j.jveb.2019.03.001.

64. Sinn DL, Gosling SD, Hilliard S. Personality and performance in military working dogs: Reliability and predictive validity of behavioral tests. Applied Animal Behaviour Science. 2010;127(1-2):51-65. doi: https://doi.org/10.1016/j.applanim.2010.08.007.
65. van der Borg JA, Beerda B, Ooms M, de Souza AS, van Hagen M, Kemp B. Evaluation of behaviour testing for human directed aggression in dogs. Applied Animal Behaviour Science. 2010;128(1-4):78-90. doi: https://doi.org/10.1016/j.applanim.2010.09.016.
66. Fleiss JL, Cohen J. The equivalence of weighted kappa and the intraclass correlation coefficient as measures of reliability. Educational and psychological measurement. 1973;33(3):613-9. doi: https://doi.org/10.1177/001316447303300309.
67. Moretti L, Hentrup M, Kotrschal K, Range F. The influence of relationships on neophobia and exploration in wolves and dogs. Animal Behaviour. 2015;107:159-73. doi: https://doi.org/10.1016/j.anbehav.2015.06.008.
68. Klausz B, Kis A, Persa E, Miklósi Á, Gácsi M. A quick assessment tool for human-directed aggression in pet dogs. Aggressive behavior. 2014;40(2):178-88. doi: https://doi.org/10.1002/ab.21501.
69. Beerda B, Schilder MB, van Hooff JA, de Vries HW. Manifestations of chronic and acute stress in dogs. Applied Animal Behaviour Science. 1997;52(3-4):307-19. doi: https://doi.org/10.1016/S0168-1591(96)01131-8.
70. Pedretti G, Canori C, Biffi E, Marshall-Pescini S, Valsecchi P. Appeasement function of displacement behaviours? Dogs' behavioural displays exhibited towards threatening and neutral humans. Animal Cognition. 2023:1-10. doi: https://doi.org/10.1007/s10071-023-01742-9.
71. Lazzaroni M, Range F, Backes J, Portele K, Scheck K, Marshall-Pescini S. The Effect of Domestication and Experience on the Social Interaction of Dogs and Wolves With a Human Companion. Frontiers in Psychology. 2020;11. doi: https://doi.org/10.3389/fpsyg.2020.00785.
72. Cafazzo S, Marshall-Pescini S, Lazzaroni M, Virányi Z, Range F. The effect of domestication on post-conflict management: Wolves reconcile while dogs avoid each other. Royal Society Open Science. 2018;5(7):171553. doi: https://doi.org/10.1098/rsos.171553.
73. Cimarelli G, Marshall-Pescini S, Range F, Berghänel A, Virányi Z. Relationship quality affects social stress buffering in dogs and wolves. Animal Behaviour. 2021;178:127-40. doi: https://doi.org/10.1016/j.anbehav.2021.06.008.
74. Virányi Z, Gácsi M, Kubinyi E, Topál J, Belényi B, Ujfalussy D, et al. Comprehension of human pointing gestures in young human-reared wolves (Canis lupus) and dogs (Canis familiaris). Animal cognition. 2008;11:373-87. doi: https://doi.org/10.1007/s10071-007-0127-y.
75. Nawroth C, Wiesmann K, Schlup P, Keil N, Langbein J. Domestication and breeding objective did not shape the interpretation of physical and social cues in goats (Capra hircus). Scientific Reports. 2023;13(1):19098.
76. Gácsi M, Kara E, Belényi B, Topál J, Miklósi Á. The effect of development and individual differences in pointing comprehension of dogs. Animal cognition. 2009;12(3):471-9. doi: https://doi.org/10.1007/s10071-008-0208-6.
77. Morrill K, Hekman J, Li X, McClure J, Logan B, Goodman L, et al. Ancestry-inclusive dog genomics challenges popular breed stereotypes. Science. 2022;17(6592):e0274383. doi: https://doi.org/10.1126/science.abk0639.
78. Bray EE, Gnanadesikan GE, Horschler DJ, Levy KM, Kennedy BS, Famula TR, et al. Early-emerging and highly heritable sensitivity to human communication in dogs. Current Biology. 2021;31(14):3132-6. e5. doi: https://doi.org/10.1016/j.cub.2021.04.055.
79. Gray MM, Granka JM, Bustamante CD, Sutter NB, Boyko AR, Zhu L, et al. Linkage disequilibrium and demographic history of wild and domestic canids. Genetics. 2009;181(4):1493-505. doi: https://doi.org/10.1534/genetics.108.098830.
80. Svartberg K. Breed-typical behaviour in dogs—Historical remnants or recent constructs? Applied Animal Behaviour Science. 2006;96(3-4):293-313.

81. Sutter NB, Ostrander EA. Dog star rising: the canine genetic system. Nature Reviews Genetics. 2004;5(12):900-10. doi: https://doi.org/10.1038/nrg1492.

82. Dingemanse NJ, Barber I, Wright J, Brommer J. Quantitative genetics of behavioural reaction norms: genetic correlations between personality and behavioural plasticity vary across stickleback populations. Journal of evolutionary biology. 2012;25(3):485-96. doi: https://doi.org/10.1111/j.1420-9101.2011.02439.x.

83. Araya-Ajoy YG, Dingemanse NJ. Repeatability, heritability, and age-dependence of seasonal plasticity in aggressiveness in a wild passerine bird. Journal of Animal Ecology. 2017;86(2):227-38. doi: https://doi.org/10.1111/1365-2656.12621.

84. Bradshaw JW, Pullen AJ, Rooney NJ. Why do adult dogs 'play'? Behavioural processes. 2015;110:82-7. doi: https://doi.org/10.1016/j.beproc.2014.09.023.

85. Kolm N, Temrin H, Miklósi Á, Kubinyi E, Garamszegi LZ. The link between selection for function and human-directed play behaviour in dogs. Biology letters. 2020;16(9):20200366. doi: https://doi.org/10.1098/rsbl.2020.0366.

86. Hansen Wheat C, Fitzpatrick J, Tapper I, Temrin H. Wolf (Canis lupus) hybrids highlight the importance of human-directed play behavior during domestication of dogs (Canis familiaris). Journal of Comparative Psychology. 2018;132(4):373. doi: https://doi.org/10.1037/com0000119.

87. Brownell D. The Brownell-Marsolais scale: a proposal for the qualitative evaluation of SAR/disaster K9 candidates. Adv Rescue Technol. 2002;5:57-67.

Supplementary Information

S1 Exemplary video showing the different subtests of the test battery

S2 Equipment information

S3 Table. Detailed Ethogram

S4 Table. Detailed inter- and intra-rater reliability results

S5 Table. Detailed test-retest reliability outcome

Fig1

Click here to access/download;Figure;Fig1.tif

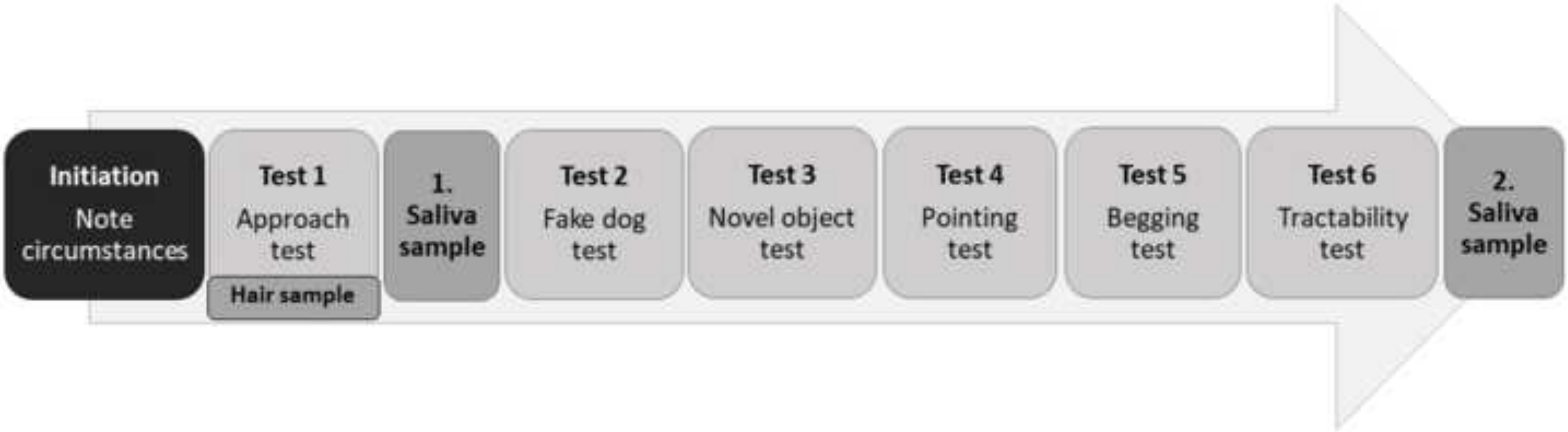

Fig2

Click here to access/download;Figure;Fig2.tif

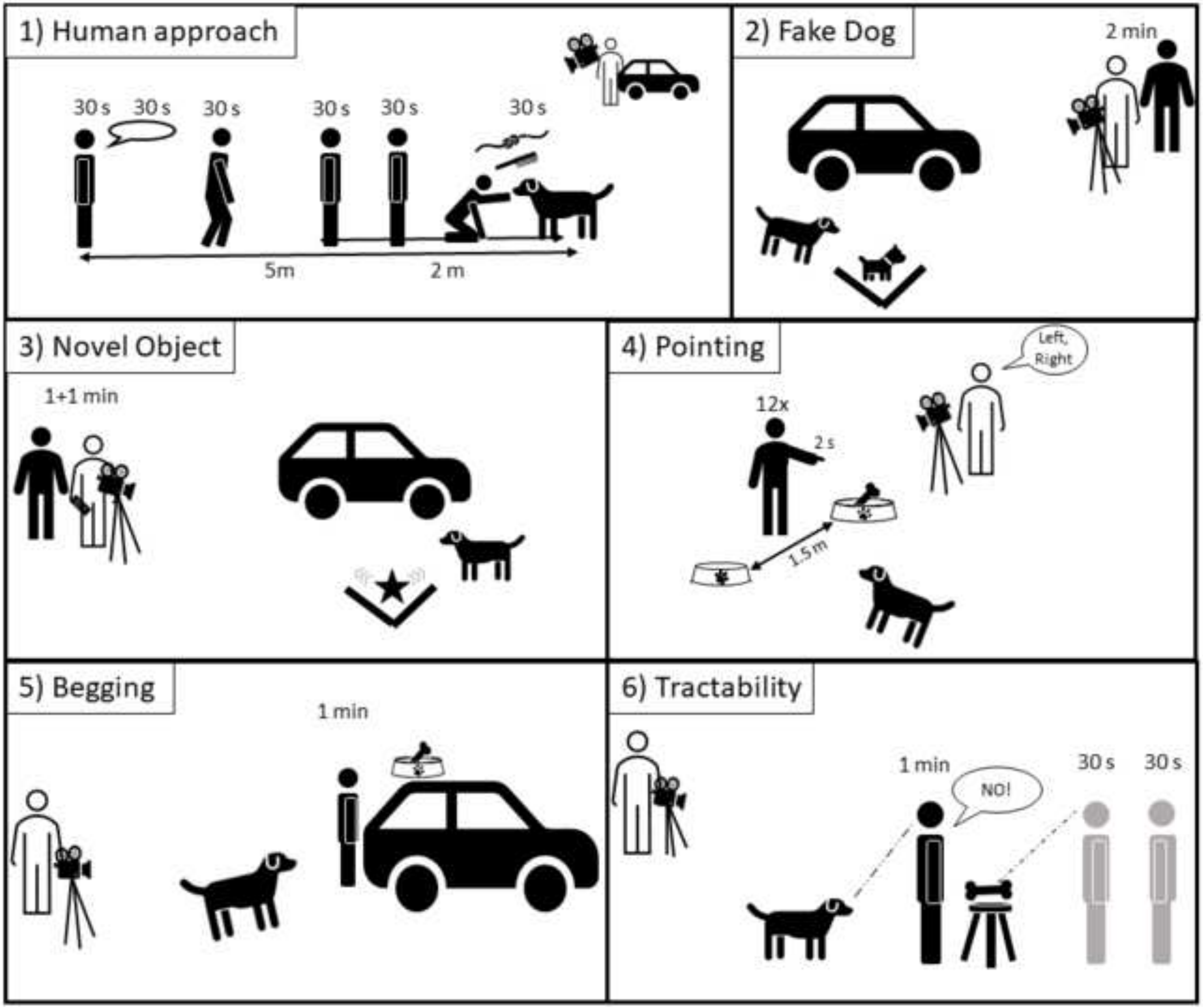

S1 Video

Click here to access/download
Supporting Information
S1_Video.mp4

Click here to access/download
Supporting Information
S2_Equipment information.pdf

S3 Table

Click here to access/download
Supporting Information
S3_Table.docx

S4 Table

Click here to access/download
Supporting Information
S4_Table.docx

S5 Table

Click here to access/download
Supporting Information
S5_Table.docx

Click here to access/download
Supporting Information - Compressed/ZIP File Archive
DSAdult22_PlosOne_Data.zip

Street-wise dog testing: feasibility and reliability of a behavioural test battery for free-ranging dogs in their natural habitat

Svenja Capitain[1*], Giulia Cimarelli[2], Urša Blenkuš[2], Friederike Range[2], Sarah Marshall-Pescini[2]*

[1] Department of Physics, Chemistry and Biology (IFM), Linköping University, Linköping, Sweden

[2] Domestication Lab, Konrad Lorenz Institute of Ethology, Department of Interdisciplinary Life Sciences, University of Veterinary Medicine Vienna, Vienna, Austria

* Corresponding author

Svenja.capitain@vetmeduni.ac.at (SC)

Sarah.marshall@vetmeduni.ac.at (SMP)

Abstract

Behavioural scientists are increasingly recognizing the need to conduct experiments in the wild to achieve a comprehensive understanding of their species' behaviour. For domestic dogs (*Canis familiaris*), such progress ~~is~~has been slow ~~lagging behind~~. While the life in human households is often regarded as dogs' natural habitat, this classification disregards most of the global dog population. The value of ~~including~~ experimentally testing free-ranging dogs' ~~as study subjects~~ cognition and behaviour is increasingly being recognized, but no comprehensive ~~behavioural and cognitive~~ test batteries have been conducted on those populations so far, leaving ~~-~~ the~~and their~~ feasibility and reliability of such endeavours ~~are~~ unknown. This study is the starting point to fill this gap by pioneering and validating an elaborate behavioural test battery on~~with~~ street-living dogs. Therein, six common temperament tests (human-/conspecific-directed sociability, neophobia, tractability) and dog-human communication paradigms (pointing, inaccessible object) were adapted to the street conditions. We evaluated the feasibility of the test battery, the coding reliability of the measures, and investigated their temporal consistency in a retest of the same individuals six weeks later (test-retest reliability). The test battery proved feasible with most dogs participating in all subtests, and it showed satisfactory inter- and intra-rater reliability (0.84 and 0.93 respectively), providing evidence that complex behavioural tests can be conducted even in highly variable street conditions. Retesting revealed that some behaviours could be captured reliably across time, especially when the subtest was particularly engaging (e.g., human approach, point following). In contrast, the low retest reliability for subtests relying on sustained novelty and behaviours that were highly susceptible to disturbances (e.g., gazing) reflects the difficulties of street dog testing, including standardisation in disturbance-prone environments, ecology-dependent adaptation of methods, and intrinsic differences between pet and free-ranging dogs. ~~Bearing the suggestions made in the discussion in mind~~With some adaptations, this test battery can be valuable in investigating cognition, behavioural profiles, and domestication in such an understudied population as free-ranging dogs.

Introduction

The behaviour of dogs has been of central interest to humanity for millennia – possibly since the early stages of dog domestication 15.000 years ago through human-directed selection for specific traits [1-3]. ~~,~~ ~~hypothesised to have come about through the selection for specific behavioural traits [hypothesised as: tameness: 1, sociability: 2, and/or deferential behaviour: 3, lack of aggression: 4].~~ While dogs appear already in the work of early scientists such as Darwin [5] or Pavlov [6], it is only in the last 30 years that the research into dogs’ behaviour and cognition has been recognized for its intrinsic value, resulting in an explosion of studies focusing on dogs’ cognitive and human-directed capabilities [reviewed by 7, 8], their temperament and personality traits [9], and the influence of domestication [reviewed in 10] and our own anthropogenic environment [e.g., as model for aging: 11] on behavioural development. ~~multiple aspects from understanding of human communication, theory of mind, learning processes, and beyond [reviewed by 7, 8]. Likewise, comparative studies have seen a surge of interest in dogs’ temperament traits and personality [9], with comparisons to other species shedding light on the profound influence of our own anthropogenic environment [e.g., as model for aging: 11] and domestication [reviewed in 10] on behavioural development.~~

~~Importantly, h~~However, these efforts have primarily revolved around a relatively limited subset of the global dog population – those under immediate human care and control, such as pets, working, and shelter dogs [9]. While easy to access, this subset represents less than a quarter of the global dog population with the other 75-83% consisting of free-ranging dogs [12, 13]. These animals might or might not be owned but share the characteristic that their movement and mate-choice are largely unrestricted by human control [14], even though the majority still resides as street dogs that depend on human settlements for food and shelter [12].

Efforts to explore the behaviour of free-ranging dogs have so far predominantly relied on observational methods [e.g., 15, 16, reviewed in 17]~~. While this has led to a more in depth understanding,~~ leading to important insights into ~~of~~ these populations. While such studies are ~~, it is comparatively~~ time-intensive and lack~~s~~ the experimental manipulation required to answer some of the questions ~~being~~ addressed in the lab

~~with pet, shelter and working dogs~~, they were so far chosen over experimental approaches due to~~.~~ ~~S~~several challenges that are associated with conducting ~~come to mind when hypothesizing the use of~~ more standardized test batteries for free-ranging dogs in their natural habitat: ~~First,~~ Beside the risk of ~~with little previous knowledge of the test subject, direct interaction might be hazardous regarding~~ aggression ~~or~~ and disease status [e.g., rabies: 18]~~. Second~~, restraining the animals for a test would be unethical~~, potentially dangerous,~~ and obstruct the testing of natural behaviour. Hence, tests must rely on voluntary participation and transportable, flexible test setups. ~~Thus, third, the~~Established methodologies and apparatuses thus have to be adapted to the mostly unexplored street conditions, with some inevitable loss of direct comparability, given the different contexts in which animals are tested [19-21]. Finally, this lack of a stable test environment impedes standardisation and the variability and disturbance risk might distort results [22], particularly if the aim is to test the highly dynamic populations repeatedly [reviewed in 17, 20, 23]. ~~Fourth, our limited knowledge of free-ranging dogs' behaviour during experiments requires adaptation of methodologies and apparatuses used with other dog populations, with some inevitable loss of direct comparability, given the different contexts in which animals are tested~~ [19-21]~~. Finally, repeated testing remains a challenging prospect given the rapidly changing dynamics in free-ranging populations~~ [reviewed in 17, 20, 23]~~.~~

Nonetheless, there are compelling reasons to experimentally assess the behaviour and personality of free-ranging dogs. For one, without studying a representative sample of the global dog population encompassing at least some of the vastly different life experiences and mating choice they may have, our knowledge about dogs' cognitive abilities, human-animal interactions, the connection between genetics and personality, and beyond remains compromised [20, for more specific ideas see 22]. Secondly, it has been shown that, while the admixture of different breeds can be high in free-ranging populations [24], a large proportion is represented by genetically distinct populations that predate the recent strict artificial selection for breed formation [14, 24]. Compared to our heavily selected modern breeds where domestication traits might have become uncoupled [25, 26], those populations provide important insights to domestication research both from a genetic and ecological perspective [27]. ~~This is particularly true for evolutionary questions and the~~

~~understanding of the domestication process. The intensive artificial selection in our modern breed dogs might have de-coupled morphological and behavioural traits hypothesized to co-occur in domestic species~~ [25, 26]~~ and the use of modern breed dogs as a model for domestication has therefore been contested by a number of authors [28, 29]. In support of this and contrary to the general assumption, the majority of free-ranging dog populations are not a mixture of breeds but rather genetically distinct populations that predate the relatively more recent artificial selection and breed formation [14, 24]. Thus free-ranging dogs might actually be a better candidate to elucidate the main domestication process in dogs from a genetic and ecological perspective~~ [27]~~, than modern breed dogs living in 'western-style' pet-owner relationships [30].~~ ~~Furthermore~~Finally, free-ranging dogs' pervasive presence in human settlements (e.g., 2,930 dogs/km² in Kathmandu, Nepal [31]) means that millions of people spend their daily life in dogs' direct proximity. ~~Improving~~ Increasing our understanding of their behaviour and behavioural profiles could therefore improve both human and animal welfare and aid in effective population control [22, 27].

Some pioneering groups have already initiated work ~~with~~ on free-ranging dogs, demonstrating that an experimental approach, particularly if involving short, one-off tests can indeed be conducted under the variable yet natural conditions in which they reside ~~in~~ [19, 21, 32-34]. While presenting a valuable start, these tests have focused on single behavioural aspects in short tests [e.g., persistence in 19, pointing in 21], leaving the possibility of longer tests, test batteries, and repeated testing that is needed to explore behavioural profiles, largely unexplored.

Furthermore, critical information regarding the reliability of measures obtained from such tests on the streets remains scarce – information that is vital not only for singular behavioural testing but especially for comprehensive temperament assessments. Several aspects of reliability should therein be considered: the coding reliability, which describes how well the behaviours defined in the ethogram and the testing conditions allow for consistency in a rater's~~'~~ observations over time ("intra-rater reliability") and with others ("inter-rater reliability"), and the "test-retest reliability", which assesses a test's ability to capture the behavioural traits reliably. A reliable test would ~~therein~~ be expected to elicit the same behaviour in an individual again when being tested in the same test twice ("retest"). This is particularly important in terms

of personality traits, which are defined as being consistent over time and context in an individual [9]. Test-retest reliability outcomes are often rather low in pet breed and shelter dogs, but some kind of consistency over time has often been reported [35, 36]. Whether this ~~will~~ also pertains to free-ranging dogs and/or under highly variable street conditions remains to be explored.

In response to this research gap, our study endeavours to ~~address~~ achieve a twofold objective. First, to pilot and evaluate the feasibility of a test battery specifically tailored to street dogs (i.e., free-ranging dogs living in and around human settlements), encompassing a range of behaviours commonly assessed in dog cognition or temperament tests and suggested to be relevant ~~in~~ for different domestication hypotheses [tameness: 1, sociability: 2, deferential behaviour: 3, lack of aggression: 4]. ~~.~~These behaviours include human-directed approachability, conspecific sociability ~~through~~ using a 'fake dog' test, neophobia and exploration in a novel object test, dogs' understanding of human communication in a pointing test and their use of human-directed communication (gazing) in a begging test, as well as their propensity to shy away from conflict in a tractability test [37-41]. Second, we assess the reliability of this test battery in the natural street setting, including inter-rater, intra-rater and test-retest reliability. We draw conclusions from the results on free-ranging dog testing overall, as well as how this test battery could be adapted to be more ~~pervasively~~ widely used in the field ~~and free-ranging dog testing overall~~.

Methods

Ethics statement

Ethical approval for this study was obtained from the Ethical committee at the Agronomic and Veterinary Institute Hassan II (Comité d'Éthique de l'Institut Agronomique et Vétérinaire Hassan II) in Rabat, Morocco (Protocol number: CESASPV_2023_05). The street dogs' participation was voluntary, i.e., dogs were not restrained or forced to take part in the tests, and they were able to leave at any time. Moreover, the procedures were non-invasive and in accordance with the European Union Directive on the protection of animals used for scientific purposes (EU Directive 2010/63/EU).

Study area and population

The testing of the study subjects was conducted along beach, industrial, and urban areas in the Sous-Massa region in Morocco. The study subjects were part of a big dynamic free-ranging dog population living as scavengers in and around human settlements, hence classifying as street dogs [20]. The behaviour battery was first piloted on a random sample of eight adult street dogs (4 males, 4 females). Since the test battery was finalized after the first three and thus remained the same for the other five pilot dogs, they were added to the final test sample. Hence, a total of 36 adult street dogs (20 males, 16 females; 5 pilot plus 31 test sample dogs) were tested to assess the feasibility and reliability of the finalized test. Twenty-six of those dogs (15 males, 11 females) were found again for the retest.

Procedure

The test battery consisted of six short behavioural subtests and three physiological samplings conducted in direct succession (Fig 1). The subtests were designed to resemble behaviour tests that have commonly been used to assess pet, shelter, or working dog temperament traits, dogs' cognitive abilities, or traits that have been suggested as central during the domestication process (see description of the respective subtests). The three physiological samples (saliva and hair) were part of the test battery to assess the feasibility of sampling in such a setting and allow for later use of those samples in future exploration.

Fig 1. The sequence of the employed test battery. Subtests in light and physiological sample collections in dark grey.

For the test-retest reliability measure, the test battery was conducted a second time ("retest") with the same study subjects within an average time span of 46.28 days (min. 33, max. 76 days) which was similar to other test validation procedures [e.g., 42, 43-45]. To this end, the location of the initial test was noted, and the area was frequently visited again after five to six weeks until the dog was found, identified with previous pictures, and tested again. It was noted if the dog was retested in a different location than the first test (i.e., not in visual vicinity of the previous location). The procedure in the first and retest was exactly the same as described below. For the object-based subtests (Fake dog and Novel object subtest, see below), two slightly

different-looking versions were used respectively for the test and the retest to create novelty. The order of presentation of the two stimuli was randomized across subjects.

Initiation and general circumstances

Two experimenters were involved in each testing occasion: E1 was always the same person (SC) and conducted the actual tests while E2 (three different people) video-recorded, helped set up the subtests, or distracted other dogs if necessary. Dogs were tested individually and had to be alone before and during the test. Tests were conducted between sunrise and 10 a.m. before the areas became too crowded. The experimenters accessed the study area by car and all dogs encountered alone were tested. If at any point during testing, the dog ran off more than 10 m, the subtest during which the dog left was terminated and E1 tried to lure the dog back. If the dog ran too far to be retrieved, or did not respond to the lure, the whole test was terminated. If anyone other than the experimenter approached and visibly distracted the dog (e.g., head turn), the test was interrupted until the criterium was restored and the test resumed. If at any point dogs showed active signs of aggression, the test was terminated (however, this never occurred). For each dog, location, time, weather, health status, and activity upon arrival of E1 (e.g., sleeping, walking, …) were noted.

The exact setup of each subtest is displayed in Fig 2, the procedure of each subtest is demonstrated in the supporting information (S1 Video).

Fig 2. The set-up of each of the six subtests in the presented test battery. The main experimenter (E1) is presented in black (or grey in later stages of the subtest), the assistant experimenter (E2) in white.

Human Approach Test (Subtest 1)

Target: Approachability, human-directed sociability/ docility, tameness, play. This test was modelled after similar tests used in widescale personality assessment tests (e.g., the Dog Mentality Assessment (DMA) in the Swedish Kennel Club [46] and has in part been used with Ethiopian village dogs [34]. Beside the assumption that human-directed sociability is an essential trait under selection during the domestication

process [2], assessing the actual human-directed behaviour in a free-ranging population is provides valuable insights for disease- and population control.

Here, E1 stepped out of the car and whistled shortly to grab the dog's attention. She then stepwise proceeded to 1) stand quietly looking at the subject for 30 s, 2) called the dog whilst making friendly gestures (snapping, tapping on leg) for another 30 s, 3) approached the dog for 30 s up to 2 m distance, 4) stood for another 30 s, 5) approached the dog to one body length distance and paused another 30 s, and 6) crouched for another 30 s all while continuing to call the dog over. If the dog approached to less than 10 cm of E1 during any of those phases, E1 kneeled and tried to pet the dog for 30 s. If tolerated, E1 used a small brush to collect hair samples. The dog was then invited to play tug of war with a rag toy (1 m long) for 30 s.

Fake Dog Test (Subtest 2)

Target: Conspecific-directed sociability and aggression. Several studies support the idea that stuffed dogs can be used to reliably assess dogs' initial behaviour towards conspecifics [37, 47]. A reduction in conspecific aggression has been hypothesised as an important shift from wild to domestic types [48], and understanding intraspecific aggression in free-ranging dogs can provide important applications for the local population.

Hence, subjects were exposed to a "Giant Jack Russel Terrier" stuffed fake dog (brand: 'Melissa & Douk'; wither height 28 cm; S2 Supplementary Information). The two used versions differed in ear posture (pointy or droopy), tail length (medium and long), and colour (one original, one wrapped in dark bags) in the test and in the re-test respectively, in a randomized order across subjects. The fake dog was positioned 5 m from the car behind a V-shape visual barrier with its opening towards the car. This visual barrier allowed only the approaching subject but no other surrounding dogs to see the fake dog. E1 guided the subject towards the fake dog (if needed with food) and quickly walked >5 m away as soon as the subject saw the fake dog. The subject's behaviour was observed for 2 min.

Novel Object Test (Subtest 3)

Target: Neophobia, ~~(~~exploration/~~,~~novelty-seeking~~, curiosity)~~. Presenting a dog with an unknow object is commonly used to assess novelty-seeking and boldness [41, 49], and explorative-behaviour is hypothesised to be lower in domesticated animals as part of the domestication syndrome [48].

To present an object that was truly novel to the dogs, we fixed a 90 – 100 cm high foil balloon with a wooden stick on top of a remotely controlled car (31x18x18 cm, Model: DEERC DE42 RC, S2 Supplementary information). Two balloon forms were used (dinosaur, a number (zero, five) sprayed in neon green and blue) for test and retest and randomized between study subjects. The set up was the same as the Fake dog test. As soon as the dog saw the novel object, E2 started moving it back and forth for 1 min through remote control. The toy was then left inactive for another 1 min. The dog's behaviour was observed for the full 2 min.

Pointing Test (Subtest 4)

Target: Dog-human communication skill, willingness to follow commands, cooperative communication [reviewed in 30, 50]. Studies have shown that a wide array of species, including canids, can follow pointing cues [for review see 50]. Exploring this phenomenon in free-ranging dogs with differing levels of human experience will further our understanding of the role of life-experience vs. species predisposition (but see [21] for pioneering work in this regard).

The test started with four warm-up trials: E1 placed a baited bowl (12 cm diameter, 10 cm height) on the ground 2 m from the dog, standing 1 m away herself and verbally called the dogs attention. After the dog ate the food, the bowl was picked up, baited, and placed down again to familiarize the dog with the fact that the bowl contained retrievable food. The subtest was terminated if the dog did not approach the bowl within 30 s two times in a row. The warm-up trials were followed by twelve test trials: one empty and one baited bowl (both rubbed in food for smell, baited outside of the dog's view) were placed 1.5 m apart 2 m from the dog. E1 stood equidistant behind the bowls, attracted the dog's attention, and upon eye contact, pointed to the baited bowl for 2 s (momentary distal pointing). The dog was allowed to eat the food if it approached the correct bowl on the first choice, otherwise the bowls were immediately collected by E1. Each trial could

be repeated twice if the dog did not choose within 30 s, followed by one repetition of the warm-up trial (i.e., only one bowl with food). If after the third repetition, the dog did not choose, the subtest was terminated. We used six different randomized sequences related to the positioning of the baited/pointed bowl to the right or left of the experimenter (e.g., RLRLLLRLRRLLR) that were called out to E1 by E2.

Begging Test (Subtest 5)

Target: human-directed communication, gazing behaviour, suggested to be help-seeking behaviour [38, reviewed by 39, but see for alternative explanations: 51, 52]. Also a behaviour suggested as a trait under selection during domestication but the wide variability between individuals depending on human socialisation calls for further investigation with differentially socialised populations [53, 54].

The dog was allowed to eat from the baited bowl three times. Afterwards, the bowl was visibly baited and placed out of reach but still visible on the car, a wall, or a tree. E1 stepped 1 m to the side and faced away from the bowl with a neutral facial expression. The dog's behaviour was observed for 1 min.

Tractability test (Subtest 6)

Target: Social inhibition, tractability/deferential behaviour (i.e., the ease to yield to higher command, conflict avoidance [3]). According to the deferential hypothesis [3], tractability has been a central selection factor in dog domestication. Furthermore, assessing dogs' inclination to avoid conflicts with humans in a resource scenario may also have it uses in a more applied setting.

E1 gave the dog a piece of food, then took a big piece of meat, showed it to the dog, and visibly placed it on top of a small stool (40 cm height). E1 loudly stepped between dog and stool, stared the dog in the eyes and firmly said "No, it's mine!". If the dog attempted to get to the stool, E1 loudly stepped in their way and repeated "No" in a firm tone. After 1 min of this, E1 stepped 1 m to the side and stood still for 30 s while looking at the food. Finally, E1 turned around and looked away for 30 s. The test was finished as soon as the dog ate the food or after the last 30 s.

Video coding

The tests were recorded by an action camera (4K 24fps WiFi Sports Action Camera or Action Cam Jeemak WiFi) fixed on E1's chest and a camera (Sony HDR-CX405 Full HD Camcorder, hand-held (subtest 1) or on a tripod (subtest 2-6)) handled by E2. BORIS (version 7.13.8, (Friard & Gamba, 2016)) was used for the video coding.

The ethogram aimed at capturing the target behaviours described for each subtest. We used a bottom-up approach by initially coding 55 variables with different modifiers (i.e., the person or object the behaviour was coded towards) that measured almost every potentially relevant aspect in the different subtests. The coded variables are displayed in Table 1 with a detailed ethogram in S3 Table. Social behaviours were based on the ethograms used at the Wolf Science Center of the University for Veterinary Medicine, Vienna [10], and to observe free-ranging dogs in Italy [16, 55].

This list of variables was narrowed down by 1) assessing their codability through the inter- and intra-rater analysis (for acceptable agreement see Statistical Analysis), 2) assessing the variables' centrality in the subtest by analysing their frequency (i.e., excluding variables that were displayed in less than 10% of tests), and 3) assessing their consistency through the test-retest reliability. Only behavioural variables that passed step 1) and 2) went on to be analysed in step 3) to ensure that the test-retest reliability was based on reliably coded and not overly skewed data [56].

Table 1. Coded behavioural variables and occurrence across subtests.

Category	Behavioural variable	Human approach	Fake Dog	Novel Object	Pointing	Begging	Tractability
**Proximity**	Close	E1	Fake dog, E1+E2	Novel object, E1+E2	-	E1	E1, stool
	Medium	E1	Fake dog, E1+E2	Novel object, E1+E2	-	E1	E1, stool
**Tail**	Wagging	E1	Fake dog	Novel object	E1	E1	E1
	Between legs	Yes	Yes	Yes	-	Yes	Yes
**Gazing**		E1	Fake dog	Novel object, E1+E2	-	E1	E1
**Vocalization**	Barking	E1	Fake dog	Novel object	-	E1	E1
	Growling	E1	Fake dog	Novel object	-	E1	E1
	Whining	E1	Fake dog	Novel object	-	E1	E1
**Displacement**	Nose-licking	Yes	Yes	Yes	-	Yes	Yes

	Body shake	Yes	Yes	Yes	-	Yes	Yes
	Stretching	Yes	Yes	Yes	-	Yes	Yes
	Yawning	Yes	Yes	Yes	-	Yes	Yes
	Sniffing the ground	Yes	-	-	-	-	-
**Physical contact**	Biting	E1	Fake dog	Novel object	-	E1	E1
	Body contact	E1	Fake dog	Novel object	-	E1	E1
	Jumping	E1	Fake dog	Novel object	-	E1	E1
	Licking	E1	Fake dog	Novel object	-	E1	E1
	Mouthing	E1	Fake dog	Novel object	-	E1	E1
	Pawing	E1	Fake dog	Novel object	-	E1	E1
	Sniffing	E1	Fake dog	Novel object	-	E1	E1
**Reactions**	Stand tall	E1	Fake dog	Novel object	-	E1	E1
	Bare teeth	E1	Fake dog	Novel object	-	E1	E1
	Lunge	E1	Fake dog	Novel object	-	E1	E1
	Head dip	E1	Fake dog	Novel object	-	E1	E1
	Belly exposure	Yes	Yes	Yes	-	Yes	Yes
	Flee	E1	Fake dog	Novel object	-	E1	E1
	Crouch	E1	Fake dog	Novel object	-	E1	E1
	Risk assessment	E1	Fake dog	Novel object	-	E1	E1
	Play	E1	Fake dog	Novel object	-	E1	E1
	Friendly	E1	Fake dog	Novel object	-	E1	E1
	Bow	E1	Fake dog	Novel object	-	E1	E1
**Marking**		Yes	Yes	Yes	-	Yes	Yes
**Defecating**		Yes	Yes	Yes	-	*Yes*	Yes
**Non-visible**		Yes	Yes	Yes	Yes	Yes	Yes
**Disturbance**		Yes	Yes	Yes	Yes	Yes	Yes
**Subtest specific variables**			Latency to approach fake dog	Latency to approach novel object	Observation of gesture: no/yes	Attempts to reach bowl	Latency to eat food
		Phase of first approach		Phase of first approach	Successful choice: no/yes		Phase of eating food
			Genital sniffing		No choice	2-way gaze alternation	
						3-way gaze alternation	

The table shows all behavioural variables (column 2) of a certain category (column 1) that were coded in the respective subtests (columns 3 - 8). The words in the subtest columns describe towards what person or object the behaviour was coded, while ‘yes’ means that the behaviour was coded regardless of direction, and a minus (-) indicates that the behaviour was not coded in the subtest. Behavioural variables were either coded as duration (D), frequency (F), occurrence (O), or latency since the beginning of the respective subtest (L). Coloured cells indicate that the variable occurred in less than 10 % of tests and was thus excluded from the test-retest analysis.

For the intra-rater reliability measures, a random subsample of 20% of the test videos (n=13; balanced between test and retest, full test batteries and tests that were terminated halfway through) were re-coded by the initial rater after three weeks. For the inter-rater reliability, a random subsample of 20% of the full-test videos (n=9) were coded independently by a second rater [41, for similar methods see 57]. Beforehand, the second rater was trained on three sample videos and the ethogram was further clarified where needed.

Statistical analysis

Inter- and intra-rater reliability was analysed for each behavioural variable summed across all subtests and modifiers to assess their codability. Test-retest reliability, on the other hand, was analysed for every single variable by modifier and subtest. Occurrence variables (e.g., in which phase the dog approached, termination, etc.) were converted into ordinal variables before analysis. To normalize the data for comparison between test and retest, the time the dog was not visible or a disturbance was coded was subtracted from the total time of the subtest of the individual, and the behavioural data was then divided by this normative time. All individual subtests in which more than 70% of the time was coded as disturbance and/or not visible were excluded from further analysis. In case of the test-retest reliability, the specific subtest in both the test and the retest were excluded to allow for fair comparison.

The statistical analysis was then conducted in R (Version 4.2.2 [58]). To account for the systematic errors between raters/rating occasions, intraclass correlation coefficients (ICCs) were calculated to assess the reliability measures [59, 60]. Additionally, considering that both raters/rating occasions coded the same videos and that the goal was to generalize the reliability results, a two-way random effects ANOVA with an absolute agreement estimate was used to assess the inter- and intra-rater reliability [reviewed by 61]. According to the classification by [62], an ICC below 0.5 is considered poor reliability, between 0.5 and 0.75 it is moderate, between 0.75 and 0.9 it is good, and between 0.9 and 1.0 the reliability is considered excellent. For a behaviour to be regarded as having acceptable coding reliability and move on to the test-retest analysis, the ICC threshold for the inter- and intra-rater reliability was set to a moderate 0.70 [63] which is in accordance with similar studies [weighted average of 0.68 in a review of six studies by 9, 0.77 in 64]. However, careful reconsideration was employed in case the threshold was just about missed by taking

both coding reliabilities (inter- and intra-rater) into consideration and examining the p-value (assuming significance level α=0.05). For the test-retest reliability in the repeated sample, a two-way mixed effects ANOVA with absolute agreement estimate was applied [62]. Since significance largely depends on sample size and significant test-retest reliabilities in pet dog tests commonly range around an ICC of 0.5 which would usually be classified as poor to moderate reliability [average ICC of 0.43 in a meta-analysis of 31 studies in 35, e.g., 0.58 in 36, 0.57 in 41], a test-retest reliability with a p-value below 0.05 rather than a specific ICC was chosen as reliability indicator. Only variables that were displayed in more than 10% of the testing occasions were included in the test-retest reliability to circumvent unreliable analysis of heavily zero-inflated and low-prevalence data [for a similar approach see 56, 63, 65]. For the ordinal variables, two-way ANOVA’s with absolute agreement estimate (equivalent to weighted Cohen~~s~~’s Kappa according to [66]) were used to analyse the coding and test-retest reliability. If the ICC was poor (i.e., below 0.5) for a certain variable, systematic biases in the measurements of both rating occasions were explored through scatter plots and variables were excluded if necessary. To control for the test-retest reliability being low due to disturbances during the tests, we conducted the same analysis again but excluded all individual subtests in which any disturbances were coded (46 of 235 subtests). Disturbance had been coded when the dog clearly turned its head towards an interfering human/dog/other animal for more than three seconds during a running subtest and while the test subject was visible on the video.

Lastly, because curiosity- or exploration-based tests may be more prone to lower consistency due to repeated exposure to similar stimuli [49], we tested if the dogs systematically showed a decreased reaction (exploration and fear) in the fake dog and novel object retest compared to the first test. To that end, applicable variables that were retained in the test-retest reliability (i.e., had good coding reliability and occurred in more than 10% of tests) were summed into an exploration-related and a fear-related variable [therein drawing from 67, 68] for each of the two subtests respectively. After transforming these four left-skewed variables with a square root transformation, a paired t-test was used to compare whether they differed significantly between test and retest.

Results

Subject characteristics

Out of the 36 initially tested street dogs, 23 (14 males, 9 females) were tested in the entire test battery, while nine dogs (5 males, 4 females) terminated the test during the Human approach subtest and four dogs (1 male, 3 female) after the first two or three subtests by running away. For the retest, 26 and thus 72% of the 36 initially tested street dogs were found again: 19 of those were dogs who went through the full test battery initially and 7 were dogs who had run away during the first testing. Fourteen were retested in a different location than the first test. All 26 dogs completed the same number of subtests in the retest as they did in the initial test.

Changes in methods and variables

After the test was adapted in the pilot testing, some additional changes from the above-presented procedure had to be made throughout the main testing period. For one, the play-part (rope presentation for tug-of-war) in the Human approach test caused four (28.57%) of the first 14 dogs to run away and terminate the test. For two more, the play phase had to be terminated to keep the dog from leaving. Thus, the play phase was dropped half-way through the main sample and the variable 'Interacting with the toy' was excluded from the analysis.

In addition, two more variables had to be excluded due to the following reasons: 'Auto-grooming' because the dogs had varying amounts of parasites, wounds, and dirt in their fur which influenced the scratching rate, and 'Sniffing the ground' in all but the Human approach test because food was dropped on the ground during the subtests and the different ground surfaces influenced the dogs' sniffing behaviour.

Intra- and inter-rater reliability

Both the intra- and inter-rater reliability coding, assessed across all subtests and modifiers, is reported in Table 2. Almost all variables passed the target ICC-threshold of 0.7 and had a significant p-value at 0.015 or below (the exact statistical outputs for each variable are reported in S4 table). The average of both inter-

and intra-rater reliability for all variables was above the target threshold of an ICC of 0.7, with a good reliability of 0.84 in the inter-rater and 0.93 in the intra-rater analysis.

There were only few variables that did not clear the threshold. ‘Tail between the legs’ and ‘Mouthing’ had moderate inter-rater reliability, meaning they still had an ICC above 0.5, and the p-value was below 0.05. Likewise, ‘Medium proximity’ had a moderate reliability within and between raters. The inter-rater reliability for ‘Head dip’, ‘Friendly’ and ‘Stand tall’, on the other hand, were poor with an ICC below 0.5 with a p-value above 0.05.

While ‘Tail between the legs’ and ‘Mouthing’ did not clear the initially defined threshold, they were both close and had a p-value of 0.02 which is substantially below the usual 0.05 significance level. Since previous studies have deterred from the arbitrary 0.7 ICC threshold in favour of significant p-values (p<0.05) [9], and both present crucial behaviours in dogs, we tentatively chose to keep them in the analysis going forward but treat their interpretation with utmost care. The other behaviours fell below the 0.05 significance level and were thus excluded from further analysis.

Table 2. Summary of the coding reliability across subtests.

**GENERAL**			
**Category**	**Behavioural variable**	**Inter-rater ICC**	**Intra-rater ICC**
**Proximity**	Close	0.90	0.97
	Medium	0.53	0.52
**Tail**	Wagging	0.91	0.91
	Between legs	0.64	0.99
**Gazing**		0.90	0.98
**Vocalization**	Barking	NA	NA
	Growling	0.80	NA
	Whining	NA	NA
**Displacement**	Nose-licking	0.88	0.96
	Shaking	0.80	NA
	Stretching	0.88	1.00
	Yawning	0.94	0.97

**SUBTEST SPECIFIC**			
**Subtest**	**Behavioural variable**	**Inter-rater ICC**	**Intra-rater ICC**
**Human approach**	Phase of first Approach	1.00	1.00
**Fake Dog**	Latency	1.00	0.97
	Genital sniffing	0.92	1.00
**Novel object**	Phase of first approach	1.00	1.00
	Latency	0.85	1.00
**Pointing**	Observation of gesture: yes	1.00	0.98
	Observation of gesture: no	1.00	0.99
	Successful choice	1.00	1.00
	Unsuccessful choice	1.00	1.00
	No choice	1.00	1.00
**Begging**	Attempts to get bowl	0.99	0.99
**Begging/ Tractability**	2-way gaze alternations	0.82	0.79

	Sniffing the ground	0.82	0.79
**Physical contact**	Biting	NA	NA
	Body contact	1.00	0.74
	Jumping	0.95	0.98
	Licking	NA	0.84
	Mouthing	0.64	0.95
	Pawing	NA	0.99
	Sniffing	0.97	0.98
**Reactions**	Stand tall	-0.03	0.91
	Bare teeth	NA	NA
	Lunge	NA	NA
	Head dip	-0.08	0.85
	Belly exposure	NA	NA
	Flee	0.77	0.94
	Crouch	0.90	0.79
	Risk assessment	0.99	0.86
	Play	0.98	0.98
	Friendly	0.44	0.97
	Bow	0.81	0.76
**Marking**		NA	0.99
**Defecating**		NA	NA
	3-way gaze alternations	0.86	0.87
**Tractability**	Latency	0.90	1.00
	Phase of eating	1.00	1.00
**Average**		**0.84**	**0.93**

For each variable, the inter- and intra-rater reliability analysed across subtests and modifiers is displayed. Based on similar studies, a coding reliability of an ICC of 0.7 or above was regarded as acceptable (values with no specific highlight). Moderate reliability below this threshold (i.e., 0.5<ICC<0.7) is highlighted in light grey, and poor reliability (i.e., ICC<0.5) is marked in dark grey. All variables above an ICC of 0.7 had a p-value below 0.015, variables with an ICC between 0.5 and 0.7 had a p-value below 0.05, and all variables below an ICC of 0.5 had a non-significant p-value (p>0.05). Detailed analysis results with the 95% confidence interval, F-value statistic, degrees of freedom, and p-value can be found in S4 Table. Variables marked with NA did not occur in the coded tests.

Exclusion of infrequent variables

In the next step, variables that did not occur in more than 10% of the respective subtests were identified (Table 1) and excluded from the test-retest reliability analysis. In short, close proximity, tail wagging, gazing, nose licking and yawning were the only variables that occurred frequently in all subtests. On the

other hand, growling, whining, stretching, shaking, biting, licking, mouthing, marking, defecating, baring teeth, lunging, belly exposure, and bow were not displayed in more than 10% of tests in any of the subtests. Even after summing most of them into one pooled 'stress' variable [following the classification of 69, 70], they did not pass the 10% occurrence threshold and were hence excluded from the test-retest analysis. All subtest-specific variables except 'attempt to reach the bowl' in the begging test, occurred frequently enough to pass the 10% threshold.

Test-retest reliability

The final test-retest reliability analysis was conducted for each variable by subtest and modifier, only taking the variables into account that 1) were not already dropped from the ethogram/tests throughout the testing (i.e., auto-grooming, sniffing the ground, play), 2) had an acceptable coding reliability, and 3) occurred in more than 10% of all tests. The resulting test-retest reliabilities are reported in Table 3.

The average test-retest reliability was poor with an average ICC of 0.23. To see whether the poor outcome was influenced by disturbances during the tests that altered the dogs' behaviour, we conducted the same analysis but excluded all subtests in which a disturbance had been coded (i.e., when the dog clearly turned its head towards an interfering human/dog/other animal for more than three seconds during a running subtest and while the test subject was visible on the video). While this changed the reliability of some behaviours, the average test-retest reliability did not improve (Table 3; the exact statistical outputs of both analyses can be found in S5 Table).

To summarize by subtest, the Human approach test performed well with ten out of 15 behaviours showing significant test-retest reliability, resulting in an average test-retest reliability of 0.32. In the Fake dog test, only 'tail between the legs' had a moderate retest reliability, with 'close proximity to the fake dog' and 'nose-mouth licking' also being moderately reliable after tests with disturbances were removed. The average test-retest reliability was low (ICC = 0.06). Similarly, only 'close proximity to E1 and E2' were significant in the Novel object test, being joined by 'nose-mouth licking' after disturbed tests were excluded, resulting in an average test-retest reliability of 0.12. The average reliability of the Pointing test was significant with

an average ICC of 0.51 with all subtest-specific variables, as well as ‘tail-wagging’, showing moderate test-retest reliability. The Begging test had a low test-retest reliability (average ICC = 0.16) with only the ‘close proximity to E1’ being significant across time when only looking at tests without disturbances. Lastly, ‘close proximity to E1’, ‘gazing at E1’, the ‘phase in which the dog ate the food’, ‘three-way gaze alternation between person and stool’ and ‘fleeing’ had significant reliability in the Tractability test, with the addition of ‘nose-mouth licking’ after disturbances were removed. The average test-retest reliability was 0.22.

Test-retest comparison for the curiosity-based subtests

The summed variables constituted of the following behaviours: “Exploration”-variable: ‘close’ and ‘medium proximity’, ‘gazing’, ‘tail wagging’, ‘friendly’, and ‘sniffing’ the fake dog/novel object, as well as ‘genital sniffing’ in the case of the fake dog. “Fear”-variable: ‘head dip’ and ‘tail between the legs’ for the fake dog subtest. Only ‘flee’ was retained as fear-representing behaviour in the novel object but was displayed so rarely, that it could not be analysed here.

The dogs showed significantly less exploration and less fear behaviours towards the fake dog in the retest than the first test (exploration: $t(14)=3.16$, $p<0.01$; fear: $t(15)=-3.08$, $p<0.01$). Exploration behaviours shown towards the novel object did not differ between test and retest ($t(11)=1.0$, $p=0.34$).

Table 3. Summary of the test-retest reliability in all retained variables.

Category	Behavioural variable	Human approach			Fake Dog			Novel Object			Pointing			Begging			Tractability		
		**M.**	**All**	**w/D**	**M.**	**All**	**w/D**	**M.**	**All**	**w/D**	**M.**	**All**	**w/D**	**M.**	**All**	**w/D**	**M.**	**All**	**w/D**
**Proximity**	Close	*E1*	0.71	0.77	*FD*	0.20	0.74	*NO*	-0.09	0.06	-			*E1*	0.8	0.76	*E1*	0.75	0.72
					E1+E2	-0.08	-0.17	*E1+E2*	0.74	0							*Stool*	-0.04	-0.02
**Tail**	Wagging	*E1*	0.61	0.62	*FD*	0.14	-0.12	*NO*	0.04	NA	*E1*	0.5	0.2	*E1*	0.36	0.3	*E1*	-0.14	-0.06
	Between legs	*Yes*	0.53	0.52	*Yes*	0.71	0.72											0.29	0.28
**Gazing**		*E1*	**0.35**	0.34	*FD*	0.16	0.11	*NO*	-0.35	-0.06				*E1*	0	0.24	*E1*	**0.48**	0.44
								E1+E2	-0.26	-0.08				*Bowl*	0.34	0.12	*Stool*	0.18	0.14
**Vocalization**	Barking	*E1*	0.85	0.85	*FD*	0.12	0.12												
**Displacement**	Nose-licking	*Yes*	0	-0.01	*Yes*	0.16	0.56	*Yes*	0.37	0.61	-			*Yes*	-0.2	-0.28	*Yes*	0.41	0.51
	Yawning	*Yes*	-0.03	-0.08	*Yes*	-0.06	0	*Yes*	0.48	0.45	-			*Yes*	-0.09	-0.1	*Yes*	-0.21	-0.2
	sniffing the ground	*Yes*	-0.04	-0.04	*Excl.*			*Excl.*			-			*Excl.*			*Excl.*		
**Physical contact**	Body contact	*E1*	0.55	0.55															
	Jumping	*E1*	0.5	**0.46**															
	Sniffing	*E1*			*FD*	0.03	0.19	*NO*	-0.04	0	-			*E1*	-0.09	-0.05			
**Reactions**	Flee	*E1*	-0.08	-0.16				*NO*	0.32	NA							*E1*	0.76	0.58
	Crouch																*E1*	-0.05	-0.02
	Friendly	*E1*	**0.45**	0.56	*FD*	-0.07	0.15							*E1*	0.34	0.35	*E1*	0.13	0.14
	Head dip	*E1*	-0.08	-0.09	*FD*	-0.21	0	*NO*	0	NA							*E1*	-0.07	-0.02
	Play	*E1*	-0.03	-0.06															
	Risk assesment				*FD*	-0.04	-0.04												
	Stand tall				*FD*	-0.12	-0.29												
**Subtest-specific variables**	Phase of first Approach	*E1*	**0.49**	0.51				*NO*	0.2	0	-			-			*Stool*	0.65	0.75
	Latency of first approach				*FD*	-0.15	0.2	*NO*	0.05	-0.9	-			-			*Stool*	-0.11	-0.08
	Genital sniffing				*FD*	0.15	-0.15												

	Observation of gesture: yes	-			-			-			*E1*	0.51	0.12	-			-		
	Observation of gesture: no	-			-			-			*E1*	**0.33**	-0.2	-			-		
	Successful choice	-			-			-			*Bowl*	0.64	0.38	-			-		
	Unsuccessful choice	-			-			-			*Bowl*	0.55	0.06	-			-		
	No choice	-			-			-			*Bowl*	0.52	0.17	-			-		
**Gaze alternations**	2-way	-			-			-			-			*E1/ bowl*	0.03	-0.08	*E1/ stool*	0.14	0.07
	3-way	-			-			-			-			*E1/ bowl*	0.1	0.08	*E1/ stool*	**0.38**	**0.38**
**Average per subtest**			**0.32**	**0.32**		**0.06**	**0.13**		**0.12**	**0.10**		**0.51**	**0.12**		**0.16**	**0.13**		**0.22**	**0.23**
**Average across the test battery**			**0.23**	**0.17**															

For each variable and subtest, the modifier (M.) and the test-retest reliability is displayed for all tests (All) and for only the tests did not have any coded disturbances (w/D) respectively. The modifiers were the main experimenter (E1), the fake dog (FD), the novel object (NO), the bowl, or the stool. Test-retest reliabilities with a p-value below 0.05 were regarded as acceptable. Behaviours with non-significant test-retest reliability are indicated with a light-grey background and in bold if they were significant but regarded as poor reliability (i.e., below an ICC of 0.5). Variables that were excluded due to low occurrence (i.e., less than 10% of testing occasions) are not shown unless their exclusion only applied to certain subtests. In that case, they are left empty or marked with NA if it only applies to the analysis without disturbances. Variables marked with a minus (-) were not coded in the respective subtest. Detailed analysis results with the 95% confidence interval, F-value statistic, and p-value can be found in S5 Table.

Discussion

In this study, we presented what, to our knowledge, is the first attempt at testing free-ranging dogs in a behavioural test battery and report feasibility and reliability of the test measures. Testing the dogs in their natural environment was indeed feasible and the coding reliability was high, indicating that this (relative to previous studies) long and complex behaviour test battery can be conducted with street dogs. The range from good to poor test-retest reliability, on the other hand, reflects the challenges inherent in conducting ~~dog~~ behaviour tests ~~conducted~~ with free-ranging ~~animals~~ dogs on the streets. ~~are faced with and we provide possible explanations and further recommendations for the use of this test battery and behavioural testing of free-ranging dogs in general.~~

Although the subtests in this test battery were adapted from mostly ‘lab-based’ ~~enclosed, standardized pet and working dog~~ behaviour assessments [e.g., 37, 41, 49] [but see 21], ~~bringing the test to the free-roaming dogs~~ and standardizing the set up and testing our dog population in the streets ~~and beach environments of our populations~~ turned out to be surprisingly manageable. Importantly, ~~, and~~ there were no terminations due to aggression towards people or equipment and~~. More importantly,~~ most dogs completed all six subtests and sample collections despite an average test duration of 30 minutes, suggesting that street dogs (at least those of this population) can remain motivated throughout longer and more complex procedures than those used so far [e.g., 19, 33, 34, 71].

Similarly, the concern that testing and filming in the variable street conditions would negatively impact the coding reliability was unfounded. Our results surpassed the target of a mean ICC of 0.7 with good average inter- and intra-rater reliability of 0.84 and 0.93 respectively. Contrary to our expectations, the coding reliability in this street test was thereby *on par* or even better than many pet and working dog studies [ICC 0.68 in meta-analysis by 9, ICC >0.9 in 41, ICC 0.77 in 64], suggesting that our chosen variables were not only well defined but could also be coded reliably despite the more challenging ~~street~~ conditions. Similarly encouraging inter-rater results have been reported for a few other street dog tests as well [19, 21], further highlighting~~underlining~~ that such results are achievable for street testing in general.

However, when looking at the reliability of the individual variables, a few did not meet the threshold. Notably, 'medium proximity', i.e., two body lengths distance from ~~the dog to~~ the experimenter ~~or object of interest~~, was the only ~~one that was~~variable which was low both in the ~~on both~~ inter- and intra-rater reliability analyses. With the constant adaptation of the camera angles to adjust for the variability of the street conditions, it might thus be best to stick to distances that are easier to identify such as the more reliable 'close proximity' (i.e., one body length distance). Additionally, 'head dip', 'stand tall', and 'friendly' all had poor agreement between the raters, which might be due to the complex~~ity~~ definitions of these behaviours~~, with their definitions relying on a variety of subtle behaviours and body postures exhibited together~~ (~~for the detailed definitions see~~ S3 Table). While these definitions were adapted from previously used ethograms [17, 72, 73], the filming in street conditions likely made the subtle cues harder to detect ~~or the behaviours might have been misinterpreted due to the difference from the natural conspecific setting they were initially defined in~~. Given the poor coding reliability of those traits, they should thus be dropped from the presented ethogram unless a more reliable definition or training method can be found and validated, and care should be taken in the establishment of future tests to balance between the complexity of target behaviours and filming feasibility in the streets.

While these outcomes ~~so far~~ paint a positive picture for street dog testing so far, the feasibility in terms of capturing the desired traits cannot be claimed without looking at the test's ability to elicit the same behaviours consistently over time. The presented test battery resulted in an average test-retest reliability of 0.23, which, according to [62], is regarded as poor reliability. While the definition of personality traits is firmly bound to their consistency over time and across contexts [9], capturing such behavioural consistency has been an issue for many test batteries. A meta-analysis of 31 studies revealed an average trait consistency of 0.43 in pet, shelter and working dogs, which was considered significant although the studies within displayed highly variable results ranging from -0.73 to 1.00 [35]. We found a similarly high variability in the test-retest reliability of the behaviours we assessed, with subtest reliability varying between a moderate ICC of 0.51 for the pointing test and a poorly reliable 0.06 for the fake dog test. Capturing consistent

personality or behavioural scores over time is hence an issue not unique to the testing of street dogs in their natural habitat.

Intriguingly, most of the variables that were reliable over time were human-directed behaviours, including proximity to and tail wagging at the experimenter, body contact, and time of first approach. Likewise, the subtests in which the experimenter constantly interacted with the dog (i.e., human approach, pointing, tractability test) had the ~~most reliable variables and~~ highest average test-retest reliability over time. ~~This is~~Our results are consistent ~~in accordance~~ with other studies that report high reliability and strong heritability for sociability and biddability in pet dogs [e.g., 41, 49, 77] as well as dogs' performance during pointing tasks [76, 78], which~~, with the same consistency result in free-ranging dogs,~~ might tentatively point to a genetic basis for these traits ~~on which the selection process may have acted upon during dog domestication~~ [2]. Simultaneously, while it has been shown that dogs already follow such pointing cues as pups [74] and that their performance is stable regardless of keeping condition, time spent with the owner, or training [76], ~~,~~we cannot rule out that the individuals' life experience with human food provisioning played a part in these stable outcomes (as human socialisation seems to play a big part in pointing performance in other species [e.g., wolves: 74, goats: 75]).

More important in terms of street dog testing, however, is that the discrepancy in reliability between these social tests ~~and behaviours~~ and the other subtests (fake dog, novel object, and begging test in particular) might be driven by the~~ir~~ former's high interactivity, leaving the animal little time to be distracted. Contrary to pet dogs, ~~F~~free-ranging dogs have been shown to gaze at an inattentive person ~~that does not attend to them directly~~ less than at an attentive person ~~while such a difference does not occur in pet dogs~~ [33]. Moreover, ~~the reality of~~ testing in the free-ranging dogs' natural habitat means testing in highly variable and frequently disturbed environments~~an often highly chaotic environment, including frequent disturbances and environmental variations~~. W~~hile we~~e tried to account for this by excluding all subtests with coded disturbances in a second analysis, however this did not improve reliability, possibly ~~One reason might be that our choice of coding disturbances only when the dog was visible and attending to the distraction for several seconds~~because we underrepresented~~the influence of the~~ the disturbances to our~~the~~ study subjects.

Indeed, only six of the 35 dogs were alone in both tests (i.e., initial test and retest). For all others, additional dogs appeared during the test(s) ~~entered the testing area throughout one or both testing occasions~~ and had to be ‘distracted away’ by the second experimenter. The appearance of other dogs is an unavoidable occurrence, and whilst testing can continue in such cases, it ~~Besides the probable influence of those disturbances on the overall retest reliability, they~~ might explain why the subtests in which the dog had more time to focus on their environment without the direct engagement by the experimenter (fake dog, novel object, begging) showed a lower retest reliability. Whether behaviours like gazing and yawning and the entire begging test~~,~~ (which does not only lack interaction but also novelty)~~,~~ can reliably reflect a dog’s behaviour under such circumstances is hence questionable and requires further rigorous testing in alternative test setups on the streets.

To address the disturbance problem on the streets, we propose four not mutually exclusive approaches: 1) Have at least one additional person on the team that is solely responsible for~~to~~ distracting approaching dogs out of sight. 2) Allow for longer between-subtest-phases if the disturbance is temporary and let the dog return to an attentive state rather than pushing through the test. However, one should keep in mind that this changes the time dogs spend in the overall test procedure and carefully consider an influence on the respective goal. 3) If the distraction is substantial, terminating the test and either repeating (though the measures from the initial testing should be used for the repeated subtests) or resuming the test on a different day might be an option, depending on the respective test set up, goal, and familiarity with the population. 4) Meticulously coding any distraction might allow for correction of the disturbances, or at least provide deeper understanding of their influence on the behaviour, based on which decisions for future testing can be made.

Even after considering the disturbance problem, the consistency of the dogs’ behaviour in the fake dog and novel object tests was notably low. This might seem surprising since both tests are regularly used in dog temperament assessments [e.g., 37, 65]. However, we could not find any reference to the retest reliability of fake dog tests, and even highly standardised studies exploring novel object tests and novelty-seeking and fearlessness traits ~~in general~~ often showed ~~that their~~poor test-retest reliability ~~even in highly standardised~~

~~settings is often poor~~ (i.e., ICC<0.50) [e.g., Novelty-seeking: 0.48 in 41, Environmental Sureness: 0.16 in 64]. Accordingly, it has been proposed that test-retest outcomes are sensitive to the novelty of the object and situation due to learning effects [49]. Indeed, the fact that the free-ranging dogs explored the fake dog significantly more in the initial test while showing less fear in the retest supports the assumption that the animals partially remembered the initial encounter and adapted their behaviour accordingly. Although we made efforts to modify the appearance of the fake dog and novel object for the retest, the situation, procedure, and set-up remained the same and seem to have lacked the novelty needed to "fool" the animals twice within just six weeks. Nevertheless, it needs to be stressed that the street dogs frequently displayed appropriate conspecific, startling, and investigation behaviour in the initial presentation. ~~-~~Additionally considering that those subtests have been validated with fake vs. real dogs in case of the fake dog test [37, 47] and deemed reliable despite similar retest problems in case of the novel object [e.g., 41, 49], we suggest that these two subtests are still suitable to test the street dogs when only a single exposure to the test is needed. ~~Particularly~~ Additionally for the fake dog test, the social behaviours towards the stimulus were mostly exhibited in the first half of the subtest, suggesting that it is the initial reaction to the fake dog that is the most representative of conspecific behaviours. ~~however, the rather short interaction time most dogs showed with the fake dog suggests that~~ A~~a~~ shorter ~~exposure (i.e.,~~ test duration~~)~~ and/or movement in the fake dog [e.g., 47] ~~might have to~~could thus be considered to decrease not only the influence of distractions ~~possibilities~~, but also the risk that neophobia and boldness traits replace the conspecific behaviours as soon as the dogs realize that the fake dog is not in fact real.

At the same time, these two subtests act as a reminder of the importance of appropriate between-test-intervals, particularly for test batteries investigating behavioural profiles. With free-ranging dogs, the balance between waiting out potential learning effects and retesting early enough to find a considerable number of test subjects again can be difficult, especially if the study population is not well-known. ~~Turnover rates can be highly variable between locations and while we were familiar with the dynamics in the surrounding area [20], we had not yet worked with this particular population of street dogs. In addition, more than half of our study subjects were retested in a different location. The~~ Our substantial retesting rate

of 72% within an average of six weeks was hence surprising, though the considerable variation between the individuals (min. 33, max. 76 days) has to be acknowledged. Meticulous notes on appearance, ~~exact finding~~ location, and particular behaviours were crucial in this effort~~, not only for retesting but also to avoid testing of the same dog as a different individual~~. While working with a well-known population would streamline such efforts, our results show that it is not a prerequisite. In addition, using fewer memorable cues (e.g., the V-shaped wooden board in the Fake dog and Novel object subtest) might reduce learning effects and allow for a shorter between-test-interval with better outcomes than presented here.

Beside these two probable explanations for the low test-retest reliability in some subtests, an additional – though highly speculative – explanation might be that free-ranging dogs could be less genetically fixed in some behavioural traits than modern breed dogs and thus behave less consistently in general. This idea might not be too farfetched, considering that ~~breed dogs have been selected for specific characteristics and have lost considerable genetic variation along the way~~ [79]~~, whereas~~ free-ranging dogs are still subject to free mate choice and the instability of their environment [14]. Behavioural plasticity for certain traits (e.g., adaptiveness to the highly dynamic group compositions, weather conditions, or anthropogenic changes) might therefore be a ~~much more useful~~valuable and thus widely retained trait in free-ranging dogs to deal with those~~e~~ constant environmental changes~~ in their environment~~. Meanwhile, the directional selection for certain reliable breed characteristics in modern breed dogs [80, 81] and the resulting considerable loss of genetic variation [79] ~~, while it~~ might have ~~been selected against~~decreased behavioural plasticity in our modern breeds. Indeed, the study of reaction norms in wild fish and birds ~~has~~ provides~~d~~ cautious evidence that behavioural plasticity (i.e., intra-individual variability) can have a heritable component [82, 83]. And though a meta-analysis targeting the question whether behaviour-selected working-breed dogs were more fixed in their behaviour than non-selected non-working breed dogs could not find any support for this hypothesis [35], it was reported that an unstable rearing environment does not seem to have an effect on the consistency of behaviour, further highlighting the stability of certain traits in breed dogs [76]. Exploring how this compares to free-ranging dogs is an exciting avenue for further research.

A last consideration regarding the test itself pertains to the exclusion of the play component in the Human approach test and what that teaches us about the need to consider ~~your~~one's study subject in all its facets. Play is frequently used in temperament tests to assess sociability [36, 40, 46], and ~~it~~ the retainment of play behaviour into adulthood has also been suggested as a consequence of domestication in several species~~a potential part of the domestication syndrome in terms of neotenic behaviour~~ [reviewed in 48, 84]. When applying this subtest with the street dogs, the low engagement rate and ~~often~~ frequent fearful behaviour ~~of the animal~~ towards the toy might be explained by the negative or lack of ~~or negative~~ experience such unowned free-ranging scavengers had with pulling on an object with a human [23]. ~~In a similar incident~~Similarly, [19] ~~found~~ suggested that free-ranging dogs' lower persistence in comparison to pet dogs was likely due to their need to preserve energy and a lack of experience with human-manipulated items. ~~Alternatively, the recent findings that both ancient breeds [85] and wolf hybrids [86] also express lower levels of human-directed play behaviour than most modern breeds might suggest that a selection for human-directed play has only relatively recently taken place in modern breed dogs (e.g. as reward use in working dogs [87] or bonding experience between dogs and humans [84]). In addition~~On the other hand, our anecdotal observation with free-ranging populations indicates multiple incidents of conspecific and object play~~, running after birds or balls seemingly for fun, and 'playing' with the bowl during the pointing test~~. Whether play behaviour does indeed persist into adulthood in free-ranging dogs in the conspecific context or solitary play, but not with a human partner, or whether our choice of method was just ill-considered, remains to explored and emphasizes how carefully the subject population, its ecology and life experience must be considered despite our extensive experience with the species itself.

Finally, ~~there are~~ two important limitations ~~that~~ need to be addressed in terms of free-ranging dog testing as well as the representativeness of this study. First, the reliance on voluntary participation when ~~consideration that~~ testing adult free-ranging dogs ~~must rely on voluntary participation~~ creates a natural bias for more social animals, especially in longer and more interactive tests, and might be particularly challenging for feral populations (i.e., not reliant on human settlements). This bias is somewhat reduced in our Human approach test since the only selection criteria for starting a test was that the dog was seemingly alone. ~~Thus at least~~

~~the first steps of the test consisting of calling the dog and attempting a friendly approach were carried out with all dogs we found alone. If a dog then fearfully ran away as soon as we attempted the approach, ~~Hence, even the dogs that ran away fearfully upon approach ~~this was~~were analysed as part of our dataset. ~~Although a minority, these dogs are part of our population and ~~T~~t~~he fact that we were able to retest more than half of these 'escaping' individuals and they manifested the same behaviour on second testing provides evidence that this measurement was indeed reliable. Nevertheless, ~~it is the case that ~~these individuals~~overly fearful animals ~~ were lost for all the following subtests and are therefore not part of the majority of the reliability analysis. ~~Similarly, [21] found in a testing effort with 160 street dogs that half of their testing subjects could be classified as fearful and that these dogs were significantly less likely to approach and choose in a pointing task than neutral and affiliative dogs. ~~At the same time, ~~any ~~constraining ~~of ~~a free-ranging animal ~~during a test ~~would undoubtedly alter its natural behaviour in a test situation~~influence its behaviour to a point where it cannot be regarded as natural anymore~~. Explorable options to test such dogs could be the use of shorter tests with little human presence wherever possible (e.g., reaction- or exploration-based tests) or testing populations that now live in a fenced shelter but genetically belong to the clade of free-ranging dogs and had spent most of their lives roaming freely. However, the influence of recent life experiences and social context in the shelter ~~with the latter option, whether the influence of life experience and current social contexts might have an influence ~~on the target behaviour ha~~s~~ve to be strongly considered [22]. Secondly, the fact that our study population has previously been recognized as exceptionally friendly [20] ~~kept us from being able~~did not allow us to analyse the reliability of aggressive ~~behaviours ~~and many stress-related behaviours. For example, only 16.7% of our sample fled immediately ~~ran away during the sociability test~~ and none showed aggression, while more than 50% of an Ethiopian village dog sample fled upon approach and 11% reacted aggressively (though one needs to consider that these dogs were not always tested alone or outside [34]). While expanding the presented~~is~~ test battery to other populations ~~might be a way to start~~would initiate the ~~to ~~characteris~~ze~~ation of different free-ranging dog populations and what may affect differences in their behavioural profiles, we acknowledge that these limitations are likely difficult to be overcome. ~~-~~E~~xhibition of e~~xtreme fear or the slightest sign of human-directed aggression will necessarily

lead to aborting the test (particularly considering the risk of lethal disease, [e.g., 18]). And while using a fake dog rather than a real dog was indeed an attempt to test for the presence of aggression in a safe way, collecting additional~~complementary~~ observational data (regarding both human and conspecific directed behaviour) of the same individuals may be a complementary method allowing for multiple measures of specific behavioural traits.

In conclusion, the easy implementation, successful participation of most ~~free-ranging~~street dogs, and the high coding reliability presented here confirm that free-ranging dogs can be tested in longer test batteries with complex ethograms despite the challenging ~~street~~ natural conditions. The wide variability in the test-retest reliability across subtests demonstrate that the management of disturbances, the choice of set up and test-retest intervals, and careful considerations of the ~~study~~ subjects' life experience and ecology are central challenges when ~~behavioural tests with~~testing free-ranging dogs in their natural habitat~~ face~~. At the same time, the acceptable ~~considerable~~ reliability in some of the subtest demonstrates that under well-chosen conditions, testing ~~street~~ those populations~~dogs~~ is possible and opens a vast array of intriguing pathways to explore [22]. Bearing in mind the suggestions made in the discussion~~ in mind~~, going forward the test battery presented here can be a valuable method to test questions about cognitive traits, behavioural profiles, and domestication in such an understudied population as free-ranging dogs.

Acknowledgements

We thank Professor Ikhlass El Berbri for her support and collaboration in this project. Additionally, we thank the rest of our team in Morocco (in particular Andreas Berghänel, Haytem Bouchri, Manon Delaunay, and Magdalena Juskaite) without whom the project would not have been possible. We are also grateful to the population and authorities in the Sous-Massa region for supporting our field work. Finally, we want to extend our gratitude to the two reviewers for their valuable feedback and suggestions.

Bibliography

1. Trut LN. Early Canid Domestication: The Farm-Fox Experiment: Foxes bred for tamability in a 40-year experiment exhibit remarkable transformations that suggest an interplay between behavioral genetics and development. American Scientist. 1999;87(2):160-9.

2. von Holdt BM, Shuldiner E, Koch IJ, Kartzinel RY, Hogan A, Brubaker L, et al. Structural variants in genes associated with human Williams-Beuren syndrome underlie stereotypical hypersociability in domestic dogs. Science advances. 2017;3(7):e1700398. doi: https://doi.org/10.1126/sciadv.1700398.
3. Range F, Marshall-Pescini S, Kratz C, Virányi Z. Wolves lead and dogs follow, but they both cooperate with humans. Scientific reports. 2019;9(1):1-10. doi: https://doi.org/10.1038/s41598-019-40468-y.
4. Hare B, Tomasello M. Human-like social skills in dogs? Trends in cognitive sciences. 2005;9(9):439-44. doi: https://doi.org/10.1016/j.tics.2005.07.003.
5. Darwin C. The descent of Man; and on Selection in relation to Sex: J. Murray; 1871.
6. Pavlov IP. The scientific investigation of the psychical faculties or processes in the higher animals. Science. 1906;24(620):613-9.
7. Udell MA, Wynne CD. A review of domestic dogs'(Canis familiaris) human-like behaviors: or why behavior analysts should stop worrying and love their dogs. Journal of the experimental analysis of behavior. 2008;89(2):247-61. doi: https://doi.org/10.1901/jeab.2008.89-247.
8. Bensky MK, Gosling SD, Sinn DL. The world from a dog's point of view: a review and synthesis of dog cognition research. Advances in the Study of Behavior. 2013;45:209-406.
9. Gartner MC. Pet personality: A review. Personality and Individual Differences. 2015;75:102-13. doi: https://doi.org/10.1016/j.paid.2014.10.042.
10. Range F, Marshall-Pescini S. Domestication Hypotheses Relating to Behaviour and Cognition: Which Are Supported by the Current Data? Wolves and Dogs: Springer; 2022. p. 335-73.
11. Creevy KE, Akey JM, Kaeberlein M, Promislow DE. An open science study of ageing in companion dogs. Nature. 2022;602(7895):51-7. doi: https://doi.org/10.1038/s41586-021-04282-9.
12. Hughes J, Macdonald DW. A review of the interactions between free-roaming domestic dogs and wildlife. Biological conservation. 2013;157:341-51. doi: https://doi.org/10.1016/j.biocon.2012.07.005.
13. Lord K, Feinstein M, Smith B, Coppinger R. Variation in reproductive traits of members of the genus Canis with special attention to the domestic dog (Canis familiaris). Behavioural processes. 2013;92:131-42. doi: https://doi.org/10.1016/j.beproc.2012.10.009.
14. Pilot M, Malewski T, Moura AE, Grzybowski T, Oleński K, Ruść A, et al. On the origin of mongrels: evolutionary history of free-breeding dogs in Eurasia. Proceedings of the Royal Society B: Biological Sciences. 2015;282(1820):20152189. doi: https://doi.org/10.1098/rspb.2015.2189.
15. Bonanni R, Cafazzo S. The social organisation of a population of free-ranging dogs in a suburban area of Rome: a reassessment of the effects of domestication on dogs' behaviour. The social dog: Elsevier; 2014. p. 65-104.
16. Cafazzo S, Valsecchi P, Bonanni R, Natoli E. Dominance in relation to age, sex, and competitive contexts in a group of free-ranging domestic dogs. Behavioral Ecology. 2010;21(3):443-55. doi: https://doi.org/10.1093/beheco/arq001.
17. Range F, Marshall-Pescini S. Wolves and Dogs: Between Myth and Science: Springer Nature; 2022.
18. Hampson K, Coudeville L, Lembo T, Sambo M, Kieffer A, Attlan M, et al. Estimating the global burden of endemic canine rabies. PLoS neglected tropical diseases. 2015;9(4):e0003709. doi: https://doi.org/10.1371/journal.pntd.0003709.
19. Lazzaroni M, Range F, Bernasconi L, Darc L, Holtsch M, Massimei R, et al. The role of life experience in affecting persistence: A comparative study between free-ranging dogs, pet dogs and captive pack dogs. PLOS ONE. 2019;14(4):e0214806. doi: https://doi.org/10.1371/journal.pone.0214806.
20. Lazzaroni M, Marshall-Pescini S. Der Streuner- das fehlende Bindeglied. In: Kitchenham K, editor. Streuner Hunde: Von Moskaus U-Bahn-Hunden bis Indiens Underdogs: Komsos; 2020.
21. Bhattacharjee D, Mandal S, Shit P, Varghese MG, Vishnoi A, Bhadra A. Free-ranging dogs are capable of utilizing complex human pointing cues. Frontiers in Psychology. 2020;10:2818. doi: https://doi.org/10.3389/fpsyg.2019.02818.

22. Horn L, Cimarelli G, Boucherie PH, Šlipogor V, Bugnyar T. Beyond the dichotomy between field and lab—the importance of studying cognition in context. Current Opinion in Behavioral Sciences. 2022;46:101172. doi: https://doi.org/10.1016/j.cobeha.2022.101172.
23. Paul MM, Sreejani Sen; Sau, Shubhra; Nandi, Anjan K.; Bhadra, Anindita. High early life mortality in free-ranging dogs is largely influenced by humans. Sci Rep. 2016;6(19641). doi: https://doi.org/10.1038/srep19641.
24. Boyko AR, Boyko RH, Boyko CM, Parker HG, Castelhano M, Corey L, et al. Complex population structure in African village dogs and its implications for inferring dog domestication history. Proceedings of the National Academy of Sciences. 2009;106(33):13903-8. doi: https://doi.org/10.1073/pnas.0902129106.
25. Hansen Wheat C, Fitzpatrick JL, Rogell B, Temrin H. Behavioural correlations of the domestication syndrome are decoupled in modern dog breeds. Nature communications. 2019;10(1):1-9. doi: https://doi.org/10.1038/s41467-019-10426-3.
26. Hansen Wheat C, van der Bijl W, Wheat CW. Morphology does not covary with predicted behavioral correlations of the domestication syndrome in dogs. Evolution letters. 2020;4(3):189-99. doi: https://doi.org/10.1002/evl3.168.
27. Vanak AT, Gompper ME. Dogs Canis familiaris as carnivores: their role and function in intraguild competition. Mammal review. 2009;39(4):265-83. doi: https://doi.org/10.1111/j.1365-2907.2009.00148.x.
28. Larson G, Fuller DQ. The evolution of animal domestication. Annual Review of Ecology, Evolution, and Systematics. 2014;45:115-36. doi: https://doi.org/10.1146/annurev-ecolsys-110512-135813.
29. Sánchez-Villagra MR, Geiger M, Schneider RA. The taming of the neural crest: a developmental perspective on the origins of morphological covariation in domesticated mammals. Royal Society open science. 2016;3(6):160107. doi: https://doi.org/10.1098/rsos.160107.
30. Range F, Marshall-Pescini S. Comparing wolves and dogs: current status and implications for human 'self-domestication'. Trends in Cognitive Sciences. 2022. doi: https://doi.org/10.1016/j.tics.2022.01.003.
31. Kato M, Yamamoto H, Inukai Y, Kira S. Survey of the stray dog population and the health education program on the prevention of dog bites and dog-acquired infections: a comparative study in Nepal and Okayama Prefecture, Japan. Acta Medica Okayama. 2003;57(5):261-6. doi: https://doi.org/10.18926/AMO/32829.
32. Bhattacharjee D, Sau S, Das J, Bhadra A. Free-ranging dogs prefer petting over food in repeated interactions with unfamiliar humans. Journal of Experimental Biology. 2017;220(24):4654-60. doi: https://doi.org/10.1242/jeb.166371.
33. Brubaker L, Dasgupta S, Bhattacharjee D, Bhadra A, Udell MA. Differences in problem-solving between canid populations: Do domestication and lifetime experience affect persistence? Animal Cognition. 2017;20(4):717-23. doi: https://doi.org/10.1007/s10071-017-1093-7.
34. Ortolani A, Vernooij H, Coppinger R. Ethiopian village dogs: Behavioural responses to a stranger's approach. Applied Animal Behaviour Science. 2009;119(3-4):210-8. doi: https://doi.org/10.1016/j.applanim.2009.03.011.
35. Fratkin JL, Sinn DL, Patall EA, Gosling SD. Personality consistency in dogs: a meta-analysis. PloS one. 2013;8(1):e54907. doi: https://doi.org/10.1371/journal.pone.0054907.
36. Valsecchi P, Barnard S, Stefanini C, Normando S. Temperament test for re-homed dogs validated through direct behavioral observation in shelter and home environment. Journal of Veterinary Behavior. 2011;6(3):161-77. doi: https://doi.org/10.1016/j.jveb.2011.01.002.
37. Barnard S, Kennedy D, Watson R, Valsecchi P, Arnott G. Revisiting a previously validated temperament test in shelter dogs, including an examination of the use of fake model dogs to assess conspecific sociability. Animals. 2019;9(10):835. doi: https://doi.org/10.3390/ani9100835.

38. Heberlein MT, Turner DC, Range F, Virányi Z. A comparison between wolves, Canis lupus, and dogs, Canis familiaris, in showing behaviour towards humans. Animal Behaviour. 2016;122:59-66. doi: https://doi.org/10.1016/j.anbehav.2016.09.023.

39. Mendes JWW, Resende B, Savalli C. A review of the unsolvable task in dog communication and cognition: comparing different methodologies. Animal Cognition. 2021;24(5):907-22. doi: https://doi.org/10.1007/s10071-021-01501-8.

40. Svartberg K. The hierarchical structure of dog personality in a new behavioural assessment: A validation approach. Applied Animal Behaviour Science. 2021;238:105302. doi: https://doi.org/10.1016/j.applanim.2021.105302.

41. Turcsán B, Wallis L, Virányi Z, Range F, Müller CA, Huber L, et al. Personality traits in companion dogs—Results from the VIDOPET. PLoS One. 2018;13(4):e0195448. doi: https://doi.org/10.1371/journal.pone.0195448.

42. Barnard S, Marshall-Pescini S, Passalacqua C, Beghelli V, Capra A, Normando S, et al. Does Subjective Rating Reflect Behavioural Coding? Personality in 2 Month-Old Dog Puppies: An Open-Field Test and Adjective-Based Questionnaire. PLOS ONE. 2016;11(3):e0149831. doi: https://doi.org/10.1371/journal.pone.0149831.

43. Brady K, Hewison L, Wright H, Zulch H, Cracknell N, Mills D. A spatial discounting test to assess impulsivity in dogs. Applied Animal Behaviour Science. 2018;202:77-84. doi: https://doi.org/10.1016/j.applanim.2018.01.003.

44. Plutchik R. Individual and breed differences in approach and withdrawal in dogs. Behaviour. 1971;40(3-4):302-11.

45. Wright HF, Mills DS, Pollux PM. Behavioural and physiological correlates of impulsivity in the domestic dog (Canis familiaris). Physiology & behavior. 2012;105(3):676-82. doi: https://doi.org/10.1016/j.physbeh.2011.09.019.

46. Svartberg K, Forkman B. Personality traits in the domestic dog (Canis familiaris). Applied animal behaviour science. 2002;79(2):133-55. doi: https://doi.org/10.1016/S0168-1591(02)00121-1.

47. Reid PJ, Cussen VA, Collins LK, Lockwood R. The Utility of Model Dogs for Assessing Conspecific Aggression in Fighting Dogs. Applied Animal Behaviour Science. 2022:105710. doi: https://doi.org/10.1016/j.applanim.2022.105710.

48. Kaiser S, Hennessy MB, Sachser N. Domestication affects the structure, development and stability of biobehavioural profiles. Frontiers in Zoology. 2015;12(1):1-11. doi: https://doi.org/10.1186/1742-9994-12-S1-S19.

49. Svartberg K, Tapper I, Temrin H, Radesäter T, Thorman S. Consistency of personality traits in dogs. Animal Behaviour. 2005;69(2):283-91. doi: https://doi.org/10.1016/j.anbehav.2004.04.011.

50. Krause MA, Udell MA, Leavens DA, Skopos L. Animal pointing: Changing trends and findings from 30 years of research. Journal of Comparative Psychology. 2018;132(3):326. doi: https://doi.org/10.1037/com0000125.

51. Marshall-Pescini S, Rao A, Virányi Z, Range F. The role of domestication and experience in 'looking back'towards humans in an unsolvable task. Scientific reports. 2017;7(1):46636. doi: https://doi.org/10.1038/srep46636.

52. Lazzaroni M, Marshall-Pescini S, Manzenreiter H, Gosch S, Přibilová L, Darc L, et al. Why do dogs look back at the human in an impossible task? Looking back behaviour may be over-interpreted. Animal Cognition. 2020;23(3):427-41.

53. Miklósi Á, Kubinyi E, Topál J, Gácsi M, Virányi Z, Csányi V. A simple reason for a big difference: wolves do not look back at humans, but dogs do. Current biology. 2003;13(9):763-6. doi: https://doi.org/10.1016/S0960-9822(03)00263-X.

54. D'Aniello B, Scandurra A. Ontogenetic effects on gazing behaviour: a case study of kennel dogs (Labrador Retrievers) in the impossible task paradigm. Animal cognition. 2016;19:565-70. doi: https://doi.org/10.1007/s10071-016-0958-5.

55. Bonanni R, Cafazzo S, Valsecchi P, Natoli E. Effect of affiliative and agonistic relationships on leadership behaviour in free-ranging dogs. Animal Behaviour. 2010;79(5):981-91. doi: https://doi.org/10.1016/j.anbehav.2010.02.021.
56. Pompey KT. A Unified Approach to Estimating the Intraclass Correlation Coefficient and Its Bias: an Exploratory Study: University of South Carolina; 2021.
57. Boström AF, Hyytiäinen HK, Koho P, Cizinauskas S, Hielm-Björkman AK. Development of the Finnish neurological function testing battery for dogs and its intra-and inter-rater reliability. Acta Veterinaria Scandinavica. 2018;60(1):1-9. doi: https://doi.org/10.1186/s13028-018-0408-2.
58. Team RDC. R: A language and environment for statistical computing. 2010.
59. Shrout PE, Fleiss JL. Intraclass correlations: uses in assessing rater reliability. Psychological bulletin. 1979;86(2):420. doi: https://doi.org/10.1037/0033-2909.86.2.420.
60. Harvey ND. A Simple Guide to Inter-rater, Intra-rater and Test-retest Reliability for Animal Behaviour Studies. 2021. doi: https://doi.org/10.31219/osf.io/8stpy.
61. Harvey ND, Craigon PJ, Sommerville R, McMillan C, Green M, England GC, et al. Test-retest reliability and predictive validity of a juvenile guide dog behavior test. Journal of Veterinary Behavior. 2016;11:65-76. doi: https://doi.org/10.1016/j.jveb.2015.09.005.
62. Koo TK, Li MY. A guideline of selecting and reporting intraclass correlation coefficients for reliability research. Journal of chiropractic medicine. 2016;15(2):155-63. doi: https://doi.org/10.1016/j.jcm.2016.02.012.
63. Patronek GJ, Bradley J, Arps E. What is the evidence for reliability and validity of behavior evaluations for shelter dogs? A prequel to "No better than flipping a coin". Journal of Veterinary Behavior. 2019;31:43-58. doi: https://doi.org/10.1016/j.jveb.2019.03.001.
64. Sinn DL, Gosling SD, Hilliard S. Personality and performance in military working dogs: Reliability and predictive validity of behavioral tests. Applied Animal Behaviour Science. 2010;127(1-2):51-65. doi: https://doi.org/10.1016/j.applanim.2010.08.007.
65. van der Borg JA, Beerda B, Ooms M, de Souza AS, van Hagen M, Kemp B. Evaluation of behaviour testing for human directed aggression in dogs. Applied Animal Behaviour Science. 2010;128(1-4):78-90. doi: https://doi.org/10.1016/j.applanim.2010.09.016.
66. Fleiss JL, Cohen J. The equivalence of weighted kappa and the intraclass correlation coefficient as measures of reliability. Educational and psychological measurement. 1973;33(3):613-9. doi: https://doi.org/10.1177/001316447303300309.
67. Moretti L, Hentrup M, Kotrschal K, Range F. The influence of relationships on neophobia and exploration in wolves and dogs. Animal Behaviour. 2015;107:159-73. doi: https://doi.org/10.1016/j.anbehav.2015.06.008.
68. Klausz B, Kis A, Persa E, Miklósi Á, Gácsi M. A quick assessment tool for human-directed aggression in pet dogs. Aggressive behavior. 2014;40(2):178-88. doi: https://doi.org/10.1002/ab.21501.
69. Beerda B, Schilder MB, van Hooff JA, de Vries HW. Manifestations of chronic and acute stress in dogs. Applied Animal Behaviour Science. 1997;52(3-4):307-19. doi: https://doi.org/10.1016/S0168-1591(96)01131-8.
70. Pedretti G, Canori C, Biffi E, Marshall-Pescini S, Valsecchi P. Appeasement function of displacement behaviours? Dogs' behavioural displays exhibited towards threatening and neutral humans. Animal Cognition. 2023:1-10. doi: https://doi.org/10.1007/s10071-023-01742-9.
71. Lazzaroni M, Range F, Backes J, Portele K, Scheck K, Marshall-Pescini S. The Effect of Domestication and Experience on the Social Interaction of Dogs and Wolves With a Human Companion. Frontiers in Psychology. 2020;11. doi: https://doi.org/10.3389/fpsyg.2020.00785.
72. Cafazzo S, Marshall-Pescini S, Lazzaroni M, Virányi Z, Range F. The effect of domestication on post-conflict management: Wolves reconcile while dogs avoid each other. Royal Society Open Science. 2018;5(7):171553. doi: https://doi.org/10.1098/rsos.171553.

73. Cimarelli G, Marshall-Pescini S, Range F, Berghänel A, Virányi Z. Relationship quality affects social stress buffering in dogs and wolves. Animal Behaviour. 2021;178:127-40. doi: https://doi.org/10.1016/j.anbehav.2021.06.008.
74. Virányi Z, Gácsi M, Kubinyi E, Topál J, Belényi B, Ujfalussy D, et al. Comprehension of human pointing gestures in young human-reared wolves (Canis lupus) and dogs (Canis familiaris). Animal cognition. 2008;11:373-87. doi: https://doi.org/10.1007/s10071-007-0127-y.
75. Nawroth C, Wiesmann K, Schlup P, Keil N, Langbein J. Domestication and breeding objective did not shape the interpretation of physical and social cues in goats (Capra hircus). Scientific Reports. 2023;13(1):19098.
76. Gácsi M, Kara E, Belényi B, Topál J, Miklósi Á. The effect of development and individual differences in pointing comprehension of dogs. Animal cognition. 2009;12(3):471-9. doi: https://doi.org/10.1007/s10071-008-0208-6.
77. Morrill K, Hekman J, Li X, McClure J, Logan B, Goodman L, et al. Ancestry-inclusive dog genomics challenges popular breed stereotypes. Science. 2022;17(6592):e0274383. doi: https://doi.org/10.1126/science.abk0639.
78. Bray EE, Gnanadesikan GE, Horschler DJ, Levy KM, Kennedy BS, Famula TR, et al. Early-emerging and highly heritable sensitivity to human communication in dogs. Current Biology. 2021;31(14):3132-6. e5. doi: https://doi.org/10.1016/j.cub.2021.04.055.
79. Gray MM, Granka JM, Bustamante CD, Sutter NB, Boyko AR, Zhu L, et al. Linkage disequilibrium and demographic history of wild and domestic canids. Genetics. 2009;181(4):1493-505. doi: https://doi.org/10.1534/genetics.108.098830.
80. Svartberg K. Breed-typical behaviour in dogs—Historical remnants or recent constructs? Applied Animal Behaviour Science. 2006;96(3-4):293-313.
81. Sutter NB, Ostrander EA. Dog star rising: the canine genetic system. Nature Reviews Genetics. 2004;5(12):900-10. doi: https://doi.org/10.1038/nrg1492.
82. Dingemanse NJ, Barber I, Wright J, Brommer J. Quantitative genetics of behavioural reaction norms: genetic correlations between personality and behavioural plasticity vary across stickleback populations. Journal of evolutionary biology. 2012;25(3):485-96. doi: https://doi.org/10.1111/j.1420-9101.2011.02439.x.
83. Araya-Ajoy YG, Dingemanse NJ. Repeatability, heritability, and age-dependence of seasonal plasticity in aggressiveness in a wild passerine bird. Journal of Animal Ecology. 2017;86(2):227-38. doi: https://doi.org/10.1111/1365-2656.12621.
84. Bradshaw JW, Pullen AJ, Rooney NJ. Why do adult dogs 'play'? Behavioural processes. 2015;110:82-7. doi: https://doi.org/10.1016/j.beproc.2014.09.023.
85. Kolm N, Temrin H, Miklósi Á, Kubinyi E, Garamszegi LZ. The link between selection for function and human-directed play behaviour in dogs. Biology letters. 2020;16(9):20200366. doi: https://doi.org/10.1098/rsbl.2020.0366.
86. Hansen Wheat C, Fitzpatrick J, Tapper I, Temrin H. Wolf (Canis lupus) hybrids highlight the importance of human-directed play behavior during domestication of dogs (Canis familiaris). Journal of Comparative Psychology. 2018;132(4):373. doi: https://doi.org/10.1037/com0000119.
87. Brownell D. The Brownell-Marsolais scale: a proposal for the qualitative evaluation of SAR/disaster K9 candidates. Adv Rescue Technol. 2002;5:57-67.

Supplementary Information

S1 Exemplary video showing the different subtests of the test battery

S2 Equipment information

S3 Table. Detailed Ethogram

S4 Table. Detailed inter- and intra-rater reliability results

S5 Table. Detailed test-retest reliability outcome

Journal Requirements:

Please review your reference list to ensure that it is complete and correct. If you have cited papers that have been retracted, please include the rationale for doing so in the manuscript text, or remove these references and replace them with relevant current references. Any changes to the reference list should be mentioned in the rebuttal letter that accompanies your revised manuscript. If you need to cite a retracted article, indicate the article's retracted status in the References list and also include a citation and full reference for the retraction notice.

- Thank you for pointing this out. We carefully checked the references of the current manuscript (after the revision with the comments below) and could not find any retracted sources. There might, perhaps, have been one in the previous versions of this ms, but if that was the case, they have been deleted in the editorial process.

Reviewer #1:

Reviewer #1: Thank you for giving me the opportunity to review the revised version of this manuscript. I really appreciate the great effort the authors have put into changing the narrative to a methods paper. While I think the manuscript is much improved, I, however, still have some comments that I feel could still substantially improve the submission.
My main issue with the manuscript in its current form is that the great clarity and conciseness of the abstract is somewhat lost in the main text, where especially the introduction, and to some extend the discussion, get overly wordy and complicated. This is a shame, as the message in the abstract is so to the point and all the extra fill added in the manuscript text takes away from this, leaving the reader with a quite confused impression in which the developed methods end up coming across much vaguer than they actually are. I therefore mainly have general comments regarding this issue and suggestions on how to deal with them:

General answer to Reviewer #1:
We would like to take this opportunity to thank you for your valuable thoughts and explaining your point of view in detail. We agree with the majority of your points and have taken them on board (detailed below). Therein, particularly the first half of the introduction has been slimmed down and the domestication subject has been reduced, though we do argue for its merit and hence retained a more nuanced picture of it. We hope that this version makes for an easier and more satisfying read and does the topic justice.

INTRODUCTION
The narrative about changes during domestication still seems to be forced into the manuscript and it is distracting from what it is really about: a methods paper testing whether it is feasible to use a behavioral test battery on free-ranging dogs (like it is used on pet dogs).

- Thank you for seeing the merit in our paper and your detailed suggestions. We do agree that the domestication subject can be dropped in some areas (detailed below). However, we believe that disregarding the value free-ranging dogs could have for domestication research would not do the topic justice. And since reviewer 2 was delighted about the inclusion of this topic ("I am certain that the study adds great value to domestication studies focused on behavioral differences across populations"), we have chosen to retain it as a point of focus. Nevertheless, your arguments were convincing in that our iteration of the topic was not differentiated enough and took up too much space. Hence, we have cut it down in line with your suggestions and only retain it as one of the arguments for studying free-ranging populations, including the needed differentiation of breed-admixture in free-roaming populations.

For instance, in the very first line in the introduction (line 45-47) it is mentioned which behavioral traits are expected to change during dog domestication but this is irrelevant to the narrative of the

sentence and the rest of the paragraph. Why not just focus on the history of dog domestication and the interest of dogs in science?

- L45: the part about specific hypotheses has been removed to improve clarity. To underline for the lay person why behaviour might have been of interest to humans since early on, we did change the second part of the sentence to simply point to human-directed selection, though excluding the points to the specific hypotheses.

This line of argumentation leads me to the paragraph spanning line 76-92. I have already stated in my previous review that I largely agree that pet/breed dogs are not the best lineages of dogs for questions relating to domestication. This point has been emphasized by several large-scale genetic and behavioral studies. However, I do have to object to the statement that free-ranging dogs per se are better candidates for studying dog domestication. I suspect that the authors might be conflating the generic term free-ranging dogs with village dogs, which are independent canid lineages that predates breed dog formation. While true that the majority of the worlds' dog population is indeed free-ranging, all free-ranging dogs are not village dogs. A significant proportion of free-ranging dogs are abandoned pet dogs and should not automatically be labeled as an independent genetic lineage without some sort of genetic evidence hereof. I also feel that the Boyko references is particularly misguided in its use in line 86 as they show that while authentic village dog lineages do exist in Africa, many dogs are heavily admixed with breed dog lineages.

- L76-92: Thank you for pointing out this important differentiation! We have changed the paragraph to point to the possible admixture more rigorously (now L73-78).

This is not to say that I disagree with the argumentation that 1) free-ranging dog populations are worth studying (they are and we should), and 2) that some of them are better representatives than breed dogs. I just think that especially point 2) should be approached with caution and maybe in this context, where the authors do not in fact have any evidence for the genetic makeup of their free-ranging dog population, should err on the side of the narrative of point 1) and simply argue that because the vast majority of the worlds dog population is free-ranging AND thus free of strong artificial selection, we should simply make the effort to develop validated methods to quantify the behaviors of these dogs. That alone is a strong and clear argument – just like in the abstract. The other narrative (i.e. point 2)) is not important to the methods presented and just adds clutter and confusion.

- We understand your point, and it has merit! We concede that we dwelled on the domestication part too much in the face of what this paper is actually about, particularly in this indicated part. Nevertheless, we do not want to overlook the value free-ranging populations can have for this topic altogether, and we see great importance in advocating for it. And since the paragraph is about the value of testing free-ranging dogs in general rather than our specific population, we argue that our current lack of knowledge about the latter's genetics does not invalidate that point. This paper is but a starting point rather than an insight into this population in particular. Hence, we retained part of our argumentation here but shortened it and phrased it more adequately to honour the very valid point you made above about admixture interferences (see point above, L73-78).

Line 93-120 is what it is all about – rigor and repeatability, love to see it!

- Now L82-111. We are very grateful to hear that! This part has remained largely unchanged besides your comments below and the writing-related comments made by reviewer 2.

Specific comments:

Line 112: Interchanging use of free-ranging dogs and street dogs throughout the manuscript, which is a bit confusing.

- We chose to alternate between street and free-ranging dogs on purpose to differentiate between our population and free-ranging dogs overall. While we argue for the importance of considering and testing free-ranging dogs, our sample focussed on street dogs, as defined in L56 and declared in L101. Hence, we mostly use free-ranging in the intro and then switch to

street dogs in the methods and results. In the discussion, we alternate depending on whether it pertains to free-ranging populations overall or whether we can only infer to (these) street dogs in particular. While we understand that this might be a bit confusing, we do not want to make overgeneralized statements that we do not have any evidence for, and we trust the readers to understand the difference after rereading the definition in case confusion does occur. Nevertheless, to further aid this understanding, we added the definition again when introducing our methods at the end of the introduction (L101).

Line 114: Which domestication hypotheses. Why is this relevant to this methods paper?

- These were initially mentioned in the first line of the paper but we have removed them from there now. Hence, we added them in brackets here (now L103).

Line 186: Seems like the authors are testing multiple behaviors at the same time here. Playfulness could be independent of sociability, no? Will sociability and play be separated in the analyses? Ah, I can see that playfulness was dropped from the analyses…

- Indeed, we were testing sociability behaviours as well as play behaviour in this subtest as indicated in L165, divided by the different phases (Human approach – play). The two behaviour categories would have been analysed separately (as suggested by the different categories in the ethogram), but as you correctly identified, play had to be excluded due to low engagement.

Line 187: My objections to using the fake dog as a test of conspecific aggression from the first review remain unchanged given the results presented (even though the issues with the test possibly quantifying neophobia and boldness instead of conspecific aggression is now lightly touched upon in line 530-532).

- We do agree that a fake dog is not the most ideal measure to assess conspecific behaviour. Its merits in this situation (safety, time-effectiveness, flexibility, standardisation) and the fact that is has been validated repeatedly, as well as the significant drawbacks (see paragraph on the fake dog (L511-521) and in the limitations (L586-589)) are discussed in the discussion and we make suggestions for better approaches (shorter time, real life observations). Since it is the goal of this paper to present our used methods and discuss their pros and cons in depth, we will retain this part in the methods as is and trust the reader to make their own final judgement depending on their situation.

Line 211: Please also see Nawroth, C., Wiesmann, K., Schlup, P. et al. Domestication and breeding objective did not shape the interpretation of physical and social cues in goats (Capra hircus). Sci Rep 13, 19098 (2023) https://doi.org/10.1038/s41598-023-46373-9 for the latest on the pointing debate.

- Thank you for this lovely read! It was very insightful. We have added it as a reference towards the point you make right below (L472).

Line 479-483: Not necessarily. These dogs could also have learned that humans mean food, which seems highly likely given that they are all scavengers. So, the behaviors the authors here associate with consistency and heritability could simply be the result of a learned behavioral process. How can the authors disentangle these concepts when they do not know the background (genetic, social etc.) of the dogs they are testing? This part is very speculative at best.

- We agree that this part is speculative, and it was not intended otherwise. We did not mean to argue that it is hereditary in our population but rather point to other papers that have provided such evidence. Regardless, you are undoubtedly correct that we cannot disentangle whether life experience or genetics are the reason for such consistency here. We have added a few sentences in the paragraph (L470-472) to bring out that point and a possible argument that might nevertheless suggest a genetic component.

Line 502-511: This is a thoughtful paragraph. I wonder if point 3) does not leave room for habituation to the test situation, which might affect behavioral measurements?

- Now L494: That is absolutely correct. We had some further elaborations towards those points initially but deleted it to decrease the word count, hoping that the reader could infer as much themselves. But we do agree that particularly for people not familiar with such testing methods, it should be pointed out. One solution might be that the time before the

second testing should be substantially longer than 6 weeks, but seeing as we don't know when they would stop remembering the test, that is not ideal. Likewise, just starting from where the initial test was stopped might alter the behaviour in the subsequent subtests as well. Hence, we now added a half-sentence suggesting that the best way might be to repeat the test (for the sake of the missing subtests) but take the measures from the initial test for the subtests that are being repeated to exclude habituation. However, which approach is ultimately the best depends on the methods, goal, and testing constraints, and should thus be selected by the respective researchers themselves.

Line 565-566: The authors have not introduced the domestication syndrome until this point. The reader does not know that this syndrome, or neoteny, is and it is not relevant to the narrative. Strongly suggest deleting this part.

- Thank you for this observant comment. We did delete the reference to the DS and neoteny and simply referred to retained play behaviour as a frequent trait in domesticated animals (L552).

Line 571-575: This part is only relevant if the authors know for sure that their dog population predates breed formation and they currently do not have the genetic evidence to back this statement up. In this light I find this line of argumentation irrelevant and speculative in regard to the results presented.

- This part was taken out.

Reviewer #2:

I thank the Authors for addressing previous comments. The scope is much more constrained and I find it has improved the work significantly, resulting in a methods paper that domestication workers will find useful in the design of larger studies, and very informative on the challenges of behavioral studies outside of laboratory environments.

This manuscript is much improved, has better focus, and most importantly, now covers its intended scope and stays within it. The study offers a behavioral testing method for street or free-roaming dogs, and presents results on the reliability of coding and retesting in a non-laboratory environment. It is presented as the longest and most complex behavioral test for this type of setting and sample. The methods are clearly described and the protocol is well explained. The suite of tests is reported to be successful in the amount of coding reliability, and less so in the retesting consistency, but overall, is informative on the feasibility of this type of behavioral assessment and on the challenges that can be encountered. It was a very interesting read, and I learned much while doing so. I am certain that the study adds great value to domestication studies focused on behavioral differences across populations, and hopefully will serve as a starting point for larger works. Importantly, it points out certain pitfalls in standard protocols that did not work well in a street environment, all which could improve and inform future work in this area. It is very interesting that the most consistent traits were those involving human engagement/communication.

I find the Abstract needs work. The wording is awkward in several sentences, and it makes the setup of the article confusing. There were several problems with sentence structure throughout the MS. Please see the pdf where I tracked the suggested edits, as well as edits to the Abstract. Of course, these are only suggestions.

General answer to reviewer #2:

Thank you very much for this detailed effort! We have taken your suggestion seriously and changed the ms accordingly (see below for detail). Since reviewer 1 expressed his great appreciation for the abstract, we chose to keep changes in this part to a minimum – besides honouring your suggestions. We hope this has created an easier read that is satisfactory for readers from many different scopes.

A few more specific comments + In-text comments:

Abstract

- L24: lagging behind what?
  - This sentence has been changed to “has been slow” to take out the need for comparison.
- L25: THis is another confusing, awkward sentence. You need to keep the subject of the sentence clear. "no tests have been conducted on [what]..."their [subject here is the tests, not the dogs, but structure the sentence so this is clear] reliability is unknown.."
  - Thank you for pointing this out. The sentences have been restructured to provide more clarity (L24-28).
- L28; i suggest "on"
  - Accepted.
- L 40: I am not sure there is evidence of this in your paper. Is there? I agree with that your results highlight difficulties in controlling the environment, but I missed how they find evidence of "intrinsic differences between pet/street dogs.
  - We were referring particularly to the play part in the human approach test here where a standardly used play and sociability test did not work at all with the free-ranging population since they did not seem to understand this concept. This intrinsic difference might be due to both learned and potentially genetic differences, as argued in L559-562.
- L40: Reword: "bearing in mind the suggestions made in the discussion". Otherwise, sentence does not make sense. I would even start with""This test battery...." and removing the phrase before.
  - Now L41: The “bearing in mind” part has been deleted and replaced with “with some adaptations” since we did not want to advocate for this test battery in its unchanged form but do agree that the sentence structure was awkward.

Intro

- L47: should be plural
  - Accepted.
- L49: (a)if you start a phrase with "from" you need to follow up with "to"..... (b) multiple aspects of [what??] This whole phrase is vague...please specify what you are trying to say.
  - In order to shorten the intro as per reviewer 1’s request, these two sentences were merged, which in turn deleted the words you commented on here. Nevertheless, we thank you for this useful suggestion.
- L51: Again, awkward sentence structure...perhaps "there has been a surge in comparative studies..?""
  - Same as above.
- L54: I suggest either "Importantly"or "however", but not both.
  - Thank you. We chose to stick with “however”.
- L57: Wow. Very interesting.
  - Right?! Hence the need to include them more.
- L63: here you imply this is compared to something...compared to [what?]
  - Useful observation, thank you. For the sake or decreasing wordiness, we simply deleted this word.
- L84: You would need to specify what "this" is, here.
  - As above, this part has been rewritten for the sake of wordiness and better nuancing.
- L87; should be plural
  - Correct, thank you. However, this was deleted in line with reviewer 1’s comment about nuancing this paragraph.
- L93: I suggest "on"
  - Accepted.
- L95: "in which they reside". I strongly encourage never ending a sentence with a preposition.
  - A valuable suggestion, thank you. Accepted.
- L103: rater's.

- Accepted.
- L111: Grammatical suggestion: either (a) you "address" an issue or problem... (b) or you "have" objectives.
  - We have changed this to “achieve an objective”.
- L120: "to be used more.."
  - Since we do not only refer to a higher frequency but also a more widespread use, we changed this phrasing to "more widely used".

Methods

- L202: this is not the definition of neophobia, correct? I suggest correcting this, it is confusing. // Did the authors err in their definition of “neophobia”? Or is it perhaps that in the writing style it seems as if they misinterpret its meaning? Please check. (Methods section).
  - Thank you for pointing this out. It was indeed not meant as the definition but rather as a clarification for all the assessable target behaviours. The line has been written more clearly (L193).

Discussion

- L485: this sentence is unclear...you have differences between 3 things here...did you mean between 2 things?
  - This line was indeed confusing, thank you for catching that awkward phrasing. We deleted the obsolete part (L474).
- L499: do not
  - Since we referred to the begging test here, the grammar was correct. However, we see how the sentence can be confusing, so we added this part in brackets to refer to the begging test specifically rather than have it as a half sentence (L486).
- L555: 1. Line 555 (Discussion): I am not sure how one could justify making the statement that “behavioral plasticity is selected against in modern [dog] breeds. But if made, please provide a citation or source. // Please provide a citation or supportive evidence for this from a source. I am not sure how one could justify making this statement that behavioral plasticity is selected against in modern breeds.
  - We do not mean to suggest that it was consciously selected against but rather that it might have been decreased through the selection for stable behavioural traits and the loss of genetic variation associated with that. This has been rewritten accordingly to make that point clearer and references have been added for the latter two points (L540-542).
- L624: Reword to: "Bearing in mind the suggestions made..."
  - Accepted.
