# Supplementary material for: Street-wise dog testing: Feasibility and reliability of a behavioural test battery for free-ranging dogs in their natural habitat
Source: PLoS One. 2024 Mar 14;19(3):e0296509. doi: 10.1371/journal.pone.0296509 (PMC10939227; doi:10.1371/journal.pone.0296509)
Supplement: Attachment — Submitted filename: Itemized Review.docx [file pone.0296509.s007.docx]

PLOS ONE

Tackling the lack of behavioural evidence for the domestication syndrome – Feasibility

and reliability of a novel Domestication Syndrome test battery with free-ranging dogs

Abstract:

1. A brief explanation about what is meant by “human-directed interactions” vs. “disturbance-prone behaviors” vs. “less disturbance-prone” would help the reader understand your study better from the very beginning.
2. The term “free-ranging” is a bit vague and can be misinterpreted unless you define it specifically. For example, feral dogs like the Dingo have been described as free-ranging. Can your population perhaps be described further as “street dogs”? This is an important item because the type of population you are using relates directly to the testability of your hypothesis.
3. Lines 44-45: the DS is not an attempt to explain domestication, but the *traits* associated with domestication. This may seem like a trivial difference but is really important to making

your study more scholarly.

Introduction:

1. Lines 43-44: The methods/pathways used to domesticate animals are pretty well known (see Zeder 2012 for a good overview). It is the mechanisms by which *multiple morphological/phenotypic traits correlated with domestication are co-occurring* repeatedly that is misunderstood. Again, I think this is an important distinction to make throughout your paper.

Domestication is something that researchers struggle to define in a simple manner—but there is much literature on this—so defining the “type” of domestication you are testing is critical. I strongly suggest that you read Zeder 2012 for and overview of the different types of processes involved in the different types of “domestication”. Another view of what constitutes a “domestic” population is that of Lord et al. (several) – so there is much debate. This will inform whether your sampling is appropriate or not.

2. Line 56: Brain size reduction is not inconsistent among domesticated groups—quite the opposite. You can check Balcarcel et al. 2021 for a review of the literature on this subject and references therein, or Kruska’s work from the 1970s-2000’s. Your study cites Johnsson et al. 2021, which is a great paper, but I do believe there is an error in it regarding the statement of “increase” in brain size in pigs, llamas and alpacas. Johnsson et al cite Kruska (1974, 1982, 1988) and if you look at those original works, they report *decreases* in the telencephalon (not increases).
3. Lines 59-60: There is a tremendous amount of literature debating the claims of the DS, its unsuitability in describing the variation presented by different domestic populations. I would suggest discussing this a bit, to show your understanding of the subject, before getting into testing of the DS.
   a. For example, some domestication workers agree that dogs are a poor model for testing the DS hypothesis, to due to their directed artificial breeding during the last 200 yrs. (see Larson et al. 2014, McHugh & Larson 2017, Sanchez et al. 2016&2017 on lack of a universal pattern among domestics and the NCC).
   b. Dogs also don’t necessarily show snout shortening, as claimed by the DS. Please see Drake et al. 2010 on the variation in cranio-facial changes in dog breeds which highlights the shortening of the snout as an artificially-selected trait in many breeds.
4. IN GENERAL: You may want to rephrase or discuss the intricacies of the dog as a model for testing evolutionary processes. There is great potential in this model group, but not necessarily for testing the DS, in my opinion, since the DS is supposed to capture basic domestication traits common to all domestic populations…and dogs are highly artificially-selected. I think your study actually tests a *much broader* concept in domestication—the selection for tameness and reduced aggression—which is tremendously interesting.
5. Lines 69-71: Here again, I think the authors may be confounding the domestication with "domestication syndrome".
6. “Free-ranging”. I am concerned about the use of this term, and what it means for your sample. Hughes and MacDonald 2013 do not exclude feral populations under their definition of free-roaming dogs. Pilot et al. 2015 cite Hughes and MacDonald 2013, adding "not feral" to the definition, but I am not sure if there is evidence for that?
