# Supplementary material for: Street-wise dog testing: Feasibility and reliability of a behavioural test battery for free-ranging dogs in their natural habitat
Source: PLoS One. 2024 Mar 14;19(3):e0296509. doi: 10.1371/journal.pone.0296509 (PMC10939227; doi:10.1371/journal.pone.0296509)
Supplement: Attachment — Submitted filename: DSAdult22_PlosOne_Itemised Answers.docx [file pone.0296509.s008.docx]

**Additional Editor Comments:**

Dear Authors
Your manuscript has been assessed and serious flaws in the study design, DS concept and grammatical errors have been highlighted. Kindly and thoroughly address the issued raised by the reviews and resubmit for another assessment.
Thank you

We are very grateful for the reviewers’ detailed clarifications and suggestions, as well as their encouraging words and their concern with the description of the domestication-related focus. We agree with the suggestion that changing the focus of this paper to free-ranging dog testing overall provides more benefit for the scientific community and the reader. Following both reviewers’ suggestion, we have rewritten the paper by dropping the domestication syndrome constraints and refocussing on the merits this paper has.

When submitting your revision, we need you to address these additional requirements.

1. Please ensure that your manuscript meets PLOS ONE's style requirements, including those for file naming.

The requirements have been rechecked and implemented. Particularly file naming for figures and supplementary material has been corrected.

2. Please include a complete copy of PLOS’ questionnaire on inclusivity in global research in your revised manuscript.

The requested file has been uploaded.

3. We note that Figures 2 and 3 in your submission contain copyrighted images. We require you to either (1) present written permission from the copyright holder to publish these figures specifically under the CC BY 4.0 license, or (2) remove the figures from your submission.

Figure 2 has been remade to only contain MS PowerPoint pictograms which are free to use. Figure 3 has been removed and links to the specific manufacturers have been included in the supplementary information S2 in case visual information is desired.

**Reviewer 1**

Reviewer #1: GENERAL COMMENTS:
Let me start with saying that I am very happy to see a behaviour study on free-ranging dogs. However, I cannot recommend this study for publication in its current form for several reasons, the main one being that this is not a study of the domestication syndrome and I find its current presentation and narrative to be quite misleading in all honesty.
It is very unclear to me why the authors have chosen to present their manuscript so elaborate around the domestication syndrome when they a) do not confirm the presence of the domestication syndrome in the dog population (there is no test of whether the alleged domestication syndrome behaviours are in fact correlated – a central assumption of the hypothesis), and b) find that their so-called test battery fails to adequately quantify the behaviours they claim to be associated with the domestication syndrome. The eagerness to label their test battery the “Domestication Syndrome test battery” and claim that it is useful going forward in testing domestication syndrome behaviours, in spite of the very results they bring forward, therefore seem very misplaced and quite frankly puzzling. This is especially emphasized by the fact that half of this test battery includes behaviours that cannot in a meaningful way be associated with the domestication syndrome – a point the authors themselves hint at by mentioning that they will also test other domestication hypotheses with this test battery. These other behaviours, as compared to the claimed domestication syndrome behaviours, are even the only behaviours that with some confidence seem to be feasible and reliable to test in a free-ranging population. Why then label it the “Domestication Syndrome test battery”? It is simply misleading.
That being said, and while I understand that the above is a strong critique of the study, I think there is value in the study from the perspective that it is, in my opinion, actually a methods paper and it would benefit immensely from being rewritten as such. The work the authors have done in examining the feasibility and reliability in conducting in-situ behavioural testing of free-ranging dogs does have merit. So, instead of overselling this study by pinning it on a narrative that it simply cannot sustain, my strong recommendation for this manuscript is therefore that it undergoes major revision in order to be restructured to bring forward the valuable contributions it actually has in terms of the insights on general (not domestication syndrome) behavioural testing of free-ranging dogs. Highlighting the challenges of taking behavioural testing outside controlled laboratory settings and emphasize how this is a necessary next step in moving the field forward is valuable enough on its own.

GENERAL ANSWER to Reviewer #1:

Thank you for your valuable perspective and detailed concerns regarding the approach we had chosen to present this study in. The paper was indeed supposed to be a methods paper and we regret that it did not come across as such. Taking your suggestions to heart, it has been entirely rewritten, focusing on the necessity of testing free-ranging populations, how the presented test battery fits into that, and what conclusions can be drawn from our results about the possibilities and limitations of street dog testing. This pertains particularly to the introduction and partially to the discussion, where we have additionally addressed issues you raised with the methodology. The methods and results section have stayed largely the same besides clarifying specific issues you have pointed out, and renaming the Sociability test as Human approach test to clarify that all behaviours were tested. We hope that by refocussing this paper on the merits it has in terms of moving the field forward, this version is more to your satisfaction.

DETAILED COMMENTS:
Line 29: The authors need to remove the physiological reference here. Even though hair samples were collected, they were not analyzed and we have no way of knowing if a) this method is reliable in obtaining physiological parameters, and b) if these correlate with the behavioural traits in the domestication syndrome. This reference is there for misleading in this context.

With the rewriting of the abstract, this line was taken out.

Line 53: Some clarity is needed here. Selection on tameness is not the mechanism per se. Tameness is likely the behavioural trait that was selected for during initial animal domestication cascading the phenotypic changes as seen in the domestication syndrome. However, the underlying mechanism causing the presumed correlated traits of the domestication syndrome remains unknown, but has been proposed to be neural crest deficit (Wilkins et al. 2016) or attenuated ontogenesis (Balyaev 1979).

As expressed above, we want to thank you for your clarifications and suggestions particularly in terms of refocusing the paper. Since the introduction has been entirely rewritten in this effort, many of the issues you have pointed out in this comment and below have been erased from the text. Whenever this is the case, we will mark it with “erased due to focus shift”.

Line 58: What are all the morphological domestication syndrome-associated traits? Details are generally very sparse and the background is too superficial.

“erased due to focus shift”

Line 59: What physiological traits? Which traits are part of the DS and which do not fit with the hypothesis in dogs?

“erased due to focus shift”

Line 60-61: Which behavioural traits? Which traits are part of the DS and which do not fit with the hypothesis in dogs?

“erased due to focus shift”

Line 61- 66: This part has me a bit confused. It seems more like an afterthought and I am not quite sure how it fits in with the narrative. Are the authors saying that the domestication syndrome is not true in dogs or that it is expanded just in dogs with these extra features?

“erased due to focus shift”

Line 64: I am quite surprised to see the uncritical and sole use of this outdated reference (Hare and Tomasello 2005), given the long-standing debate (of +20 years) on the topic and the not so few studies refuting the uniqueness in dogs being able to follow human communicative clues. Udell et al. (2008) demonstrated that wolves outperformed dogs in a pointing test when tested under identical conditions. Likewise, Wheat et al. (2022) reanalyzed previously published data claiming that dog puppies have early emerging skills in following human pointing whereas wolves do not, but upon adequately controlling for the puppies’ different environmental exposure dogs and wolves performed equally well. Furthermore, it has been demonstrated that close to 30 species (both wild and domesticated, e.g. elephants, bats, goats, pigs and not the least wolves) across eight taxonomic groups have the ability to follow pointing gestures (see Krause et al. 2018 for review).

“erased due to focus shift”. The reference still appears in the manuscript but only as a reference to Hare’s emotional reactivity hypothesis. In addition, we want to thank you for pointing us to this useful reference. We included Krause’s observations in line 214.
Line 73-82: While I agree that it is problematic that some studies do not address the behavioural traits of the domestication syndrome in concert, I am puzzled by the argument that standardized test batteries designed to quantify behaviours, of which some are the basic behaviours of the domestication syndrome, should somehow be unfit for hypothesis testing. The argumentation brought forward by the authors here becomes quite ironic as they a) do not test all the behavioural traits of the domestication syndrome, and b) do not test the covariance between the behaviours they do test. There is thus no actual confirmation of the domestication syndrome in their free-ranging dog population, given one would accept the premise that the behaviours chosen are in fact domestication syndrome behaviours.

“erased due to focus shift”. In addition, a) is a very fair argument and a major reason why we agreed to drop the Domestication Syndrome narrative. For point b), analyses might follow in the future if the test can be established reliably on the streets, which was the main focus of this paper.

Line 83-94: I generally agree with this paragraph. However, it is too much of a blanket statement that free-ranging dogs in general is not admixed with breed dogs. For instance, Boyko et al. has a paper from 2009 showing that more than 60% of African village dogs could be classified as non-native, i.e. with a potential admixture with modern breeds. This does not mean that free-ranging dogs are not better models for these questions than modern breeds, but introgression with modern breeds must be assumed in free-breeding populations like this.

Thank you for this useful reference, it was a pleasure to read. Boyko et al. (2009) do indeed state that village dogs in some areas of Africa (esp. Namibia) show a high admixture rate while this admixture was low or non-existent in the other 84% of their sample. We have softened our statement (see line 84ff) to indicate that the “the majority of free-ranging dog populations are not a mixture of breeds but rather genetically distinct populations that predate the relatively more recent artificial selection and breed formation”, and refer to Boyko et al. (2009).

Line 100: I am starting to get a bit confused. Are the authors testing personality, the domestication syndrome or alterative domestication hypotheses?

“erased due to focus shift”

Line 103-122: My confusion grows. Now it sounds more like a methods paper…

That is indeed what it was supposed to be. We hope this version is more to your satisfaction.

Line 124-126: If the battery test all these different hypotheses (which I do not believe it does, for instance there is no test of correlation between alleged domestication syndrome traits and the majority of domestication syndrome behaviours could not be adequately quantified) how come the authors label it the “Domestication Syndrome test battery”?

“erased due to focus shift”

Line 145-146: This could create a sampling bias where only the tamest dogs participate, i.e. the most fearful dogs would not. This can ultimately affect results with exaggerated sociability scores and more fearful or less explorative dogs being underrepresented in the sample.

That is indeed an important limitation of street tests since forcing the animals to participate would likely alter their behaviour even more, and we thus have to contend with the voluntary biased sample. While the human approach test somewhat circumvents this problem by starting the data collection before the dog is aware of being approached, we have to concede that this is a problem for all the other following subtests. We have endeavoured to address this point in the discussion (line 583ff) and mention a few possible alternatives there as well.

Line 160: Which domestication syndrome traits?

“erased due to focus shift”

Line 193-203: This seems like a good test of human-directed sociability.

We are glad you agree.

Line 225-253: Subtests 4-6, sure the authors wish to test other domestication hypotheses (though see my comments on pointing above), but since these tests take up half of the test battery and also seem to have the best feasibility and reliability, why then the eagerness to label the test battery the “Domestication Syndrome test battery”. It seems to me, that this test battery unlikely to actually quantify domestication syndrome behaviours, if at all.

“erased due to focus shift”

Line 204-214: If the purpose is to quantify conspecific sociability and aggression, I am not sure I understand why the authors did not simply observe behaviours toward other dogs in the area? I am not convinced this test can be disentangled from neophobia and/or boldness.

Thank you for stating this concern. We do agree that elaborate long-term observations are the best way to assess a dog’s social behaviour, whether that is pet, working, or free-ranging dog. However, this test was conceptualized to circumvent the necessity of elaborate observations to create the possibility to test more dogs without having to learn everything about their population first. In addition, we aimed at exploring the possibility of using established pet and working dog test batteries on the streets. The fake dog test has repeatedly been validated to reliably assess dogs’ initial behaviour towards conspecifics (Barnard et al., 2019; Reid et al., 2022) and we were therefore positive that the same could be achieved on the street. With the outcome that the street dogs did indeed show appropriate conspecific behaviour (e.g., growling, barking, stand tall) upon first interaction with the fake dog that was not seen in the novel object test, as well as frequent genital sniffing, we argue that the test did achieve that goal and can be used to assess the target measures when only the initial reaction is taken into account. Nevertheless, we cannot exclude that neophobia and boldness come into play as soon as the animals realize that the opponent is not, in fact, a dog. We discuss this issue in line 528f as well as the value of observational data in line 614f.

Line 365-366: So, no aggression behaviour? Maybe one fear/stress/redirected behaviour (Yawning)?

Indeed, the aggressive behaviours and many stress behaviours were rarely shown and could thus not be retained in the test-retest analysis. We discuss the problems with that as well as possible alternatives in line 602ff**.**

Line 387: But I thought that tail between legs had poor interrater reliability and should be excluded (given that the authors adhere to the 0.07 threshold as stated in several places, e.g. line 345, 347 and 356).

We apologize for this confusion. The tail between the leg and mouthing behaviour were close to the threshold and showed marked significance (p=0.02). After long discussion and in light of the somewhat arbitrary 0.7 threshold that is heavily influenced by sample size, we chose to retain the two variables for this preliminary investigation and treat them with caution. However, we do agree that we should have stated that more clearly and understand your annoyance with the lack thereof. An explanation has been added in the methods (line 299f) and the result section (line 356ff).

Line 388: Could this low test-retest reliability be caused by habituation in spite modification of the fake dog. Maybe the testing set-up alone could be cause for habituation? Perhaps testing conspecific sociability and aggression is better in real life social contexts?

Absolutely. We share this interpretation wholeheartedly, as we have explained in line 519f. Pitfalls of short retest intervals and too similar set ups are described in the paragraph thereafter (lines 522ff), and we have added the value of long-term observations in line 614.

Line 401: Again, I am puzzled by the continued use of tail between legs even though it does not meet the authors stated 0.07 ICC threshold. I find it to be borderline misleading.

Understandably so, we apologize for this lack of explanation. Please refer to our explanation for line 387 above for our modifications.

Line 404-405: This to me indicates both habituation, as mentioned above, but also that the fake dog was indeed treated as a novel object and not a suitable model of a conspecific.

We agree with this interpretation and have discussed the limitations as mentioned above.

Table 3: I find it very misleading, and confusing, to include variables that did not pass the ICC thresholds.

Again, we apologize and agree that we should have indicated this in a better way. As mentioned above, respective measures have been taken to do so.

Line 467-475: I disagree with the interpretation of the results on sociability and the conclusions brought forward here. The sample presented here is very likely to be a result of the most social dogs in the population. This is because the dogs’ participation was entirely voluntarily and the dogs themselves could decide to leave the test at any given time. Since the study only includes test-retested dogs, this will inherently bias the sampling towards more social individuals and exclude fearful dogs.

Thank you for raising this concern. You are entirely correct that the voluntary participation creates a biased sample for the overall test-battery, and we have added a paragraph in lines 583ff to address this issue. At the same time, we would like to draw attention to three points that might alleviate your concern a little in this specific instance, i.e., the sociability (now Human approach) subtest. 1) As it was the very first subtest, the animals were not first required to come to us but rather we started the approach for this subtest before the animals were aware of it. More precisely, we appeared as normal locals to the animal right until I started the (now Human approach) test by calling their attention to me with a short vocalization. Since many animals were foraging or sleeping up to that point, they did not have a chance to flee beforehand. That means that by the time the very fearful animals started to flee, the test had already started, and their behaviour was still recorded and coded as part of the (now Human approach) test. 2) to address your concern about a bias in the retesting, we did find two-thirds of the dogs again that had already fled during the (now Human approach) subtest in the first test, creating a decent sample of fearful animals that is retained in the test-retest analysis for the sociability test. 3) And finally, the fact that only the more social animals stuck around during the tests does not negate the fact that sociability might be heritable. We do not mean to claim here that street dogs are more social than wolves (since we do not have that data and the skew would indeed cause a big issue with that) but rather that the sociability measure seems to be fairly consistent over time in an individual and could therefore serve as a trait based on which selection is possible. Rereading the sentence now I can see where it might have been interpreted differently and we have specified that we refer to consistency (line 481). To additionally counteract the perceived bias during this test, we renamed it to a more neutral “Human approach test” rather than the more directed “Sociability test”.

Line 526-532: If the authors wish to speculate about phenotypic plasticity, I encourage them to do so in a more detailed way. What would the adaptive value of this be in free-ranging dogs?

Thank you for this encouragement. We have added a few lines how behavioural plasticity might aid free-ranging dogs in adapting to their unstable and variable environment, which is not a necessity for the average breed dog anymore (lines 550f).

**Reviewer 2**

There is too little known about the variation in behavior in stray/street dogs, and this study provides a survey of the types of behavioral tests that can and cannot be performed in this difficult testing environment. The study concept is thus very interesting and worthwhile. I enjoyed reading the description of traits tested and the results and discussion. As a preliminary study, I think it would be great to see the battery of tests published so that it could be retested with a larger sample.
However, I think the title, hypotheses, and design of the paper show a fundamental misunderstanding of some of the basic concepts in domestication studies. Domestication and the Domestication Syndrome (DS) hypothesis are not equivalent concepts, and the two are confounded repeatedly throughout the paper. The DS is a well-known and highly debated hypothesis that has been tested in many ways, taken apart, and found to be highly problematic. Most domestication workers agree that is has a very specific applicability when being tested. I provided more details on relevant sources in my "Itemized Review".There is no discussion of these issues in the present manuscript. The DS aims to explain the generalized traits co-ocurring in domestic populations when compared to wild counterparts. Dogs have repeatedly been criticized as a bad model for testing "early domestication" or the DS, due to their artificial breeding. In my opinion your study does not test the DS. It tests the presence of certain human-dog interactions that reflect tameness or lack of aggression in stray dogs. It is successful in that, and would be a great survey to publish as a starting point for behavioral studies in street dogs. However, as stated in my Itemized Review, the DS is not tested here. Tameness is but one trait, and it is present in all domesticated populations that have been studied thus far. I believe the authors could redesign and re-work this study with a better understanding of what they are testing. My best wishes to you and this work. I think it would be great to see this published once it is improved.

GENERAL ANSWER TO REVIEWER #2:

We would like to thank you for your helpful clarifications regarding the domestication syndrome and detailed elaborations how this paper could be improved. We agree that the focus of this paper was not ideally represented. Taking your encouragement and the suggestion of reviewer #1 into account, we chose to entirely refocus the paper towards the possibilities and limitations of free-ranging dog testing and centre our study within that. Since we do not have a large enough sample to reliably represent the distribution of specific traits across the population (as you mention, a larger sample would be needed for that), we solely focus on the presentation and reliability validation of the presented test battery to set a starting point for more elaborate testing. In addition, we discuss important considerations our results illustrate and alternatives that might be viable. These changes can be found especially in the entirely rewritten introduction and partially rewritten discussion, while the methods and result section stayed largely the same. Finally, we defined and used the terms free-ranging and street dogs more carefully and took your suggested references on board wherever possible, thank you for those pointers. We hope that by refocussing this paper on the merits it has in terms of moving the field forward, this version is more to your satisfaction.

Abstract:

1. A brief explanation about what is meant by “human-directed interactions” vs. “disturbance-prone behaviors” vs. “less disturbance-prone” would help the reader understand your study better from the very beginning.

Thank you for pointing this out. We have entirely rewritten the abstract and therein explained the disturbance issue with more detail.

2. The term “free-ranging” is a bit vague and can be misinterpreted unless you define it specifically. For example, feral dogs like the Dingo have been described as free-ranging. Can your population perhaps be described further as “street dogs”? This is an important item because the type of population you are using relates directly to the testability of your hypothesis.

This explanation was very helpful, thank you for that. We have added a definition for free-ranging dogs in the manuscript (line 57f), and while we continue to refer to free-ranging dogs wherever appropriate, we specifically state our use of street dogs for the test battery and adapted our interpretations and discussions to refer to the population our results can or cannot be generalized to.

3. Lines 44-45: the DS is not an attempt to explain domestication, but the *traits* associated with domestication. This may seem like a trivial difference but is really important to making

your study more scholarly.

That is indeed an important differentiation. While we have erased the words pertaining to this from the ms, we will endeavour to be more accurate in our future work.

Introduction:

1. Lines 43-44: The methods/pathways used to domesticate animals are pretty well known (see Zeder 2012 for a good overview). It is the mechanisms by which *multiple morphological/phenotypic traits correlated with domestication are co-occurring* repeatedly that is misunderstood. Again, I think this is an important distinction to make throughout your paper.

Domestication is something that researchers struggle to define in a simple manner—but there is much literature on this—so defining the “type” of domestication you are testing is critical. I strongly suggest that you read Zeder 2012 for and overview of the different types of processes involved in the different types of “domestication”. Another view of what constitutes a “domestic” population is that of Lord et al. (several) – so there is much debate. This will inform whether your sampling is appropriate or not.

Thank you for the reference suggestions. While we have erased the words pertaining to this from the ms, this is certainly a note we will keep in mind.

2. Line 56: Brain size reduction is not inconsistent among domesticated groups—quite the opposite. You can check Balcarcel et al. 2021 for a review of the literature on this subject and references therein, or Kruska’s work from the 1970s-2000’s. Your study cites Johnsson et al. 2021, which is a great paper, but I do believe there is an error in it regarding the statement of “increase” in brain size in pigs, llamas and alpacas. Johnsson et al cite Kruska (1974, 1982, 1988) and if you look at those original works, they report *decreases* in the telencephalon (not increases).

That is an interesting thought and fact I was not aware of, thank you. Again something to keep in mind, though the line itself does not apply to the paper anymore.

3. Lines 59-60: There is a tremendous amount of literature debating the claims of the DS, its unsuitability in describing the variation presented by different domestic populations. I would suggest discussing this a bit, to show your understanding of the subject, before getting into testing of the DS.
   a. For example, some domestication workers agree that dogs are a poor model for testing the DS hypothesis, to due to their directed artificial breeding during the last 200 yrs. (see Larson et al. 2014, McHugh & Larson 2017, Sanchez et al. 2016&2017 on lack of a universal pattern among domestics and the NCC).
   b. Dogs also don’t necessarily show snout shortening, as claimed by the DS. Please see Drake et al. 2010 on the variation in cranio-facial changes in dog breeds which highlights the shortening of the snout as an artificially-selected trait in many breeds.

Thank you for this suggestion and we agree wholeheartedly, hence our suggestion that free-ranging dogs might be a better population. While most of this is not part of the ms anymore, we have incorporated some of it in line 80ff. Your references were very helpful in that.

4. IN GENERAL: You may want to rephrase or discuss the intricacies of the dog as a model for testing evolutionary processes. There is great potential in this model group, but not necessarily for testing the DS, in my opinion, since the DS is supposed to capture basic domestication traits common to all domestic populations…and dogs are highly artificially-selected. I think your study actually tests a *much broader* concept in domestication—the selection for tameness and reduced aggression—which is tremendously interesting.

That is a valuable suggestion and with the rewriting of this ms we have attempted to shift the focus away from the ms and incorporated more broader domestication ideas particularly in the discussion (e.g., lines 481ff)

5. Lines 69-71: Here again, I think the authors may be confounding the domestication with "domestication syndrome".

This line has been erased from the ms

6. “Free-ranging”. I am concerned about the use of this term, and what it means for your sample. Hughes and MacDonald 2013 do not exclude feral populations under their definition of free-roaming dogs. Pilot et al. 2015 cite Hughes and MacDonald 2013, adding "not feral" to the definition, but I am not sure if there is evidence for that?

Please see point 2 under abstract for our answer and implemented changes in regards to that.
