# Supplementary material for: Street-wise dog testing: Feasibility and reliability of a behavioural test battery for free-ranging dogs in their natural habitat
Source: PLoS One. 2024 Mar 14;19(3):e0296509. doi: 10.1371/journal.pone.0296509 (PMC10939227; doi:10.1371/journal.pone.0296509)
Supplement: Attachment — Submitted filename: Review 2_Reviewer2 Nov 22023.docx [file pone.0296509.s010.docx]

This manuscript is much improved, has better focus, and most importantly, now covers its intended scope and stays within it. The study offers a behavioral testing method for street or free-roaming dogs, and presents results on the reliability of coding and retesting in a non-laboratory environment. It is presented as the longest and most complex behavioral test for this type of setting and sample. The methods are clearly described and the protocol is well explained. The suite of tests is reported to be successful in the amount of coding reliability, and less so in the retesting consistency, but overall, is informative on the feasibility of this type of behavioral assessment and on the challenges that can be encountered. It was a very interesting read, and I learned much while doing so. I am certain that the study adds great value to domestication studies focused on behavioral differences across populations, and hopefully will serve as a starting point for larger works. Importantly, it points out certain pitfalls in standard protocols that did not work well in a street environment, all which could improve and inform future work in this area. It is very interesting that the most consistent traits were those involving human engagement/communication.

I find the Abstract needs work. The wording is awkward in several sentences, and it makes the setup of the article confusing. There were several problems with sentence structure throughout the MS. Please see the pdf where I tracked the suggested edits, as well as edits to the Abstract. Of course, these are only suggestions.

A few more specific comments:

1. Line 555 (Discussion): I am not sure how one could justify making the statement that “behavioral plasticity is selected against in modern [dog] breeds. But if made, please provide a citation or source.

2. Did the authors err in their definition of “neophobia”? Or is it perhaps that in the writing style it seems as if they misinterpret its meaning? Please check. (Methods section).
