# Supplementary material for: Street-wise dog testing: Feasibility and reliability of a behavioural test battery for free-ranging dogs in their natural habitat
Source: PLoS One. 2024 Mar 14;19(3):e0296509. doi: 10.1371/journal.pone.0296509 (PMC10939227; doi:10.1371/journal.pone.0296509)
Supplement: Attachment — Submitted filename: DSAdult22_SecondRevision_ItemizedAnswers.docx [file pone.0296509.s011.docx]

**Journal Requirements:**

Please review your reference list to ensure that it is complete and correct. If you have cited papers that have been retracted, please include the rationale for doing so in the manuscript text, or remove these references and replace them with relevant current references. Any changes to the reference list should be mentioned in the rebuttal letter that accompanies your revised manuscript. If you need to cite a retracted article, indicate the article’s retracted status in the References list and also include a citation and full reference for the retraction notice.

- Thank you for pointing this out. We carefully checked the references of the current manuscript (after the revision with the comments below) and could not find any retracted sources. There might, perhaps, have been one in the previous versions of this ms, but if that was the case, they have been deleted in the editorial process.

**Reviewer #1:**

Reviewer #1: Thank you for giving me the opportunity to review the revised version of this manuscript. I really appreciate the great effort the authors have put into changing the narrative to a methods paper. While I think the manuscript is much improved, I, however, still have some comments that I feel could still substantially improve the submission.
My main issue with the manuscript in its current form is that the great clarity and conciseness of the abstract is somewhat lost in the main text, where especially the introduction, and to some extend the discussion, get overly wordy and complicated. This is a shame, as the message in the abstract is so to the point and all the extra fill added in the manuscript text takes away from this, leaving the reader with a quite confused impression in which the developed methods end up coming across much vaguer than they actually are. I therefore mainly have general comments regarding this issue and suggestions on how to deal with them:

General answer to Reviewer #1:

We would like to take this opportunity to thank you for your valuable thoughts and explaining your point of view in detail. We agree with the majority of your points and have taken them on board (detailed below). Therein, particularly the first half of the introduction has been slimmed down and the domestication subject has been reduced, though we do argue for its merit and hence retained a more nuanced picture of it. We hope that this version makes for an easier and more satisfying read and does the topic justice.

INTRODUCTION
The narrative about changes during domestication still seems to be forced into the manuscript and it is distracting from what it is really about: a methods paper testing whether it is feasible to use a behavioral test battery on free-ranging dogs (like it is used on pet dogs).

- Thank you for seeing the merit in our paper and your detailed suggestions. We do agree that the domestication subject can be dropped in some areas (detailed below). However, we believe that disregarding the value free-ranging dogs could have for domestication research would not do the topic justice. And since reviewer 2 was delighted about the inclusion of this topic ("I am certain that the study adds great value to domestication studies focused on behavioral differences across populations"), we have chosen to retain it as a point of focus. Nevertheless, your arguments were convincing in that our iteration of the topic was not differentiated enough and took up too much space. Hence, we have cut it down in line with your suggestions and only retain it as one of the arguments for studying free-ranging populations, including the needed differentiation of breed-admixture in free-roaming populations.

For instance, in the very first line in the introduction (line 45-47) it is mentioned which behavioral traits are expected to change during dog domestication but this is irrelevant to the narrative of the sentence and the rest of the paragraph. Why not just focus on the history of dog domestication and the interest of dogs in science?

- L45: the part about specific hypotheses has been removed to improve clarity. To underline for the lay person why behaviour might have been of interest to humans since early on, we did change the second part of the sentence to simply point to human-directed selection, though excluding the points to the specific hypotheses.

This line of argumentation leads me to the paragraph spanning line 76-92. I have already stated in my previous review that I largely agree that pet/breed dogs are not the best lineages of dogs for questions relating to domestication. This point has been emphasized by several large-scale genetic and behavioral studies. However, I do have to object to the statement that free-ranging dogs per se are better candidates for studying dog domestication. I suspect that the authors might be conflating the generic term free-ranging dogs with village dogs, which are independent canid lineages that predates breed dog formation. While true that the majority of the worlds’ dog population is indeed free-ranging, all free-ranging dogs are not village dogs. A significant proportion of free-ranging dogs are abandoned pet dogs and should not automatically be labeled as an independent genetic lineage without some sort of genetic evidence hereof. I also feel that the Boyko references is particularly misguided in its use in line 86 as they show that while authentic village dog lineages do exist in Africa, many dogs are heavily admixed with breed dog lineages.

- L76-92: Thank you for pointing out this important differentiation! We have changed the paragraph to point to the possible admixture more rigorously (now L73-78).

This is not to say that I disagree with the argumentation that 1) free-ranging dog populations are worth studying (they are and we should), and 2) that some of them are better representatives than breed dogs. I just think that especially point 2) should be approached with caution and maybe in this context, where the authors do not in fact have any evidence for the genetic makeup of their free-ranging dog population, should err on the side of the narrative of point 1) and simply argue that because the vast majority of the worlds dog population is free-ranging AND thus free of strong artificial selection, we should simply make the effort to develop validated methods to quantify the behaviors of these dogs. That alone is a strong and clear argument – just like in the abstract. The other narrative (i.e. point 2)) is not important to the methods presented and just adds clutter and confusion.

- We understand your point, and it has merit! We concede that we dwelled on the domestication part too much in the face of what this paper is actually about, particularly in this indicated part. Nevertheless, we do not want to overlook the value free-ranging populations can have for this topic altogether, and we see great importance in advocating for it. And since the paragraph is about the value of testing free-ranging dogs in general rather than our specific population, we argue that our current lack of knowledge about the latter’s genetics does not invalidate that point. This paper is but a starting point rather than an insight into this population in particular. Hence, we retained part of our argumentation here but shortened it and phrased it more adequately to honour the very valid point you made above about admixture interferences (see point above, L73-78).

Line 93-120 is what it is all about – rigor and repeatability, love to see it!

- Now L82-111. We are very grateful to hear that! This part has remained largely unchanged besides your comments below and the writing-related comments made by reviewer 2.

Specific comments:
Line 112: Interchanging use of free-ranging dogs and street dogs throughout the manuscript, which is a bit confusing.

- We chose to alternate between street and free-ranging dogs on purpose to differentiate between our population and free-ranging dogs overall. While we argue for the importance of considering and testing free-ranging dogs, our sample focussed on street dogs, as defined in L56 and declared in L101. Hence, we mostly use free-ranging in the intro and then switch to street dogs in the methods and results. In the discussion, we alternate depending on whether it pertains to free-ranging populations overall or whether we can only infer to (these) street dogs in particular. While we understand that this might be a bit confusing, we do not want to make overgeneralized statements that we do not have any evidence for, and we trust the readers to understand the difference after rereading the definition in case confusion does occur. Nevertheless, to further aid this understanding, we added the definition again when introducing our methods at the end of the introduction (L101).

Line 114: Which domestication hypotheses. Why is this relevant to this methods paper?

- These were initially mentioned in the first line of the paper but we have removed them from there now. Hence, we added them in brackets here (now L103).

Line 186: Seems like the authors are testing multiple behaviors at the same time here. Playfulness could be independent of sociability, no? Will sociability and play be separated in the analyses? Ah, I can see that playfulness was dropped from the analyses…

- Indeed, we were testing sociability behaviours as well as play behaviour in this subtest as indicated in L165, divided by the different phases (Human approach – play). The two behaviour categories would have been analysed separately (as suggested by the different categories in the ethogram), but as you correctly identified, play had to be excluded due to low engagement.

Line 187: My objections to using the fake dog as a test of conspecific aggression from the first review remain unchanged given the results presented (even though the issues with the test possibly quantifying neophobia and boldness instead of conspecific aggression is now lightly touched upon in line 530-532).

- We do agree that a fake dog is not the most ideal measure to assess conspecific behaviour. Its merits in this situation (safety, time-effectiveness, flexibility, standardisation) and the fact that is has been validated repeatedly, as well as the significant drawbacks (see paragraph on the fake dog (L511-521) and in the limitations (L586-589)) are discussed in the discussion and we make suggestions for better approaches (shorter time, real life observations). Since it is the goal of this paper to present our used methods and discuss their pros and cons in depth, we will retain this part in the methods as is and trust the reader to make their own final judgement depending on their situation.

Line 211: Please also see Nawroth, C., Wiesmann, K., Schlup, P. et al. Domestication and breeding objective did not shape the interpretation of physical and social cues in goats (Capra hircus). Sci Rep 13, 19098 (2023) https://doi.org/10.1038/s41598-023-46373-9 for the latest on the pointing debate.

- Thank you for this lovely read! It was very insightful. We have added it as a reference towards the point you make right below (L472).

Line 479-483: Not necessarily. These dogs could also have learned that humans mean food, which seems highly likely given that they are all scavengers. So, the behaviors the authors here associate with consistency and heritability could simply be the result of a learned behavioral process. How can the authors disentangle these concepts when they do not know the background (genetic, social etc.) of the dogs they are testing? This part is very speculative at best.

- We agree that this part is speculative, and it was not intended otherwise. We did not mean to argue that it is hereditary in our population but rather point to other papers that have provided such evidence. Regardless, you are undoubtedly correct that we cannot disentangle whether life experience or genetics are the reason for such consistency here. We have added a few sentences in the paragraph (L470-472) to bring out that point and a possible argument that might nevertheless suggest a genetic component.

Line 502-511: This is a thoughtful paragraph. I wonder if point 3) does not leave room for habituation to the test situation, which might affect behavioral measurements?

- Now L494: That is absolutely correct. We had some further elaborations towards those points initially but deleted it to decrease the word count, hoping that the reader could infer as much themselves. But we do agree that particularly for people not familiar with such testing methods, it should be pointed out. One solution might be that the time before the second testing should be substantially longer than 6 weeks, but seeing as we don't know when they would stop remembering the test, that is not ideal. Likewise, just starting from where the initial test was stopped might alter the behaviour in the subsequent subtests as well. Hence, we now added a half-sentence suggesting that the best way might be to repeat the test (for the sake of the missing subtests) but take the measures from the initial test for the subtests that are being repeated to exclude habituation. However, which approach is ultimately the best depends on the methods, goal, and testing constraints, and should thus be selected by the respective researchers themselves.

Line 565-566: The authors have not introduced the domestication syndrome until this point. The reader does not know that this syndrome, or neoteny, is and it is not relevant to the narrative. Strongly suggest deleting this part.

- Thank you for this observant comment. We did delete the reference to the DS and neoteny and simply referred to retained play behaviour as a frequent trait in domesticated animals (L552).

Line 571-575: This part is only relevant if the authors know for sure that their dog population predates breed formation and they currently do not have the genetic evidence to back this statement up. In this light I find this line of argumentation irrelevant and speculative in regard to the results presented.

- This part was taken out.

**Reviewer #2:**

I thank the Authors for addressing previous comments. The scope is much more constrained and I find it has improved the work significantly, resulting in a methods paper that domestication workers will find useful in the design of larger studies, and very informative on the challenges of behavioral studies outside of laboratory environments.

This manuscript is much improved, has better focus, and most importantly, now covers its intended scope and stays within it. The study offers a behavioral testing method for street or free-roaming dogs, and presents results on the reliability of coding and retesting in a non-laboratory environment. It is presented as the longest and most complex behavioral test for this type of setting and sample. The methods are clearly described and the protocol is well explained. The suite of tests is reported to be successful in the amount of coding reliability, and less so in the retesting consistency, but overall, is informative on the feasibility of this type of behavioral assessment and on the challenges that can be encountered. It was a very interesting read, and I learned much while doing so. I am certain that the study adds great value to domestication studies focused on behavioral differences across populations, and hopefully will serve as a starting point for larger works. Importantly, it points out certain pitfalls in standard protocols that did not work well in a street environment, all which could improve and inform future work in this area. It is very interesting that the most consistent traits were those involving human engagement/communication.

I find the Abstract needs work. The wording is awkward in several sentences, and it makes the setup of the article confusing. There were several problems with sentence structure throughout the MS. Please see the pdf where I tracked the suggested edits, as well as edits to the Abstract. Of course, these are only suggestions.

General answer to reviewer #2:

Thank you very much for this detailed effort! We have taken your suggestion seriously and changed the ms accordingly (see below for detail). Since reviewer 1 expressed his great appreciation for the abstract, we chose to keep changes in this part to a minimum – besides honouring your suggestions. We hope this has created an easier read that is satisfactory for readers from many different scopes.

A few more specific comments + In-text comments:

Abstract

- L24: lagging behind what?
  - This sentence has been changed to “has been slow” to take out the need for comparison.
- L25: THis is another confusing, awkward sentence. You need to keep the subject of the sentence clear. "no tests have been conducted on [what]..."their [subject here is the tests, not the dogs, but structure the sentence so this is clear] reliability is unknown.."
  - Thank you for pointing this out. The sentences have been restructured to provide more clarity (L24-28).
- L28; i suggest "on"
  - Accepted.
- L 40: I am not sure there is evidence of this in your paper. Is there? I agree with that your results highlight difficulties in controlling the environment, but I missed how they find evidence of "intrinsic differences between pet/street dogs.
  - We were referring particularly to the play part in the human approach test here where a standardly used play and sociability test did not work at all with the free-ranging population since they did not seem to understand this concept. This intrinsic difference might be due to both learned and potentially genetic differences, as argued in L559-562.
- L40: Reword: "bearing in mind the suggestions made in the discussion". Otherwise, sentence does not make sense. I would even start with""This test battery...." and removing the phrase before.
  - Now L41: The “bearing in mind” part has been deleted and replaced with “with some adaptations” since we did not want to advocate for this test battery in its unchanged form but do agree that the sentence structure was awkward.

Intro

- L47: should be plural
  - Accepted.
- L49: (a)if you start a phrase with "from" you need to follow up with "to"..... (b) multiple aspects of [what??] This whole phrase is vague...please specify what you are trying to say.
  - In order to shorten the intro as per reviewer 1’s request, these two sentences were merged, which in turn deleted the words you commented on here. Nevertheless, we thank you for this useful suggestion.
- L51: Again, awkward sentence structure...perhaps "there has been a surge in comparative studies..?""
  - Same as above.
- L54: I suggest either "Importantly"or "however", but not both.
  - Thank you. We chose to stick with “however”.
- L57: Wow. Very interesting.
  - Right?! Hence the need to include them more.
- L63: here you imply this is compared to something...compared to [what?]
  - Useful observation, thank you. For the sake or decreasing wordiness, we simply deleted this word.
- L84: You would need to specify what "this" is, here.
  - As above, this part has been rewritten for the sake of wordiness and better nuancing.
- L87; should be plural
  - Correct, thank you. However, this was deleted in line with reviewer 1’s comment about nuancing this paragraph.
- L93: I suggest "on"
  - Accepted.
- L95: "in which they reside". I strongly encourage never ending a sentence with a preposition.
  - A valuable suggestion, thank you. Accepted.
- L103: rater's.
  - Accepted.
- L111: Grammatical suggestion: either (a) you "address" an issue or problem... (b) or you "have" objectives.
  - We have changed this to “achieve an objective”.
- L120: "to be used more.."
  - Since we do not only refer to a higher frequency but also a more widespread use, we changed this phrasing to "more widely used".

Methods

- L202: this is not the definition of neophobia, correct? I suggest correcting this, it is confusing. // Did the authors err in their definition of “neophobia”? Or is it perhaps that in the writing style it seems as if they misinterpret its meaning? Please check. (Methods section).
  - Thank you for pointing this out. It was indeed not meant as the definition but rather as a clarification for all the assessable target behaviours. The line has been written more clearly (L193).

Discussion

- L485: this sentence is unclear...you have differences between 3 things here...did you mean between 2 things?
  - This line was indeed confusing, thank you for catching that awkward phrasing. We deleted the obsolete part (L474).
- L499: do not
  - Since we referred to the begging test here, the grammar was correct. However, we see how the sentence can be confusing, so we added this part in brackets to refer to the begging test specifically rather than have it as a half sentence (L486).
- L555: 1. Line 555 (Discussion): I am not sure how one could justify making the statement that “behavioral plasticity is selected against in modern [dog] breeds. But if made, please provide a citation or source. // Please provide a citation or supportive evidence for this from a source. I am not sure how one could justify making this statement that behavioral plasticity is selected against in modern breeds.
  - We do not mean to suggest that it was consciously selected against but rather that it might have been decreased through the selection for stable behavioural traits and the loss of genetic variation associated with that. This has been rewritten accordingly to make that point clearer and references have been added for the latter two points (L540-542).
- L624: Reword to: "Bearing in mind the suggestions made..."
  - Accepted.
