# Supplementary material for: Street-wise dog testing: Feasibility and reliability of a behavioural test battery for free-ranging dogs in their natural habitat
Source: PLoS One. 2024 Mar 14;19(3):e0296509. doi: 10.1371/journal.pone.0296509 (PMC10939227; doi:10.1371/journal.pone.0296509)
Supplement: Attachment — Submitted filename: Review 2_Reviewer3 Dec 2023.docx [file pone.0296509.s013.docx]

Dear Edito and Authors,

Thank you for letting me review this work and making the changes suggested in previous revisions.

The manuscript is clearer, has better direction and accomplishes its goals of providing a baseline for behavioral tests of street/free-ranging dogs. It was very interesting to see how these tests are conducted, what the challenges are, and that they can, in fact, provide reliable information about certain behaviors in the variable conditions outside the “pet” environment.

I suggest some minor edits that will simply clarify that this is not a domestication study, but a behavioral study in the street environment. It will be informative to domestication workers to compare results from this model, but it is not a domestication itself. My apologies if this was not clear before, or if I caused any confusion.

The edits should be quick.

I am happy to clarify anything, if needed, I sometimes write quickly (!).

Best wishes,

REVIEW DETAILS:

Lines76-78. Please see my comment on distinguishing between this study and domestication research:

In order not to be misleading, I suggest changing to something akin to:

"…this study provides insights for comparison with domestication research…"

This is *not* a domestication study.

I repeat that it will add value to domestication research by providing a comparative model from street populations, but it is not an insight into domestication work itself.

Line 539. This is rather an important conclusion/position. I wonder if there is a citation here, or if the authors are the first to find this?

Line 599. Conclusions. Again, I think the study is valuable because one can compare a street model vs a domestication model, but is not suitable for "testing questions about domestication" itself. This distinction must be clear, and should not take away from the value of the study.

Note: small grammatical edits marked in the pdf.
