# Supplementary material for: Street-wise dog testing: Feasibility and reliability of a behavioural test battery for free-ranging dogs in their natural habitat
Source: PLoS One. 2024 Mar 14;19(3):e0296509. doi: 10.1371/journal.pone.0296509 (PMC10939227; doi:10.1371/journal.pone.0296509)
Supplement: Attachment — Submitted filename: DSAdult22_PlosOne_Itemized Answers.docx [file pone.0296509.s014.docx]

Dear Edito and Authors,

Thank you for letting me review this work and making the changes suggested in previous revisions.

The manuscript is clearer, has better direction and accomplishes its goals of providing a baseline for behavioral tests of street/free-ranging dogs. It was very interesting to see how these tests are conducted, what the challenges are, and that they can, in fact, provide reliable information about certain behaviors in the variable conditions outside the “pet” environment.

I suggest some minor edits that will simply clarify that this is not a domestication study, but a behavioral study in the street environment. It will be informative to domestication workers to compare results from this model, but it is not a domestication itself. My apologies if this was not clear before, or if I caused any confusion.

The edits should be quick.

I am happy to clarify anything, if needed, I sometimes write quickly (!).

Best wishes,

GENERAL ANSWER:

Thank you very much for the kind words and the clarifications. Since it seems to be quite controverse and easy to misunderstand, we decided to take out the domestication part from the conclusions. The suggested edits have been made in the rest of the manuscript and the question about the citation has been answered below. We greatly appreciate your diligent work and your constructive suggestions along this paper’s journey!

REVIEW DETAILS:

Lines76-78. Please see my comment on distinguishing between this study and domestication research:

In order not to be misleading, I suggest changing to something akin to:

"…this study provides insights for comparison with domestication research…"

This is *not* a domestication study.

I repeat that it will add value to domestication research by providing a comparative model from street populations, but it is not an insight into domestication work itself.

As described above, we decided to take out the point about domestication from the conclusions entirely in order to not mislead the reader or add to many words for clarification.

Line 428. I think you did not mean this "and" here? PLease ignore if I am wrong

A very good catch, thank you very much! The “and” has been deleted

Line 505: YOu may wish to correct the run-on structure here.

Thank you, this has been changed for a better reading experience.

Line 539. This is rather an important conclusion/position. I wonder if there is a citation here, or if the authors are the first to find this?

This is solely a speculative suggestion we make based on our observations, hence the lack of citation. We made this a bit clearer in the text now. So far, there is very little research into behavioural flexibility in dogs (to our knowledge) apart from the meta-analysis in breed dogs cited below. Further research testing this idea would therefore be a very interesting avenue.

Line 546: question "of" whether...

Changed accordingly

Line 599. Conclusions. Again, I think the study is valuable because one can compare a street model vs a domestication model, but is not suitable for "testing questions about domestication" itself. This distinction must be clear, and should not take away from the value of the study.

Now Line 602: We agree that this was phrase a bit too undifferentiated. The suggested specifications have been implemented.
